# Age, stage and senescence in plants

**DOI:** 10.1111/1365-2745.12088

**Published:** 2013-04-24

**Authors:** Hal Caswell, Roberto Salguero-Gómez

**Affiliations:** 1 Biology Department MS-34, Woods Hole Oceanographic InstitutionWoods Hole, MA, 02543, USA; 2 Max Planck Institute for Demographic ResearchRostock, 18057, Germany; 3 School of Biological Sciences, University of QueenslandBrisbane, Qld, 4072, Australia

**Keywords:** ageing, ComPADRe III database, matrix population models, plant development and life history traits, selection gradients, sensitivity, stage-structured demography, vec-permutation matrix

## Abstract

**1.** Senescence (an increase in the mortality rate or force of mortality, or a decrease in fertility, with increasing age) is a widespread phenomenon. Theories about the evolution of senescence have long focused on the age trajectories of the selection gradients on mortality and fertility. In purely age-classified models, these selection gradients are non-increasing with age, implying that traits expressed early in life have a greater impact on fitness than traits expressed later in life. This pattern leads inevitably to the evolution of senescence if there are trade-offs between early and late performance.

**2.** It has long been suspected that the stage- or size-dependent demography typical of plants might change these conclusions. In this paper, we develop a model that includes both stage- and age-dependence and derive the age-dependent, stage-dependent and age×stage-dependent selection gradients on mortality and fertility.

**3.** We applied this model to stage-classified population projection matrices for 36 species of plants, from a wide variety of growth forms (from mosses to trees) and habitats.

**4.** We found that the age-specific selection gradients within a life cycle stage can exhibit increases with age (we call these *contra-senescent* selection gradients). In later stages, often large size classes in plant demography, the duration of these contra-senescent gradients can exceed the life expectancy by several fold.

**5.** *Synthesis*. The interaction of age- and stage-dependence in plants leads to selection pressures on senescence fundamentally different from those found in previous, age-classified theories. This result may explain the observation that large plants seem less subject to senescence than most kinds of animals. The methods presented here can lead to improved analysis of both age-dependent and stage-dependent demographic properties of plant populations.

## Introduction

Senescence refers to an increase in the mortality rate, also called the force of mortality, and/or a decline in fertility, with advancing age. Theories to explain the evolution of senescence have often focused on the age-dependence of the selection gradients on mortality and fertility. These theories were originally proposed by Medawar (1952) and Williams (1957), before being fully developed by Hamilton (1966). Hamilton showed that the selection gradient on age-specific mortality is non-increasing with age and strictly decreasing after the age of first reproduction. The selection gradient on fertility is strictly decreasing with age as long as the population is not declining. In other words, changes in mortality or fertility that affect older age classes have less of an impact on fitness than the same changes happening at earlier ages. Calculations of the selection gradients for a variety of organisms (Caswell 1978) showed that the differences with age could span many orders of magnitude. Hamilton's results imply that the detrimental effects of a trait that increases mortality, or reduces fertility, at late ages can be counteracted by much smaller reductions in mortality, or increases in fertility, at earlier ages. Thus, selection will favour traits, or pleiotropic interactions between traits, or accumulations of detrimental mutations that lead to negative effects on mortality and fertility at older ages, given such age-specific effects. These predictions have been studied both theoretically and empirically for many years (e.g., Rose 1991; Charlesworth 1994, 2001; Tuljapurkar 1997; Kirkwood & Austad 2000; Baudisch 2005, 2008).

To clarify terminology, we use the term selection gradient here to refer to the directional selection gradient as defined in quantitative genetics – as a vector of partial derivatives of fitness with respect to the trait values (Lande 1982) – or, equivalently, as a vector of partial regression coefficients of fitness on the traits (Lande & Arnold 1983). The selection gradient also appears in the theory of adaptive dynamics as a vector of partial derivatives of invasion fitness on trait values (Dieckmann & Law 1996; Dercole & Rinaldi 2008). The response to selection depends on the interaction between the selection gradient and some description of the pattern of variances and covariances on which selection can operate, as Wright's equation in population genetics (1931), the multivariate breeder's equation in quantitative genetics (Lande & Arnold 1983), the Price equation (Price 1970; see Frank 1995; Day & Gandon 2007) and the canonical equation of adaptive dynamics (Dercole & Rinaldi 2008). The selection gradient was referred to by other names in the early literature; for example, the selection intensity or selection pressure (Emlen 1970), or the force of selection (Hamilton 1966).

Because of the role of the decline in selection gradients in this theory, the results have been called 'slope theorems' (Caswell 1982a). The tendency to evolve senescence is proportional to the slope, on a logarithmic scale, of the selection gradient (Caswell 1982a). We will refer to the negative slope implied by Hamilton's results as a *pro-senescent* selection gradient. A selection gradient that increases with age will be referred to as an *anti-senescent* selection gradient. Having shown that selection gradients on mortality and fertility are pro-senescent, W. D. Hamilton famously concluded that the evolution of senescence was ‘an inevitable outcome of evolution’ (1966, p. 1966). He was, in one sense, correct: given the pro-senescent selection gradients, any life history is invadable by traits that pay for reduced mortality or increased fertility early in life with increased mortality or reduced fertility late in life; that is, traits that include trade-offs between early and late life.

Hamilton's theory has been the subject of intense discussion, and two ways to avoid the conclusion of inevitable senescence have been noted. One is to assume some other kinds of trade-offs; for example, between mortality and fertility, or generated by allocation of energy (Tuljapurkar 1997; Baudisch 2008). Another is to focus on traits that modify mortality or fertility in other ways (Baudisch 2005); the selection gradients on traits that produce proportional changes in mortality or fertility are not necessarily monotonically decreasing with age.

Plants, perhaps because of their modularity (their architecture consists of a repetition of units, or modules; Harper 1980), have long been suspected of violating Hamiltonian predictions of senescence. Some species, including large trees (Lanner & Connor 2001; Issartel & Coiffard 2011) and some clonal genets (Peñuelas & Munné-Bosch 2010), live for very long periods of time. Harper (1977, p. 702) proposed that 'plants with clonal growth show no apparent senescence'. He suggested that this might be due to their indeterminate growth, because of which their demography might be more dependent on size or developmental stage than on age. Hamilton's results follow from an age-classified model, but age alone is generally a poor individual state (*i*-state) variable for plant demography (Caswell 2001). Early studies of the selection gradients on *stage-specific* survival and fertility showed that these gradients were not monotonic functions (Caswell 1982b), and it was suggested that these results might weaken or even remove altogether the selection pressure for senescence ( Caswell 1982b1982b, 1985). Vaupel *et al*. (2004) developed an optimization model based on energy allocation in a size-classified species and demonstrated that plastic growth could lead to negative senescence. Indeed, evidence of increasing fertility and decreasing mortality rate has been recently found in a long-lived herbaceous perennial species (García, Dahlgren & Ehrlén 2011).

Stage-classified demography leads to non-monotonic selection gradients, but stage is not age. Senescence refers specifically to age-dependent changes in the vital rates. Given the undeniable importance of size and stage for plant demography, what is needed is a demographic theory that can produce selection gradients on traits whose effects are jointly dependent on the age and stage of an individual.

In this paper, we develop such an analysis, based on matrix population models classified by both age and stage, using an approach introduced by Caswell (2012). This approach implements ideas about multistage demography originating in multi-state demographic models (e.g. Goodman 1969; Law 1983; Csetenyi & Logofet 1989; Lebreton 1996). We will derive the selection gradients on mortality and fertility from the model and analyse a selection of plant species of different taxa, growth forms and habits. We will demonstrate that the resulting selection gradients can differ fundamentally from those produced by age-dependent demography alone and that plant species may experience contra-senescent selection gradients for a significant part of their life cycle.

To clarify terminology, we will refer to three kinds of traits:

*Age-dependent:* a trait that affects all individuals of a given age, regardless of their stage. Such traits are the basis of our usual understanding of senescence.

*Stage-dependent:* a trait that affects all individuals in a given stage, regardless of their age. Such traits could lead to stage-dependent trade-offs, but are not strictly speaking relevant to senescence.

*Age*×*stage-dependent:* a trait that affects individuals on the basis of their joint age and stage status.

The selection gradients on age×stage-dependent traits would produce stage-specific patterns of age-dependent trade-offs. Senescence would become a property that would differ between stages in the life cycle of the species. The existence of stage-specific traits is not in doubt; plants exhibit many such traits, especially relating to reproduction, growth, shrinkage and vegetative dormancy. Thus, our analysis would lead to a theory of senescence in which gene action depends on both developmental stage and age.

## The model

The structure of the age-stage model employed here is described in detail in Caswell (2012). The symbols used are listed for convenience in Table 1. We let *s* denote the number of stages and ***ω*** the number of age classes. Demography is defined by a set of stage-specific matrices for each age:


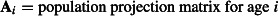
eqn 1


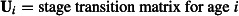
eqn 2


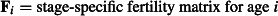
eqn 3

where 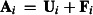, for *i* = 1,…,***ω***.

**Table 1:** Mathematical notation used in this paper. Dimensions are shown, where relevant, for matrices and vectors; *s* denotes the number of stages and *ω* the number of age classes

Quantity	Description	Dimension
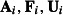	Stage-classified projection, fertility and transition matrices for age class *i*.	*s* × *s*
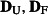	Age transition matrices for individuals already present in the population and for new individuals produced by reproduction.	*ω* × *ω*
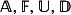	Block diagonal matrices.	*sω* × *sω*
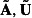 , etc.	Age-stage matrices constructed from block diagonal matrices using the vec-permutation matrix.	*sω* × *sω*
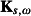 , **K**	Vec-permutation matrix	*sω* × *sω*
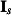	Identity matrix	*s* × *s*
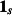	Vector of ones	*s* × 1
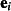	The *i*th unit vector, with a 1 in the *i*th entry and zeros elsewhere.	various
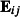	A matrix with a 1 in the (*i*,*j*) position, and zeros elsewhere.	various
⊗	Kronecker product	
∘	Hadamard, or element-by-element, product	
vec**X**	The vec operator, which stacks the columns of a *m*×*n* matrix **X** into a *mn*×1 vector.	
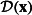	A diagonal matrix with **x** on the diagonal and zeros elsewhere.	

As in Caswell (2012), we will consider models created from a single stage-classified matrix. In such a model, individuals grow older, but their vital rates are affected only by their stage (as specified in **A**). These calculations are thus comparable with the various 'age-from-stage' calculations recently developed using Markov chains (Caswell 2001, 2006, 2009; Tuljapurkar & Horvitz 2006; Horvitz & Tuljapurkar 2008). We return to this in the Discussion.

In this model, ageing is described separately for extant individuals and for individuals newly produced by reproduction. At each time step, extant individuals are moved to the next age class by an ***ω***×***ω*** age transition matrix 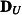. For example, if there are four age classes, 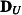 is


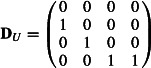
eqn 4

The ones on the subdiagonal move individuals to the next age class; the one in the lower right corner means that the last age class contains individuals age ***ω*** or older.

At each time step, individuals that are newly produced by reproduction are all placed into the first age class by an ***ω***×***ω*** fertility allocation matrix 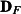. Again assuming four age classes as an example,


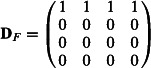
eqn 5

The population is described by a distribution of both age and stage; its state can be described at any time by the matrix


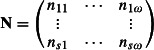
eqn 6

in which rows denote stages and columns denote age classes. The population vector **n** is created by applying the vec operator to **N**, which stacks the columns, one above the next


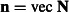
eqn 7

This vector groups stages within age classes. The vec-permutation matrix 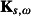 (which we denote as **K** when it is unnecessary to specify the dimensions) rearranges the entries of **n** to group age classes together within stages,


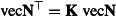
eqn 8

(Henderson & Searle 1981; a simple algorithm for calculating **K** is given in Hunter & Caswell 2005).

Between *t* and *t*+1, the model first allows individuals to move between stages, while remaining within their age classes. Then, the process of ageing moves individuals from one age class to the next. The vec-permutation matrix rearranges the population vector for each step (Hunter & Caswell 2005).

To create the projection matrix needed to project the vector **n** from *t* to *t*+1, we generate a set of block diagonal matrices for stage transitions, reproduction and ageing. For example, the transition matrices 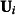 for the stage transitions of extant individuals are combined into a block matrix, for example, if there are four age classes


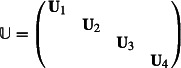
eqn 9

with similar block diagonal matrices 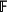, 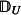 and 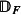. Given these components, the population projection matrix is


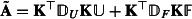
eqn 10

In the first term, 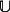 implements the stage-specific demography within each age class, **K** permutes the vector, 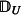 moves the individuals to the next oldest age class within each stage and then 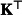 returns the vector to its original form. The second term does the same for individuals newly produced by reproduction, with 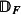 placing all new individuals into the first age class within each stage.

The population age-stage vector **n** is projected from *t* to *t*+1 as


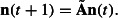
eqn 11

The population growth rate, stable age-stage distribution and reproductive value distribution are given by the dominant eigenvalue ***λ*** and corresponding right and left eigenvectors, **w** and **v**, of 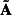. The invasion exponent, measuring fitness, can be taken as either ***λ*** or (slightly more appropriate) the rate of increase *r* = log ***λ***.

## Selection gradients on age- and stage-specific traits

The selection gradient on a trait vector ***θ*** is given by


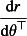
eqn 12

We will develop selection gradients for a trait vector ***μ*** of mortality rates and a vector ***ϕ*** of fertilities. To do so, we use the matrix calculus approach introduced and described in Caswell (2007, 2008). In this notation, the derivative of the *m*×1 vector **y** with respect to the *n*×1 vector **x** is written as 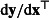 and is the *m*×*n* matrix whose (*i*,*j*) entry is the derivative of 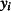 with respect to 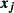:


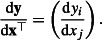
eqn 13

Thus 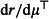 and 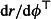 are row vectors whose entries are the derivatives of *r* with respect to mortality rates and fertilities, respectively.

### Mortality

We describe mortality by a vector ***μ*** of stage-specific mortality rates. Perturbations of ***μ*** are additive changes in mortality rates; we know from Hamilton (1966) that the selection gradient for such traits declines with age. To incorporate these rates into our model, we define a vector ***σ*** of stage-specific survival probabilities, given by ***σ*** = exp(−***μ***), where the exponential is applied element-wise. The transition matrix **U** can then be written


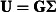
eqn 14

where **G** is a matrix of transition probabilities conditional on survival, and 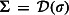 is a diagonal matrix of stage-specific survival probabilities.

### Fertility

In age-classified models, fertility always appears as the first row in the projection matrix, because reproduction always produces individuals in the first age class. In contrast, stage-classified demographic models, especially for plants, can include multiple types of offspring (e.g. dormant and germinating seeds, or several size classes of seedlings; e.g. Meagher 1982; Liu, Menges & Quintana-Ascencio 2005). Thus, perturbation of fertility must account for the possibility of multiple offspring types. To satisfy this requirement, we write fertility at age *i* in terms of a *s*×1 perturbation parameter vector ***ϕ***, by defining


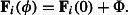
eqn 15

Here, 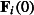 is the unperturbed fertility matrix at age *i*. The matrix **Φ** describes the perturbations. It contains 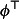 in each row that represents, in 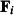, a type of offspring, and zeros elsewhere. For example, if the life cycle contains three stages, the first of which is offspring, then


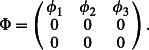
eqn 16

If the first two stages were types of new offspring, then **Φ** would contain 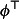 in each of the first two rows, and so on.

Derivatives of ***λ*** with respect to 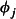 give the effects of additive changes to the production of offspring (of all types) by stage *j*. This calculation gives the selection gradient corresponding to the standard age-classified sensitivity analysis, in which there is only a single type of offspring, but also accommodates the common phenomenon of multiple types of offspring in stage-classified models.

The matrix **Φ** can be written by defining a vector **z** as


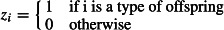
eqn 17

and then calculating


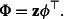
eqn 18

### Selection gradients

The selection gradient on the stage-specific mortality vector at age *i* is


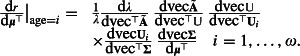
eqn 19

The selection gradient on the fertility vector at age *i* is


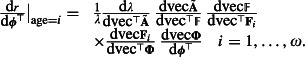
eqn 20

Expressions for each of the terms in (19) and (20) are given in Appendix A. The results of these calculations are two-dimensional arrays, giving the selection gradients on age× stage-dependent mortality and fertility, respectively. The selection gradients on the age-dependent traits are obtained by summing these arrays over all stages, and the gradients on stage-dependent traits are obtained by summing over all ages. Comparing the age-stage results with the age-specific and stage-specific results reveals the interaction between age and stage-dependent demographic processes.

### An application: selection gradients in *Arisaema serratum*

As an example, we consider a stage-classified model for the forest understorey perennial herb *Arisaema serratum* (Thunb.) Schott (Araceae), from Kinoshita (1987). The model is stage-classified, with stages defined by size (pseudo-stem diameter at ground level) and a combination of development and sex (seedling, juvenile, male and female). In individuals of this genus, a size threshold for reproduction defines a switch between non-reproductive (juvenile) and reproductive (male or female) status. Furthermore, sex is strongly correlated with size, and the same individual can alternate between non-reproductive, male and female status depending on the resources stored during the previous year (Bierzychudek 1982).

The resulting matrix model contains *s* = 19 stages; the population projection matrix is given in Table 3 of Kinoshita (1987). The population is close to replacement, with a growth rate of ***λ*** = 0.99. We constructed the age-stage model by using 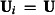 and 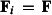, for all *i*, choosing ***ω*** so that at least 99% of the stable age-stage distribution is captured in the first ***ω***−1 age classes; in this case, ***ω*** = 27 years.

We calculated the selection gradients on age×stage-dependent mortality and fertility, using eqns (19) and (20), and calculated the age-dependent and stage-dependent gradients by summing over stage and age, respectively.

**Fig. 1:**
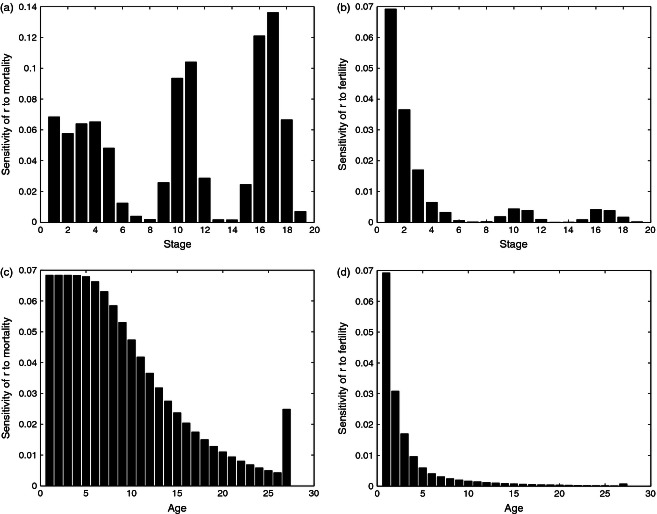
The selection gradients on mortality and fertility for *Arisaema serratum*. (a) Selection gradient on stage-specific mortality. (b) On stage-specific fertility. (c) On age-specific mortality. (d) On age-specific fertility.

As can be seen in Fig. 1, the selection gradients on stage-specific mortality and fertility are far from monotonic at later stages. The stage-specific selection gradients for mortality in the seedling stage (class 1) and juvenile stages (classes 2–7) are similar. However, the maxima for male and female stages occur at intermediate sizes. The stage-specific selection gradients for fertility are smaller than those for mortality and reach maximum values in the seedling stage (not surprisingly, as this gives the effect of a perturbation that produces extreme precocious maturation, and thus a dramatic impact on fitness).

Unlike the stage-specific selection gradients, the selection gradients on age-specific mortality and fertility are monotonic non-increasing (except, of course, for the open age interval containing individuals of age greater than or equal to ***ω***) and thus pro-senescent, over the lifespan of the species (Fig. 1c, d). The gradient for mortality follows an inverse sigmoid pattern, while the gradient for fertility exhibits a sharp exponential decay. Thus, selection on strictly age-dependent traits would be expected to lead to the evolution of senescence under Hamilton's (1966) scenario, regardless of the stage-dependence of the vital rates.

**Fig. 2:**
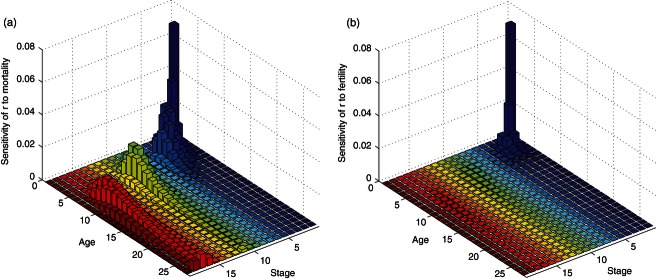
The selection gradients on age×stage-dependent mortality (a) and fertility (b) in *Arisaema serratum*. The stages have been plotted in reverse order so that the curves are more visible.

Quite a different picture emerges from the age× stage-dependent selection gradients (Fig. 2). The selection gradients on mortality *within stages* increase with age, that is, are contra-senescent, before eventually declining with age. This pattern is especially prominent in the larger size classes. Thus, age× stage-dependent traits in *Arisaema* should delay senescence up to the critical age where the selection gradient reaches its maximum and begins to decline.

**Fig. 3:**
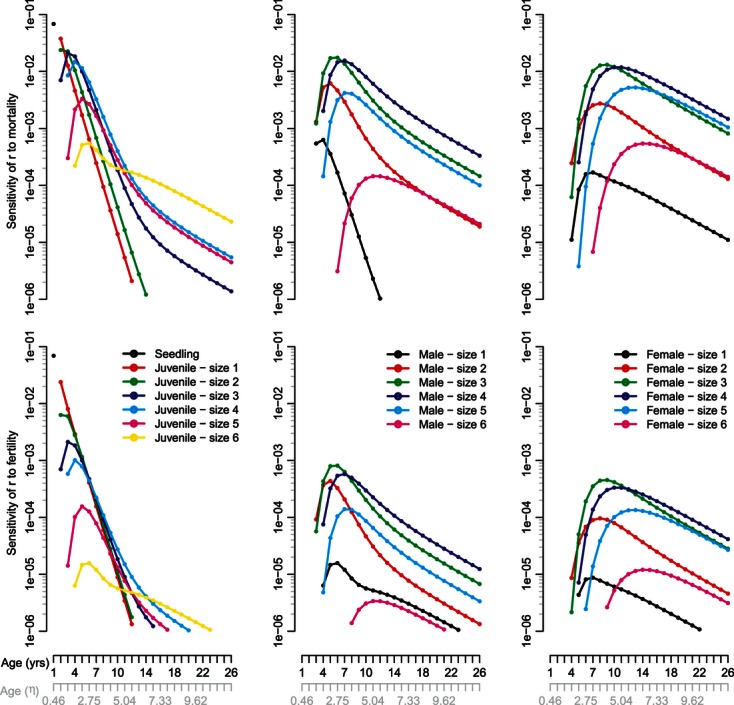
Selection gradients on mortality and on fertility for *Arisaema serratum* as a function of age and stage for juveniles, males and females. The *x*-axis shows both calendar age and age in units of life expectancy (*η*) of the first non-seedbank stage.

**Fig. 4:**
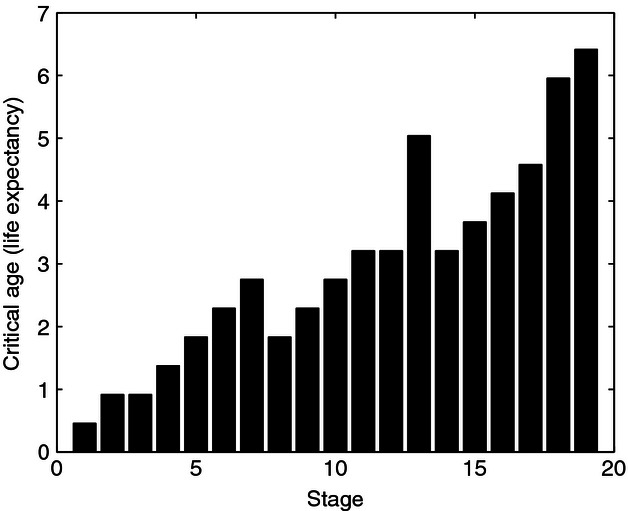
The critical age that separates contra-senescent and pro-senescent selection gradients for each stage of *Arisaema serratum*. Age is measured in units of life expectancy (*η*) of the first non-seed stage.

Figure 3 shows the same results in more detail. The slopes of the lines on this semi-logarithmic plot directly determine the size of the effect that can be accommodated by the pro- or contra-senescent selection gradient (Caswell 1982a). The fact that the slope (positive) is steeper in the contra-senescent portion than is the slope (negative) in the pro-senescent portion is an indication of the relative strength of selection against and for senescence, respectively. The peaks in these curves define the critical ages that separate the pro- and contra-senescent portions of the selection gradients. These critical ages range from 2 to 14 years. To interpret this pattern, and facilitate comparison across taxa, we have rescaled the critical ages relative to a demographically relevant duration (Baudisch 2011, Baudisch *et al*. 2013). In age-classified models, life expectancy at birth provides such a scale. Here, to avoid distortions caused by our poor understanding of lifespan in seedbanks (Baskin & Baskin 2001), we have used the life expectancy of the first non-seed stage (*FNSS*, hereafter) as a relevant scaling factor for age. We denote this life expectancy as ***η*** and calculate it from the matrix **U** using the methods presented in Caswell (2001, 2009). The results are shown in Fig. 4. Life expectancy for seedlings of *Arisaema* is ***η*** = 2.2 years. Thus, the larger stages of *Arisaema* experience contra-senescent selection gradients for as much as 6.5 times the average longevity of an individual from the *FNSS*.

## Selection gradients in a repertoire of plant species

The species analysed in the previous section, *A. serratum*, is an herbaceous perennial from a temperate forest. To extend the reach of our analyses, we carried out the same analysis on 35 other plant species (and one brown alga; see Appendix S1), of a wide range of growth habits. Data were obtained from published as well as personally communicated studies containing population matrix models for *c*. 900 plant species compiled in a database (ComPADRe III) under development at the Max Planck Institute for Demographic Research (R. Salguero-Gómez, unpubl. data). For each species, we used a projection matrix under control conditions (if relevant to the study), calculated as a mean (again, if relevant) over years and populations. Matrices obtained under experimental manipulations were excluded in these calculations to report on selection gradients under normal conditions. We then decomposed **A** into **U** and **F**, the latter including both sexual and asexual reproduction. In all cases, the stage-dependent survival probabilities 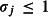, except in the perennial herb (*Chamaecrista keyensis*), where stages *j* = 4,5,6,12 slightly exceeded 1. In this case, we standardized rescaled corresponding columns of **U** so that 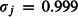 (See Appendix S1).

**Fig. 5:**
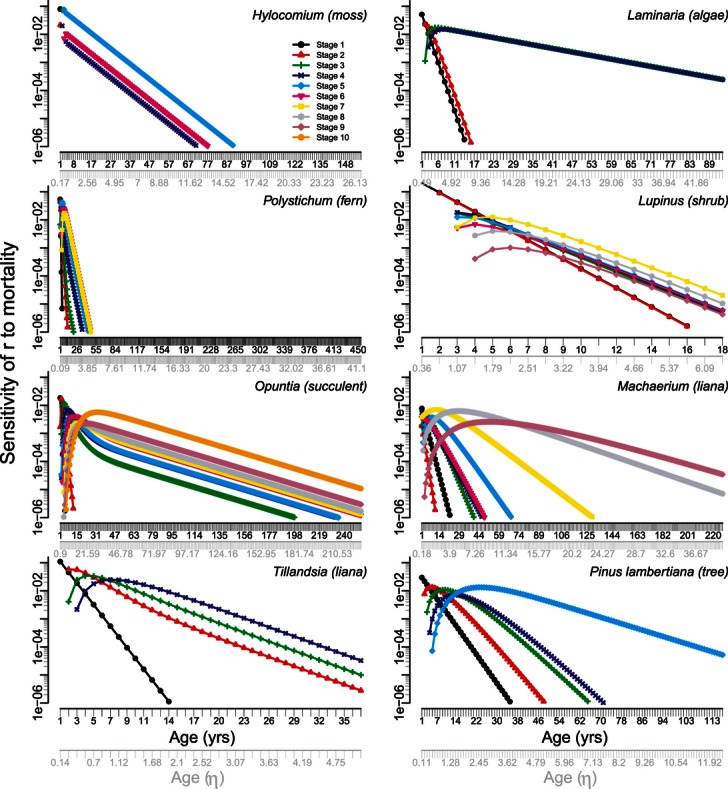
Selection gradients on age×stage-dependent mortality for a subset of the 36 species studied in this manuscript (see Appendix S1). The age axis is shown in years and in units of life expectancy (*η*) of the first non-seed stage. In all cases, gradients have been truncated at 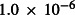.

In Fig. 5, we show the age×stage-dependent selection gradients on mortality for a selection of species: a moss (*Hylocomium splendens*), a green alga (*Laminaria digitata*), a fern (*Polystichum aculeatum*), a shrub (*Lupinus arboreus*), a succulent (*Opuntia rastrera*), a liana (*Machaerium cuspidatum*), an epiphyte (*Tillandsia recurvata*) and a tree (*Pinus lambertiana*). We have compiled the complete graphical results for all 36 species in the Appendix S1.

**Fig. 6:**
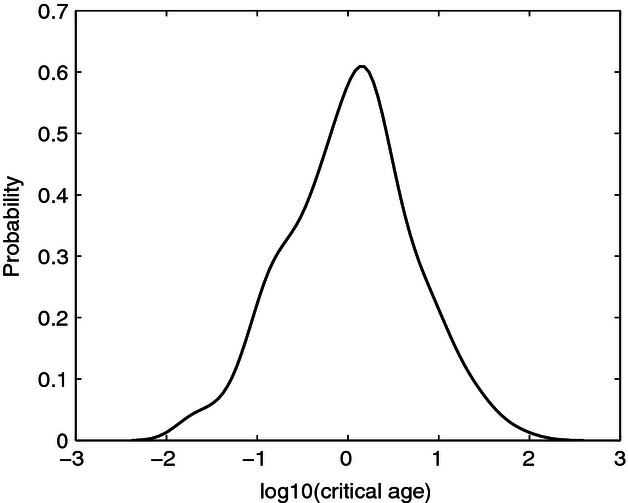
The probability density function of logarithm of the critical age, in units of life expectancy (*η*) of the first non-seed stage, among all stages of 36 species of plants. Generated using a Gaussian kernel smoother.

Figure 6 shows the distribution of the critical ages, rescaled by life expectancy ***η***, for all stages of all 36 species analysed in Appendix S1. The median critical age is 1.2 life expectancies, and the mean is 3.5, with a standard deviation of 8.1. It is apparent from these figures that many plant species experience contra-senescent selection gradients in some stages of their life cycle and that those gradients persist for times on the order of 1–10 life expectancies (see the Appendix S1 for details of each species).

Within each species, there is some overlap among the selection gradients within each of the stages. The smaller the degree of this overlap, the more selection will be stage-specific. Quantifying this overlap and analysing its consequences are open problems.

## Discussion

The beauty of Hamilton's (1966) theory of senescence is that it provides a baseline – age-classified demography, additive perturbations and trade-offs between mortality or fertility at earlier and later ages – against which alternatives must be compared. The age trajectory of the selection gradient plays a central role in the theory of senescence (e.g. Hamilton 1966; Charlesworth 1994, 2001; Tuljapurkar 1997). As Charlesworth (2000, p. 930) put it, 'Our understanding of the evolution of senescence is, at one level, very complete; we know that senescence is an evolutionary response to the diminishing effectiveness of selection with age and that this explains many aspects of the comparative biology of senescence'. (He goes on to say that it is less clear what genetic mechanisms are involved.) This highlights the importance of understanding the patterns of age-dependent, stage-dependent and age×stage-dependent selection gradients in species, like plants, that differ from humans in their plastic growth, complex life history or modular morphology.

We have found that, in most cases, age×stage-dependent traits experience very different selection gradients than do the strictly age-dependent traits considered by Hamilton (1966) and most of the literature on the evolution of senescence. Up until some critical age, often greater than the life expectancy of the study species, age×stage-dependent traits experience contra-senescent, rather than pro-senescent selection (Fig. 6). This finding provides some support for the idea that senescence does operate differently in the larger and later stages of plant life histories (e.g. Hibbs 1979; Harcombe & Marks 1983; Greenwood 1987). The high phenotypic plasticity displayed in the development of plants (Bradshaw 1984; Schlichting 1986; Pigliucci, Murren & Schlichting 2006; Magyar *et al*. 2007), whereby adults of some species can 'rejuvenate' when trimmed (Hackett 1985; Crane, Schaffer & Davenport 1992) or grafted (Huang *et al*. 1992), and large individuals can naturally lose over 80% of their aboveground biomass, shrinking to very small size classes (Golubov *et al*. 1999), provides great opportunities for the operation of stage-dependent traits. It may also provide an opportunity for these modular organisms to 'reset' the ageing clock (Salguero-Gómez & Casper 2010).

Further study of age×stage-dependent traits would be useful in the study of senescence. Baudisch (2005) showed that the selection gradients on age-dependent traits depend on whether the traits affect mortality or fertility in additive or proportional fashion. In this paper, we have examined only additive perturbations of mortality and fertility, because that is where the most powerful comparison with age-dependent theory can be made. The analysis of proportional perturbations remains an open problem.

In our analyses, the age×stage-dependent model is constructed from a single stage-specific projection matrix. As a result, the selection gradients on mortality and fertility within a given stage are proportional (except in cases where no survival is possible within a stage; H. Caswell, unpubl. data). A genuinely age- and stage- classified model, in which stage-specific rates were estimated at each age, would not be restricted in this way. To our knowledge, only one such model has ever been reported (van Groenendael & Slim 1988). Such models require, obviously, more data than a purely stage-classified model, because they would require estimates of the vital rates as a function of both age and size. The development of such models would greatly extend our understanding of the selective pressures on senescence (and other life history traits) in plants (see Shefferson & Roach 2013 for an example of a way to combine age and stage data). The model structure reported here (and in Caswell 2012) makes it much easier to develop such models, as well as other models including multiple structures (e.g. a mathematically rigorous version of the second-order model of Ehrlén (2000). Hopefully, this will encourage the analysis of age×stage-dependent data that may already exist.

## Appendix A

### Derivation of selection gradients

The selection gradients on age×stage-dependent mortality and fertility are given by eqns (19) and (20), respectively. These expressions are an exercise in the chain rule for matrix calculus (Magnus & Neudecker 1985). Ecological presentations of the basics of matrix calculus can be found in Caswell (2007, 2008, 2009). In this Appendix, we derive each of these terms.

The derivative of ***λ*** with respect to the entries of  is given in Caswell (2010) as

eqn 21

where **w** and **v** are the right and left eigenvectors corresponding to ***λ*** and ⊗ denotes the Kronecker product. The eigenvectors are scaled so that . This is the matrix calculus counterpart of the familiar expression  (Caswell 2001).

The derivative of  with respect to  is given by equations 22 and 24 of Caswell (2012) as

eqn 22

where  is a (*n*×*n*) identity matrix and  is a matrix with 1 in the (*i*,*i*) entry and zeros elsewhere. The derivative of  with respect to  is similar,

eqn 23

The derivative of  with respect to the vector of mortality rates ***μ*** is obtained by taking the differential of 14,

eqn 24

and applying the vec operator,

eqn 25

Taking the differential of  and applying the vec operator gives

eqn 26

The differential ; thus, the final result is

eqn 27

As expected, the derivative with respect to mortality is negative. To avoid the inconvenience of plotting negative numbers, in this paper, we have plotted the sensitivity with respect to *reductions* in mortality.

The derivative of  with respect to the fertility vector ***ϕ*** is obtained from 18 by noting that

eqn 28

eqn 29

Combining all these steps as in 19 and 20 gives

eqn 30

eqn 31

While these expressions are impressive at first, they are easily evaluated in matrix-oriented languages such as matlab.

## Supporting Information

**Appendix S1.** Age- and stage-specific selection gradients for 36 selected plant species.
