# Supplementary material for: Age, stage and senescence in plants
Source: J Ecol. 2013 Apr 24;101(3):585–95. doi: 10.1111/1365-2745.12088 (PMC3664411; doi:10.1111/1365-2745.12088)
Supplement: Supplementary file 1 [file jec0101-0585-SD1.pdf]

Age, stage, and senescence in plants

Online Only

Appendix S1: Age- and stage-specific selection gradients for selected plant species

Hal Caswell*
Biology Department MS-34
Woods Hole Oceanographic Institution
Woods Hole MA 02543 USA
and
Max Planck Institute for Demographic Research

Roberto Salguero-Gómez
Max Planck Institute for Demographic Research
Rostock, Germany

February 18, 2013

*Correspondence author. Email: hcaswell@whoi.edu

Contents

S1 Selection gradients for selected plant species **3**

S1.1 *Arisaema serratum* . . . 7
S1.2 *Aspasia principissa* . . . 10
S1.3 *Borderea chouardii* . . . 13
S1.4 *Calathea ovandensis* . . . 16
S1.5 *Calathea micans* . . . 19
S1.6 *Calochortus lyallii* . . . 22
S1.7 *Chamaecrista keyensis* . . . 25
S1.8 *Chamaelirium luteum* . . . 28
S1.9 *Cryptantha flava* . . . 31
S1.10 *Cytisus scoparius* . . . 34
S1.11 *Dicerandra frutescens* . . . 37
S1.12 *Dicymbe altsonii* . . . 40
S1.13 *Geonoma macrostachys* . . . 43
S1.14 *Hylocomium splendens* . . . 46
S1.15 *Hypericum cumulicola* . . . 49
S1.16 *Iridaea splendens* . . . 52
S1.17 *Laminaria digitata* . . . 55
S1.18 *Lupinus arboreus* . . . 58
S1.19 *Machaerium cuspidatum* . . . 61
S1.20 *Opuntia rastrera* . . . 64
S1.21 *Panax quinquefolium* . . . 67
S1.22 *Parashorea chinensis* . . . 70
S1.23 *Periandra mediterranea* . . . 73
S1.24 *Pinguicula alpina* . . . 76
S1.25 *Pinus lambertiana* . . . 79
S1.26 *Pinus ponderosa* . . . 82
S1.27 *Polystichum aculeatum* . . . 85
S1.28 *Primula vulgaris* . . . 88
S1.29 *Pterocarpus angolensis* . . . 91
S1.30 *Pterocarya rhoifolia* . . . 94
S1.31 *Rhododendron ponticum* (Endangered) . . . 97
S1.32 *Rhododendron ponticum* (Invasive) . . . 100
S1.33 *Rhopalostylis sapida* . . . 103
S1.34 *Rourea induta* . . . 106
S1.35 *Tillandsia recurvata* . . . 109
S1.36 *Viburnum furcatum* . . . 112

Appendix S1 Selection gradients for selected plant species

In this appendix, we present the selection gradients on mortality and fertility for a selection of 36 plant (and red algae) species of a variety of growth forms, habitats and taxa. Table 1 lists their citations, as well as taxonomic, ecological and matrix model characteristics of these species. Data were obtained from the ComPADRe III database of plant and animal population projection models, under development at the Max Planck Institute for Demographic Research, Rostock, Germany.

For each species, we show three views of the gradients. The first shows the sensitivities of $r = \log\lambda$ to age-specific mortality, age-specific fertility, stage-specific mortality, and stage-specific fertility. The second shows a surface view of the selection gradients on age$\times$stage-specific mortality and fertility. The third figure shows the age$\times$stage-specific gradients as functions of age and of age normalized by the life expectancy of the first non-seedbank stage in the life-cycle of the species.

	Species (Family)	Citation	Growth form	Habitat	Status	s	α	FNSS
1	*Arisaema serratum* (Araceae)	Kinoshita 1987	Perennial herb	Temperate	Common	19	6	1
2	*Aspasia principissa* (Orchidaceae)	Zotz & Schmidt 2006	Epiphyte	Tropical	Endangered	7	6	1
3	*Borderea chouardii* (Dioscoraceae)	García & Antor 1995	Perennial herb	Alpine	Common	8	3	2
4	*Calathea ovandensis* (Marantaceae)	Horvitz & Schemske 2005	Perennial herb	Tropical	Common	8	6	2
5	*Calathea micans* (Marantaceae)	Le Corff & Horvitz 2005	Perennial herb	Tropical	Common	10	4	1
6	*Calochortus lyallii* (Liliaceae)	Miller, Antos & Allen 2007	Perennial herb	Grassland	Common	9	2	1
7	*Chamaecrista keyensis* (Fabaceae)	Liu, Menges & Quintana-Ascencio 2005	Perennial herb	Temperate	Endangered	8	3	2
8	*Chamaelirium luteum* (Liliaceae)	Meagher 1982	Perennial herb	Temperate	Common	24	16	1
9	*Cryptantha flava* (Boraginaceae)	Lucas, Forseth & Casper 2008	Subshrub	Desert	Common	7	6	1
10	*Cytisus scoparius* (Fabaceae)	Neubert & Parker 2004	Shrub	Temperate	Invasive	7	5	2
11	*Dicerandra frutescens* (Lamiaceae)	Menges *et al.* 2006	Perennial herb	Temperate	Endangered	5	3	2
12	*Dicymbe altsonii* (Fabaceae)	Zagt 1997	Tree	Tropical	Common	15	3	2
13	*Geonoma macrostachys* (Arecaceae)	Svenning 2002	Palm	Tropical	Common	10	6	1
14	*Hylocomium splendens* (Hylocomiaceae)	Okland 1995	Moss	Alpine	Common	6	5	2
15	*Hypericum cumulicola* (Clusiaceae)	Quintana-Ascencio, Menges & Weekley 2003	Perennial herb	Temperate	Endangered	6	4	1
16	*Iridaea splendens* (Gigartinaceae)	Ang *et al.* 1990	Red algae	Marine	Common	8	4	1
17	*Laminaria digitata* (Laminariaceae)	Chapman 1993	Green algae	Shore	Common	5	4	2
18	*Lupinus arboreus* (Fabaceae)	Kauffman & Maron 2006	Shrub	Dune	Common	9	8	2
19	*Machaerium cuspidatum* (Fabaceae)	Nabe-Nielsen 2004	Liana	Tropical	Common	9	7	1
20	*Opuntia rastrera* (Cactaceae)	Mandujano *et al.* 2001	Succulent	Desert	Common	10	9	2
21	*Panax quinquefolium* (Araliaceae)	Nantel, Gagnon & Nault 1996	Perennial herb	Temperate	Endangered	5	3	2
22	*Parashorea chinensis* (Dipterocarpaceae)	Chien, Zuidema & Nghia 2008	Tree	Tropical	Endangered	15	9	1
23	*Periandra mediterranea* (Fabaceae)	Hoffmann & Solbrig 2003	Sub-shrub	Tropical	Common	8	7	1
24	*Pinguicula alpina* (Lentibulariaceae)	Svennson *et al.* 1993	Perennial herb	Tundra	Common	11	5	1
25	*Pinus lambertiana* (Pinaceae)	van Mantgem & Stepheson 2005	Tree	Temperate	Common	5	1	1
26	*Pinus ponderosa* (Pinaceae)	van Mantgem & Stepheson 2005	Tree	Temperate	Common	5	1	1
27	*Polystichum aculeatum* (Dryopteridaceae)	de Groot 2011	Fern	Temperate	Common	7	3	2
28	*Primula vulgaris* (Primulaceae)	Valverde & Silvertown 1998	Perennial herb	Mediterranean	Common	5	3	2
29	*Pterocarpus angolensis* (Fabaceae)	Desmet, Shackleton & Robinson 1996	Tree	Montane	Common	4	3	1
30	*Pterocarya rhoifolia* (Juglandaceae)	Kaneko & Kawano 2002	Tree	Temperate	Common	7	1	1
31	*Rhododendron ponticum* (Ericaceae)	Salguero-Gómez 2004	Tree	Temperate	Endangered	4	2	1
32	*Rhododendron pontium* (Ericaceae)	Salguero-Gómez 2004	Shrub	Temperate	Invasive	4	2	1
33	*Rhopalostylis sapida* (Arecaceae)	Enright & Watson 1992	Palm	Temperate	Common	8	4	1
34	*Rourea induta* (Connaraceae)	Hoffmann 1999	Shrub	Savannah	Common	14	11	1
35	*Tillandsia recurvata* (Bromeliaceae)	T. Valverde & R. Bernal pers. comm.	Epiphyte	Tropical	Common	4	3	1
36	*Viburnum furcatum* (Adoxaceae)	Hara et al. 2004	Shrub	Temperate	Common	7	5	1

Table 1: List of species and characteristics. s: matrix dimension; α: first reproductive stage; FNSS: first non-seedbank stage.

References to studied species

Ang, P., de Wreede R. D., Shaughnessy, F. & Dyck, L. (1990) A simulation model for an *Iridaea splendens* (Gigartinales, Rhodophyta) population in Vancouver, Canada. *Hydrobiologia*, **204/205**, 191–196.

Chapman, A. R. O. (1993) 'Hard' data for matrix modelling of *Laminaria digitata* (Laminariales, Phaeophyta) populations. *Hydrobiologia*, **260/261**, 263–267.

Chien, P. D., Zuidema, P. A., & Nghia, N. H. (2008) Conservation prospects for threatened Vietnamese tree species: results from a demographic study. *Population Ecology*, **50**, 227–237.

Desmet, P. G., Shackleton, C. M. & Robinson, E. R. (1996) The population dynamcis and life-history of a *Pterocarpus angolensis* DC. population in the Northern Province, South Africa. *South AFrica Journal of Botany*, **62**, 160–166.

Enright, N. J. & Watson, A. D. (1992) Population dynamics of the nikau palm, *Rhopalostylis sapida* (Wendl. et Drude), in a temperate forest remnant near Auckland, New Zealand. *New Zealand Journal of Botany*, **20**, 29–43.

García, M. B. & Antor R. J. (1995) Age and size structure in populations of a long-lived dioecious geophyte: *Borderea pyrenaica* (Dioscoreaceae). *International Journal of Plant Science*, **156**, 236–243.

de Groot, G. A. (2001) *The Fate of a Colonizer: Succesful but Lonely?* PhD thesis. Utrecht University, the Netherlands. p. 197.

Hara, M., Kanno, H., Hirabuki, Y. & Takehara, A. (2004) Population dynamics of four understory shrub species in beech forest. *Journal of Vegetation Science*, **15**, 475–484.

Hoffmann, W. A. (1999) Fire and population dynamics of woddy plants in a neotropical savanna: matrix model populations. *Ecology*, **80**, 1354–1369.

Hoffmann, W. A. & Solbrig, O. T. (2003) The role of topkill in the differential response of savanna woody species to fire. *Forest Ecology and Management*, **180**, 273–286.

Horvitz, C. C. & Schesmke, D. W. (1995) Spatiotemporal variation in demographic transitions of a tropical understory herb: projection matrix analysis. *Ecological Monographs*, **65**, 155–192.

Kaneko, Y. & Kawano, S. (2002) Demography and matrix analysis on a natural *Pterocarya rhoifolia* population developed along a mountain stream. *Journal of Plant Research*, **115**, 341–354.

Kauffman, M. J. & Maron, J. L. (2006) Consumers limit the abundance and dynamics of a perennial shrub with a seed bank. *The American Naturalist*, **168**, 444–470.

Kinoshita, A. (1987) Sex change and population dynamics in *Arisaema* I. *Arisaema serratum* (Thunb.) Schott. *Plant Species Biology*, **2**, 15–28.

LeCorff, J. & Horvitz, C. C. (2005) The contrasting roles of chasmogamous and cleistogamous reproduction in population growth and population spread in an ant- dispersed neotropical herb, Calathea micans (Marantaceae). *Ecological Modelling*, **188**, 41–51.

Liu, H., Menges, E. S., & Quintana-Ascencio, P. F. (2005) Population viability analyses of *Chamaecrista keyensis*: effects of fire season and frequency. *Ecological Applications*, **15**, 210–221.

Lucas, R. W., Forseth, I. N. & Casper, B. B. (2008) Using rainout shelters to evaluate climate change effects on the demography of *Cryptantha flava*. *Journal of Ecology*, **96**, 514–522.

Mandujano, M. C., Montana, C., Franco, M., Golubov, J. & Flores-Martínez, A (2001) Integration of demographic annual variability in a clonal desert cactus. *Ecology*, **82**, 344–359.

van Mantgem, P. J. & Stepheson, N. L. (2005) The accuracy of matrix population model projections for coniferous trees in the Sierra Nevada, California. *Journal of Ecology*, **93**, 737–747.

Meagher, T. R. (1982) The population biology of Chamaelirium luteum, a dioecious member of the lily family: two-sex population projections and stable population structure. *Ecology*, **63**, 1701–1711.

Menges, E. S., Quintana-Ascencio, P. F., Weekley, C. W., & Gaoue, O. G. (2006) Population viability analysis and fire return intevals of an endemic Florida scrub mint. *Biological Conservation*, **127**, 115–127.

Miller, M. T & Antos, J. A., & Allen, G. A. (2007) Demographic differences between two sympatric liilies (*Calochortus*) wiht contrasting distributions, as revealed by matrix analysis. *Plant Ecology*, **191**, 265–278.

Nabe-Nielsen, J. (2004) Demography of *Machaerium cuspidatum*, a shade-tolerant neotropical liana. *Journal of Tropical Ecology*, **20**, 505–516.

Nantel, P., Gagnon, D. & Nault, A. (1996) Population viability analysis of American ginseng and wild leek harvested in stochastic environments. *Conservation Biology*, **10**, 608–621.

Neubert, M. G. & Parker, I. M. (2004) Projecting rates of spread for invasive species. *Risk Analysis*, **24**, 817–831.

Okland, R. H. (1995) Population biology of the lconal moss *Hylocomium splendens* in Norwegian boreal spruce forests. I. Demography. *Journal of Ecology*, **83**, 697–712.

Quintana-Ascencio, P. F., Menges, E. S. & Weekley, C. W. (2003) A fire-explicit population viability analysis of *Hypericum cumulicola* in Florida rosemary scrub. *Conservation Biology*, **17**, 433–449.

Salguero-Gómez, R. (2004) *Rhododendron ponticum L: Ecological Terminator in the UK... Ecologically Terminated in Spain?* MSc thesis. Kingston University, UK. p. 200

Svenning, J.-C. (2002) Crown illumation limits the population growth rate of a neotropical understory palm (*Geonoma macrostachys*, Aracaceae). *Plant Ecology*, **159**, 185–199.

Svennson, B. M., Carlsoon, B. A., Karlsson, P. S. & Nordell, O. (1993) Comparative long-term demography of three species of *Pinguicula*. *Journal of Ecology*, **81**, 635–645.

Valverde, T & Silvertown, J. (1998) Variation in the demography of a woodland understorey herb (*Primula vulgaris*) along the forest regeneration cycle: projection matrix analysis. *Journal of Ecology*, **86**, 545–562.

Zagt, R. J. (1997) *Tree Demography in the Tropical Rain Forest of Guyana.* PhD thesis. Leiden University, the Netherlands.

Zotz, G. & Schmidt, G. (2006) Population decline in the epiphytic orchid *Aspasia principissa. Biological Conservation*, **129**, 82–90.

S1.1 *Arisaema serratum*

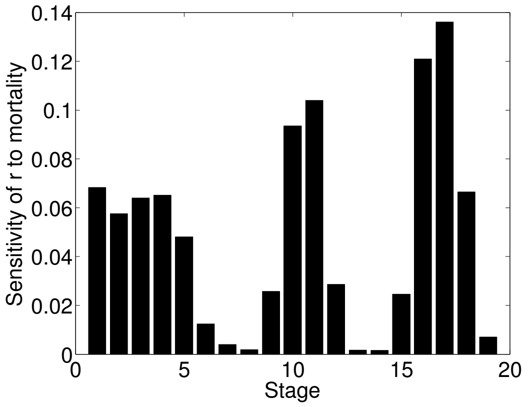

(a) Stage-specific mortality

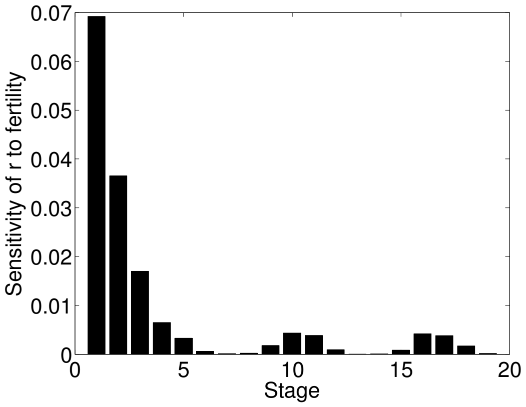

(b) Stage-specific fertility

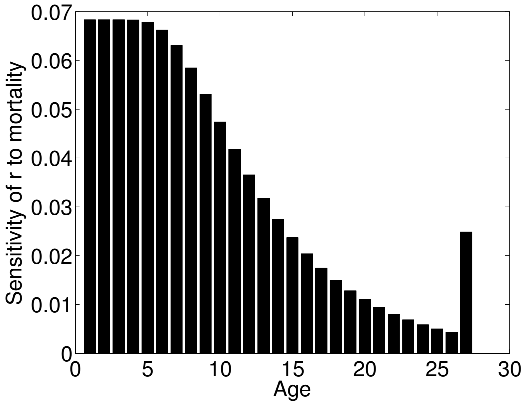

(c) Age-specific mortality

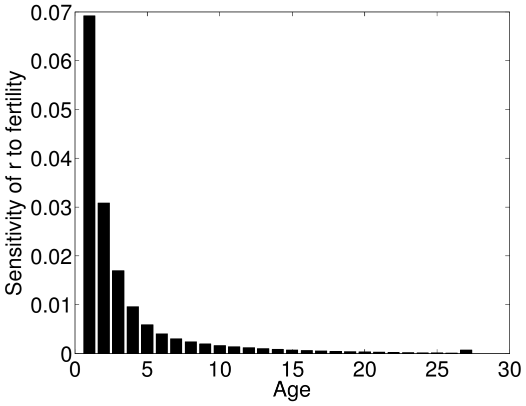

(d) Age-specific fertility

Figure 1: Selection gradients on age-specific and stage-specific mortality and fertility.

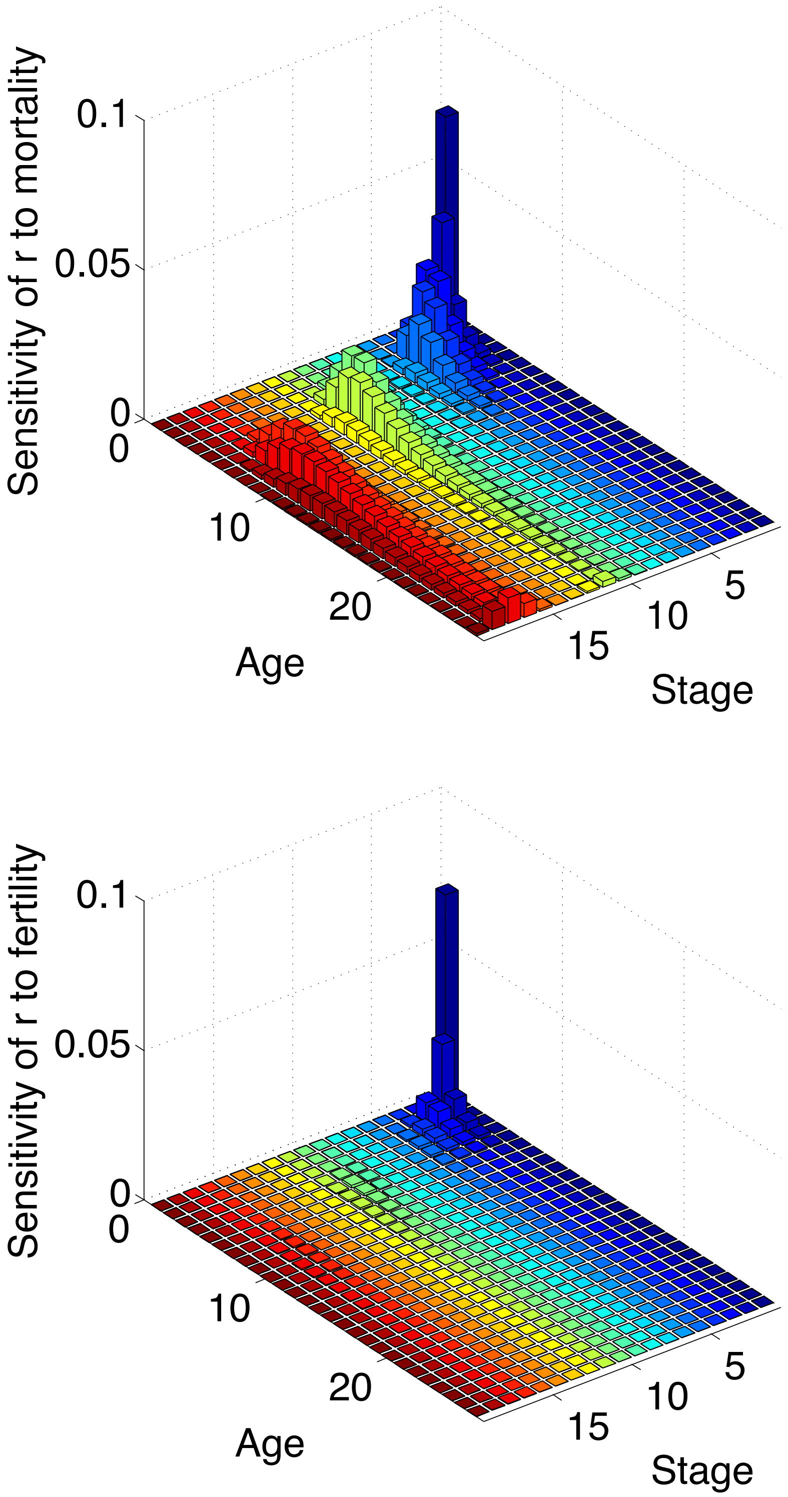

Figure 2: Selection gradients on age×stage-specific mortality and fertility.

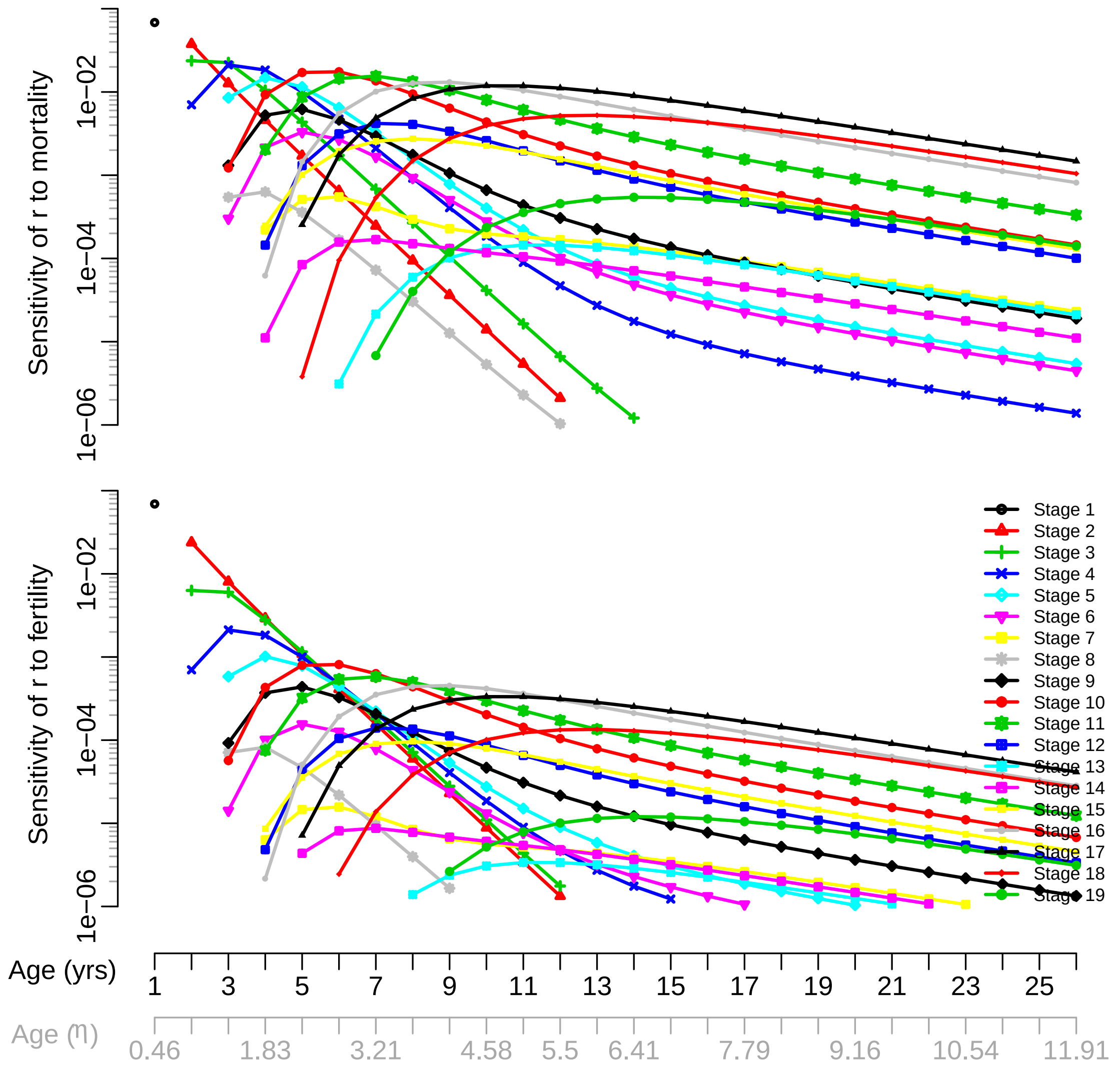

Figure 3: Selection gradients on age×stage-specific mortality and fertility. Top x axis represents age in years, whereas the bottom x axis represents time-scale normalized by the life expectancy (η) of the first non-seedbank stage (FNSS) of the species.

S1.2 *Aspasia principissa*

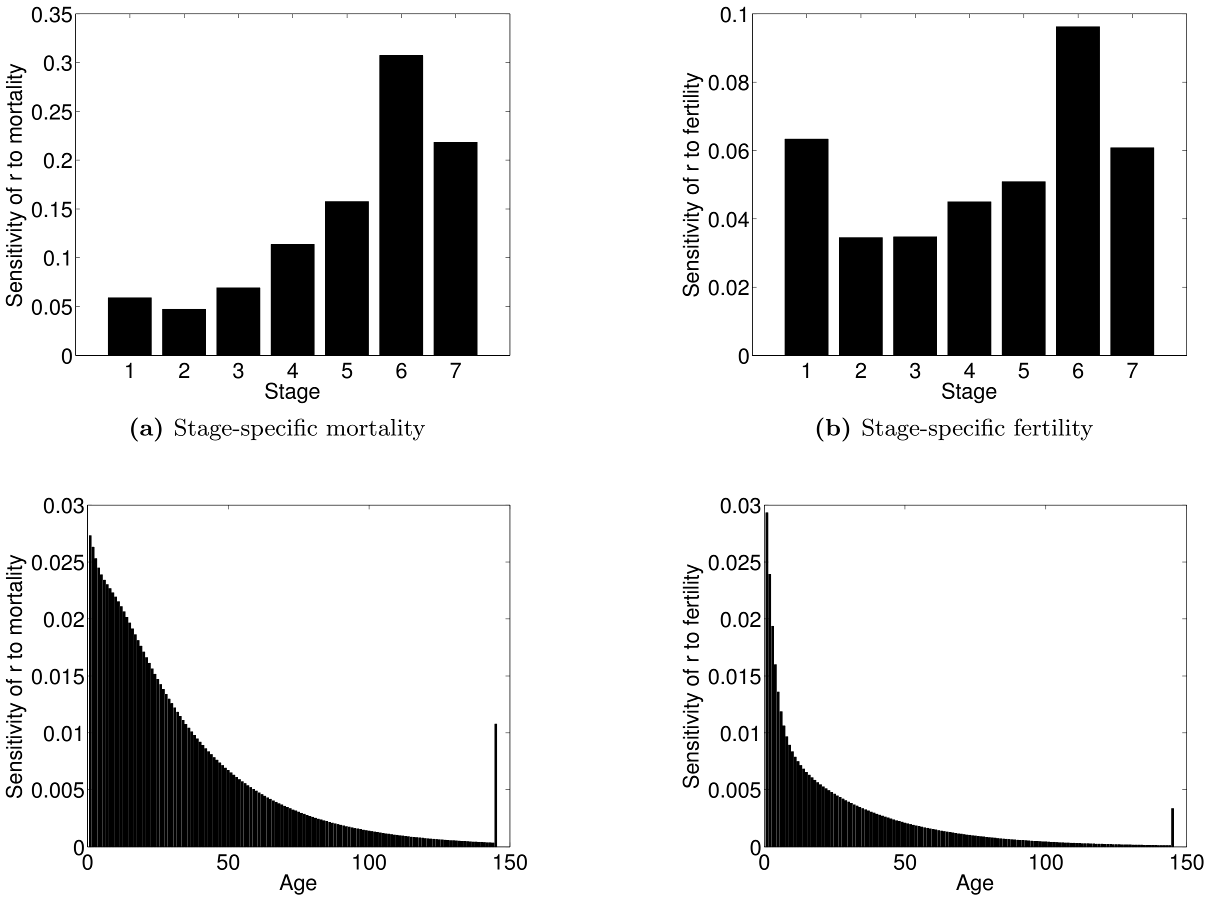

(a) Stage-specific mortality

(b) Stage-specific fertility

(c) Age-specific mortality

(d) Age-specific fertility

Figure 4: Selection gradients on age-specific and stage-specific mortality and fertility.

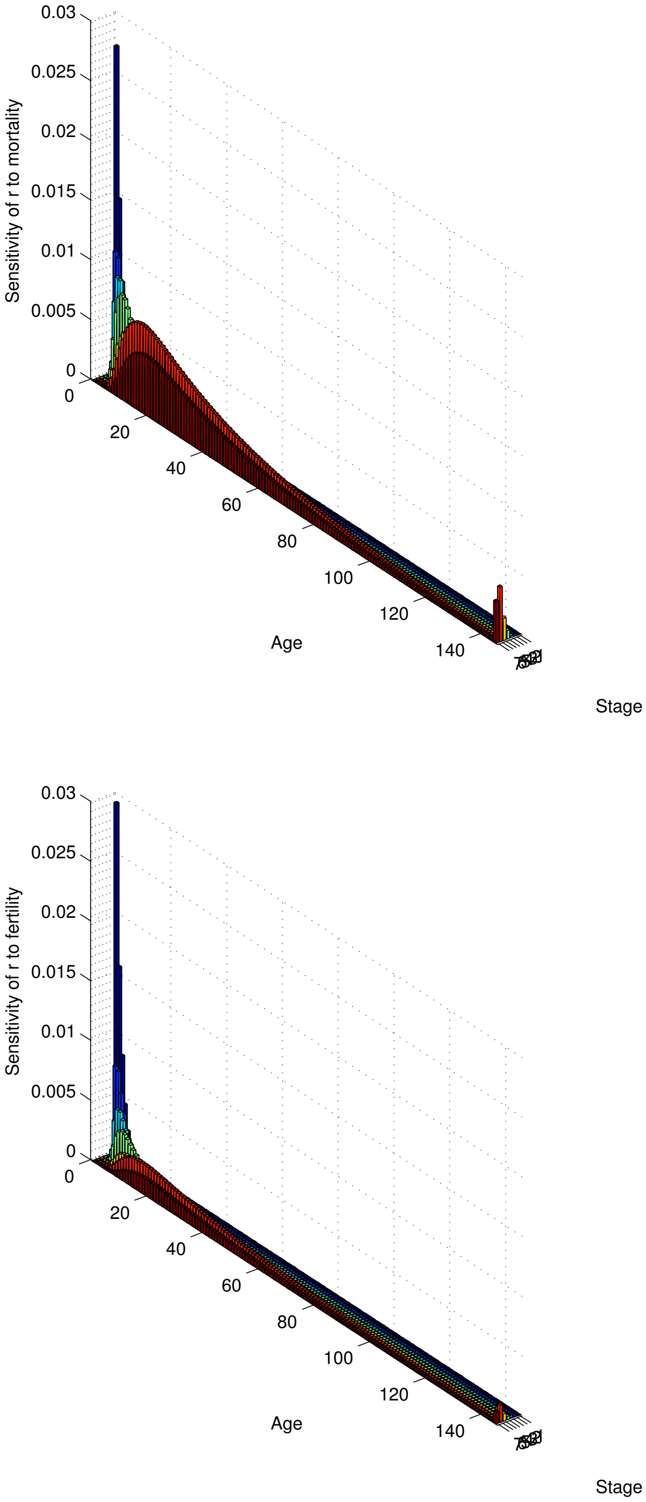

Figure 5: Selection gradients on age×stage-specific mortality and fertility.

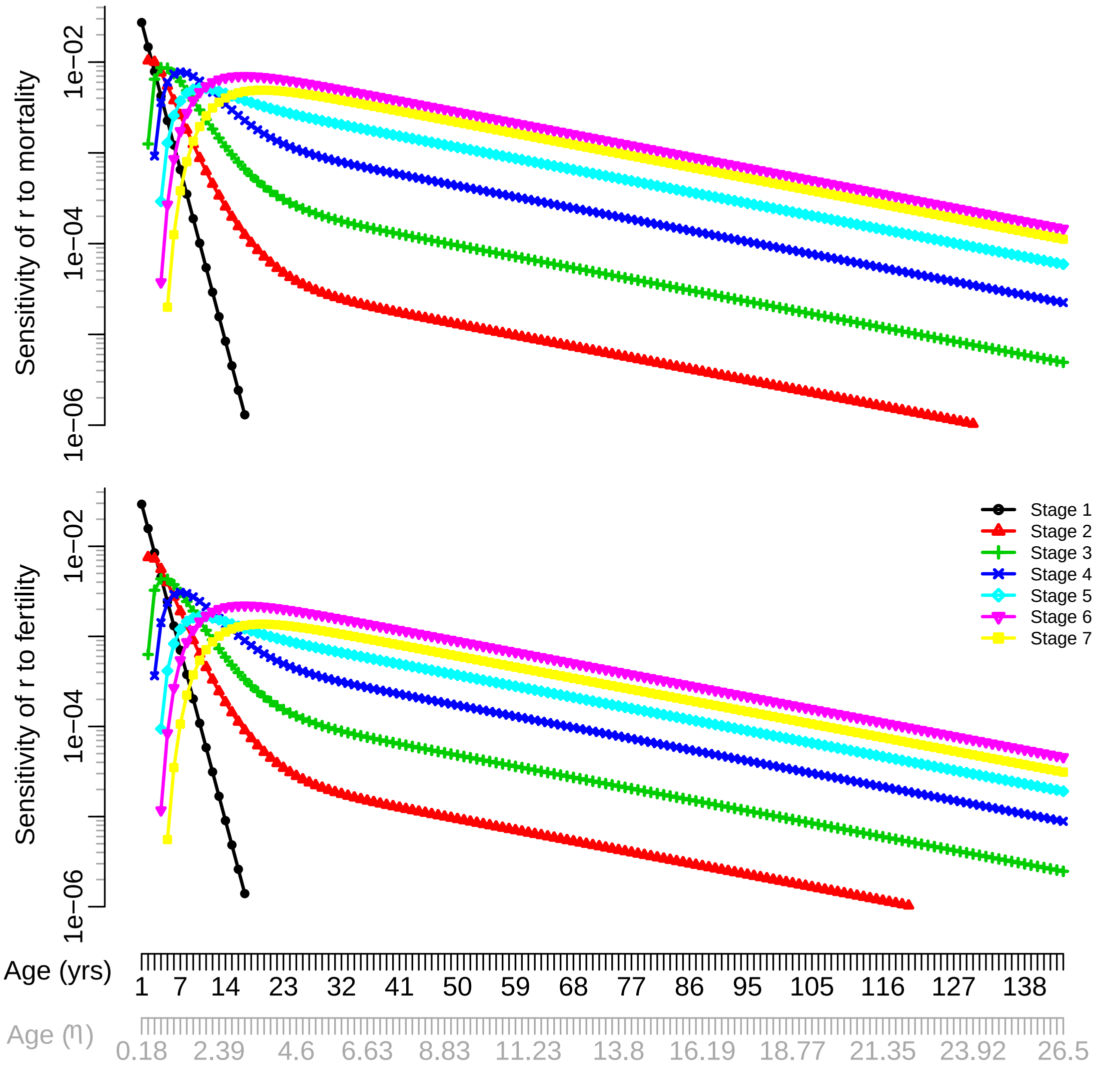

Figure 6: Selection gradients on age×stage-specific mortality and fertility. Top x axis represents age in years, whereas the bottom x axis represents time-scale normalized by the life expectancy (η) of the first non-seedbank stage (FNSS) of the species.

S1.3 *Borderea chouardii*

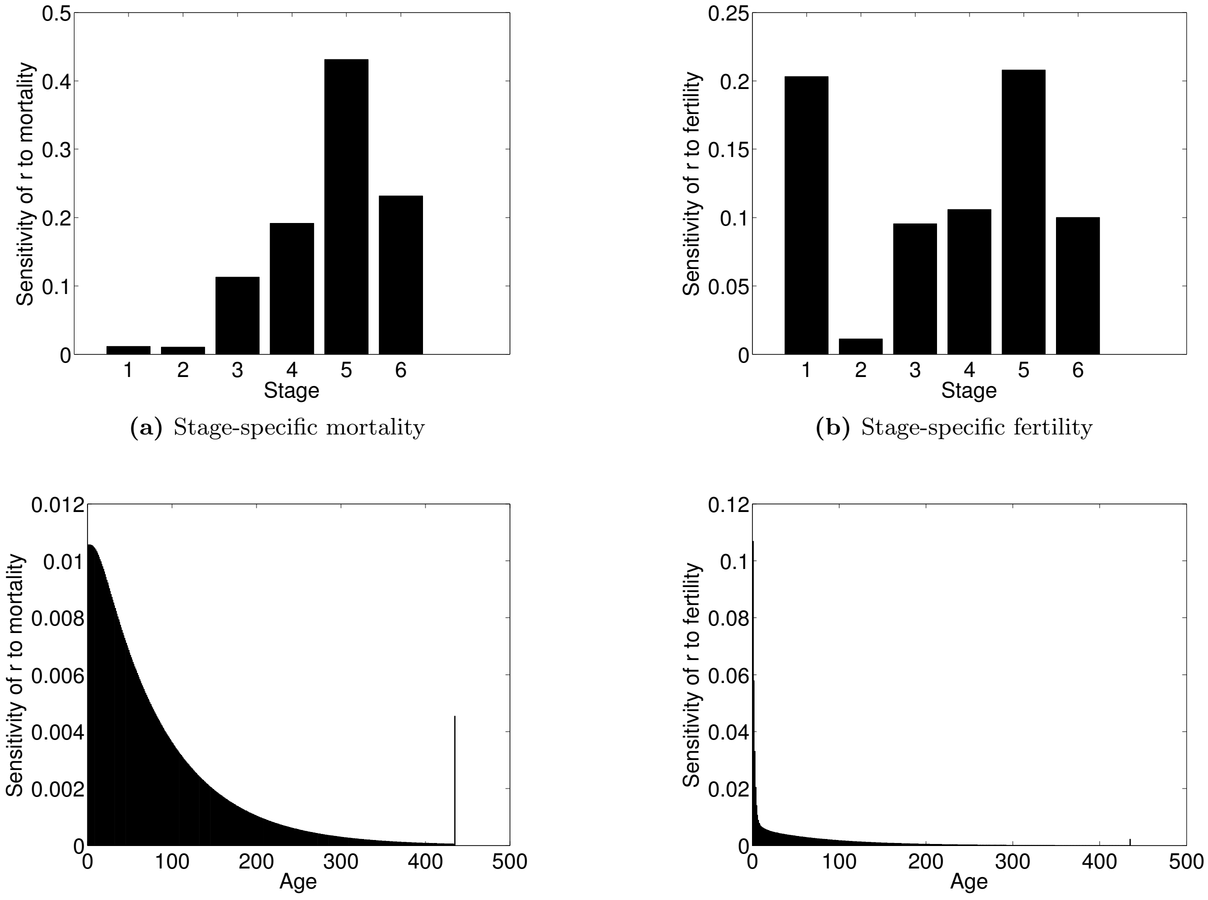

(a) Stage-specific mortality

(b) Stage-specific fertility

(c) Age-specific mortality

(d) Age-specific fertility

Figure 7: Selection gradients on age-specific and stage-specific mortality and fertility.

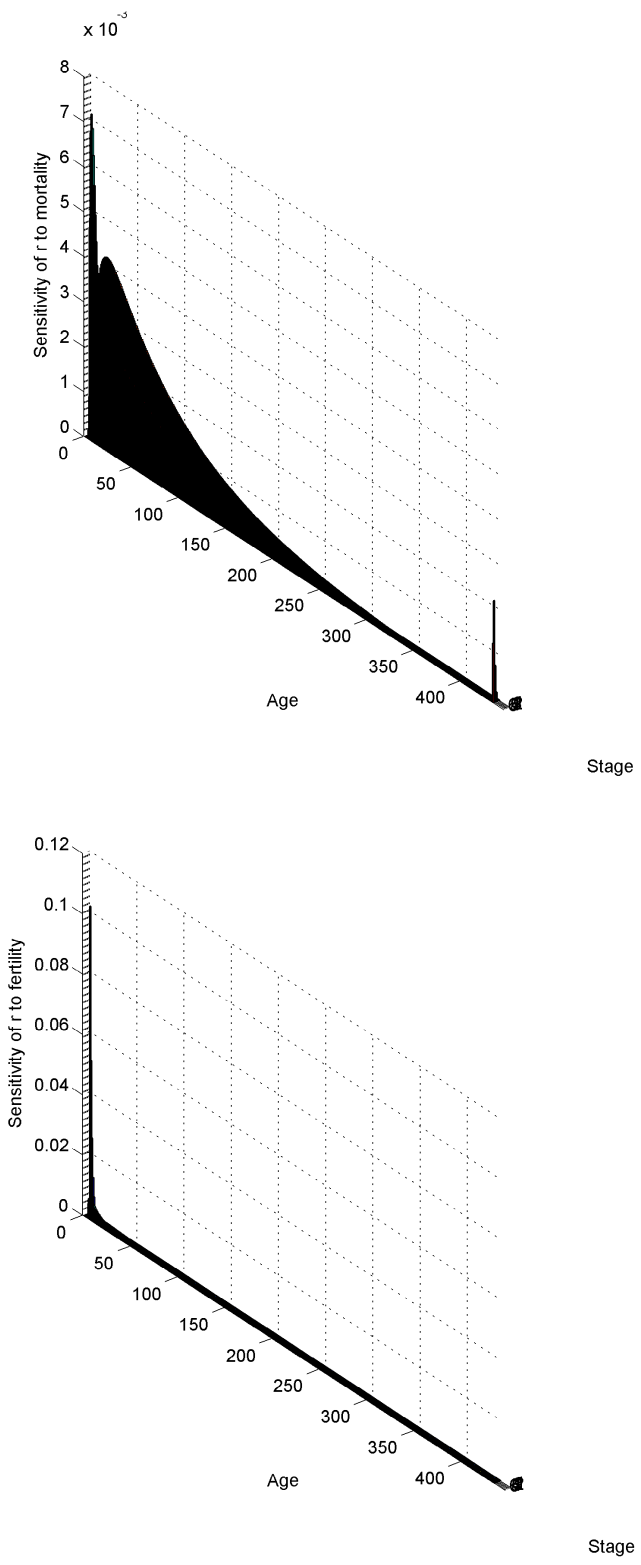

Figure 8: Selection gradients on age×stage-specific mortality and fertility.

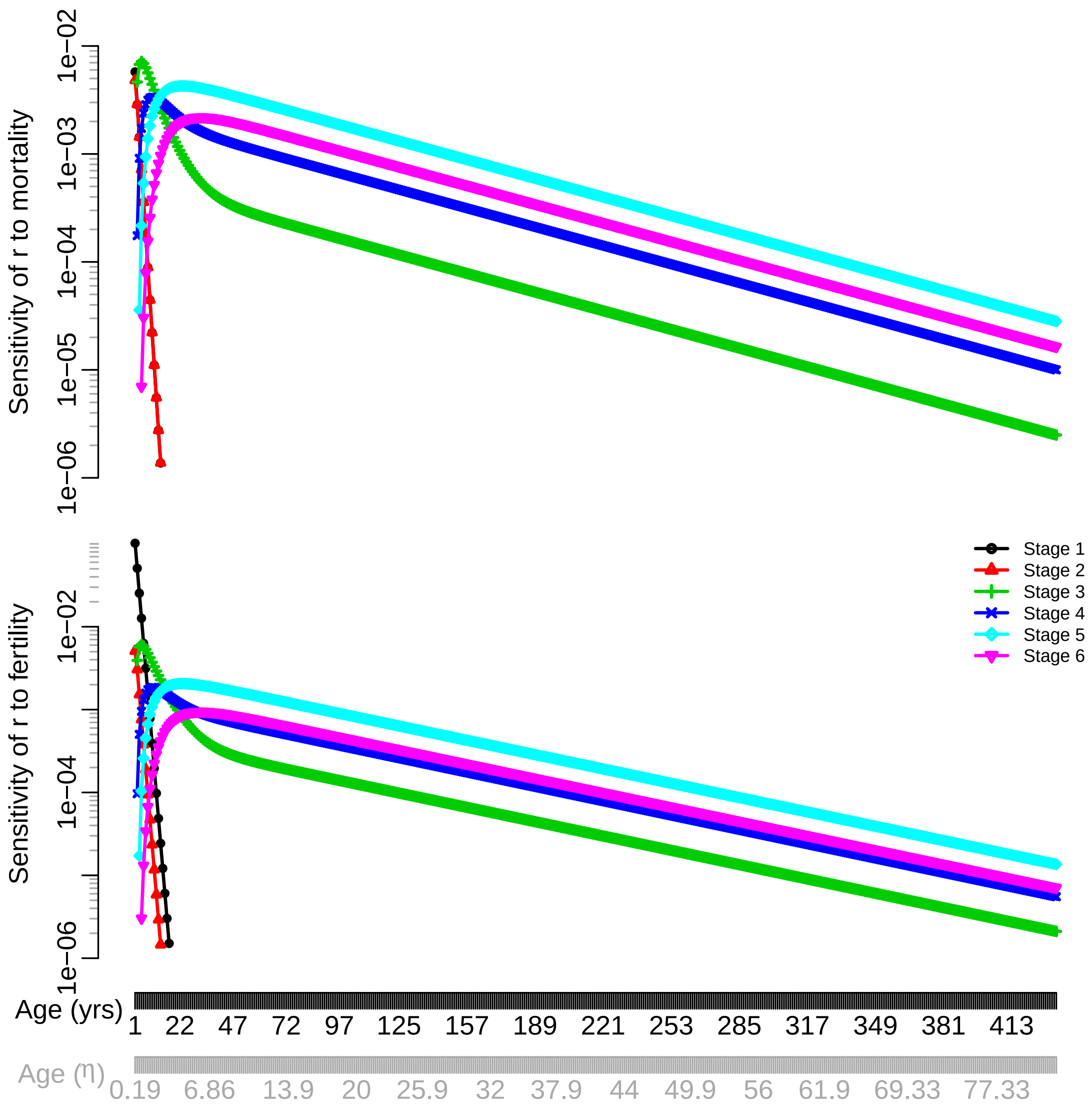

Figure 9: Selection gradients on age×stage-specific mortality and fertility. Top x axis represents age in years, whereas the bottom x axis represents time-scale normalized by the life expectancy (η) of the first non-seedbank stage (FNSS) of the species.

S1.4 *Calathea ovandensis*

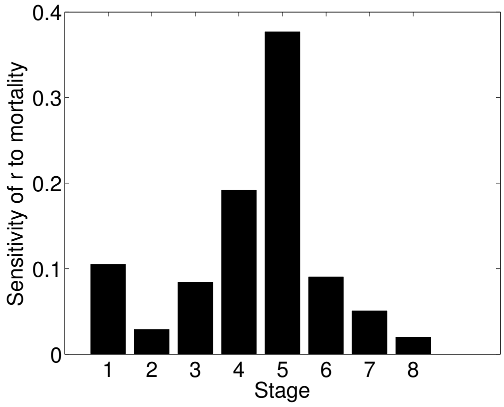

(a) Stage-specific mortality

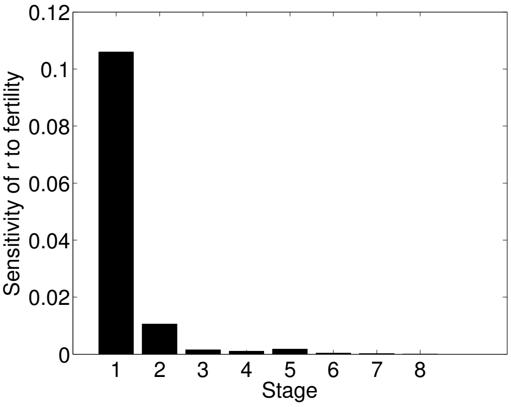

(b) Stage-specific fertility

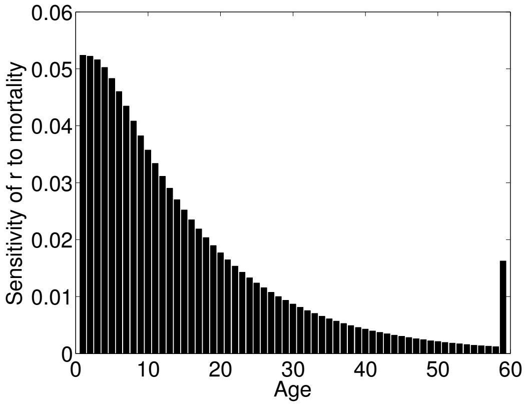

(c) Age-specific mortality

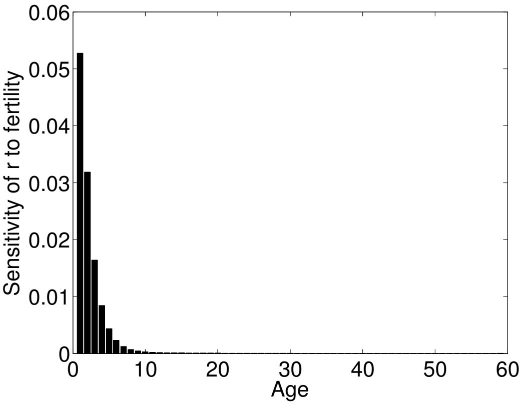

(d) Age-specific fertility

Figure 10: Selection gradients on age-specific and stage-specific mortality and fertility.

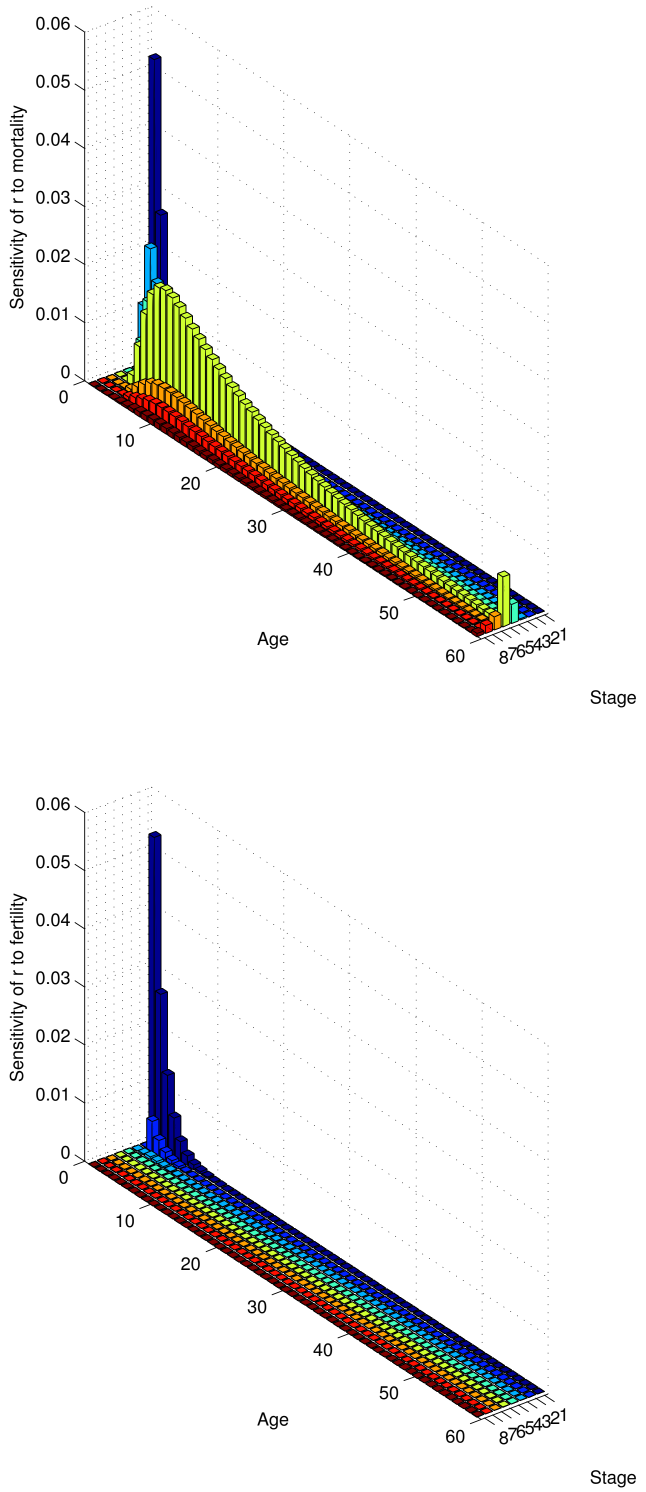

Figure 11: Selection gradients on age×stage-specific mortality and fertility.

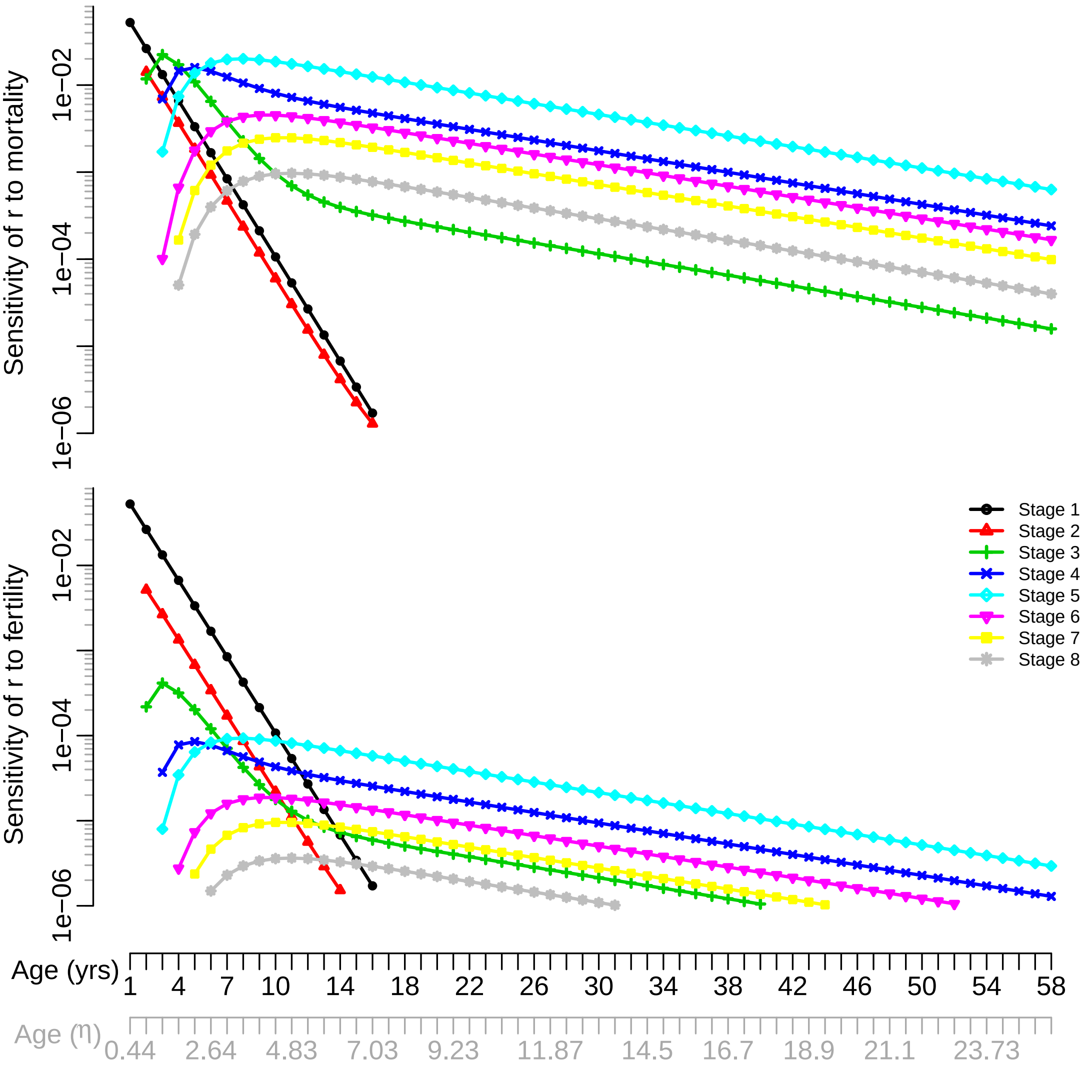

Figure 12: Selection gradients on age×stage-specific mortality and fertility. Top x axis represents age in years, whereas the bottom x axis represents time-scale normalized by the life expectancy (η) of the first non-seedbank stage (FNSS) of the species.

S1.5 *Calathea micans*

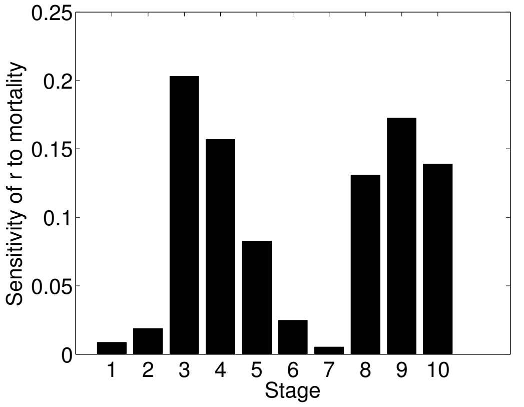

(a) Stage-specific mortality

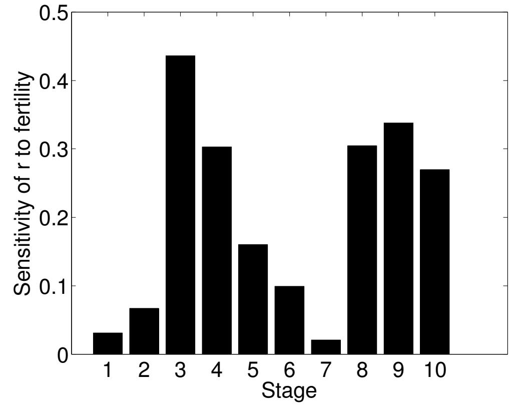

(b) Stage-specific fertility

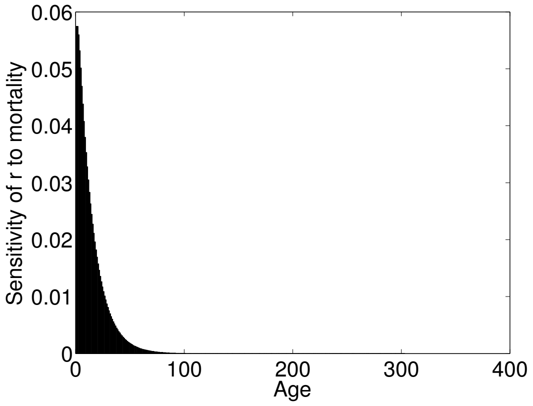

(c) Age-specific mortality

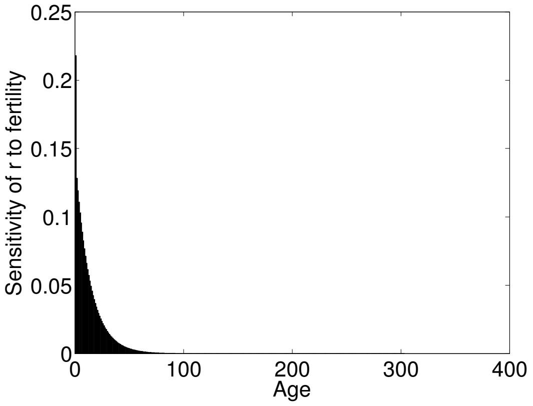

(d) Age-specific fertility

Figure 13: Selection gradients on age-specific and stage-specific mortality and fertility.

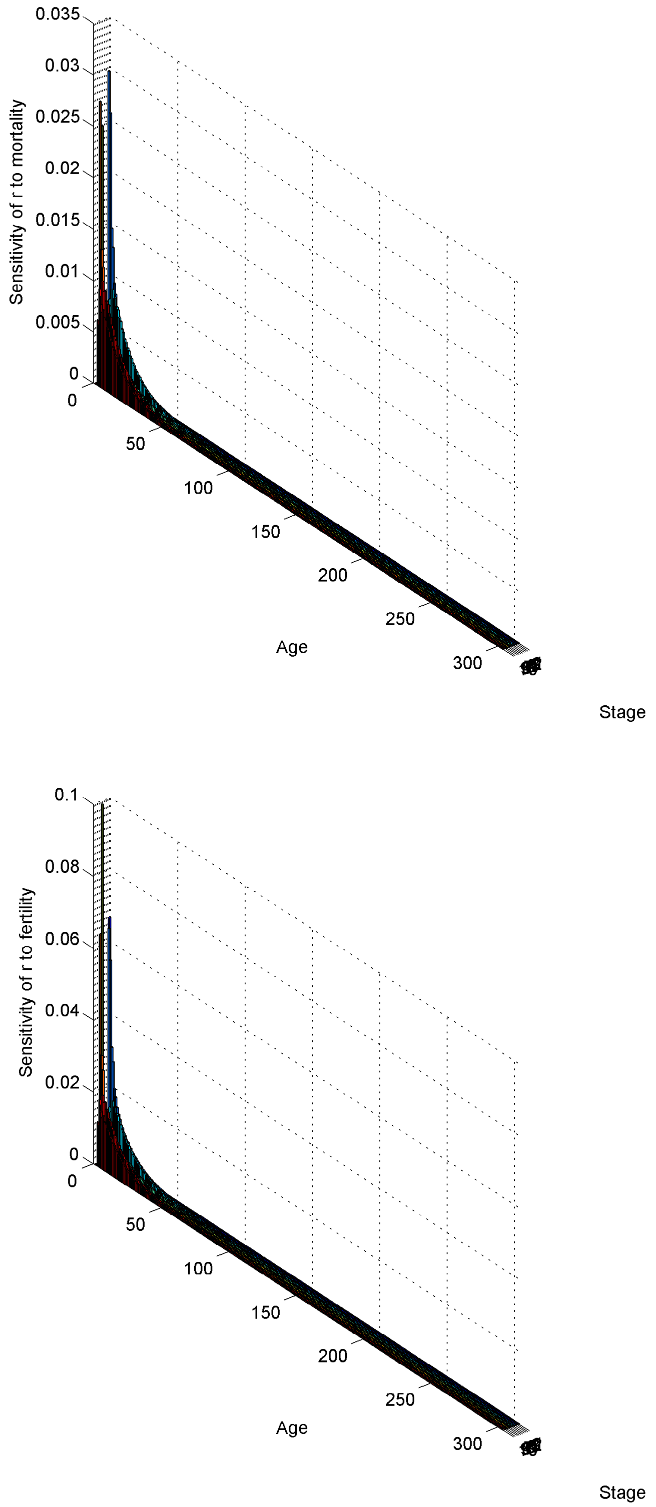

Figure 14: Selection gradients on age×stage-specific mortality and fertility.

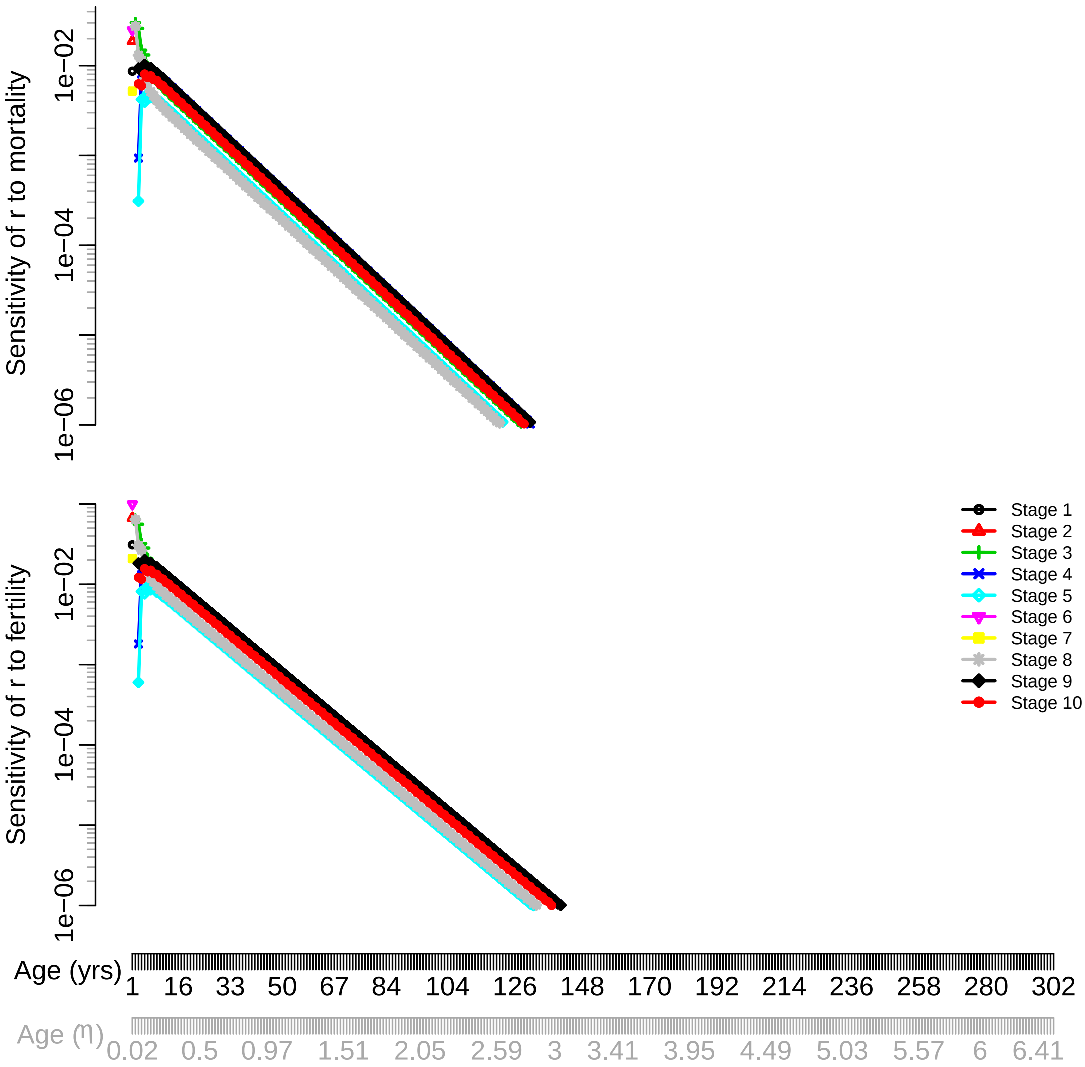

Figure 15: Selection gradients on age×stage-specific mortality and fertility. Top x axis represents age in years, whereas the bottom x axis represents time-scale normalized by the life expectancy (η) of the first non-seedbank stage (FNSS) of the species.

S1.6 *Calochortus lyallii*

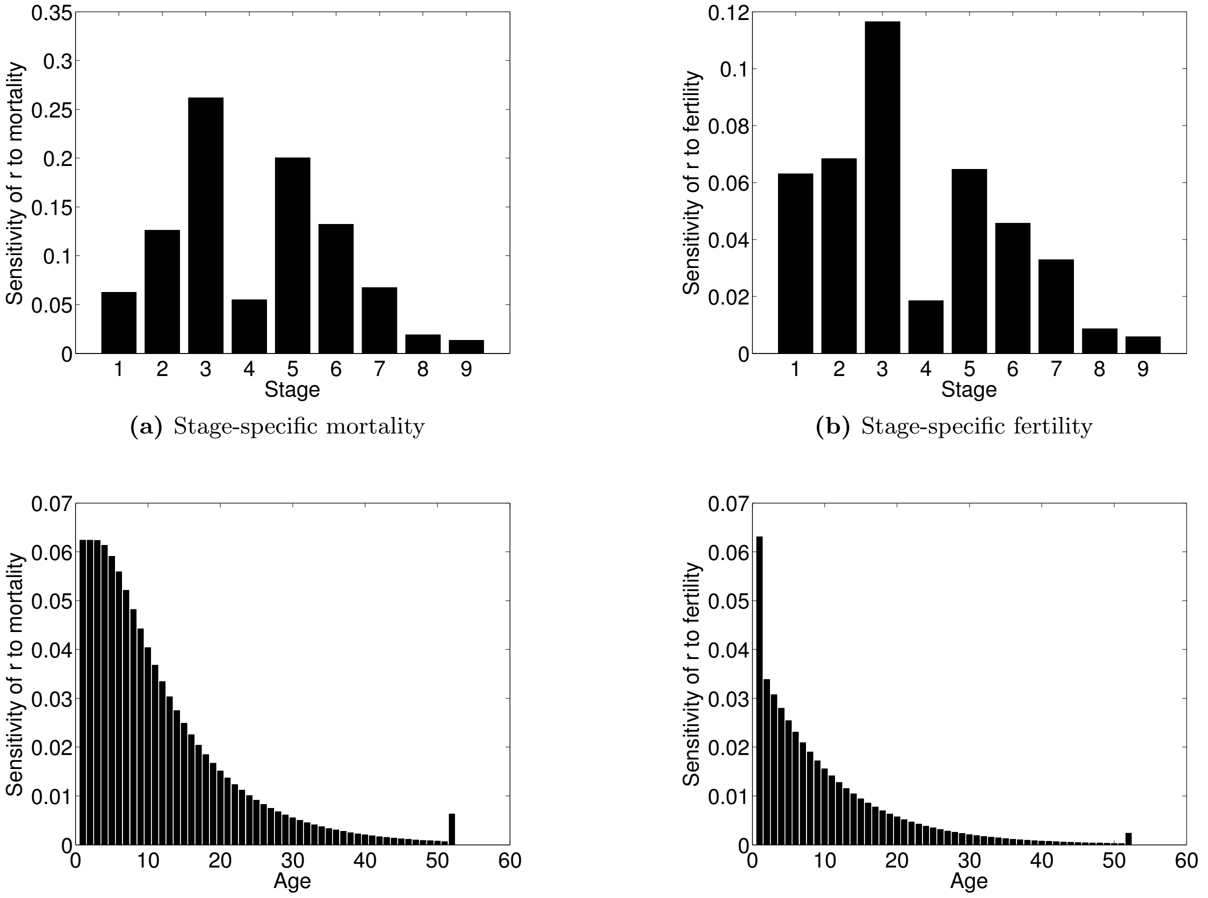

(a) Stage-specific mortality

(b) Stage-specific fertility

(c) Age-specific mortality

(d) Age-specific fertility

Figure 16: Selection gradients on age-specific and stage-specific mortality and fertility.

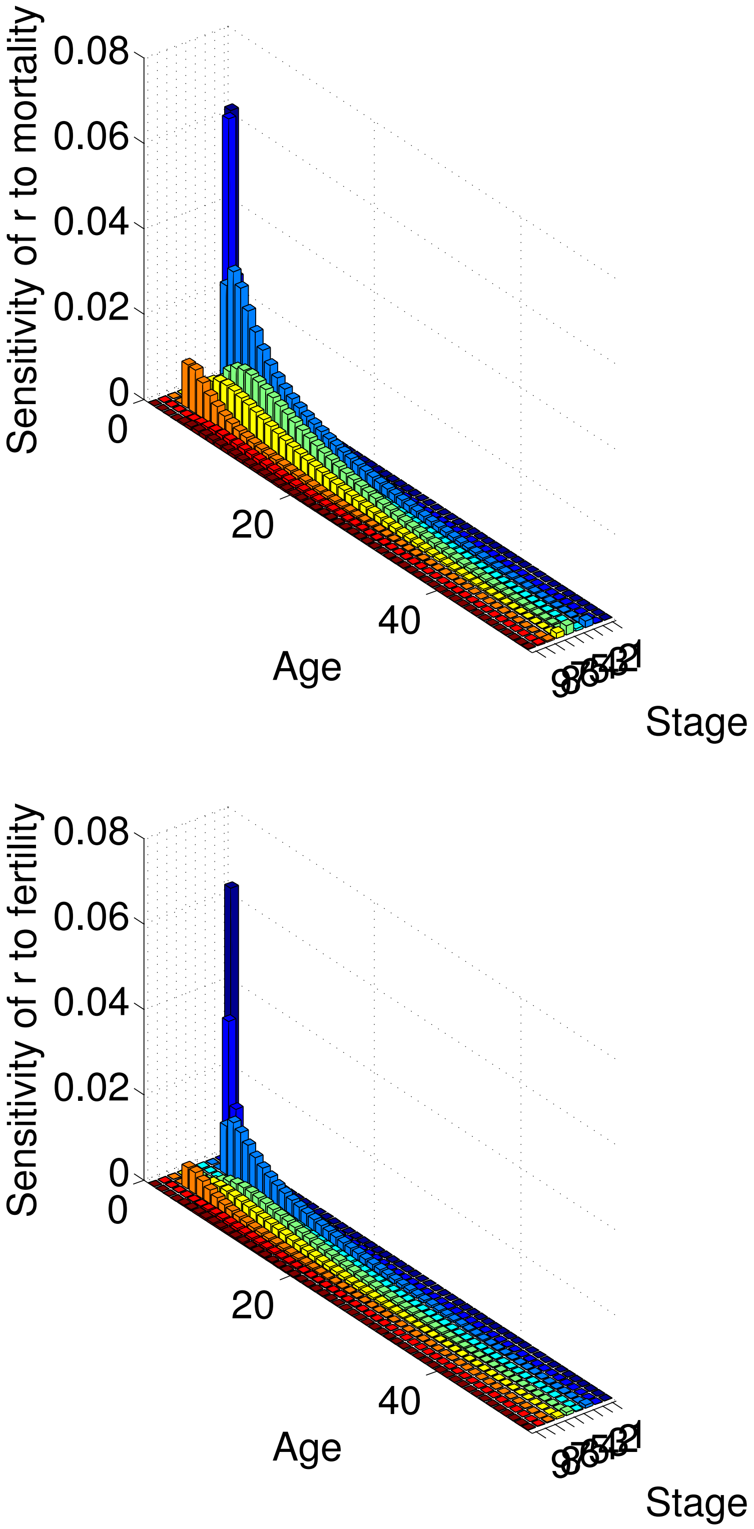

Figure 17: Selection gradients on age×stage-specific mortality and fertility.

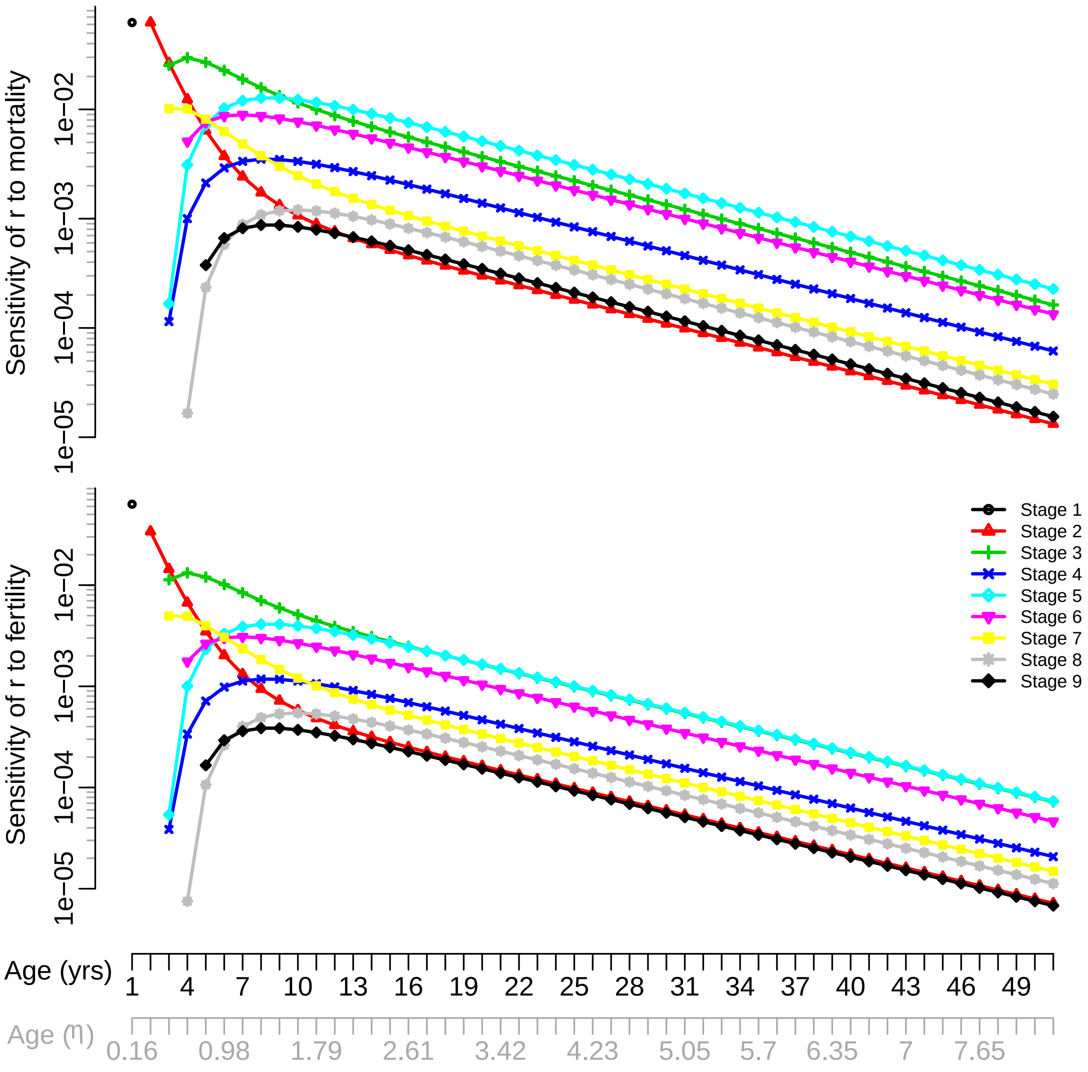

Figure 18: Selection gradients on age×stage-specific mortality and fertility. Top x axis represents age in years, whereas the bottom x axis represents time-scale normalized by the life expectancy (η) of the first non-seedbank stage (FNSS) of the species.

S1.7 *Chamaecrista keyensis*

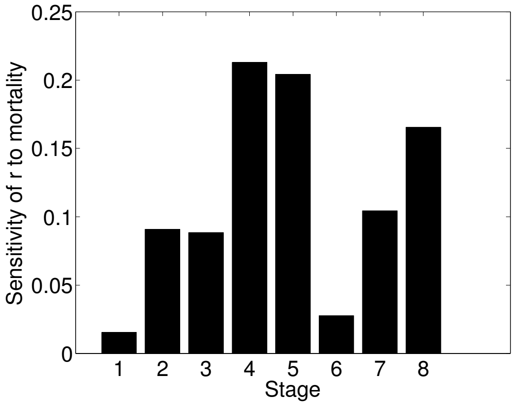

(a) Stage-specific mortality

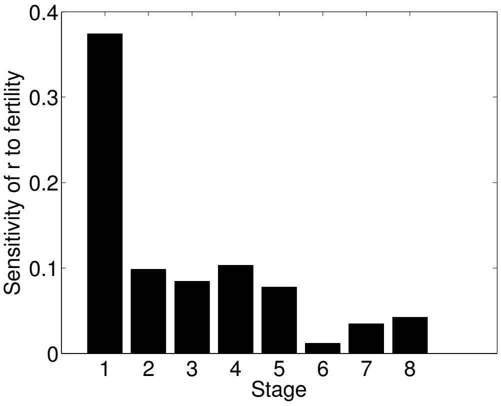

(b) Stage-specific fertility

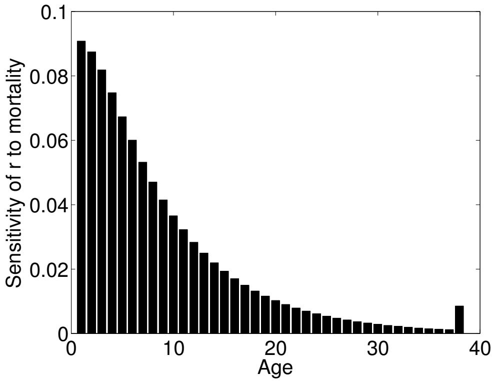

(c) Age-specific mortality

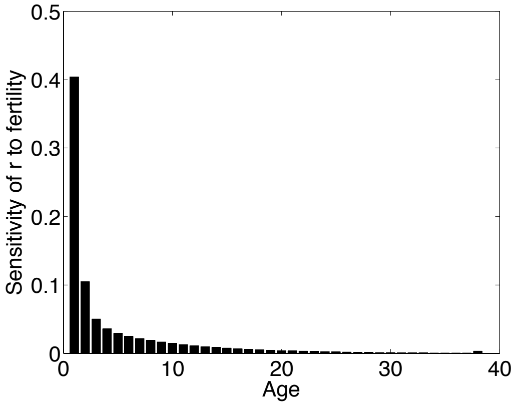

(d) Age-specific fertility

Figure 19: Selection gradients on age-specific and stage-specific mortality and fertility.

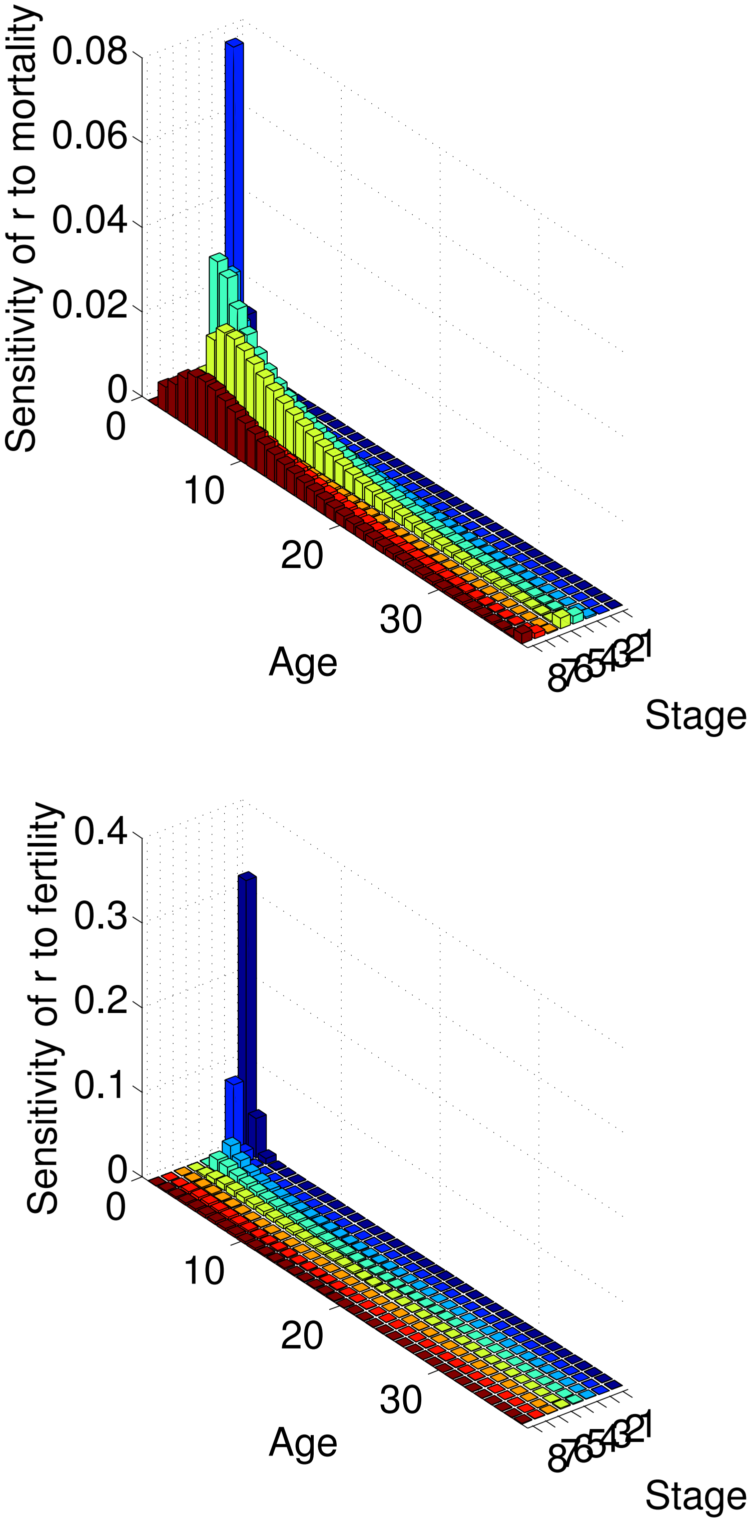

Figure 20: Selection gradients on age×stage-specific mortality and fertility.

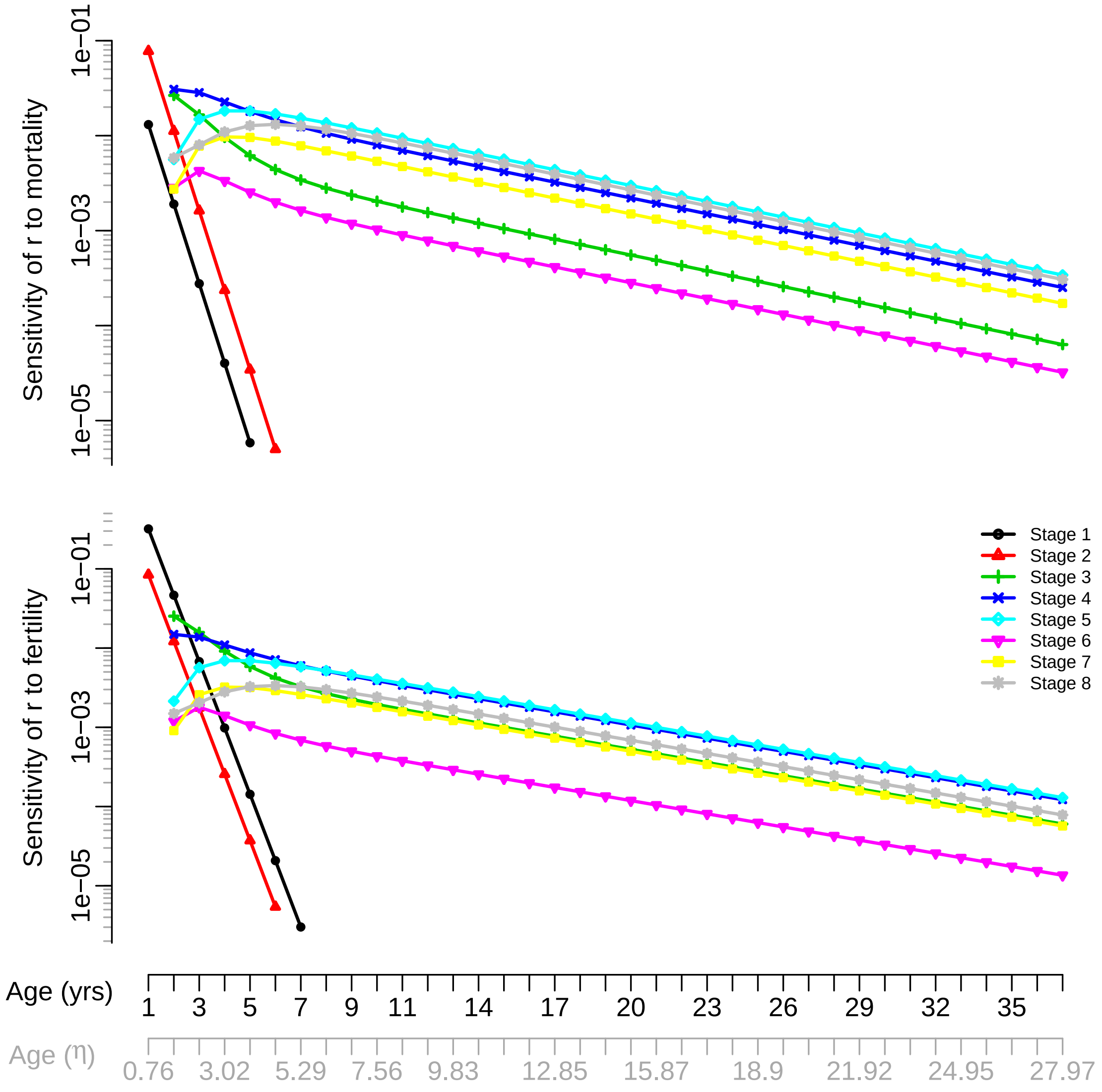

Figure 21: Selection gradients on age×stage-specific mortality and fertility. Top x axis represents age in years, whereas the bottom x axis represents time-scale normalized by the life expectancy (η) of the first non-seedbank stage (FNSS) of the species.

S1.8 *Chamaelirium luteum*

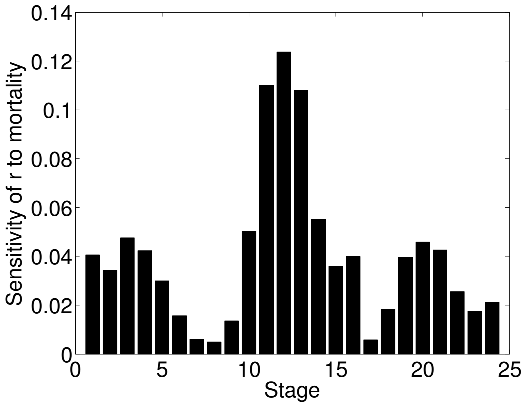

(a) Stage-specific mortality

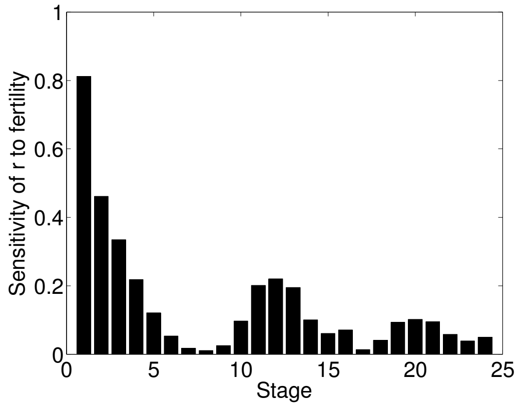

(b) Stage-specific fertility

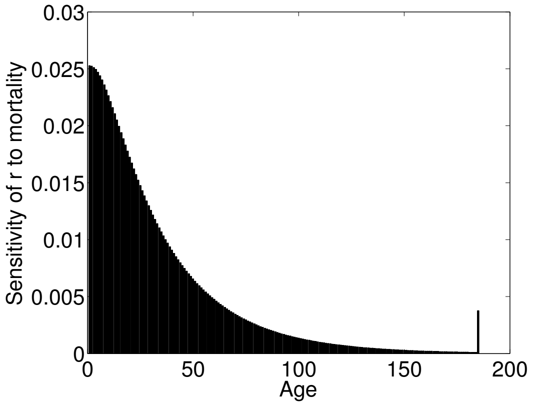

(c) Age-specific mortality

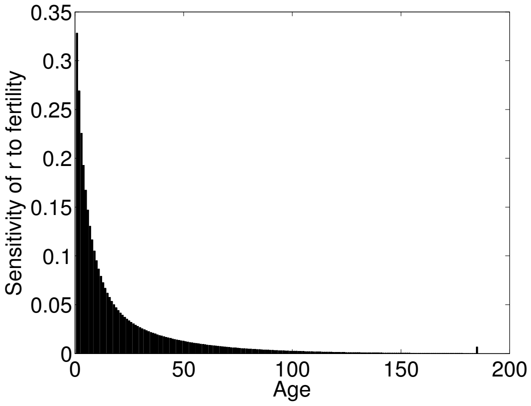

(d) Age-specific fertility

Figure 22: Selection gradients on age-specific and stage-specific mortality and fertility.

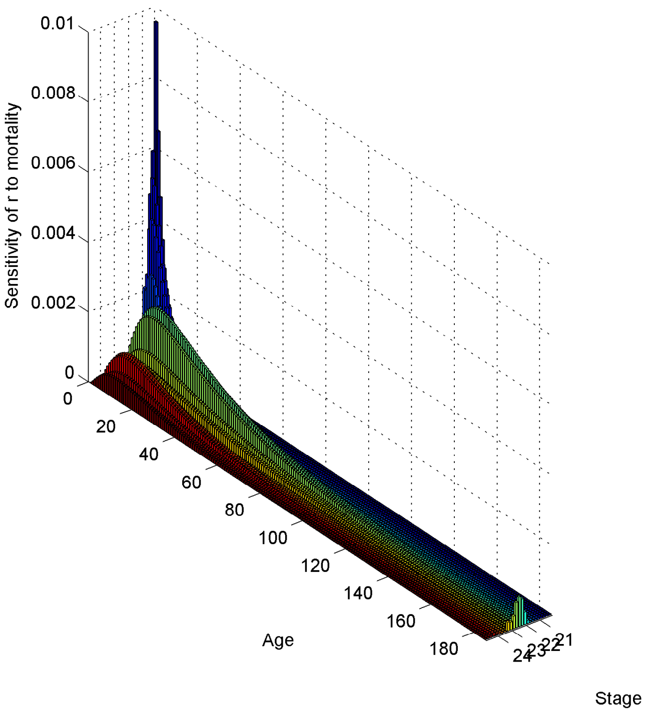

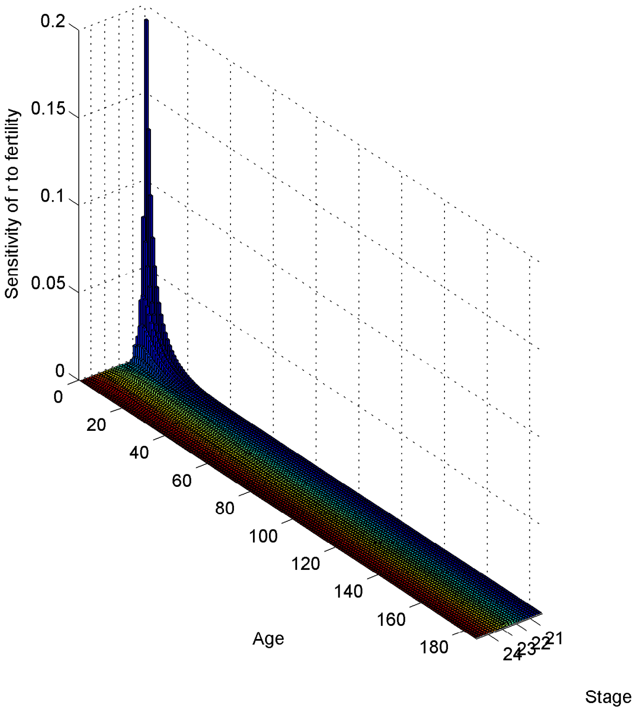

Figure 23: Selection gradients on age×stage-specific mortality and fertility.

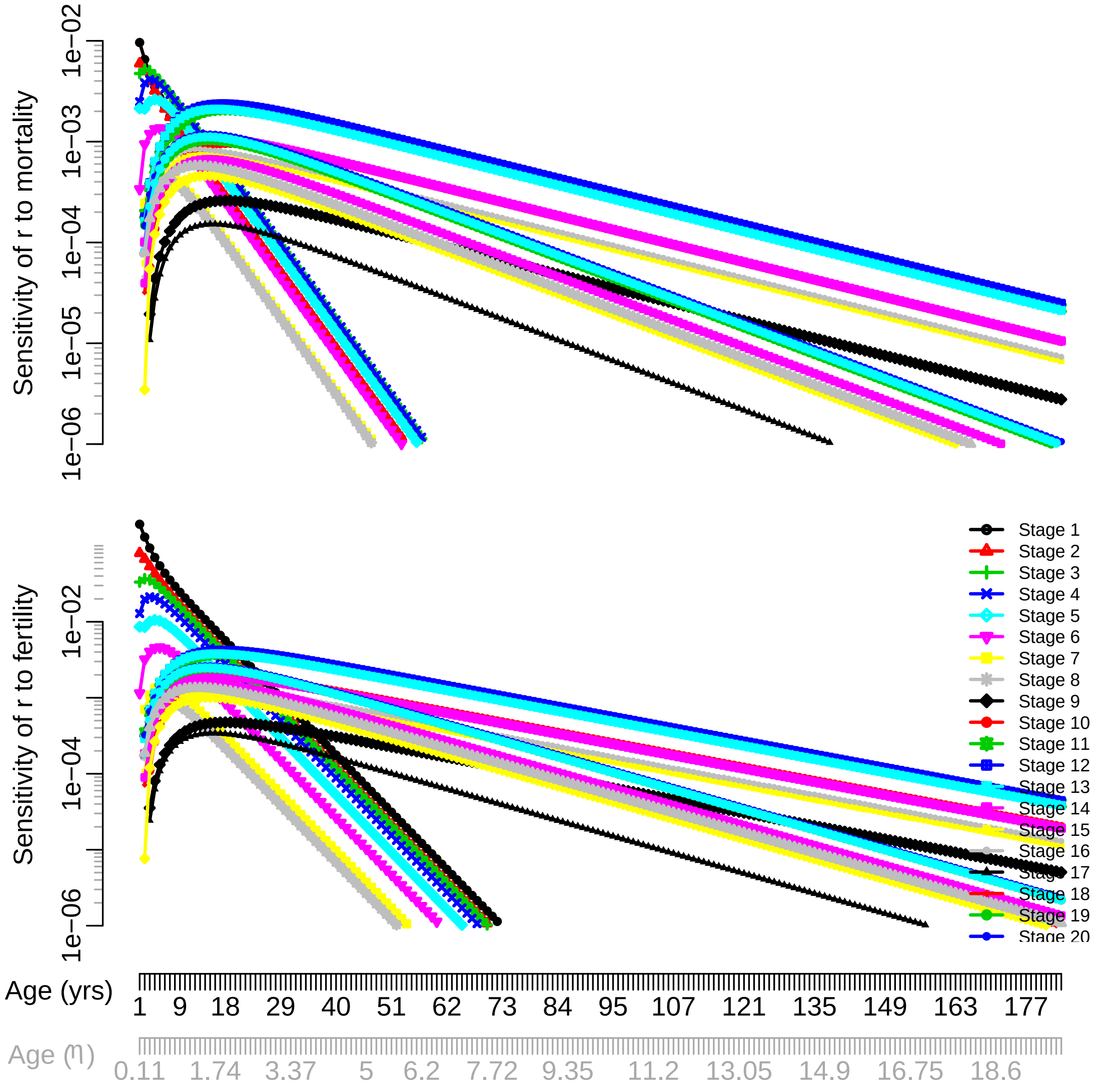

Figure 24: Selection gradients on age×stage-specific mortality and fertility. Top x axis represents age in years, whereas the bottom x axis represents time-scale normalized by the life expectancy (η) of the first non-seedbank stage (FNSS) of the species.

S1.9 *Cryptantha flava*

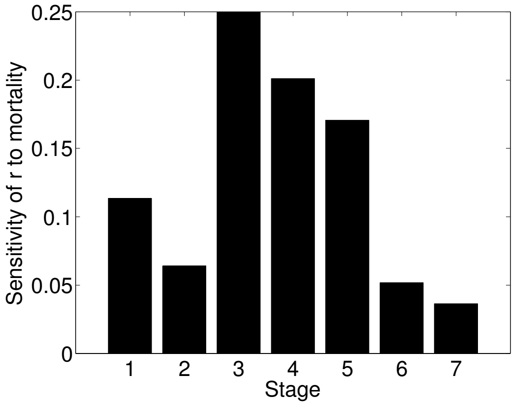

(a) Stage-specific mortality

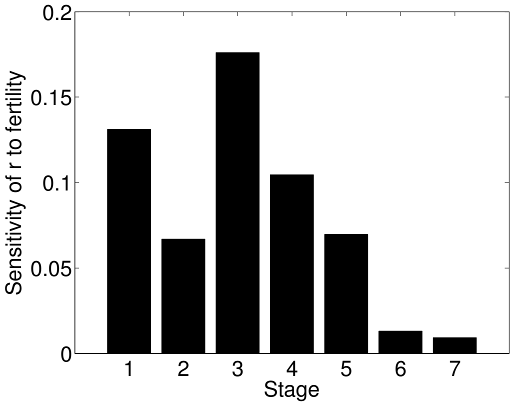

(b) Stage-specific fertility

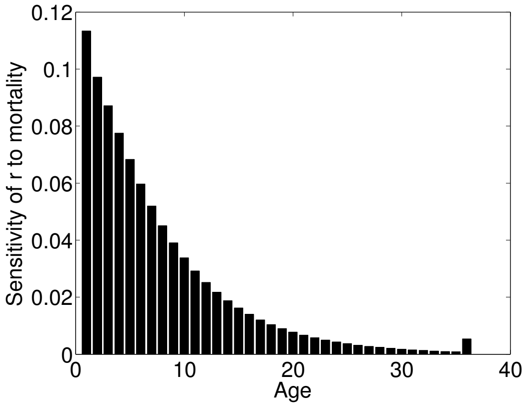

(c) Age-specific mortality

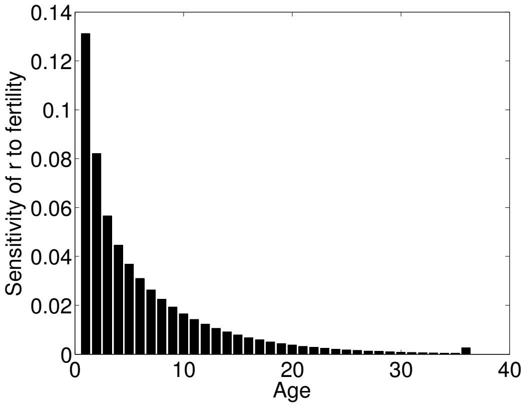

(d) Age-specific fertility

Figure 25: Selection gradients on age-specific and stage-specific mortality and fertility.

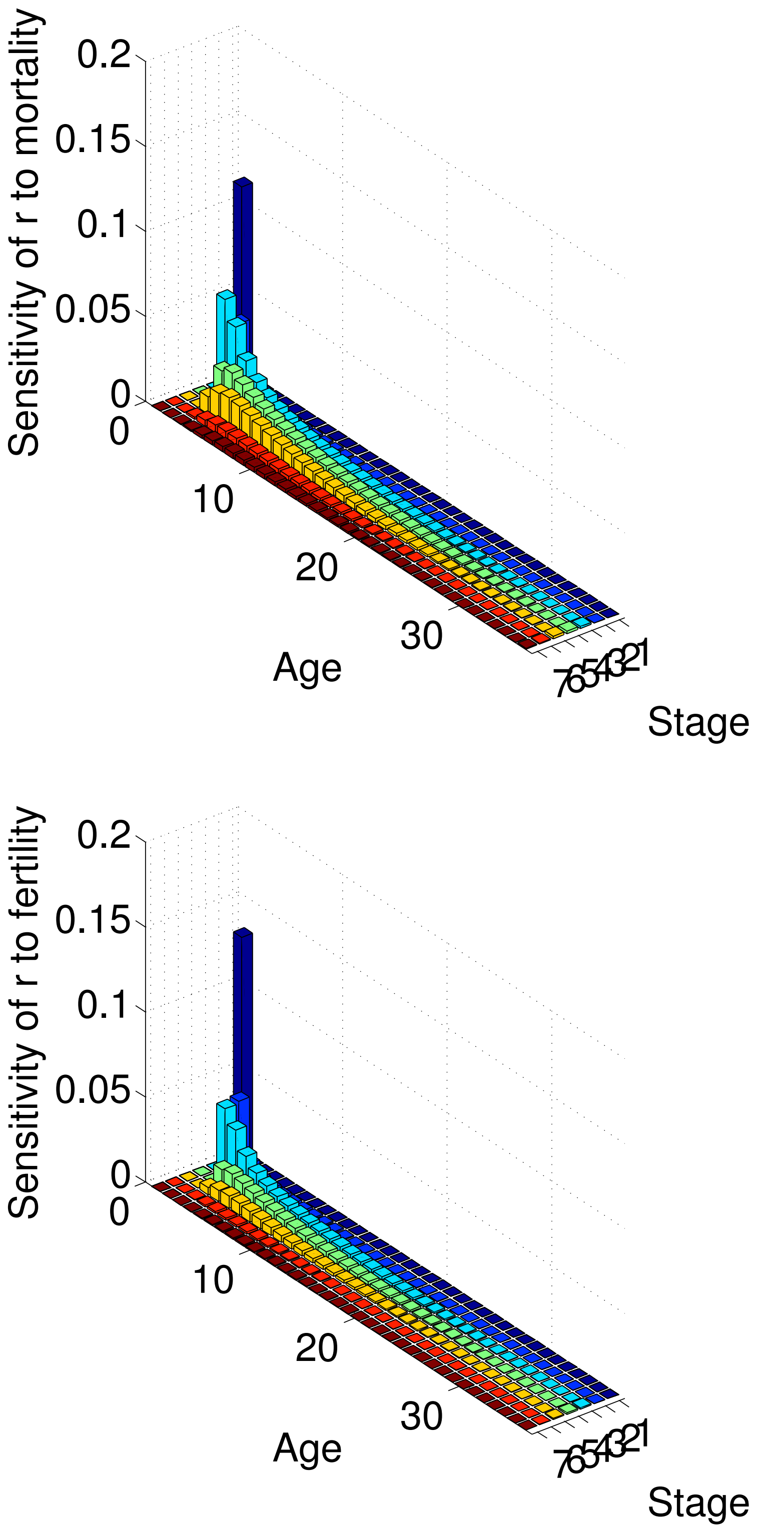

Figure 26: Selection gradients on age×stage-specific mortality and fertility.

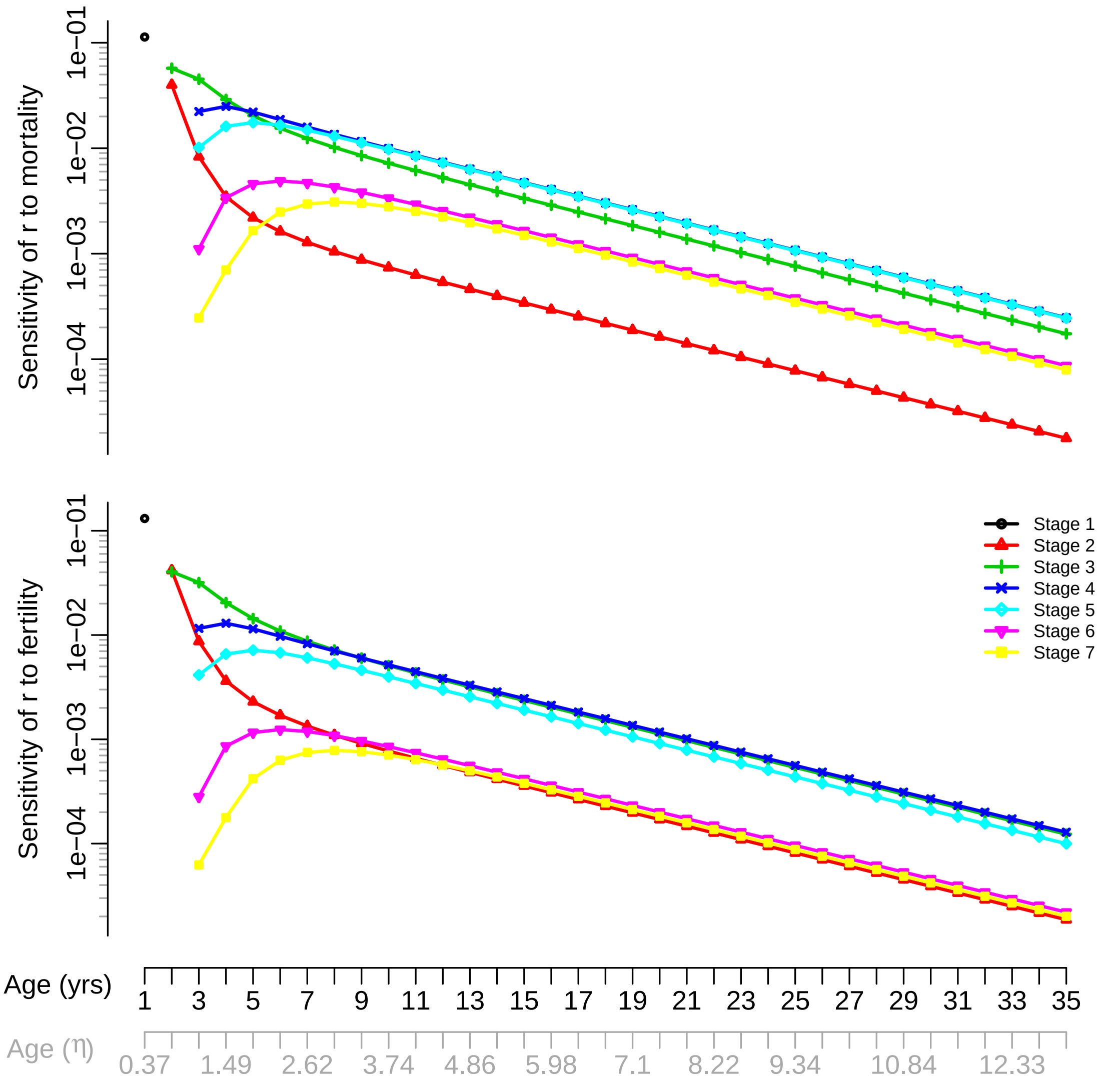

Figure 27: Selection gradients on age×stage-specific mortality and fertility. Top x axis represents age in years, whereas the bottom x axis represents time-scale normalized by the life expectancy (η) of the first non-seedbank stage (FNSS) of the species.

S1.10 *Cytisus scoparius*

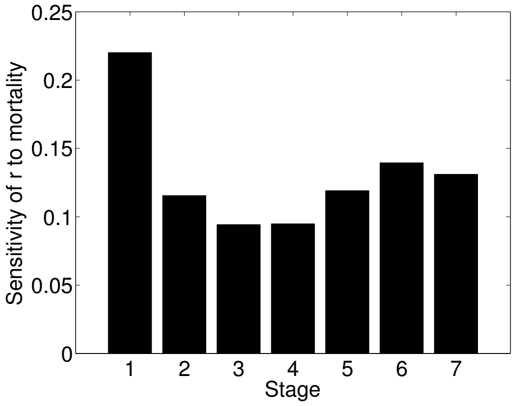

(a) Stage-specific mortality

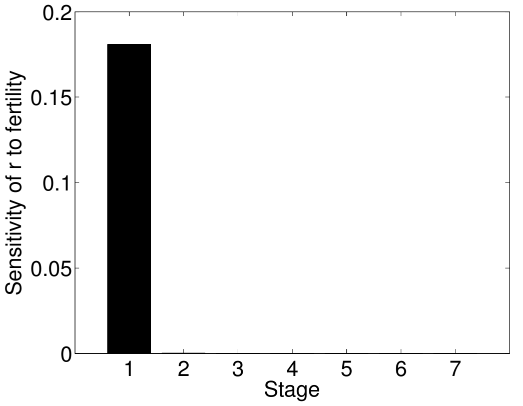

(b) Stage-specific fertility

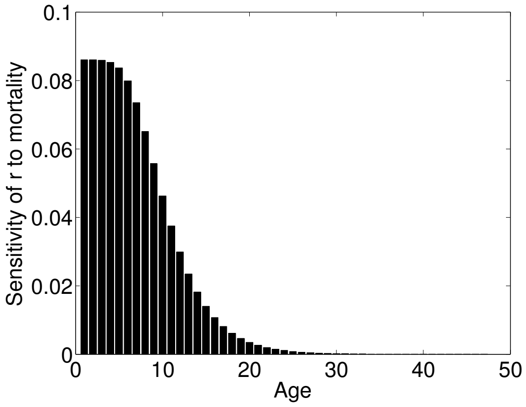

(c) Age-specific mortality

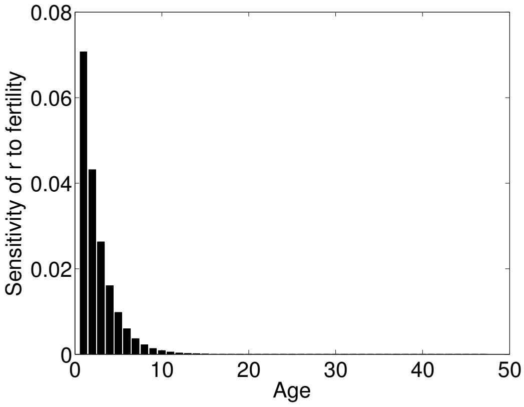

(d) Age-specific fertility

Figure 28: Selection gradients on age-specific and stage-specific mortality and fertility.

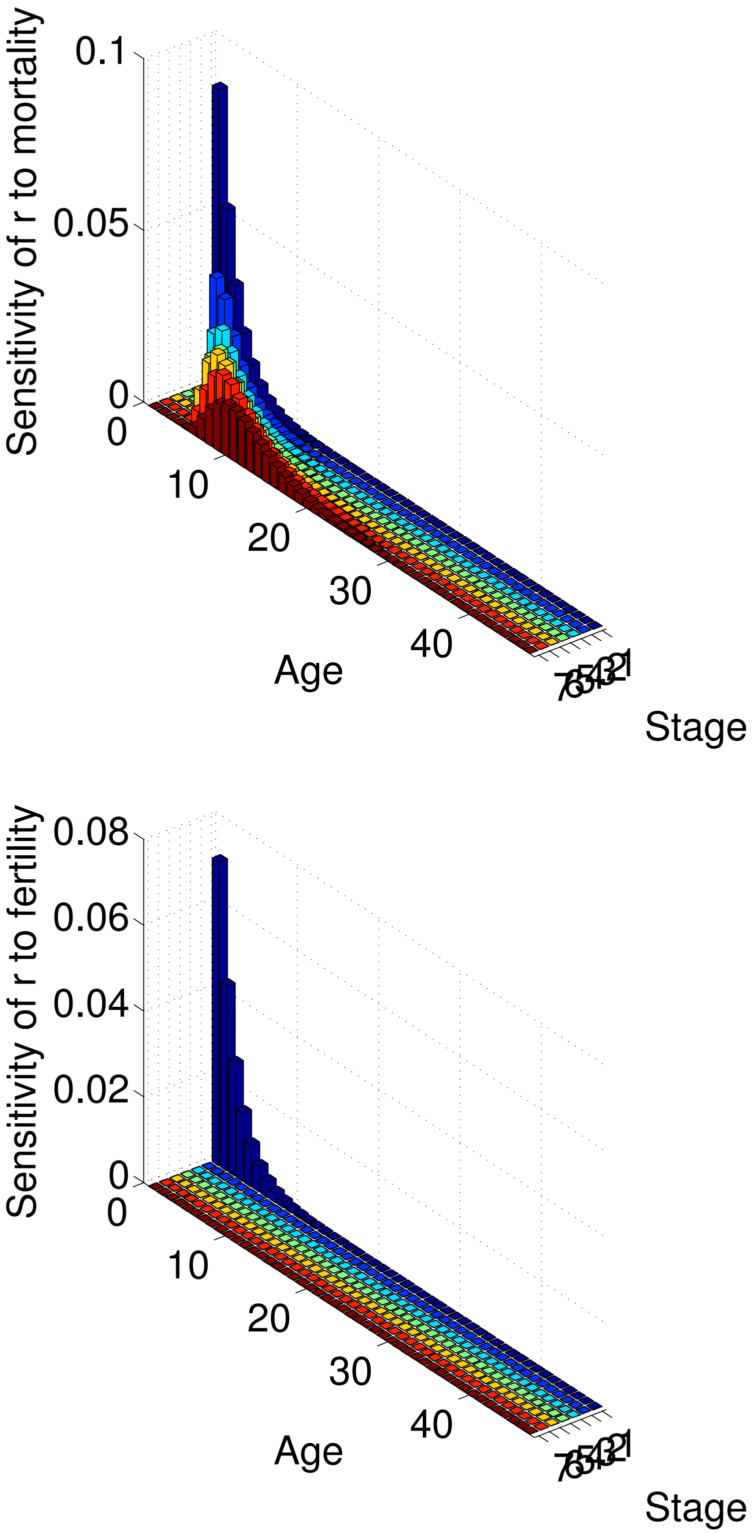

Figure 29: Selection gradients on age×stage-specific mortality and fertility.

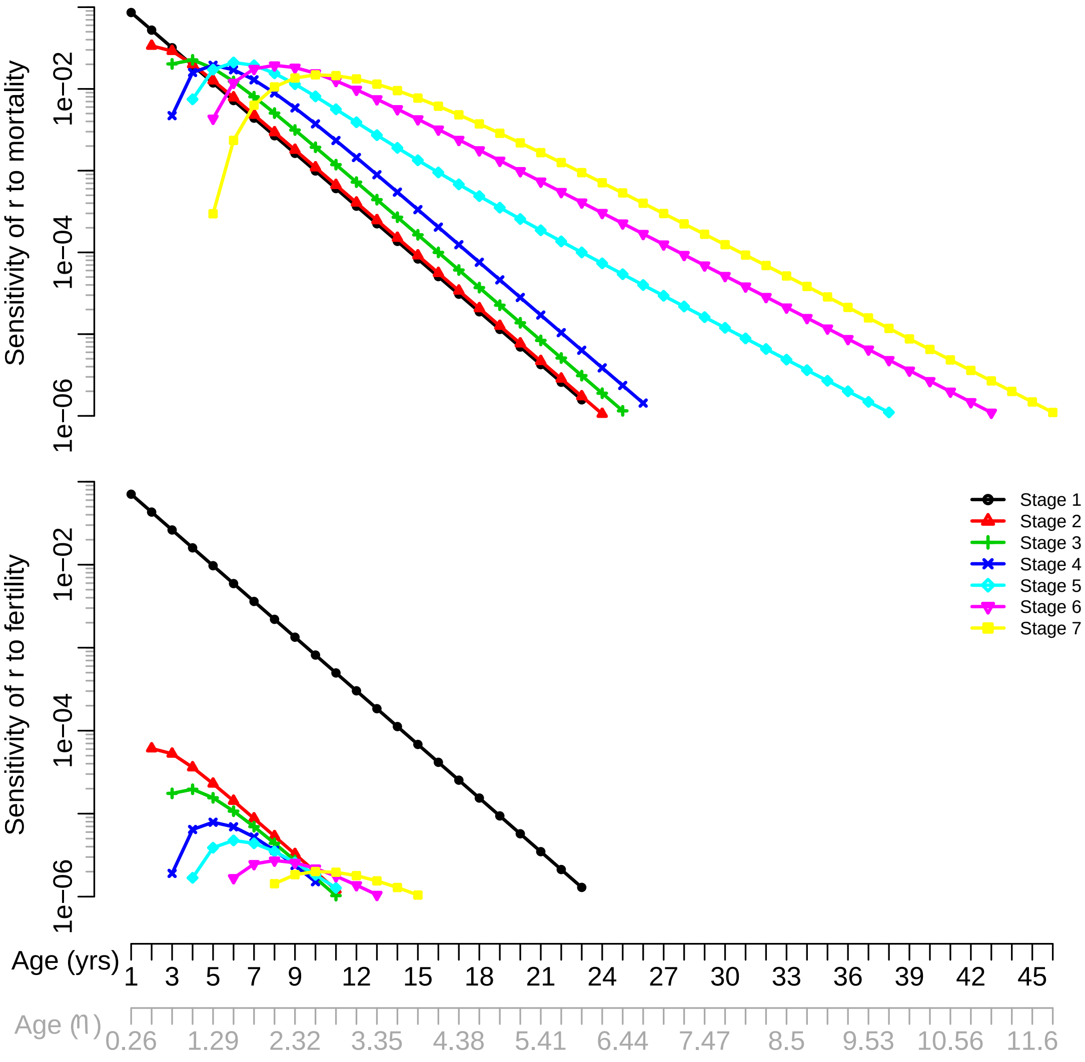

Figure 30: Selection gradients on age×stage-specific mortality and fertility. Top x axis represents age in years, whereas the bottom x axis represents time-scale normalized by the life expectancy (η) of the first non-seedbank stage (FNSS) of the species.

S1.11 *Dicerandra frutescens*

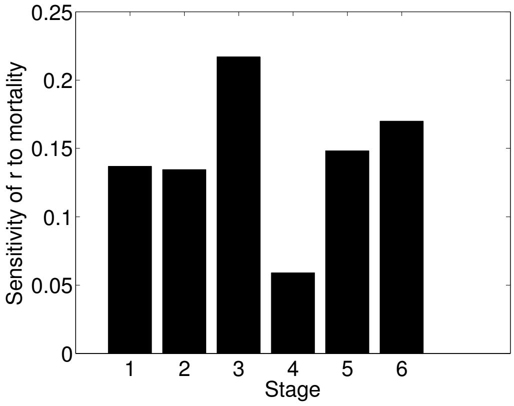

(a) Stage-specific mortality

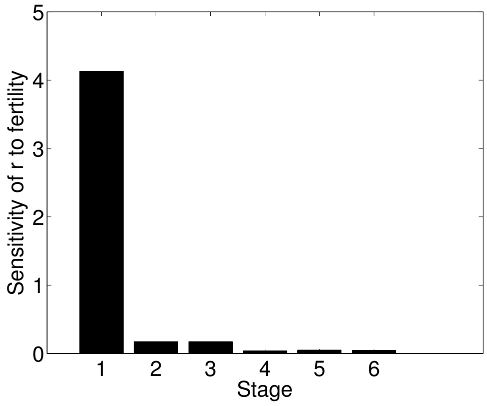

(b) Stage-specific fertility

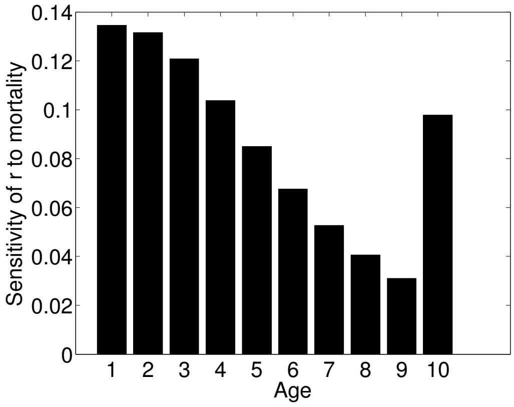

(c) Age-specific mortality

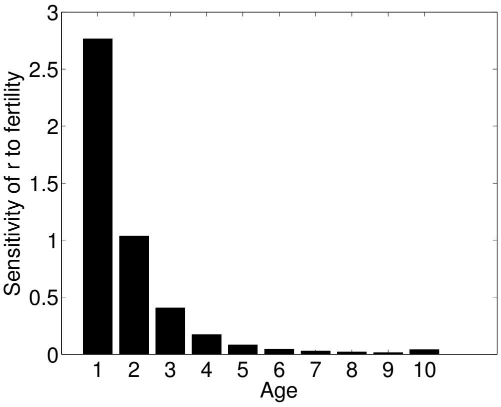

(d) Age-specific fertility

Figure 31: Selection gradients on age-specific and stage-specific mortality and fertility.

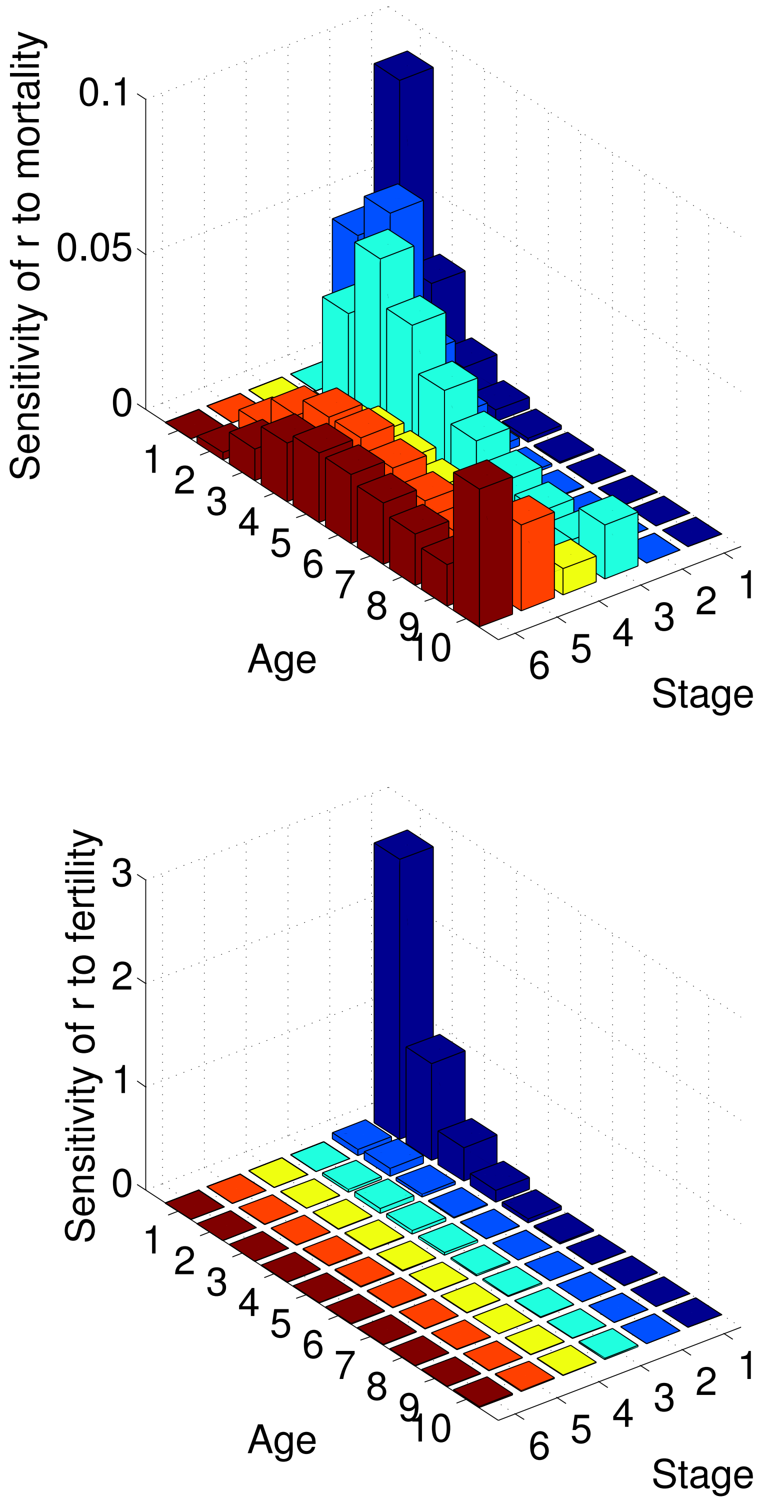

Figure 32: Selection gradients on age×stage-specific mortality and fertility.

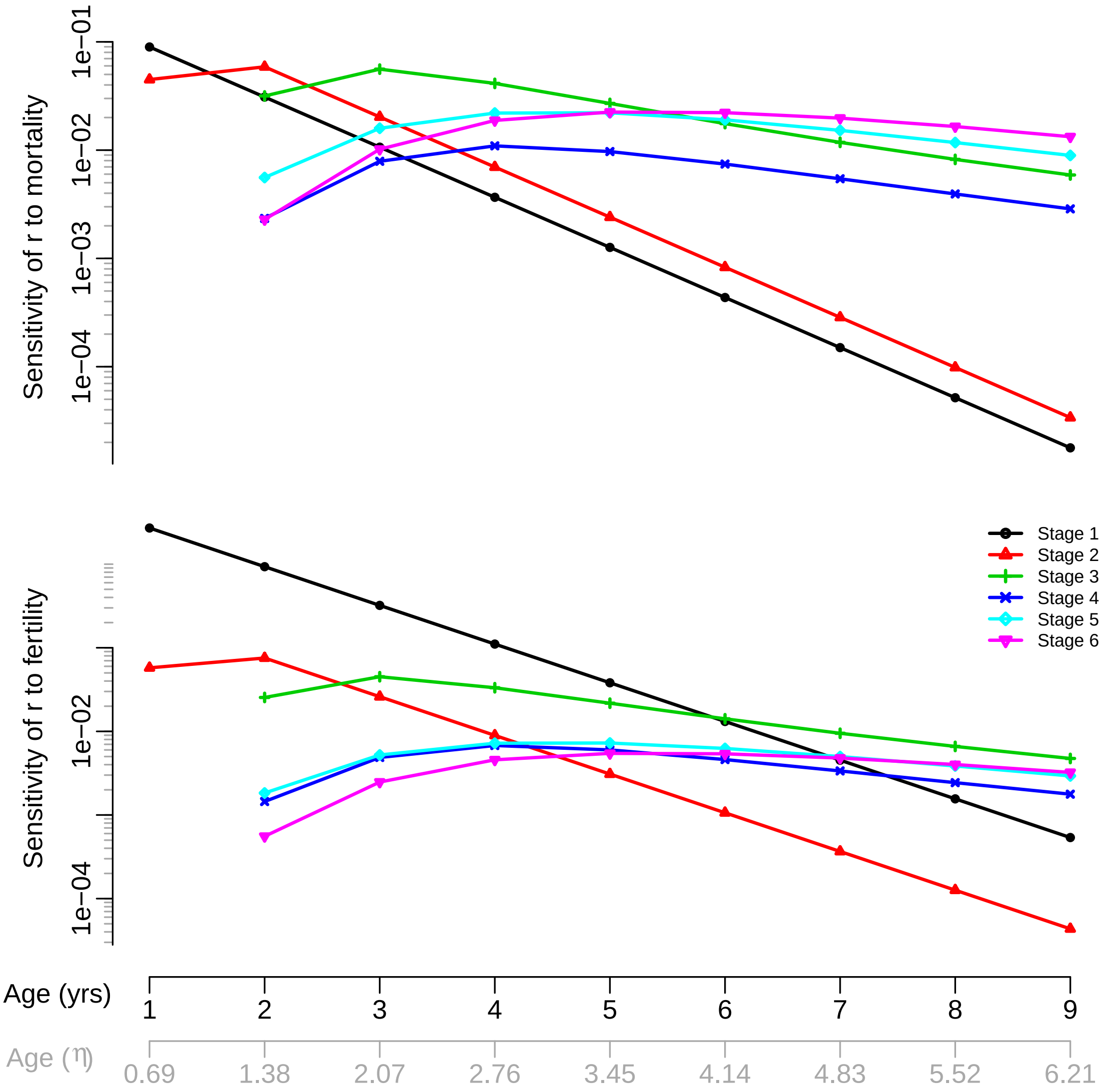

Figure 33: Selection gradients on age×stage-specific mortality and fertility. Top x axis represents age in years, whereas the bottom x axis represents time-scale normalized by the life expectancy (η) of the first non-seedbank stage (FNSS) of the species.

S1.12 *Dicymbe altsonii*

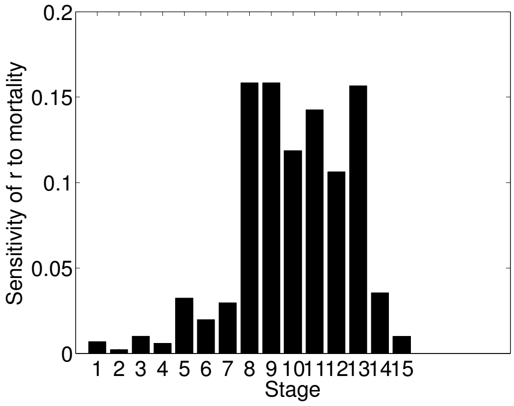

(a) Stage-specific mortality

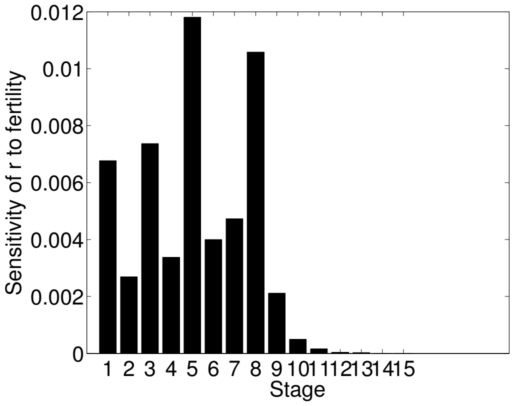

(b) Stage-specific fertility

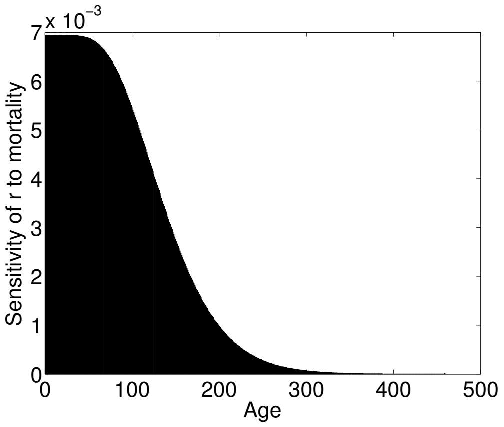

(c) Age-specific mortality

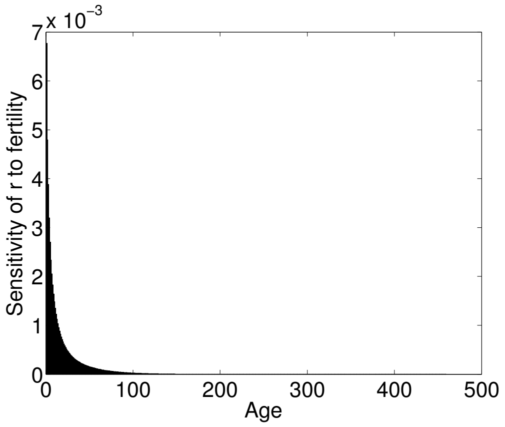

(d) Age-specific fertility

Figure 34: Selection gradients on age-specific and stage-specific mortality and fertility.

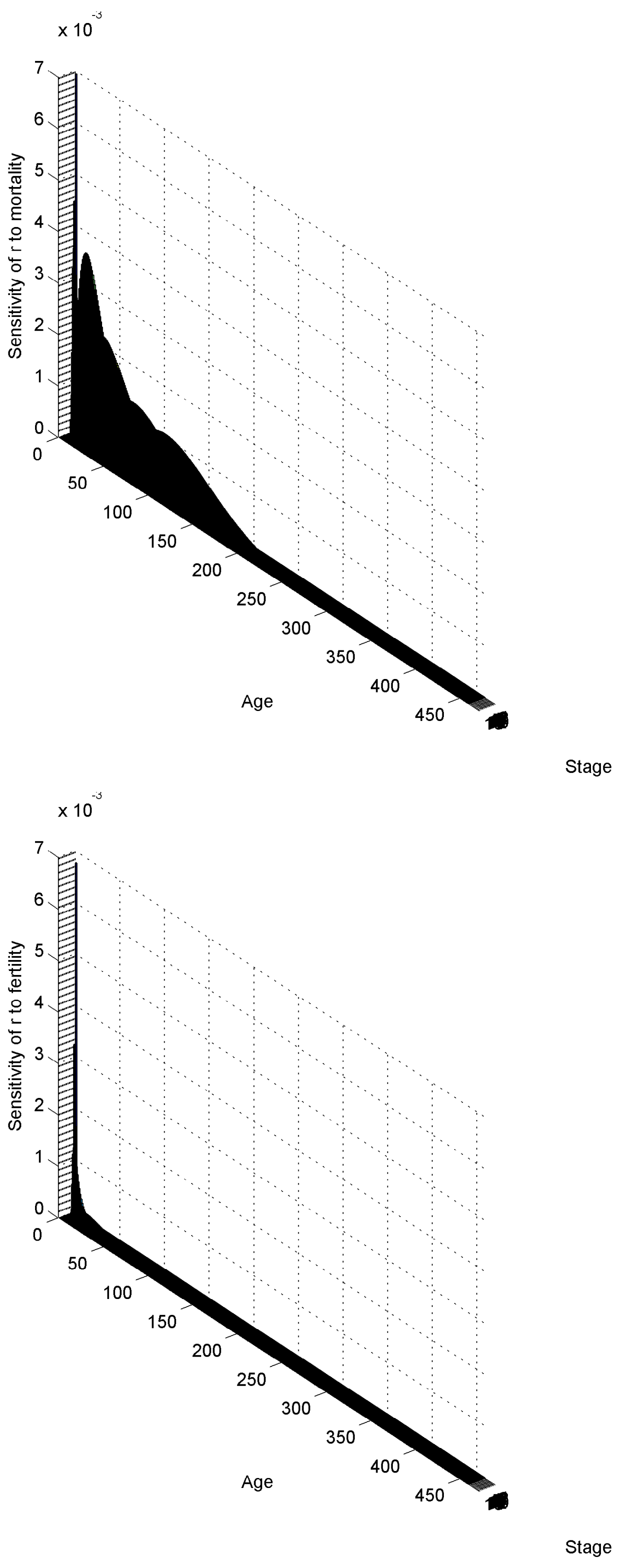

Figure 35: Selection gradients on age×stage-specific mortality and fertility.

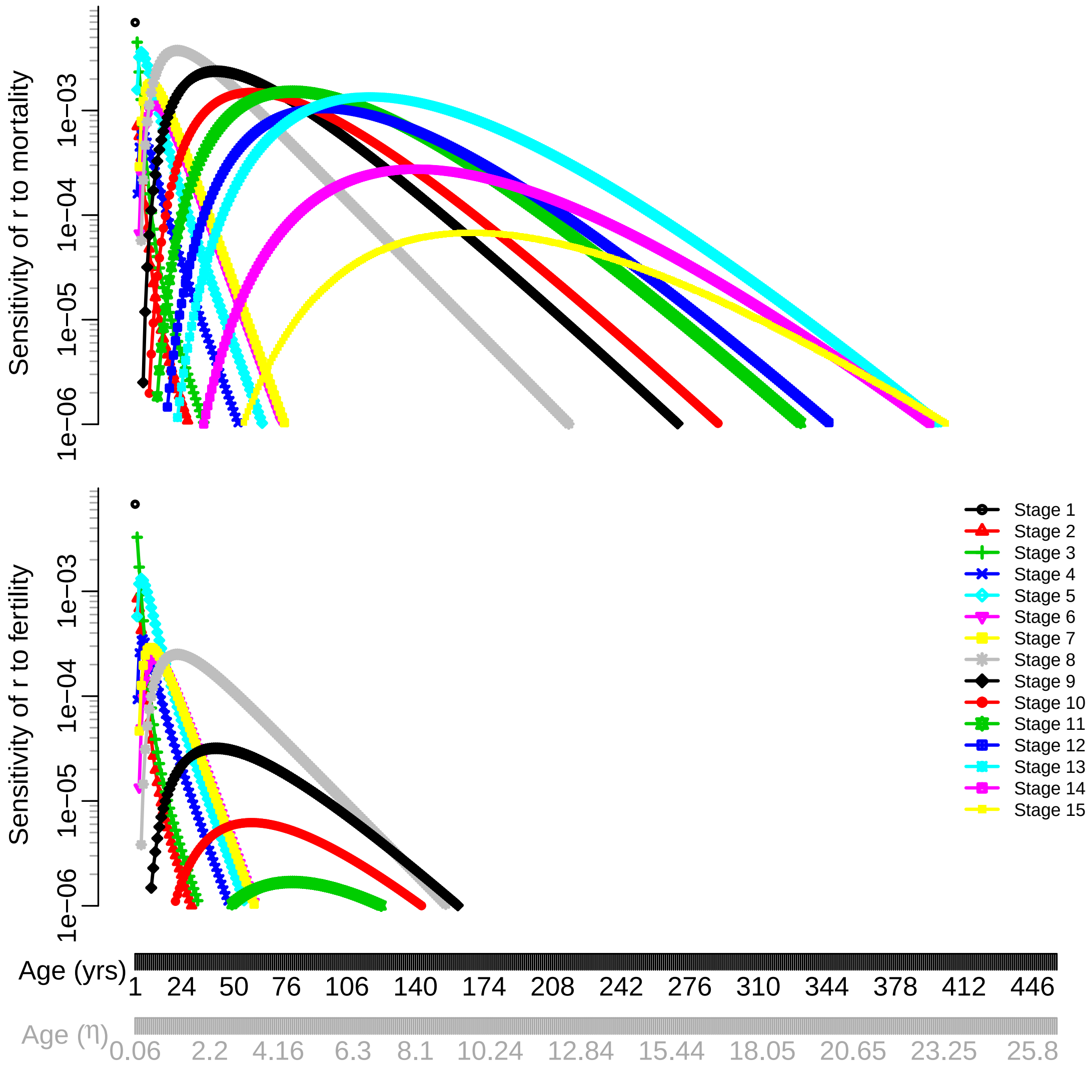

Figure 36: Selection gradients on age×stage-specific mortality and fertility. Top x axis represents age in years, whereas the bottom x axis represents time-scale normalized by the life expectancy (η) of the first non-seedbank stage (FNSS) of the species.

S1.13 *Geonoma macrostachys*

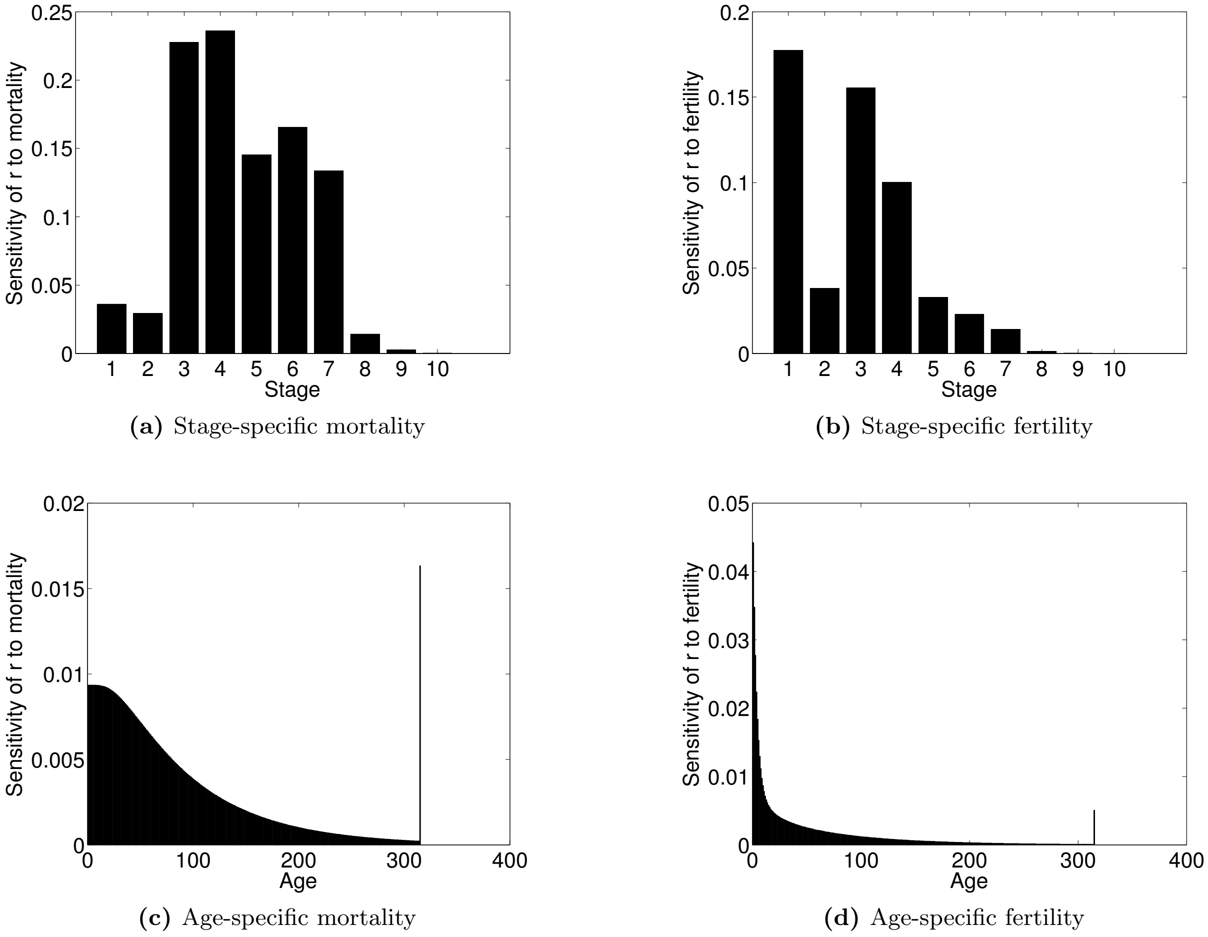

(a) Stage-specific mortality

(b) Stage-specific fertility

(c) Age-specific mortality

(d) Age-specific fertility

Figure 37: Selection gradients on age-specific and stage-specific mortality and fertility.

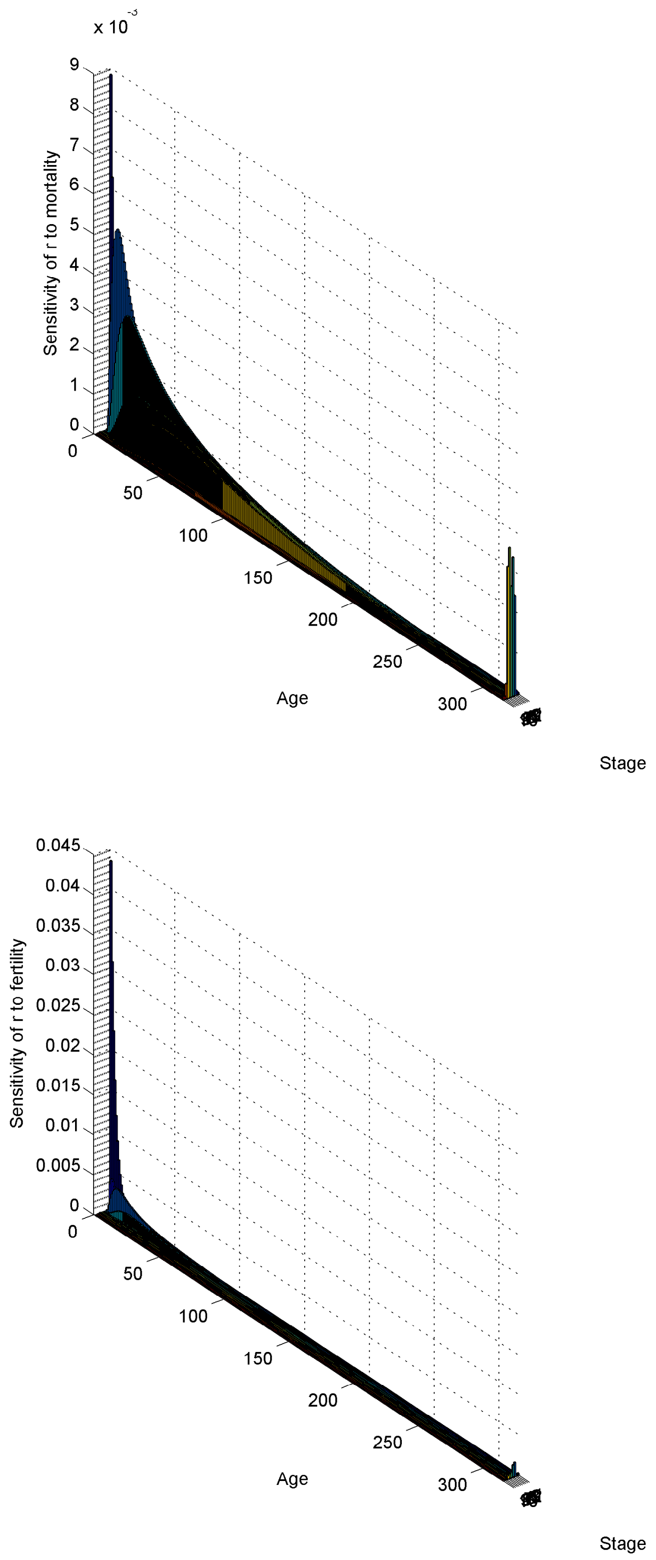

Figure 38: Selection gradients on age×stage-specific mortality and fertility.

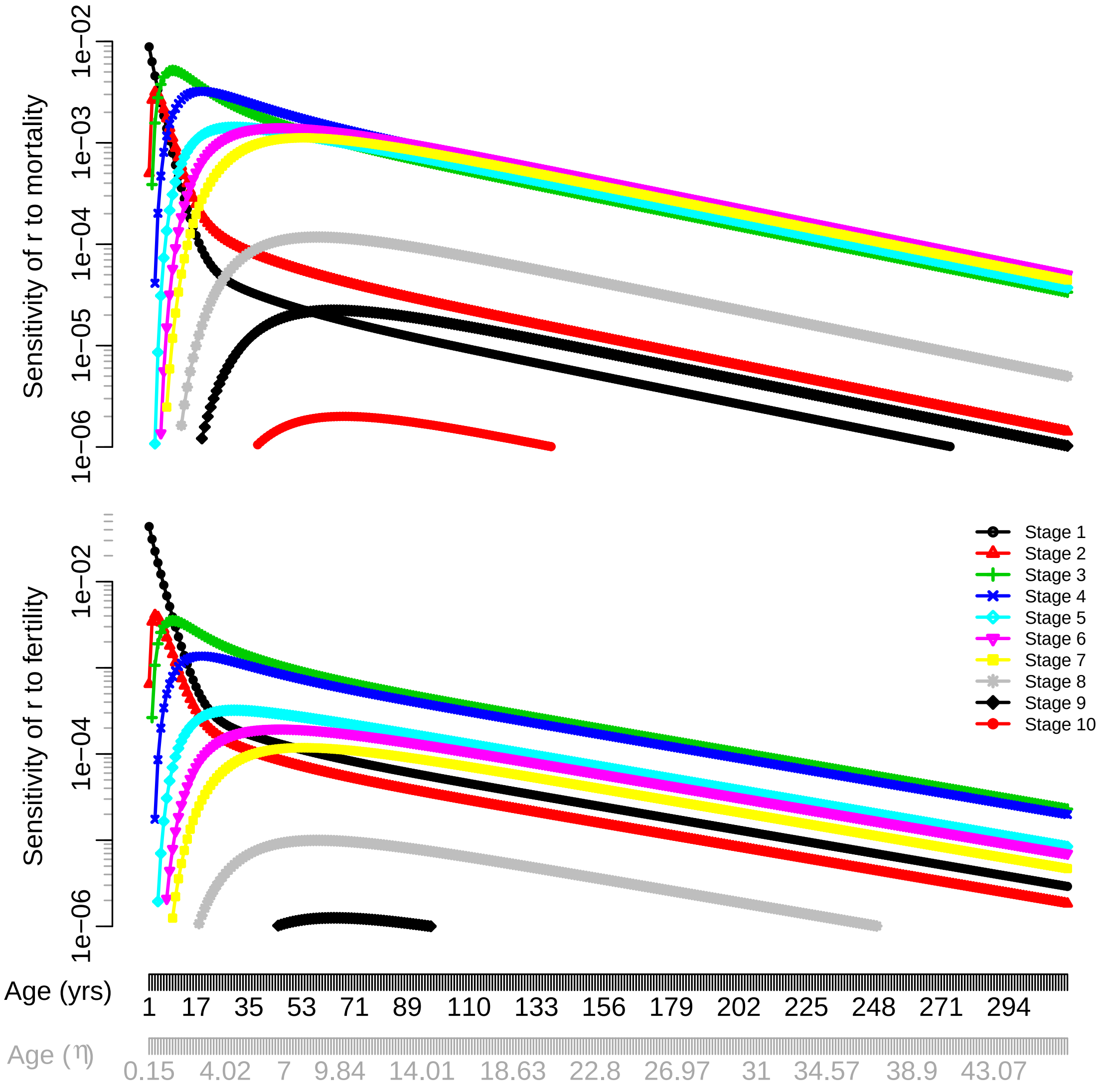

Figure 39: Selection gradients on age×stage-specific mortality and fertility. Top x axis represents age in years, whereas the bottom x axis represents time-scale normalized by the life expectancy (η) of the first non-seedbank stage (FNSS) of the species.

S1.14 *Hylocomium splendens*

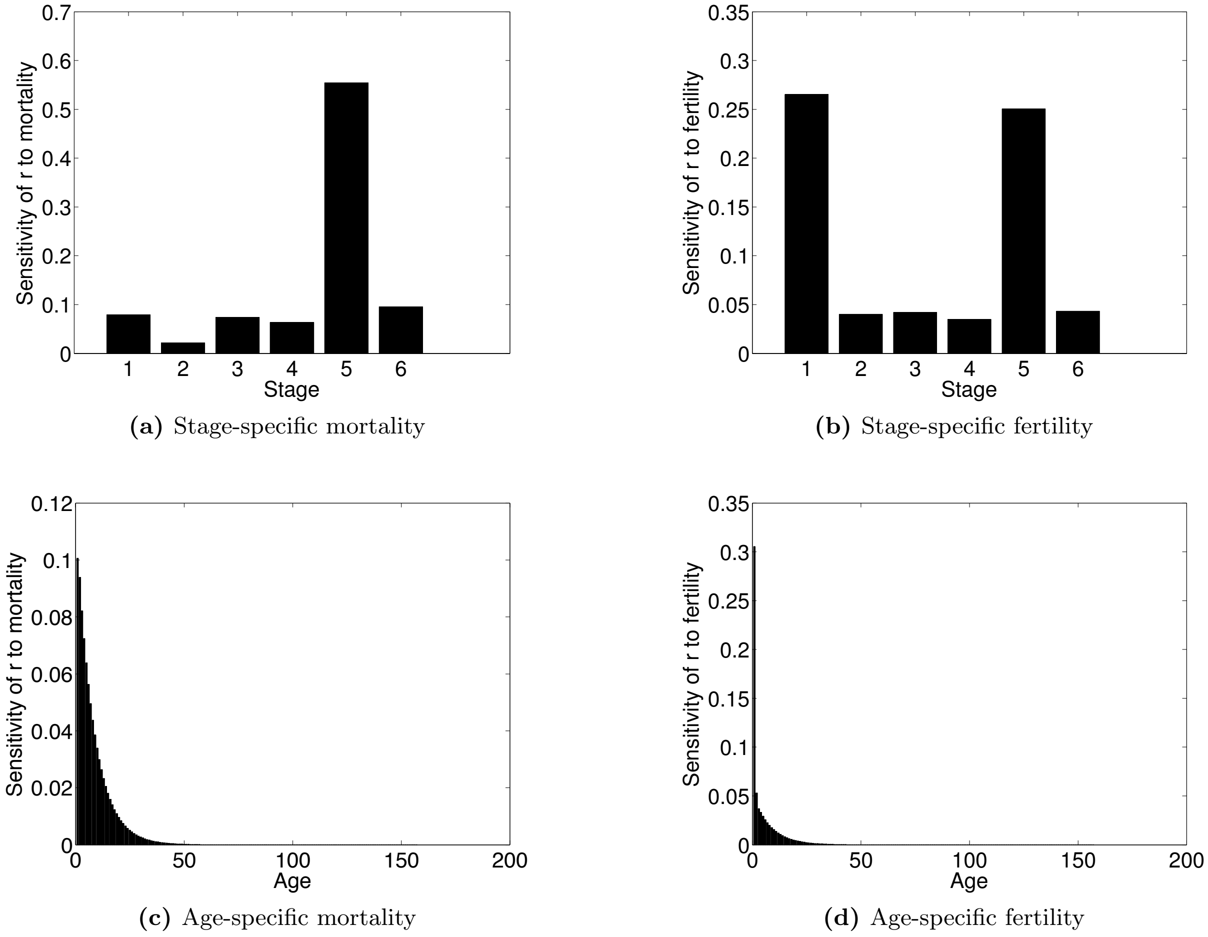

(a) Stage-specific mortality

(b) Stage-specific fertility

(c) Age-specific mortality

(d) Age-specific fertility

Figure 40: Selection gradients on age-specific and stage-specific mortality and fertility.

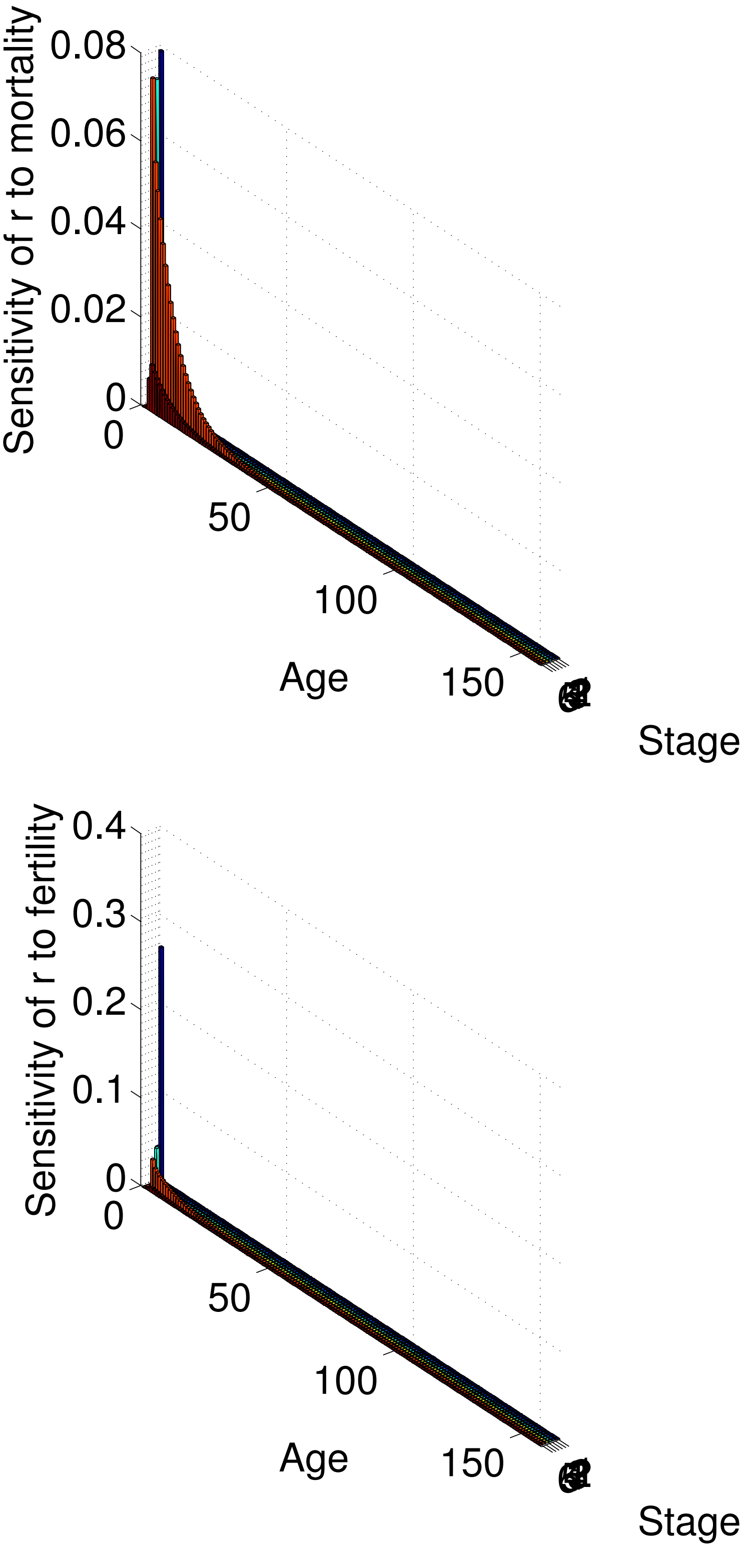

Figure 41: Selection gradients on age×stage-specific mortality and fertility.

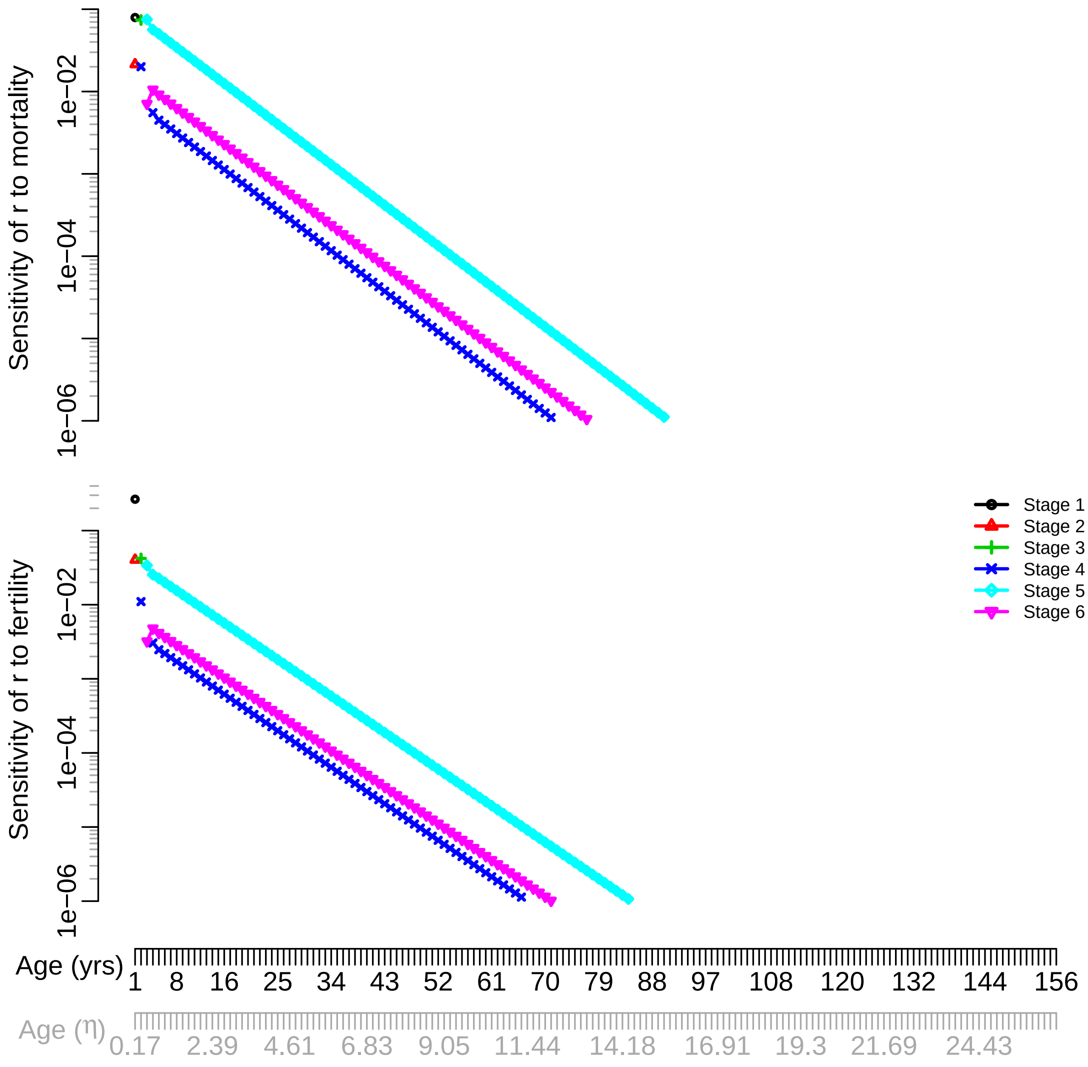

Figure 42: Selection gradients on age×stage-specific mortality and fertility. Top x axis represents age in years, whereas the bottom x axis represents time-scale normalized by the life expectancy (η) of the first non-seedbank stage (FNSS) of the species.

S1.15 *Hypericum cumulicola*

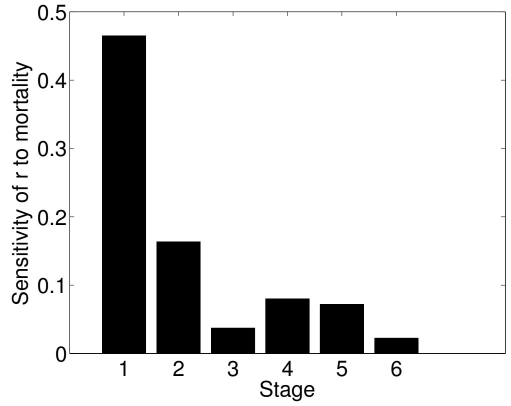

(a) Stage-specific mortality

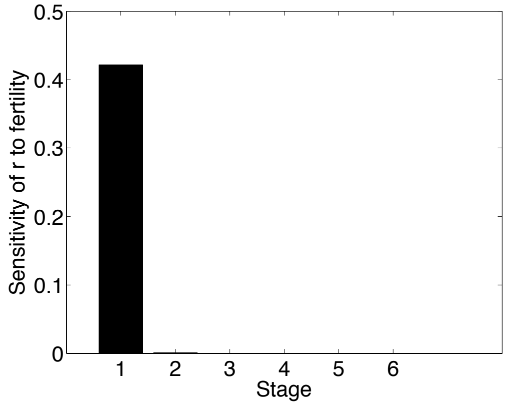

(b) Stage-specific fertility

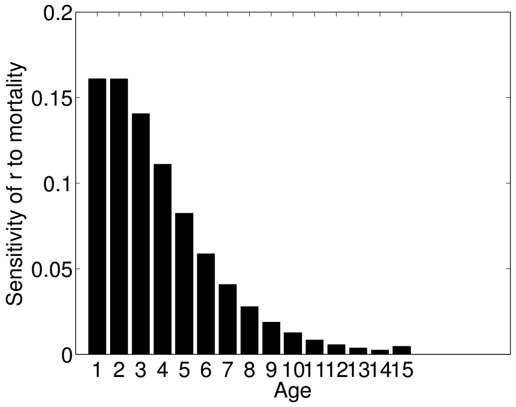

(c) Age-specific mortality

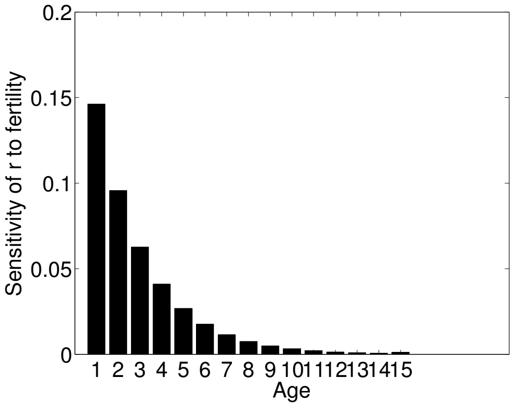

(d) Age-specific fertility

Figure 43: Selection gradients on age-specific and stage-specific mortality and fertility.

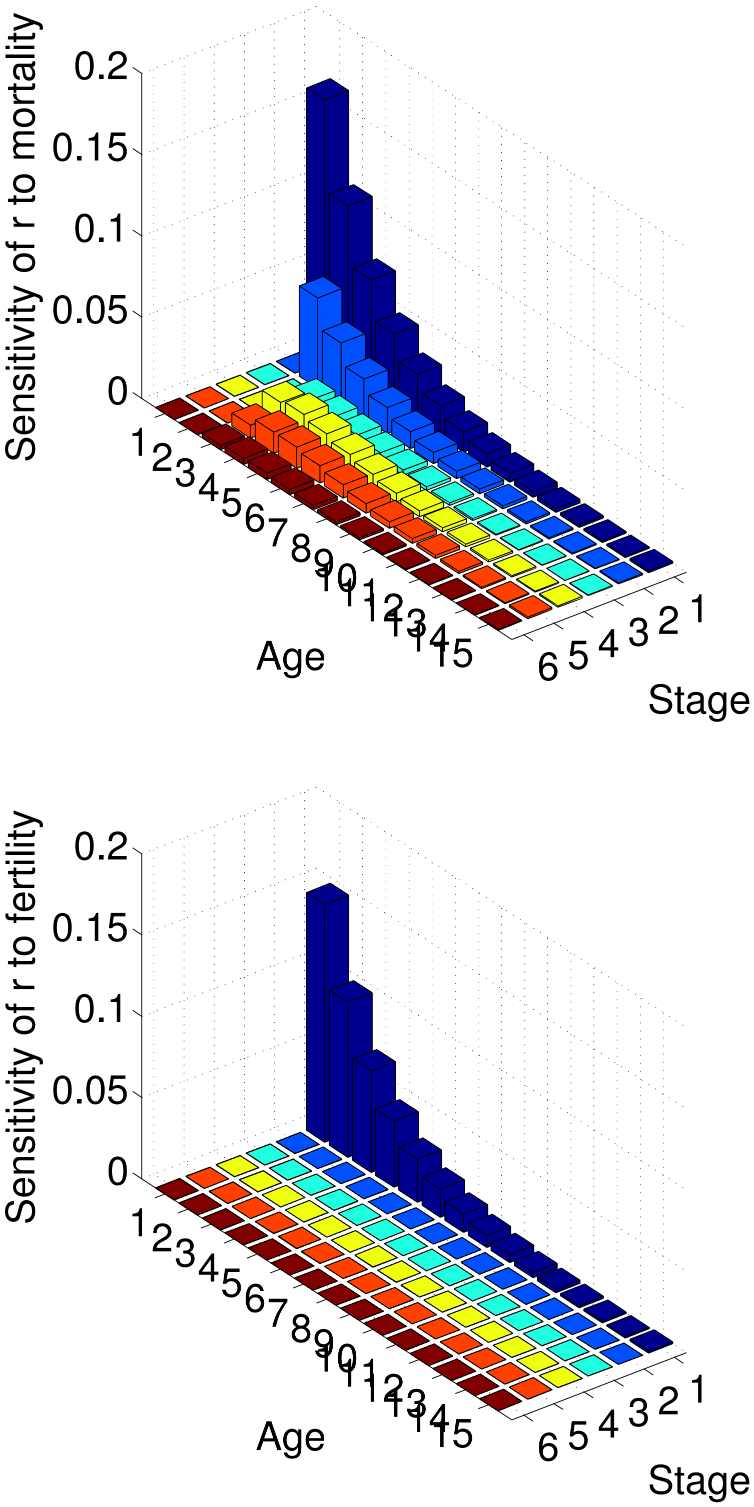

Figure 44: Selection gradients on age×stage-specific mortality and fertility.

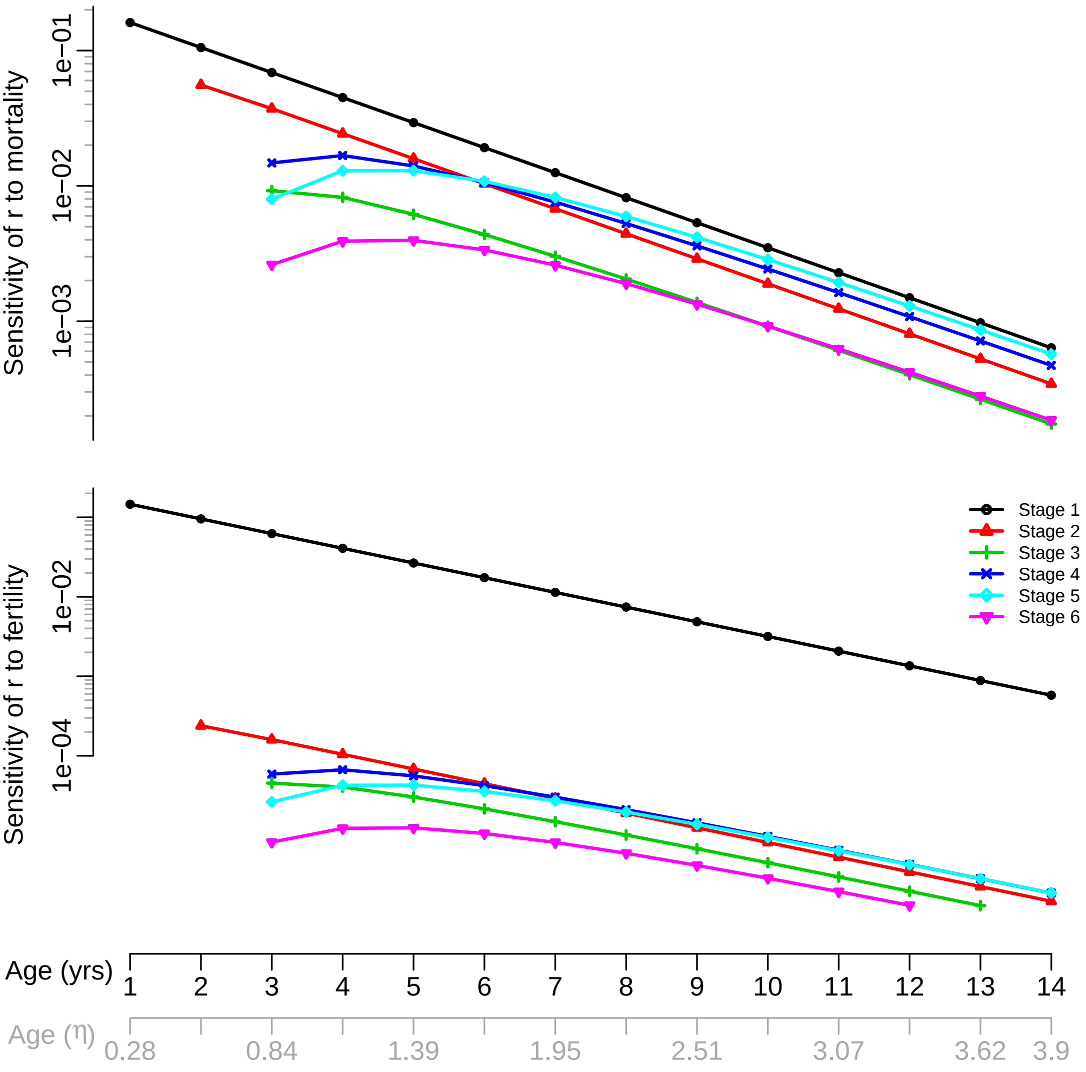

Figure 45: Selection gradients on age×stage-specific mortality and fertility. Top x axis represents age in years, whereas the bottom x axis represents time-scale normalized by the life expectancy (η) of the first non-seedbank stage (FNSS) of the species.

S1.16 *Iridaea splendens*

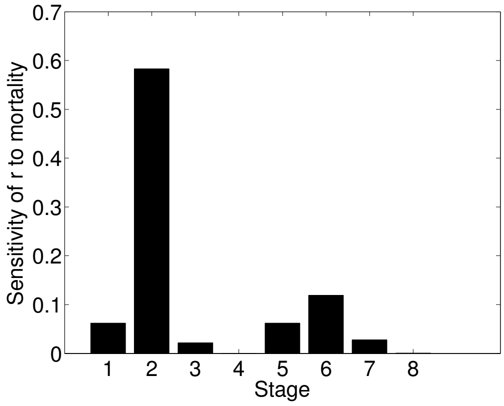

(a) Stage-specific mortality

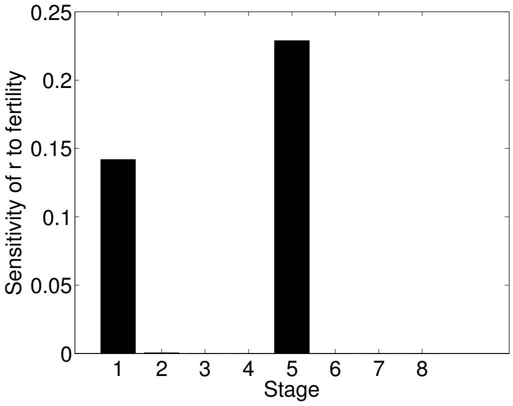

(b) Stage-specific fertility

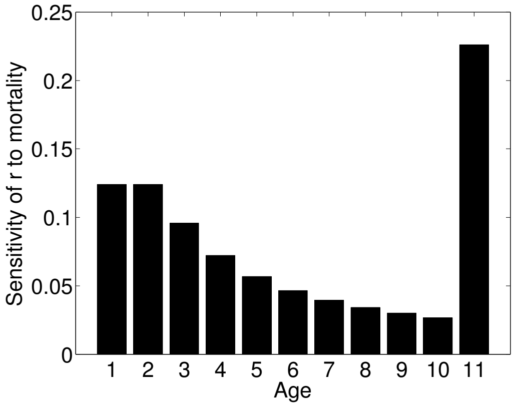

(c) Age-specific mortality

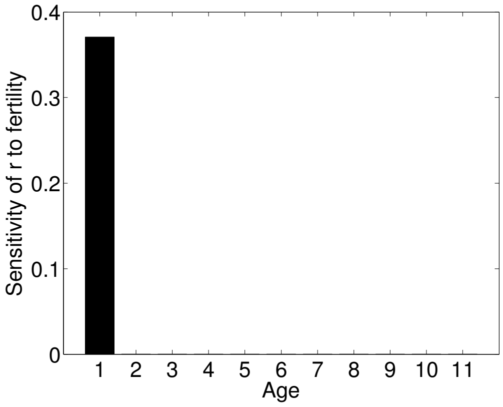

(d) Age-specific fertility

Figure 46: Selection gradients on age-specific and stage-specific mortality and fertility.

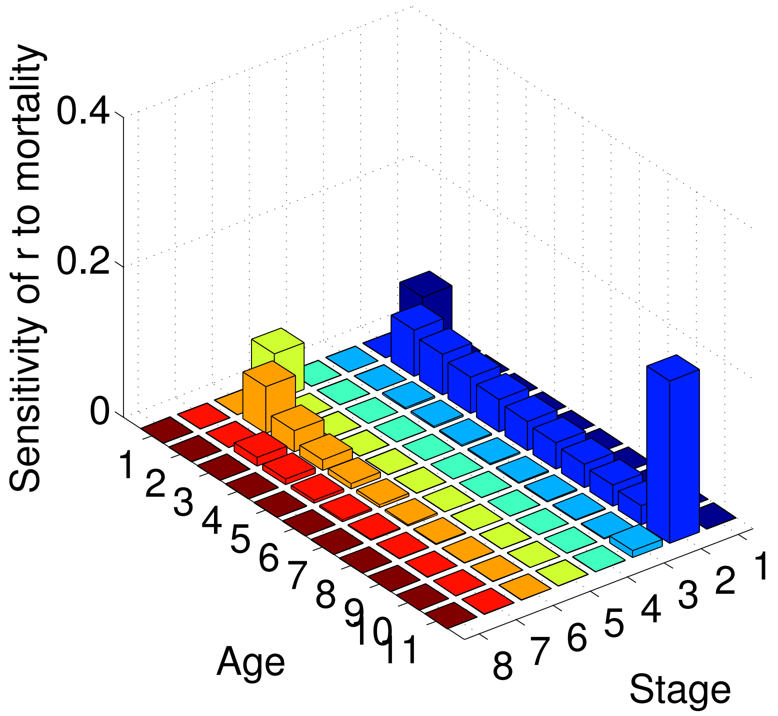

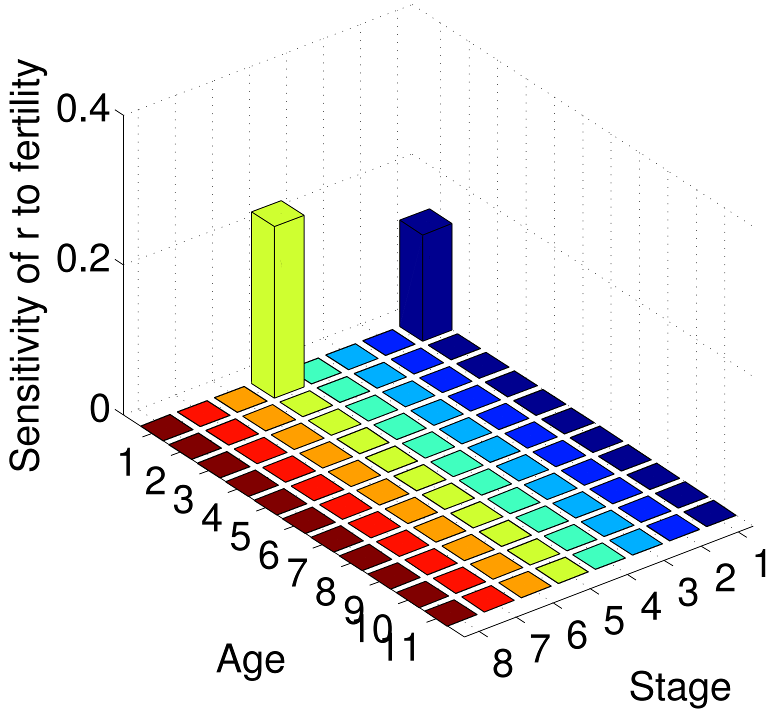

Figure 47: Selection gradients on age×stage-specific mortality and fertility.

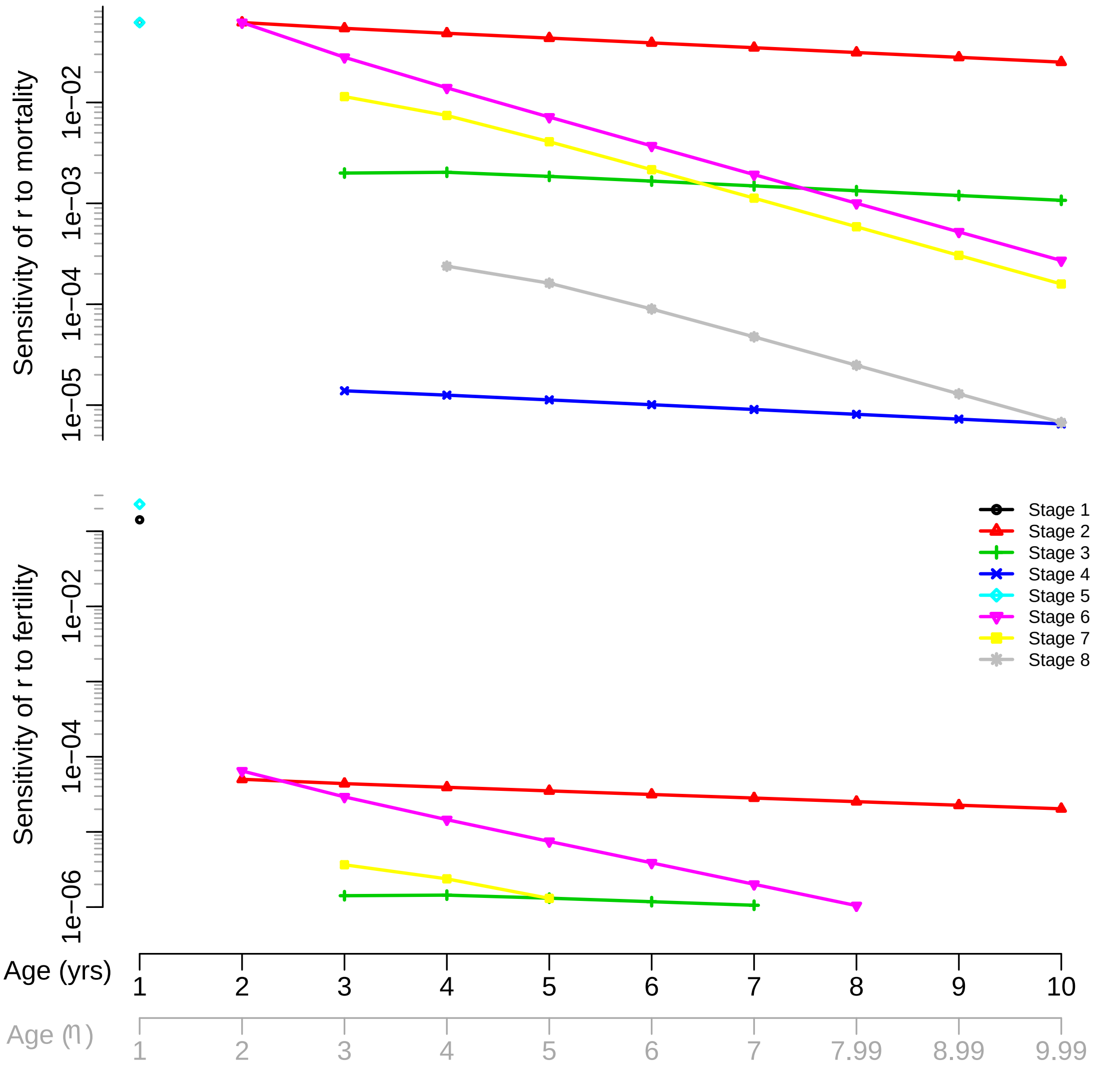

Figure 48: Selection gradients on age×stage-specific mortality and fertility. Top x axis represents age in years, whereas the bottom x axis represents time-scale normalized by the life expectancy (η) of the first non-seedbank stage (FNSS) of the species.

S1.17 *Laminaria digitata*

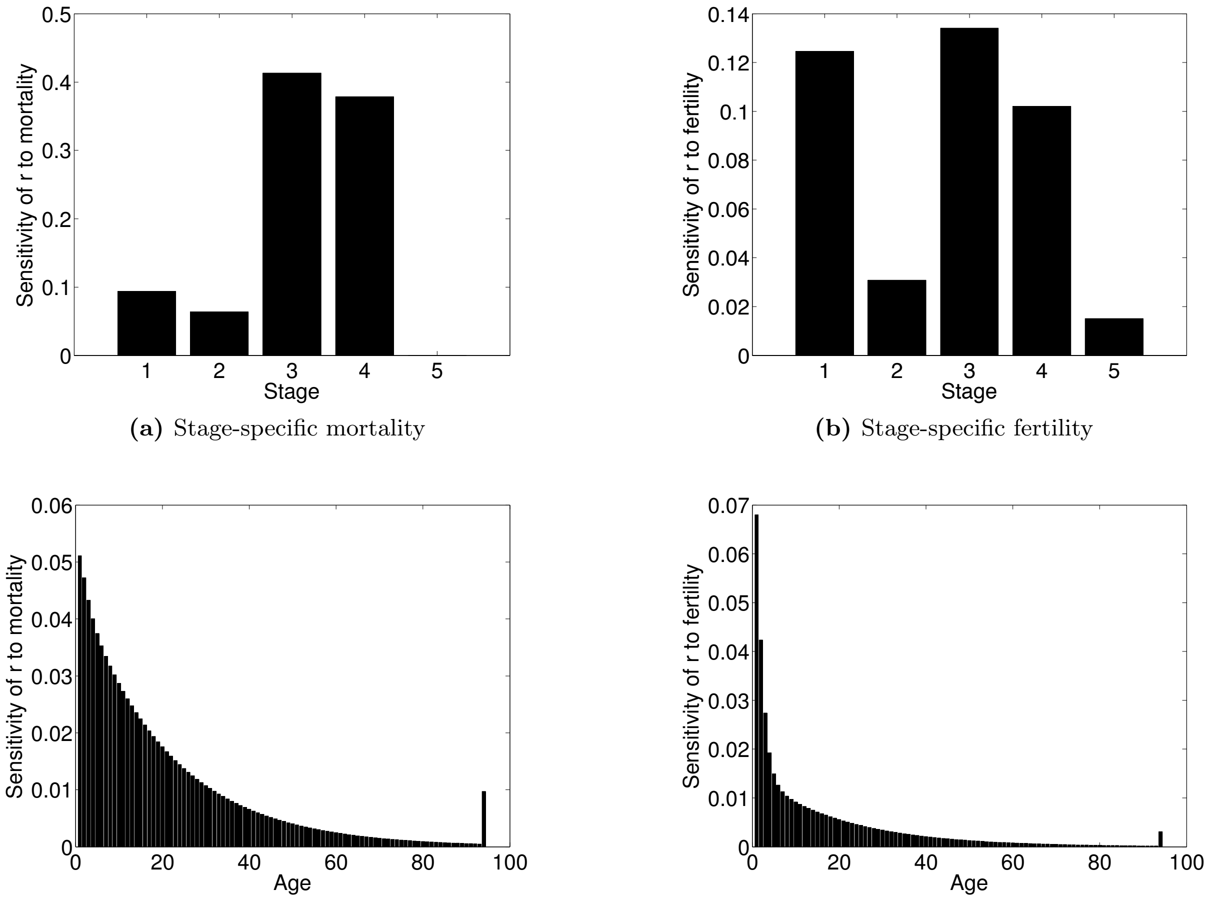

(a) Stage-specific mortality

(b) Stage-specific fertility

(c) Age-specific mortality

(d) Age-specific fertility

Figure 49: Selection gradients on age-specific and stage-specific mortality and fertility.

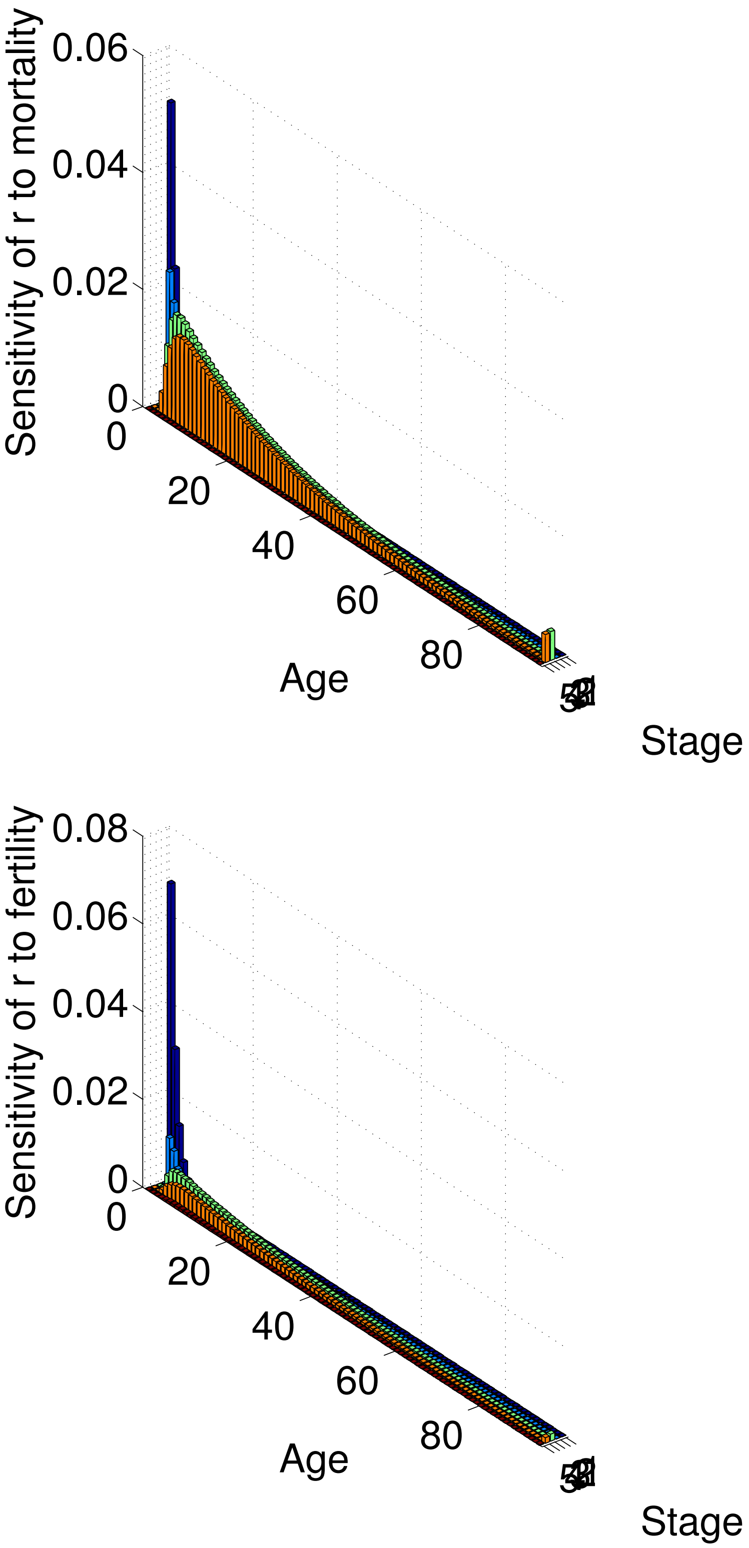

Figure 50: Selection gradients on age×stage-specific mortality and fertility.

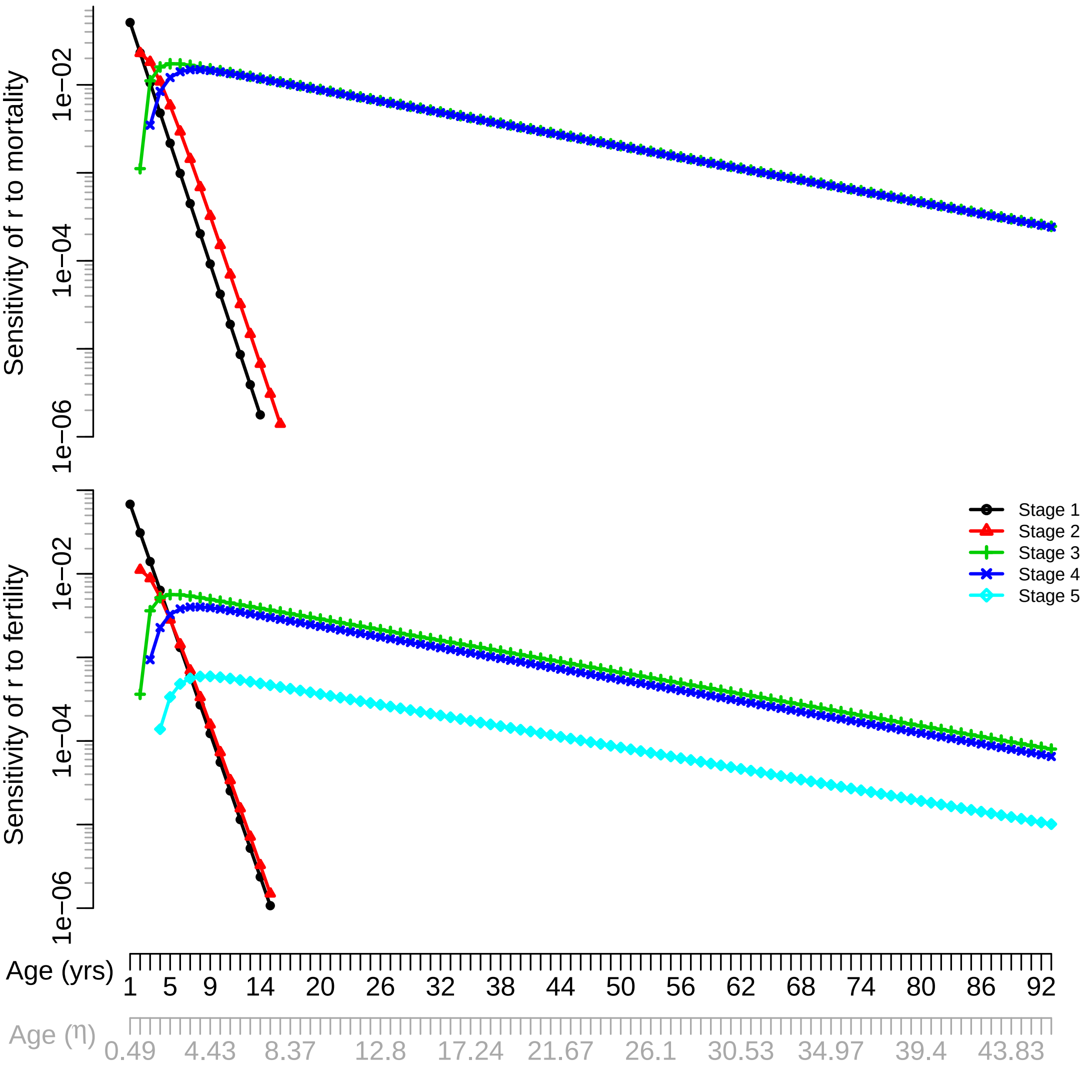

Figure 51: Selection gradients on age×stage-specific mortality and fertility. Top x axis represents age in years, whereas the bottom x axis represents time-scale normalized by the life expectancy (η) of the first non-seedbank stage (FNSS) of the species.

S1.18 *Lupinus arboreus*

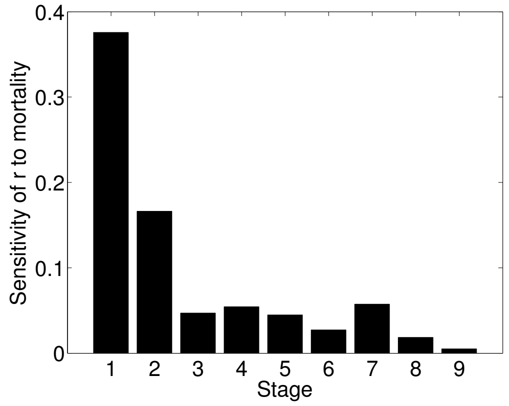

(a) Stage-specific mortality

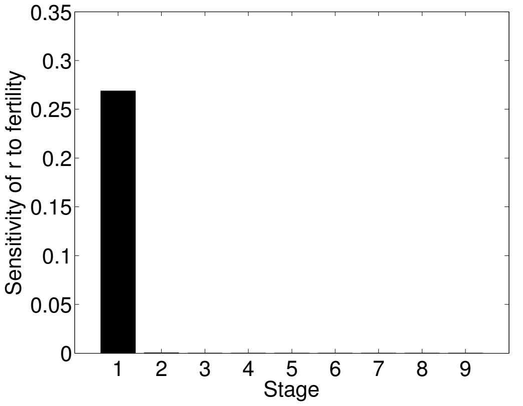

(b) Stage-specific fertility

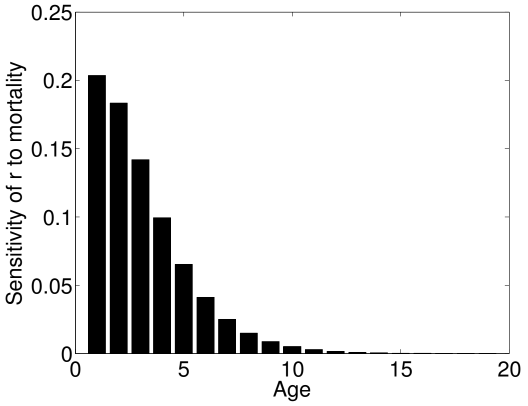

(c) Age-specific mortality

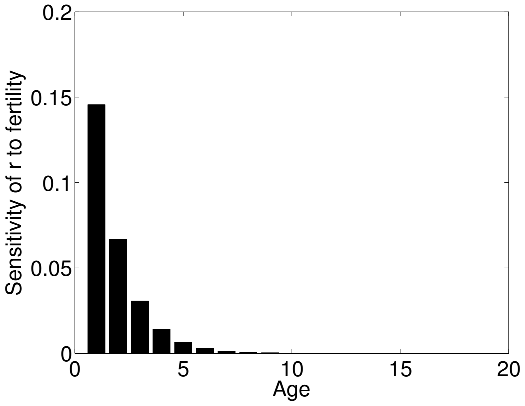

(d) Age-specific fertility

Figure 52: Selection gradients on age-specific and stage-specific mortality and fertility.

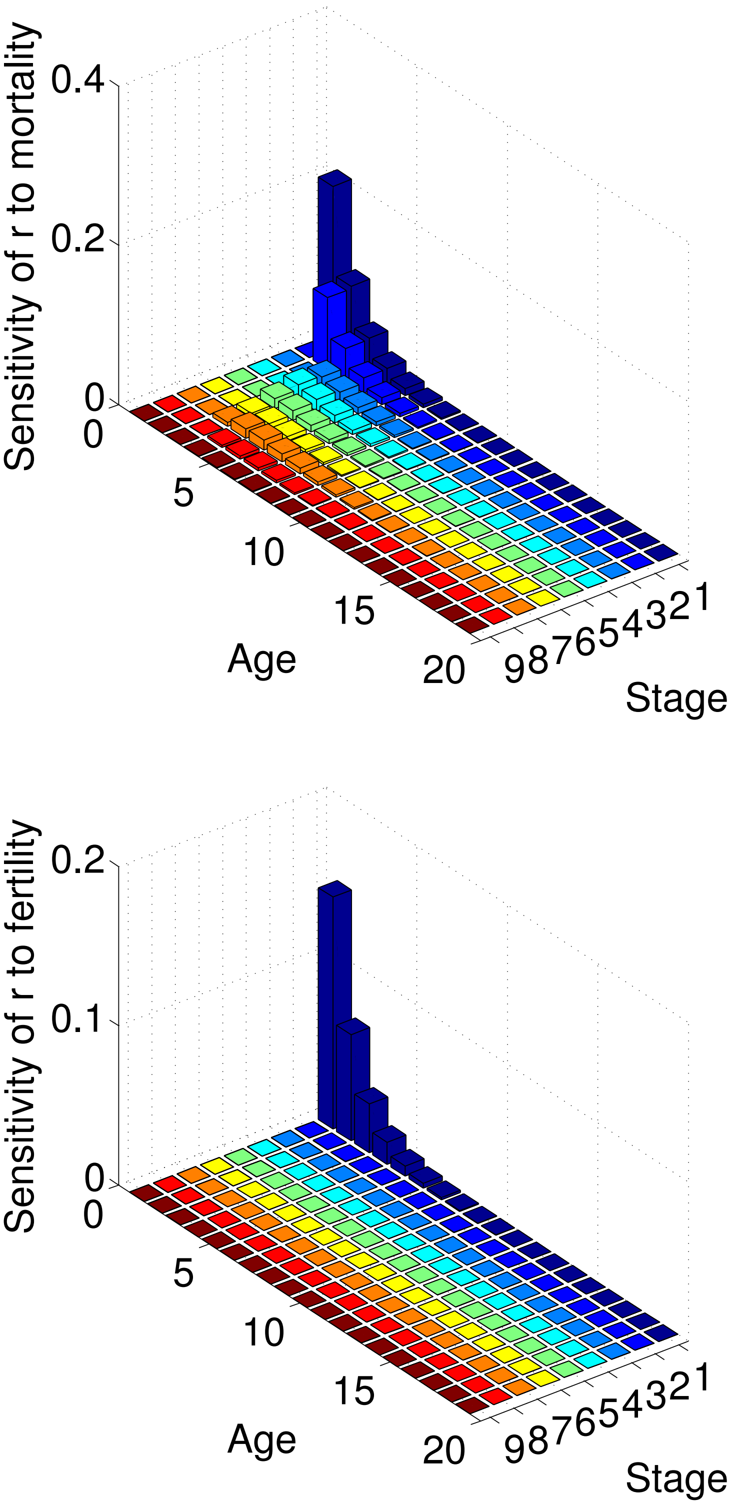

Figure 53: Selection gradients on age×stage-specific mortality and fertility.

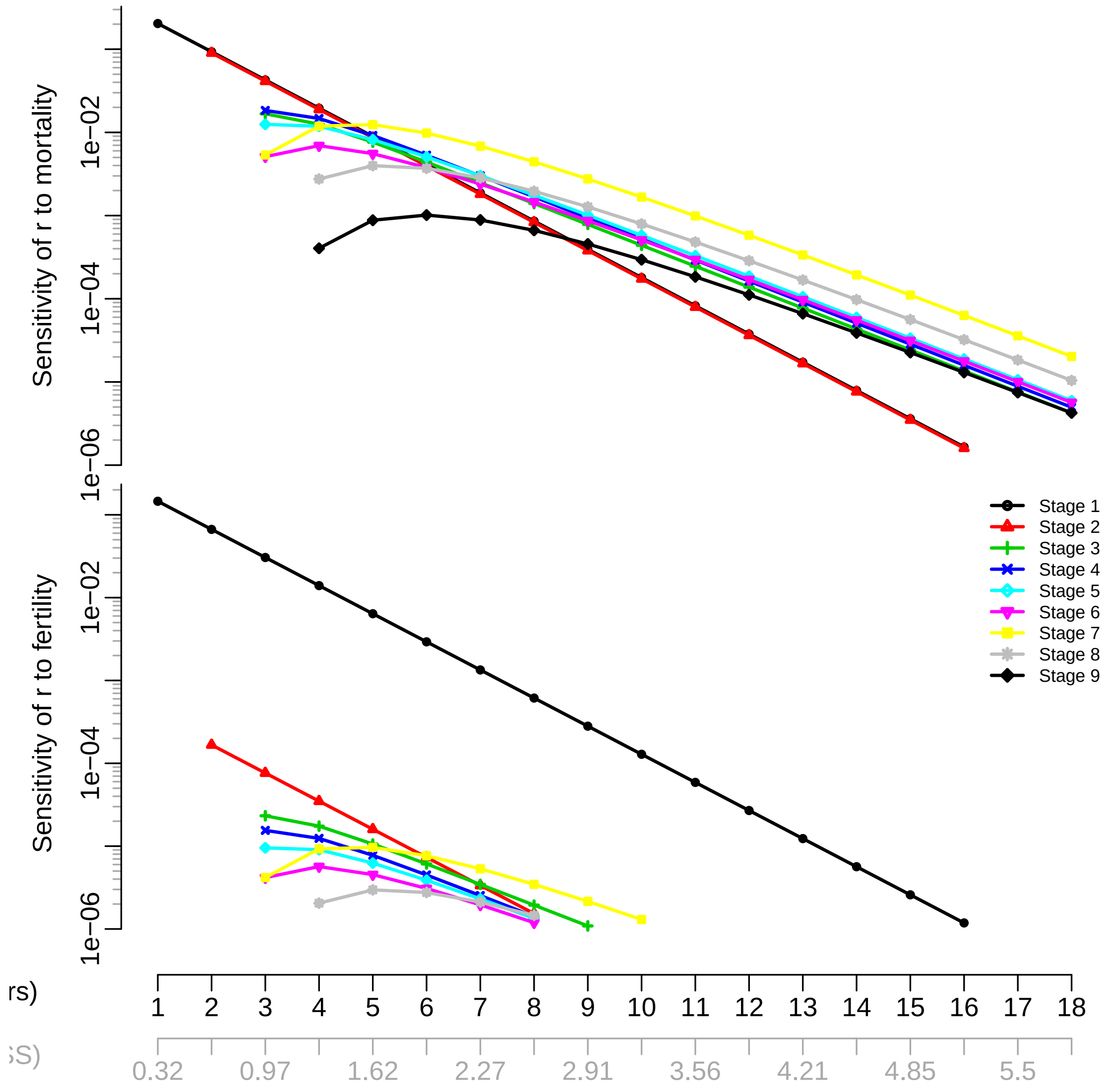

Figure 54: Selection gradients on age×stage-specific mortality and fertility. Top x axis represents age in years, whereas the bottom x axis represents time-scale normalized by the life expectancy (η) of the first non-seedbank stage (FNSS) of the species.

S1.19 *Machaerium cuspidatum*

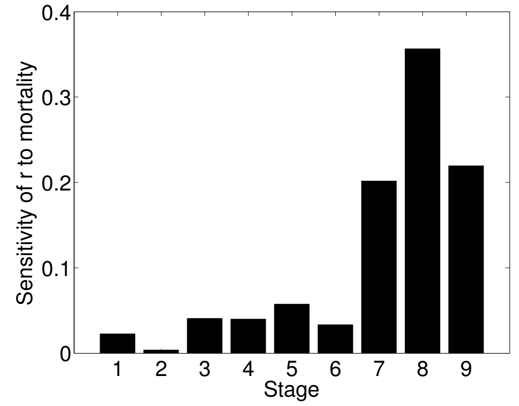

(a) Stage-specific mortality

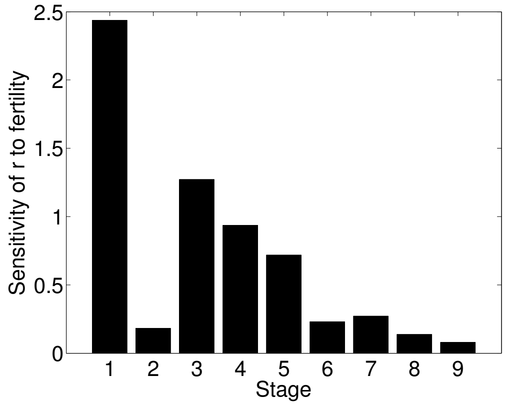

(b) Stage-specific fertility

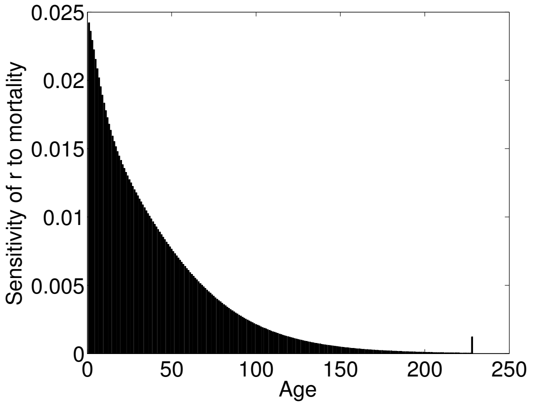

(c) Age-specific mortality

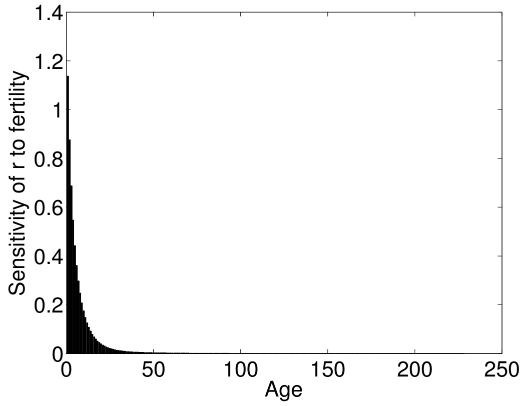

(d) Age-specific fertility

Figure 55: Selection gradients on age-specific and stage-specific mortality and fertility.

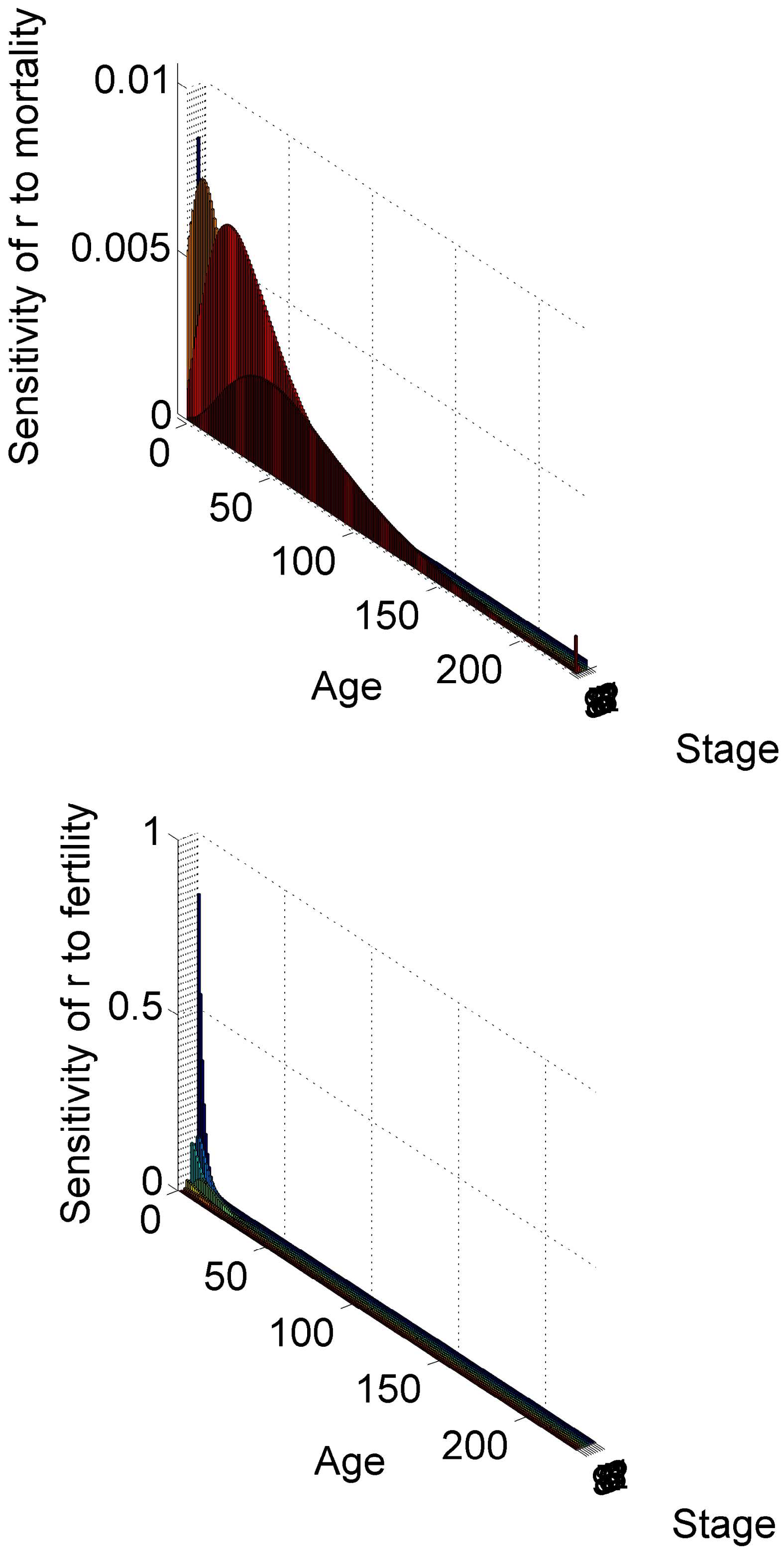

Figure 56: Selection gradients on age×stage-specific mortality and fertility.

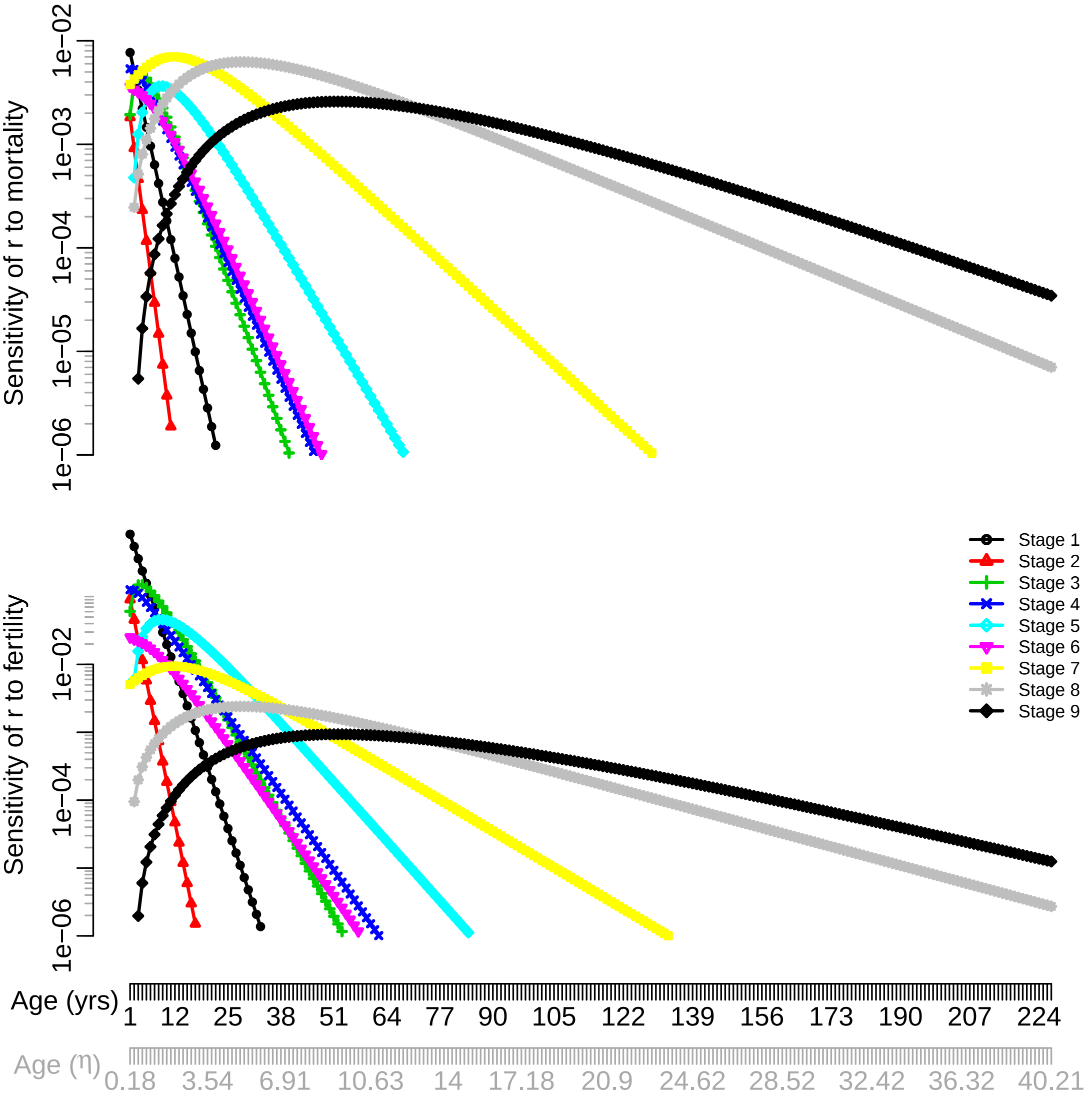

Figure 57: Selection gradients on age×stage-specific mortality and fertility. Top x axis represents age in years, whereas the bottom x axis represents time-scale normalized by the life expectancy (η) of the first non-seedbank stage (FNSS) of the species.

S1.20 *Opuntia rastrera*

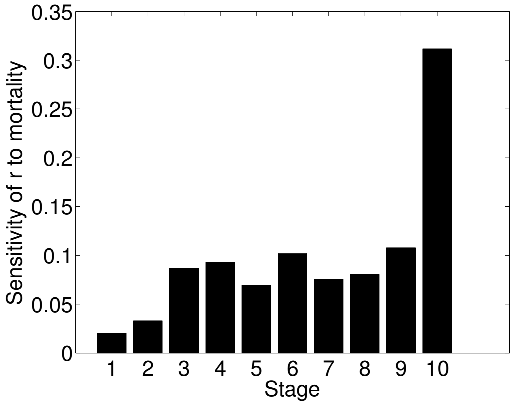

(a) Stage-specific mortality

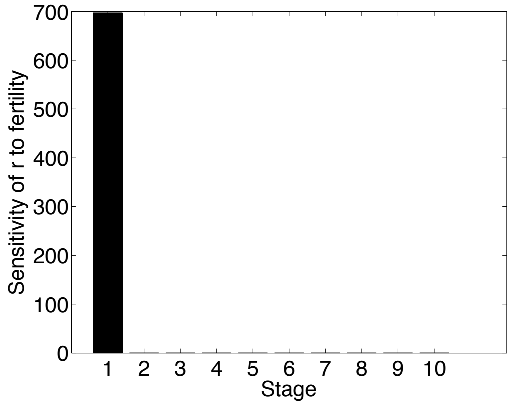

(b) Stage-specific fertility

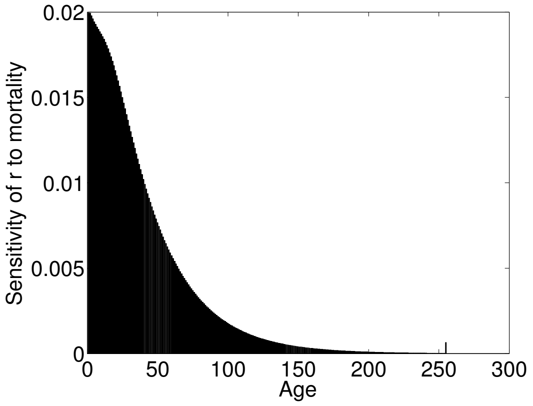

(c) Age-specific mortality

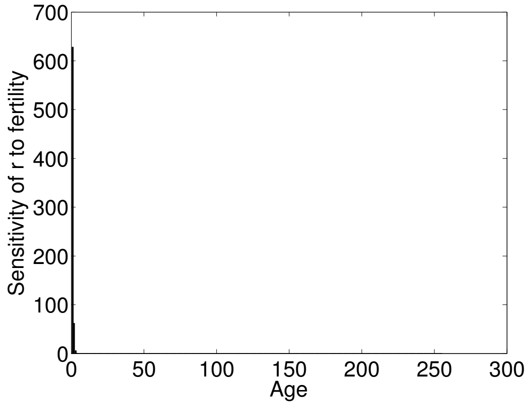

(d) Age-specific fertility

Figure 58: Selection gradients on age-specific and stage-specific mortality and fertility.

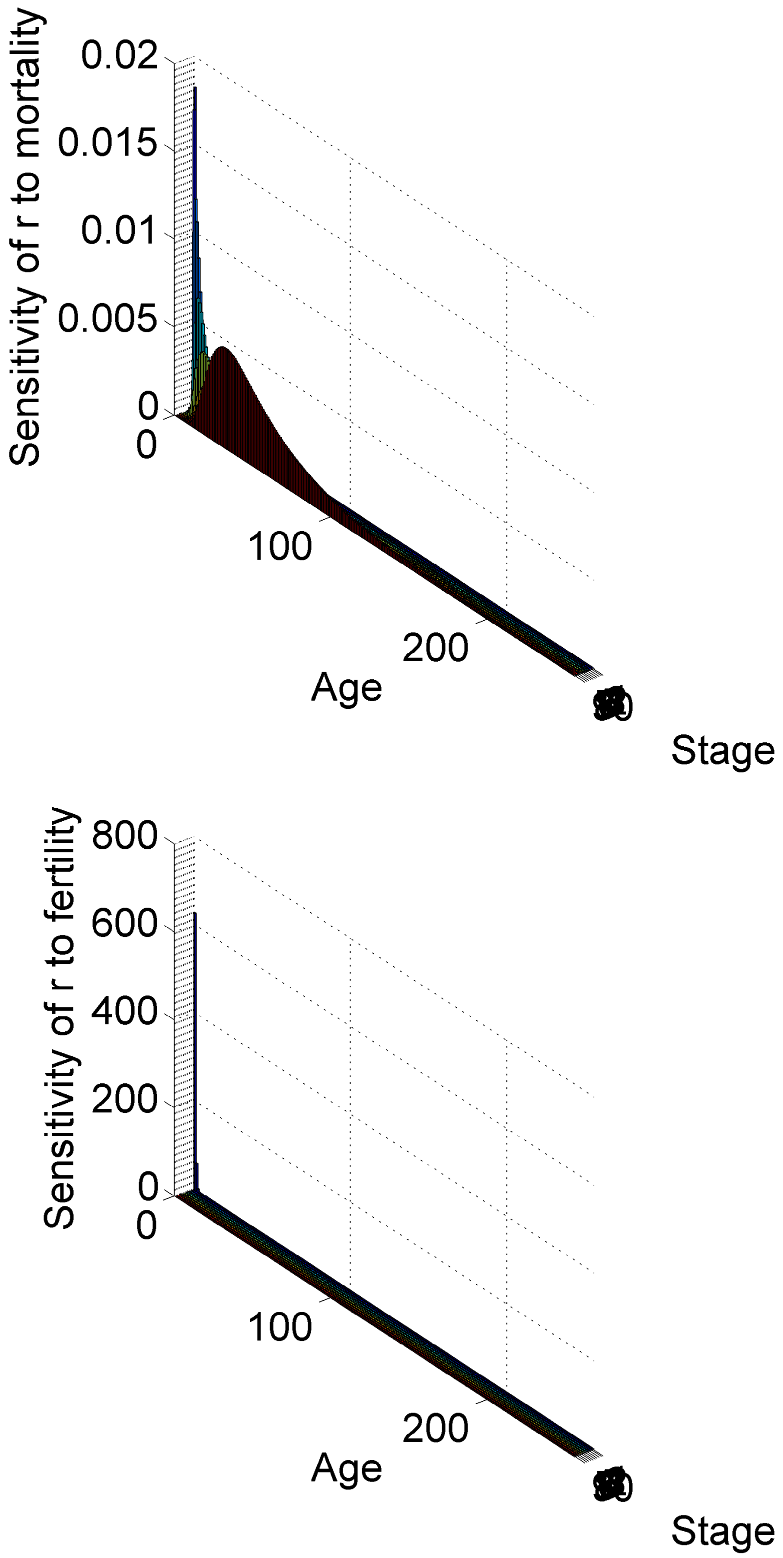

Figure 59: Selection gradients on age×stage-specific mortality and fertility.

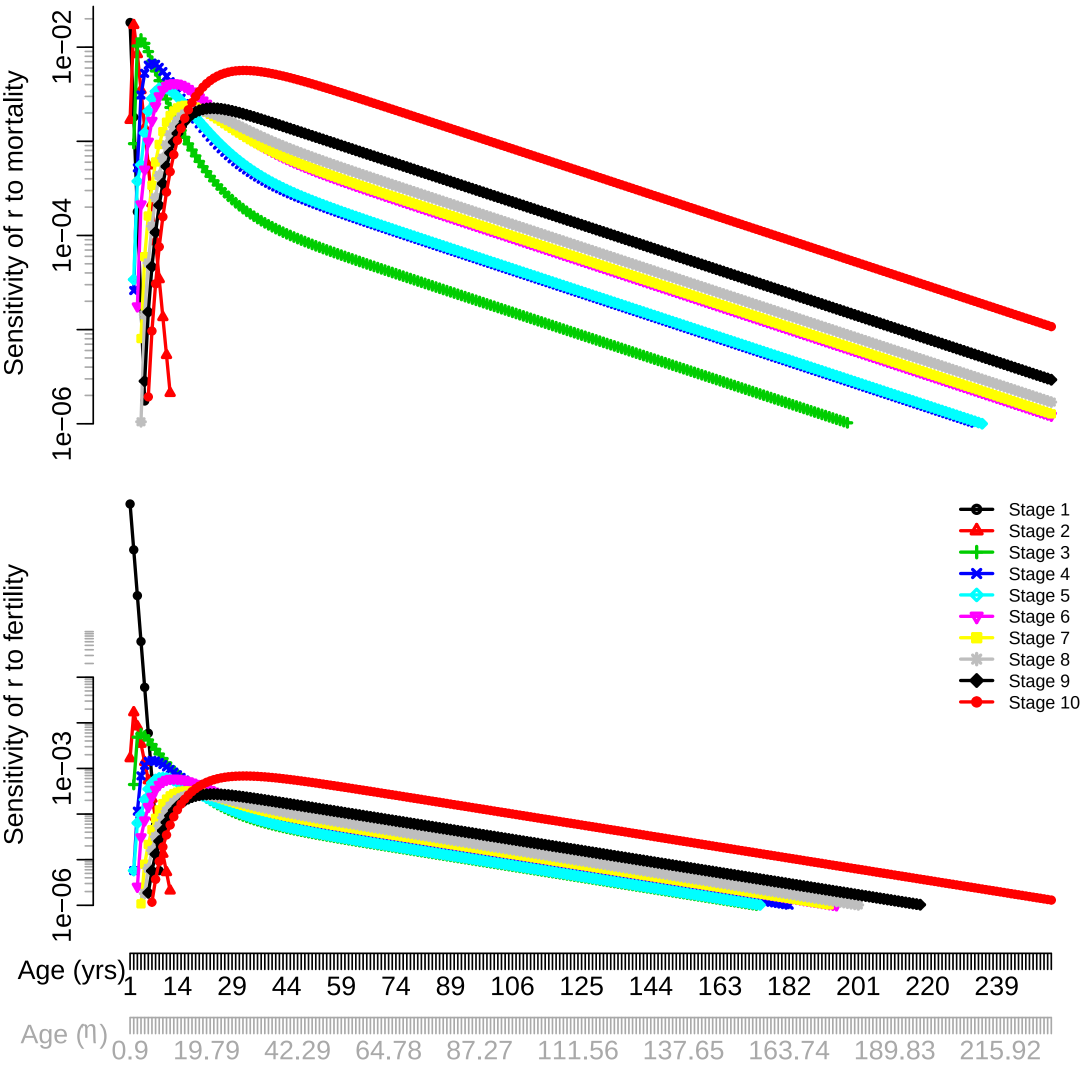

Figure 60: Selection gradients on age×stage-specific mortality and fertility. Top x axis represents age in years, whereas the bottom x axis represents time-scale normalized by the life expectancy (η) of the first non-seedbank stage (FNSS) of the species.

S1.21 *Panax quinquefolium*

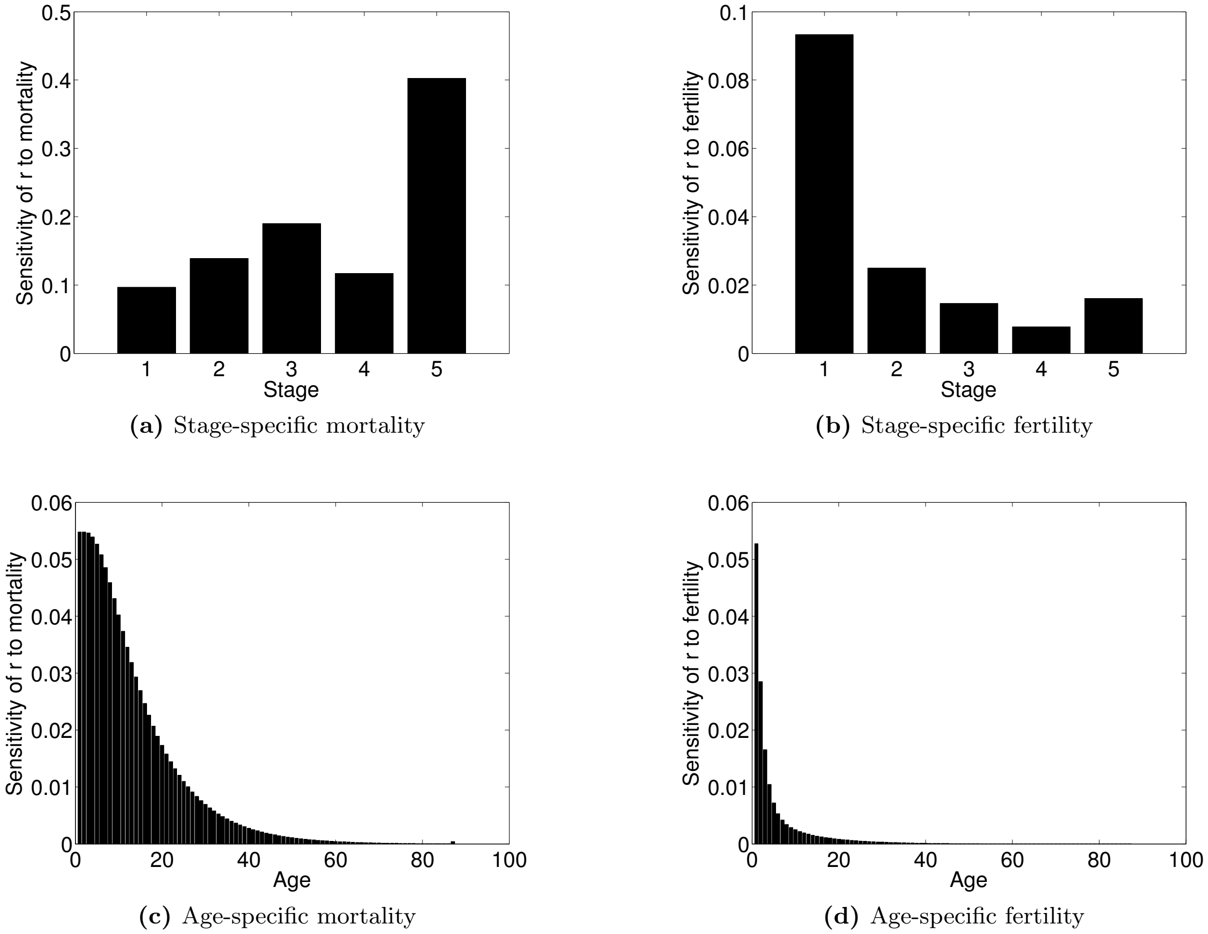

(a) Stage-specific mortality

(b) Stage-specific fertility

(c) Age-specific mortality

(d) Age-specific fertility

Figure 61: Selection gradients on age-specific and stage-specific mortality and fertility.

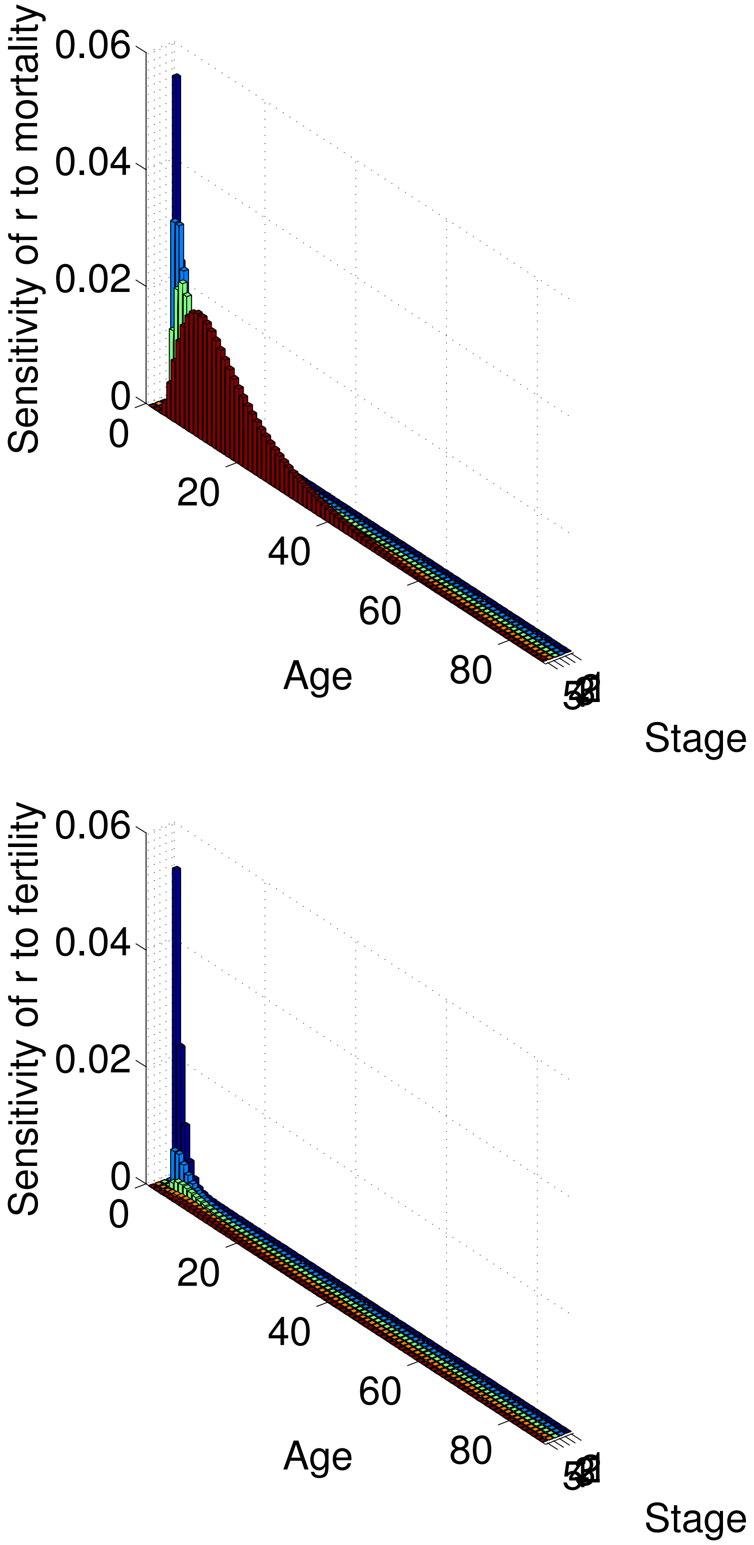

Figure 62: Selection gradients on age×stage-specific mortality and fertility.

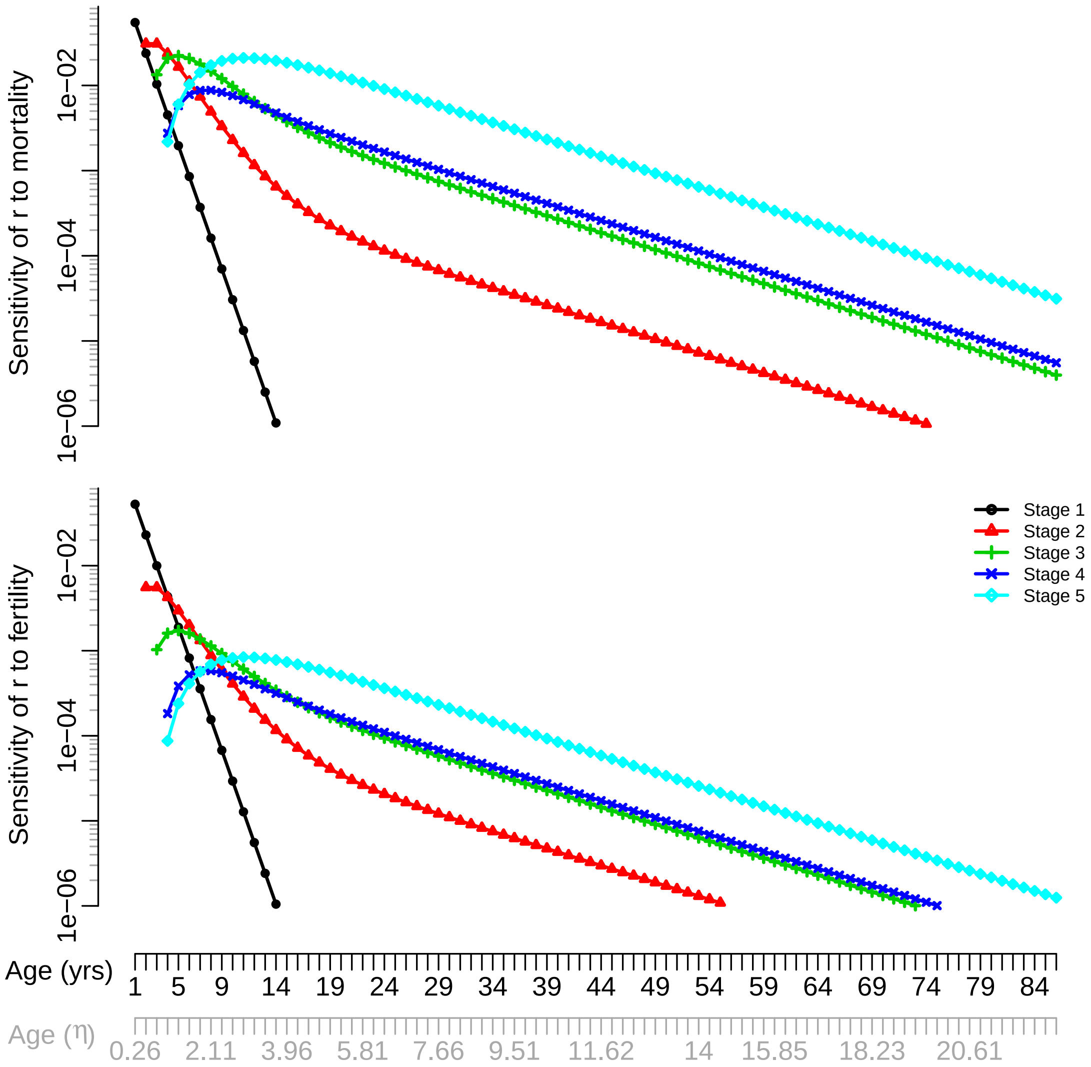

Figure 63: Selection gradients on age×stage-specific mortality and fertility. Top x axis represents age in years, whereas the bottom x axis represents time-scale normalized by the life expectancy (η) of the first non-seedbank stage (FNSS) of the species.

S1.22 *Parashorea chinensis*

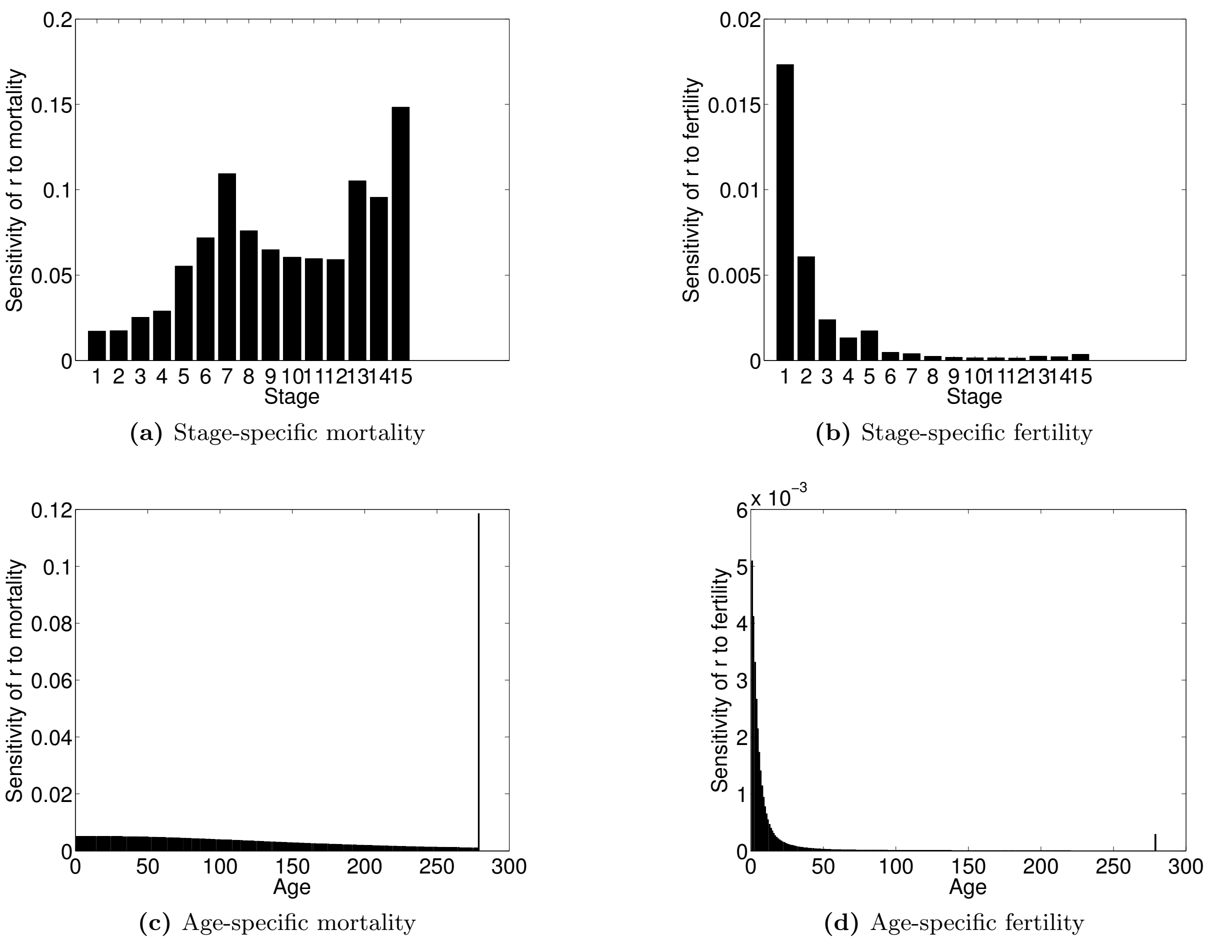

(a) Stage-specific mortality

(b) Stage-specific fertility

(c) Age-specific mortality

(d) Age-specific fertility

Figure 64: Selection gradients on age-specific and stage-specific mortality and fertility.

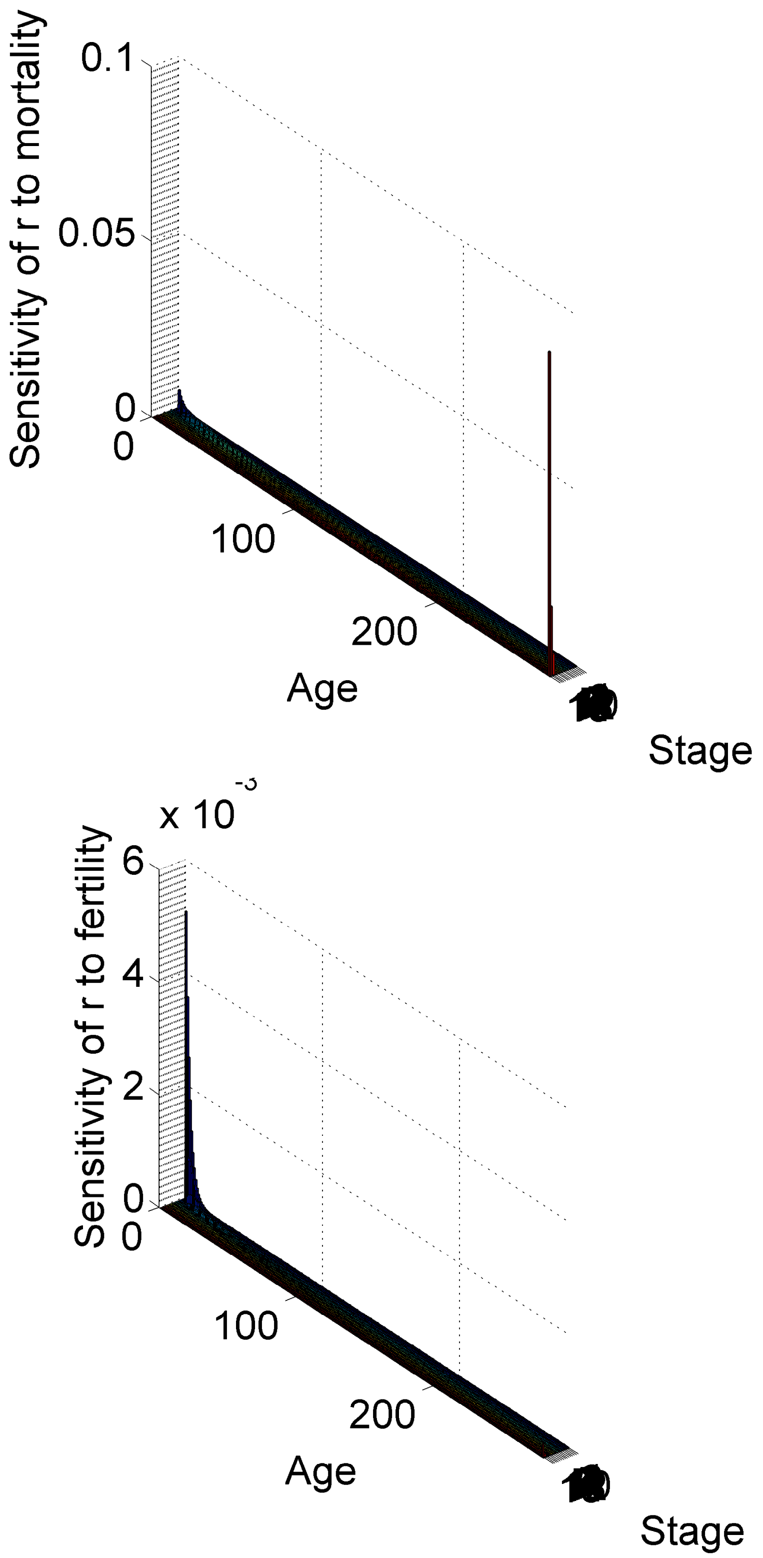

Figure 65: Selection gradients on age×stage-specific mortality and fertility.

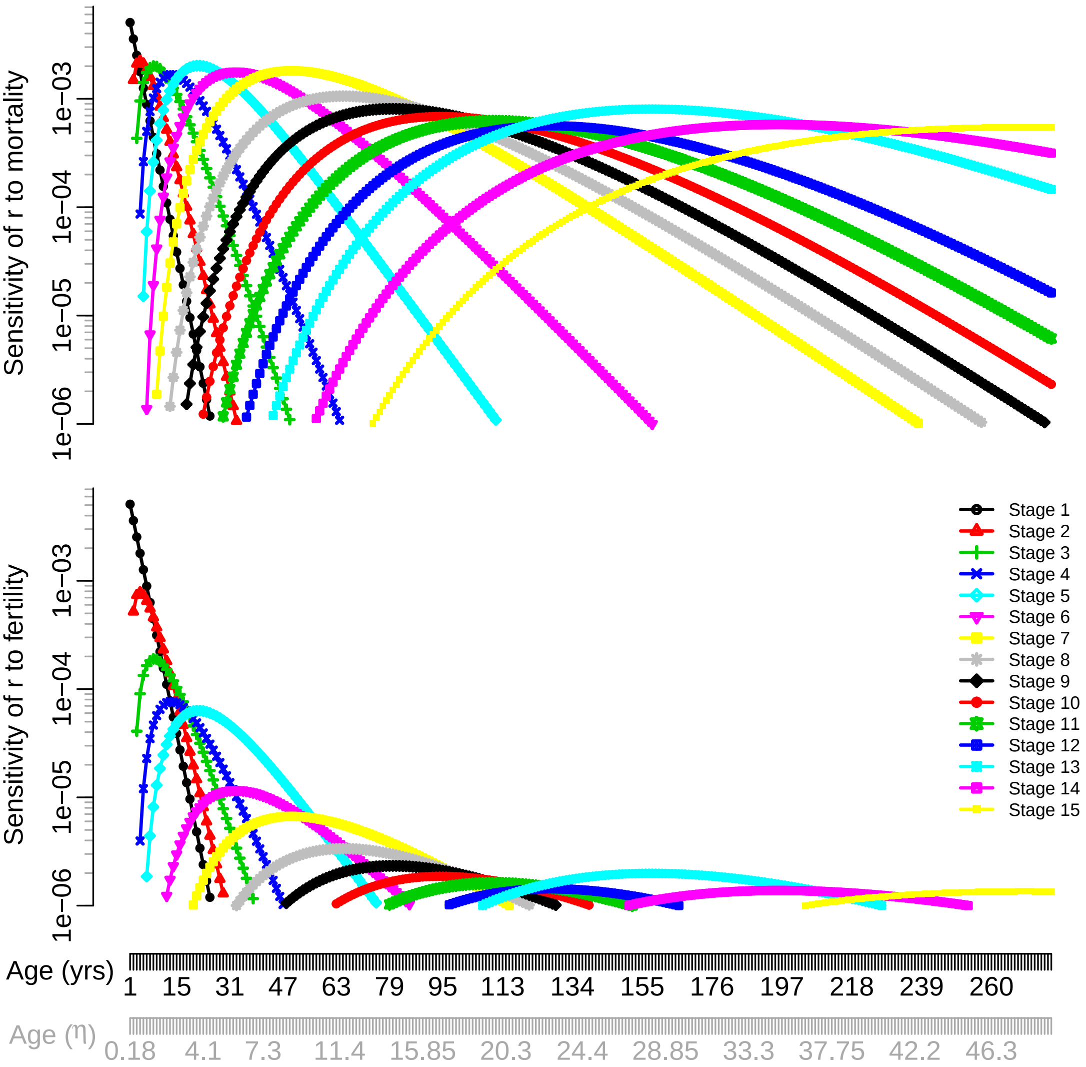

Figure 66: Selection gradients on age×stage-specific mortality and fertility. Top x axis represents age in years, whereas the bottom x axis represents time-scale normalized by the life expectancy (η) of the first non-seedbank stage (FNSS) of the species.

S1.23 *Periandra mediterranea*

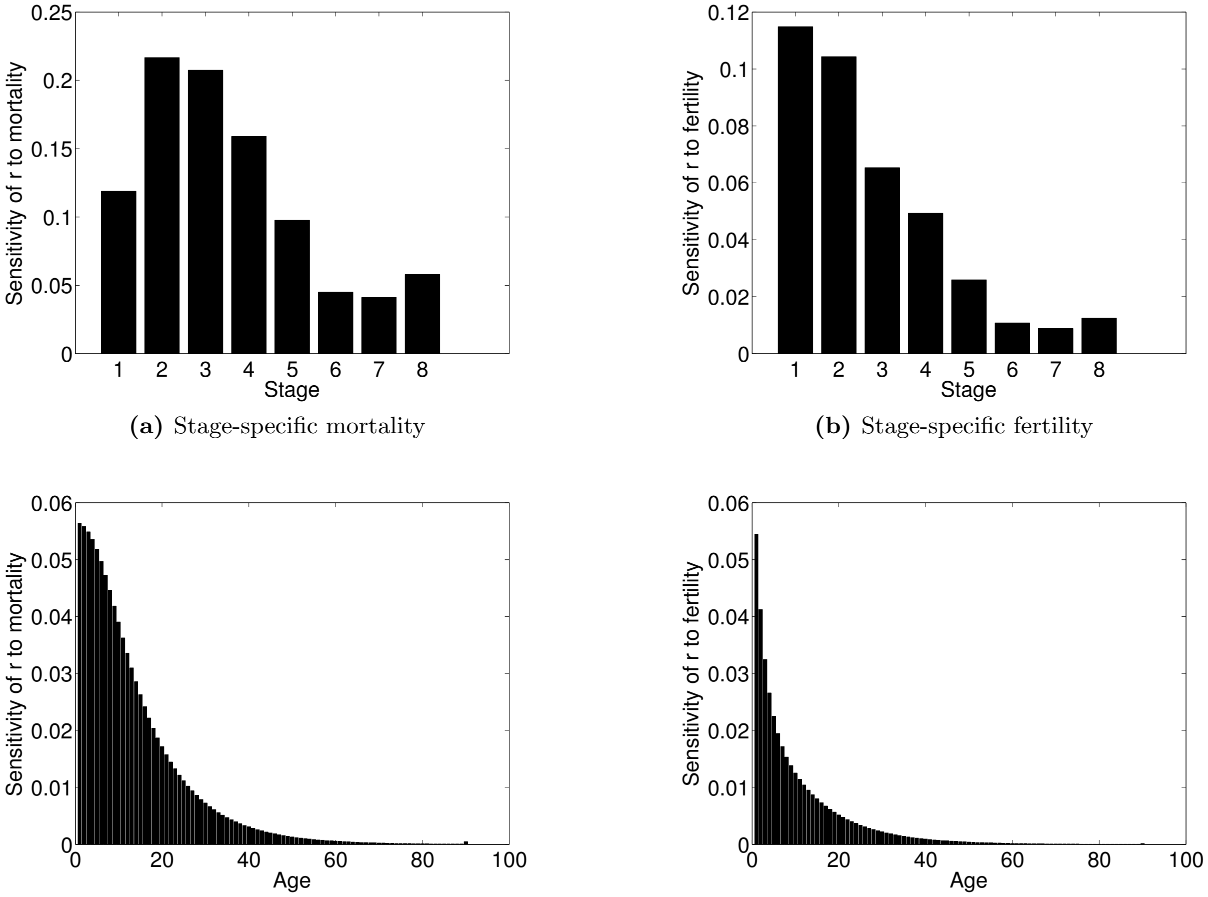

(a) Stage-specific mortality

(b) Stage-specific fertility

(c) Age-specific mortality

(d) Age-specific fertility

Figure 67: Selection gradients on age-specific and stage-specific mortality and fertility.

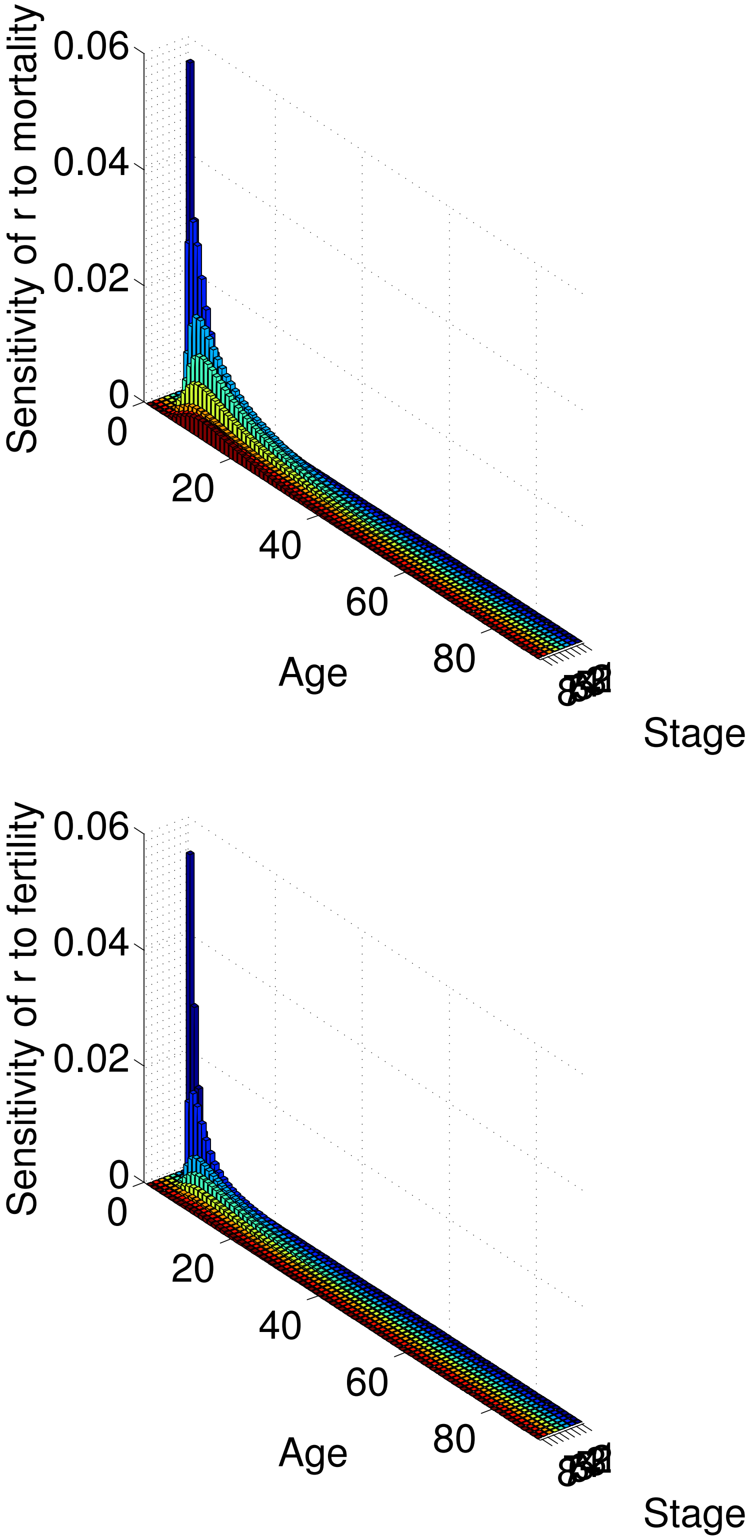

Figure 68: Selection gradients on age×stage-specific mortality and fertility.

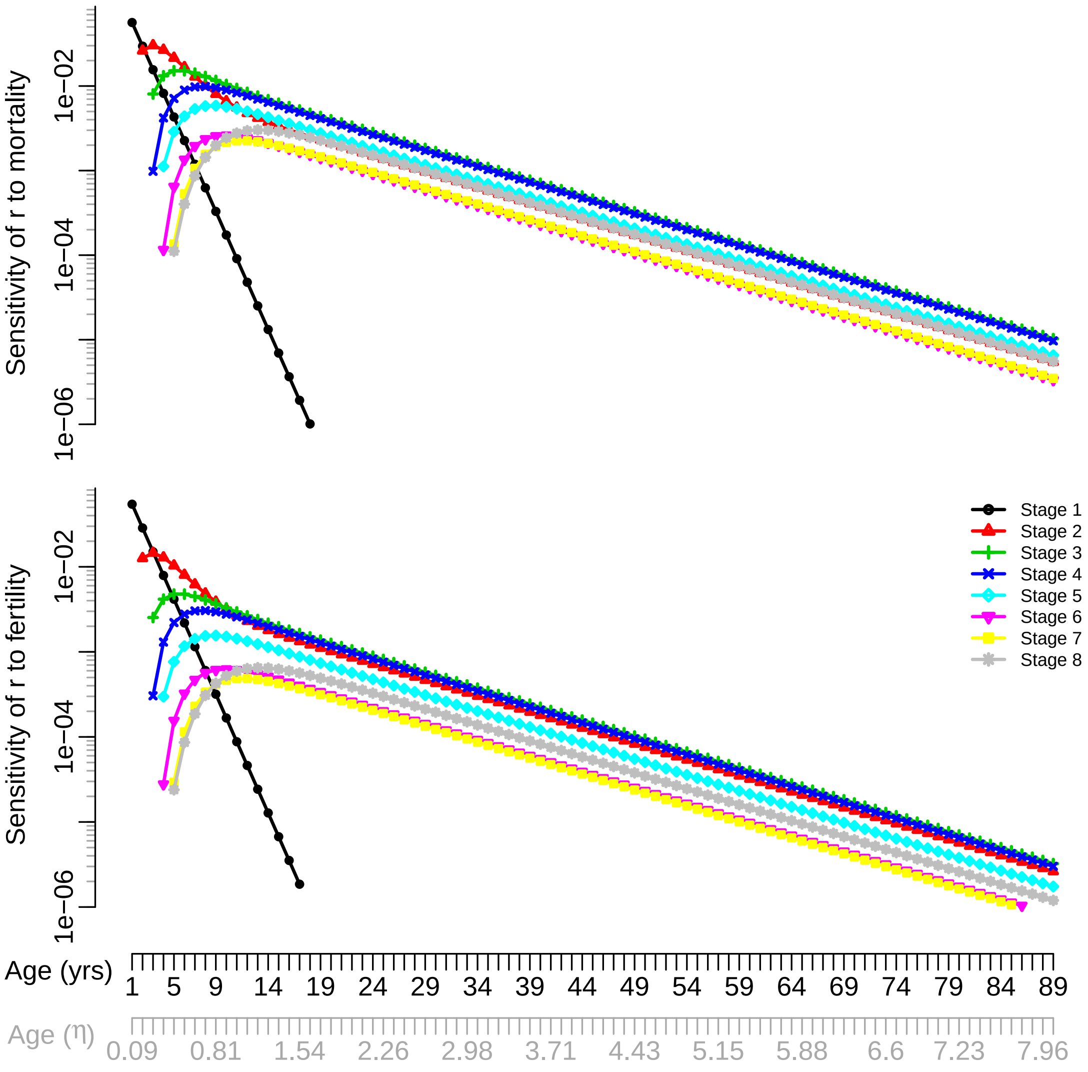

Figure 69: Selection gradients on age×stage-specific mortality and fertility. Top x axis represents age in years, whereas the bottom x axis represents time-scale normalized by the life expectancy (η) of the first non-seedbank stage (FNSS) of the species.

S1.24 *Pinguicula alpina*

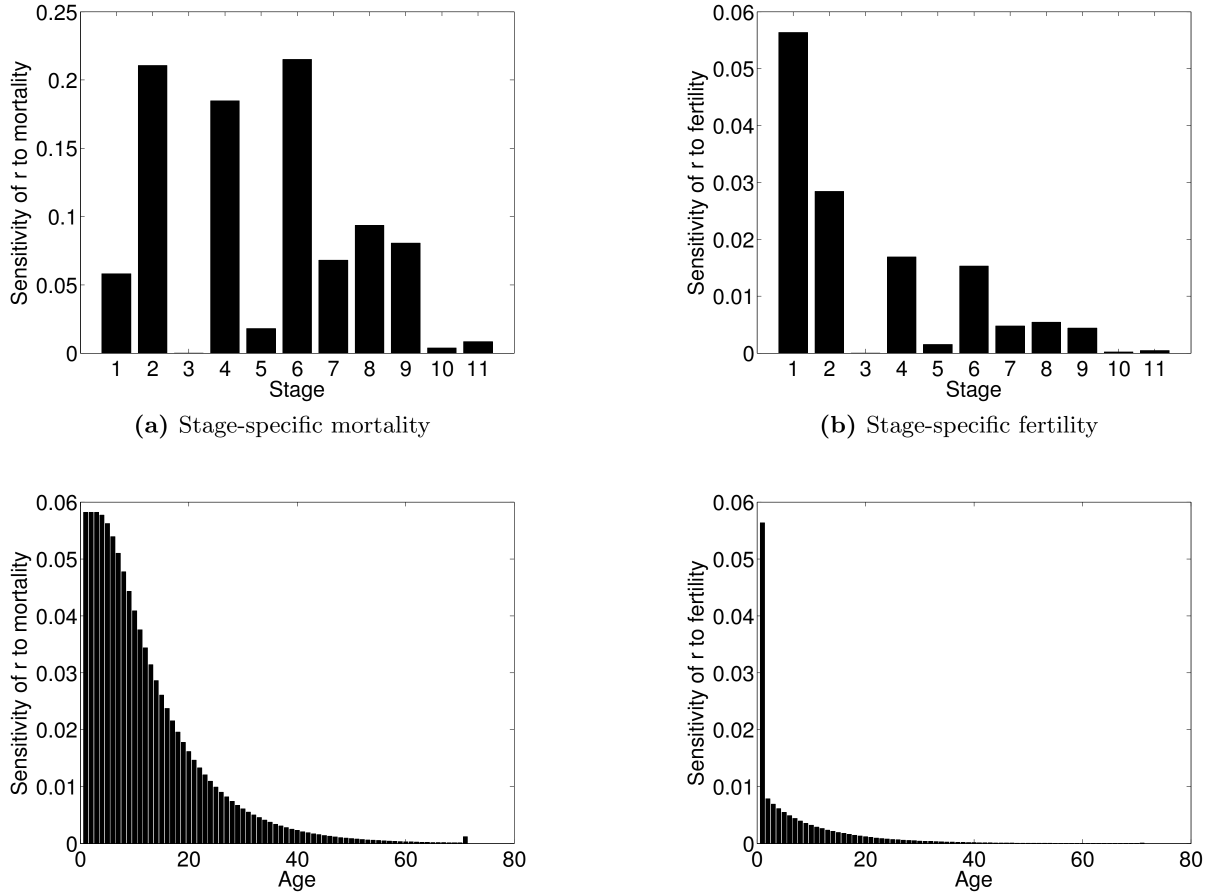

(a) Stage-specific mortality

(b) Stage-specific fertility

(c) Age-specific mortality

(d) Age-specific fertility

Figure 70: Selection gradients on age-specific and stage-specific mortality and fertility.

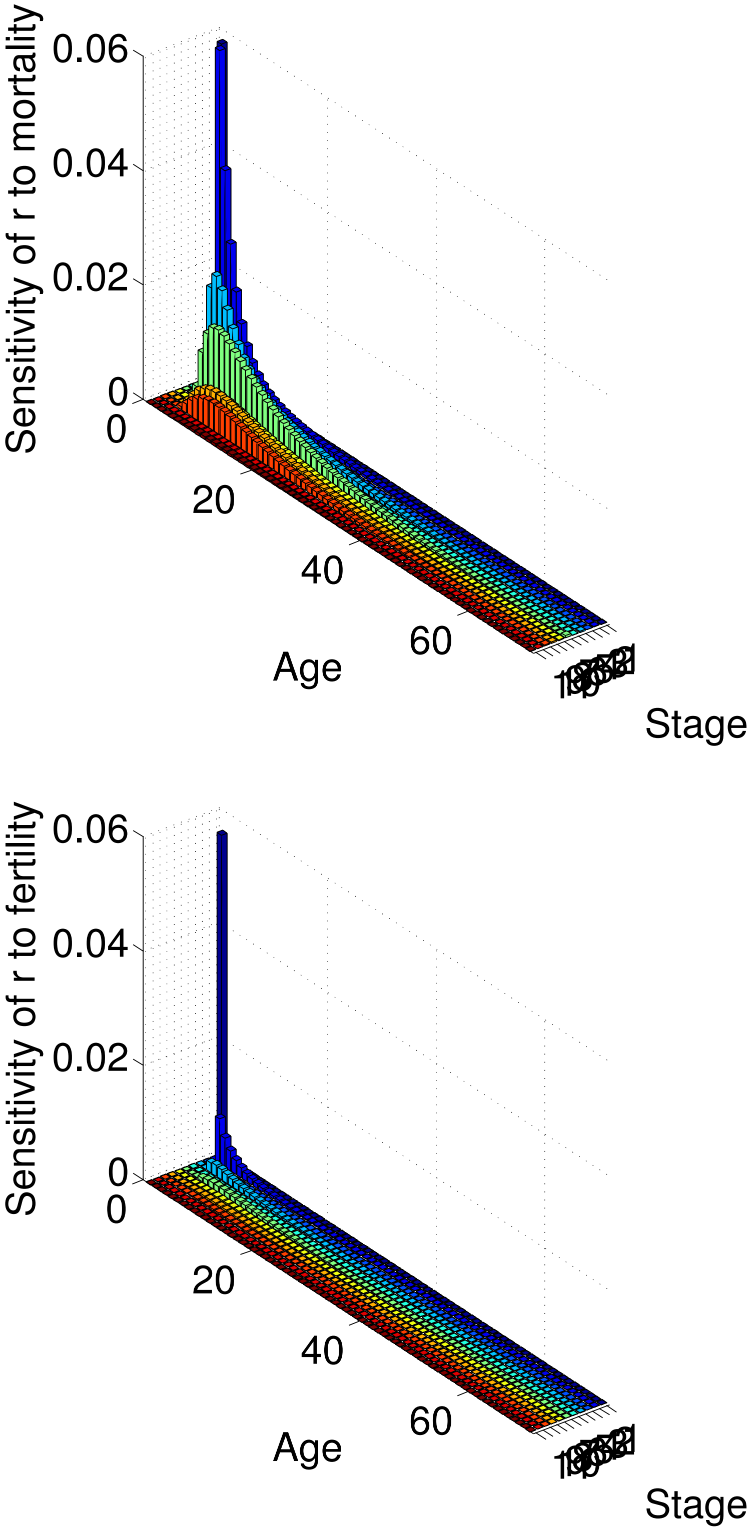

Figure 71: Selection gradients on age×stage-specific mortality and fertility.

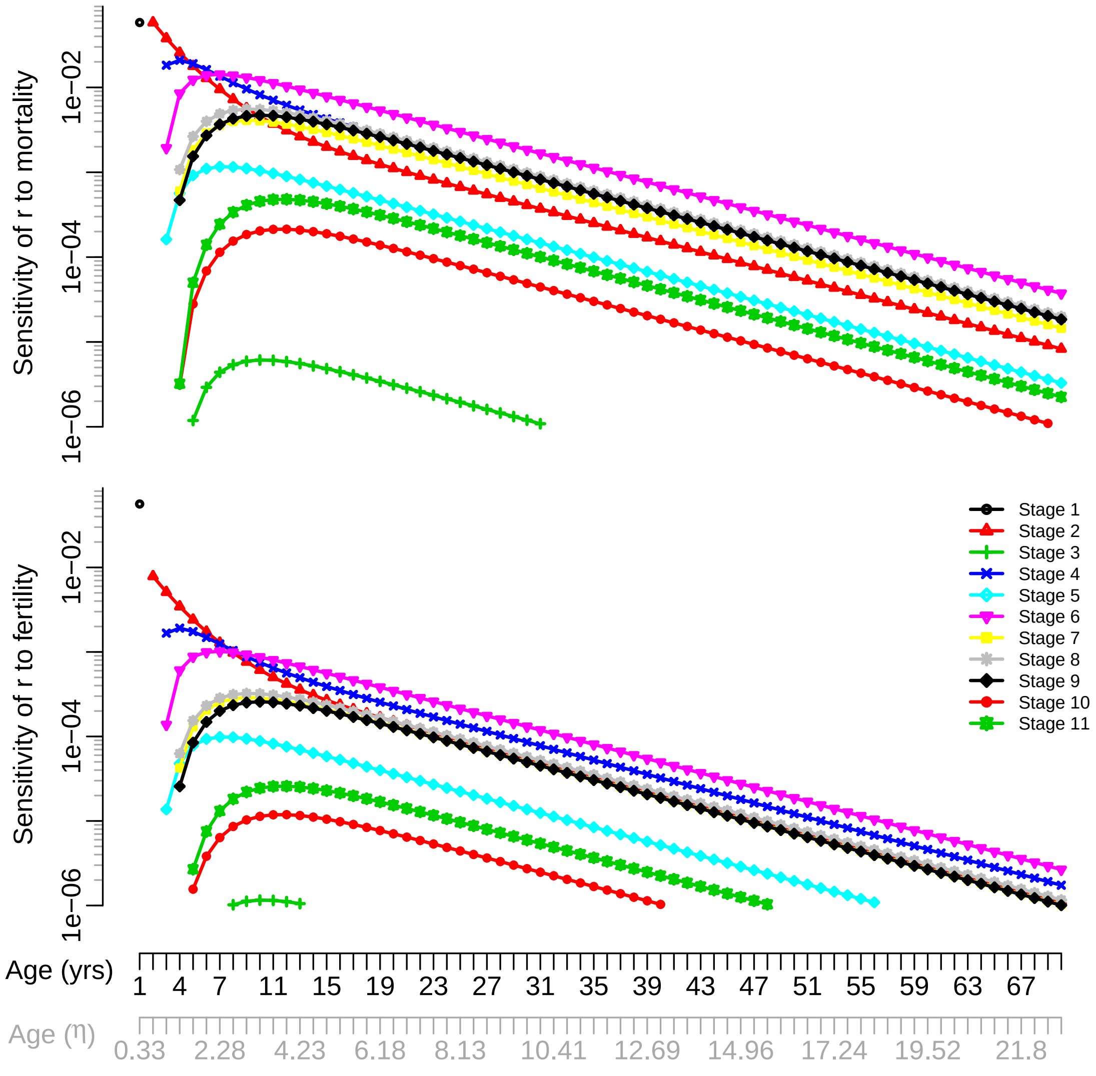

Figure 72: Selection gradients on age×stage-specific mortality and fertility. Top x axis represents age in years, whereas the bottom x axis represents time-scale normalized by the life expectancy (η) of the first non-seedbank stage (FNSS) of the species.

S1.25 *Pinus lambertiana*

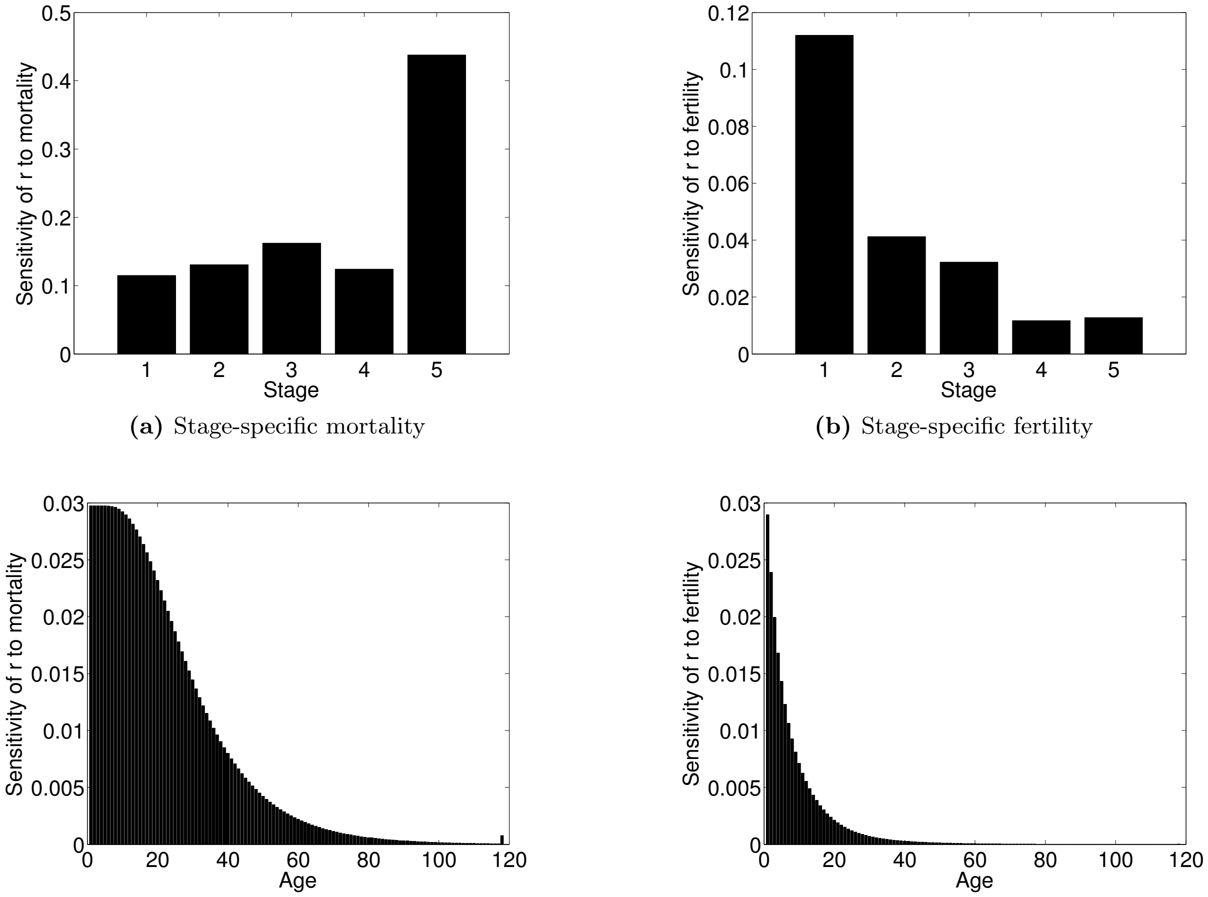

(a) Stage-specific mortality

(b) Stage-specific fertility

(c) Age-specific mortality

(d) Age-specific fertility

Figure 73: Selection gradients on age-specific and stage-specific mortality and fertility.

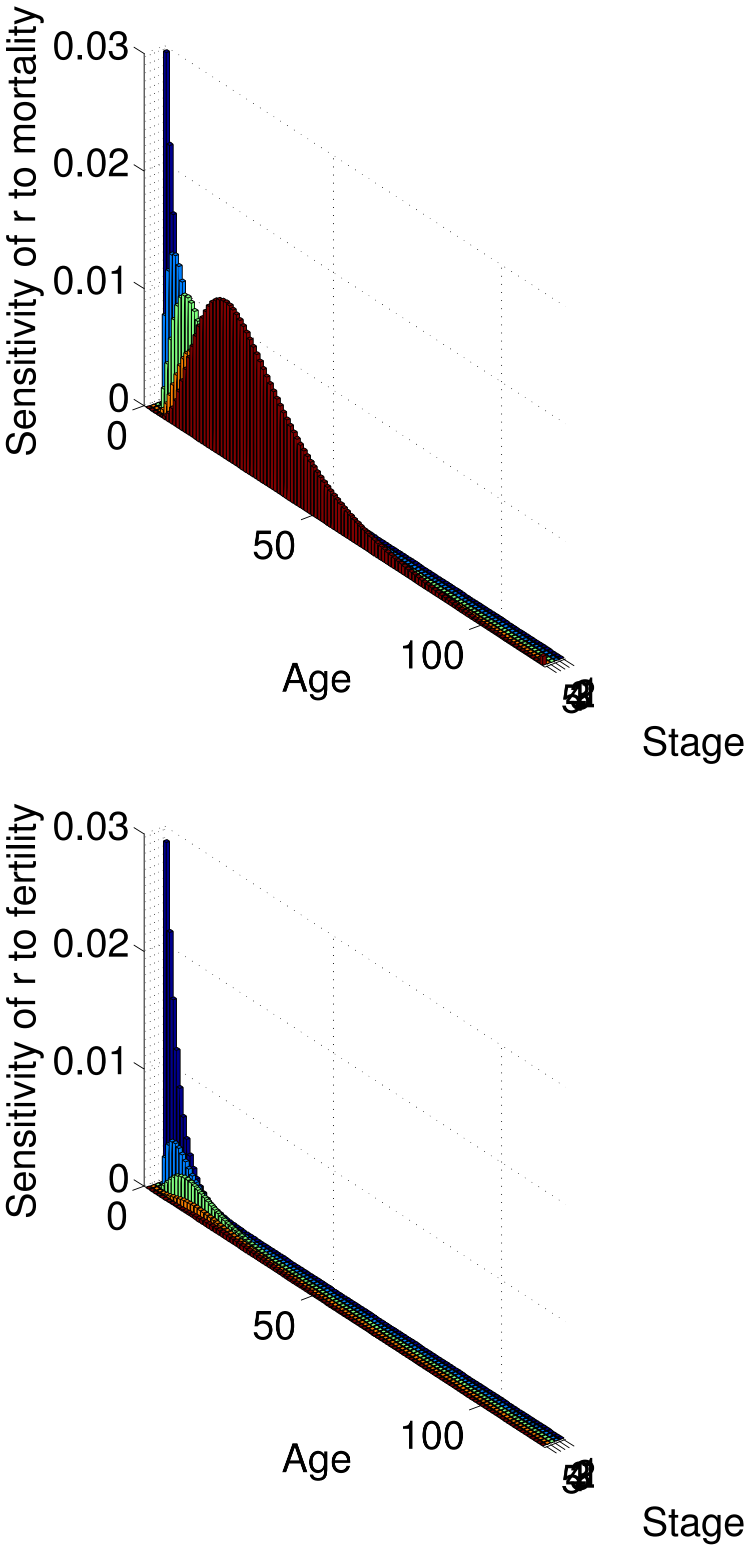

Figure 74: Selection gradients on age×stage-specific mortality and fertility.

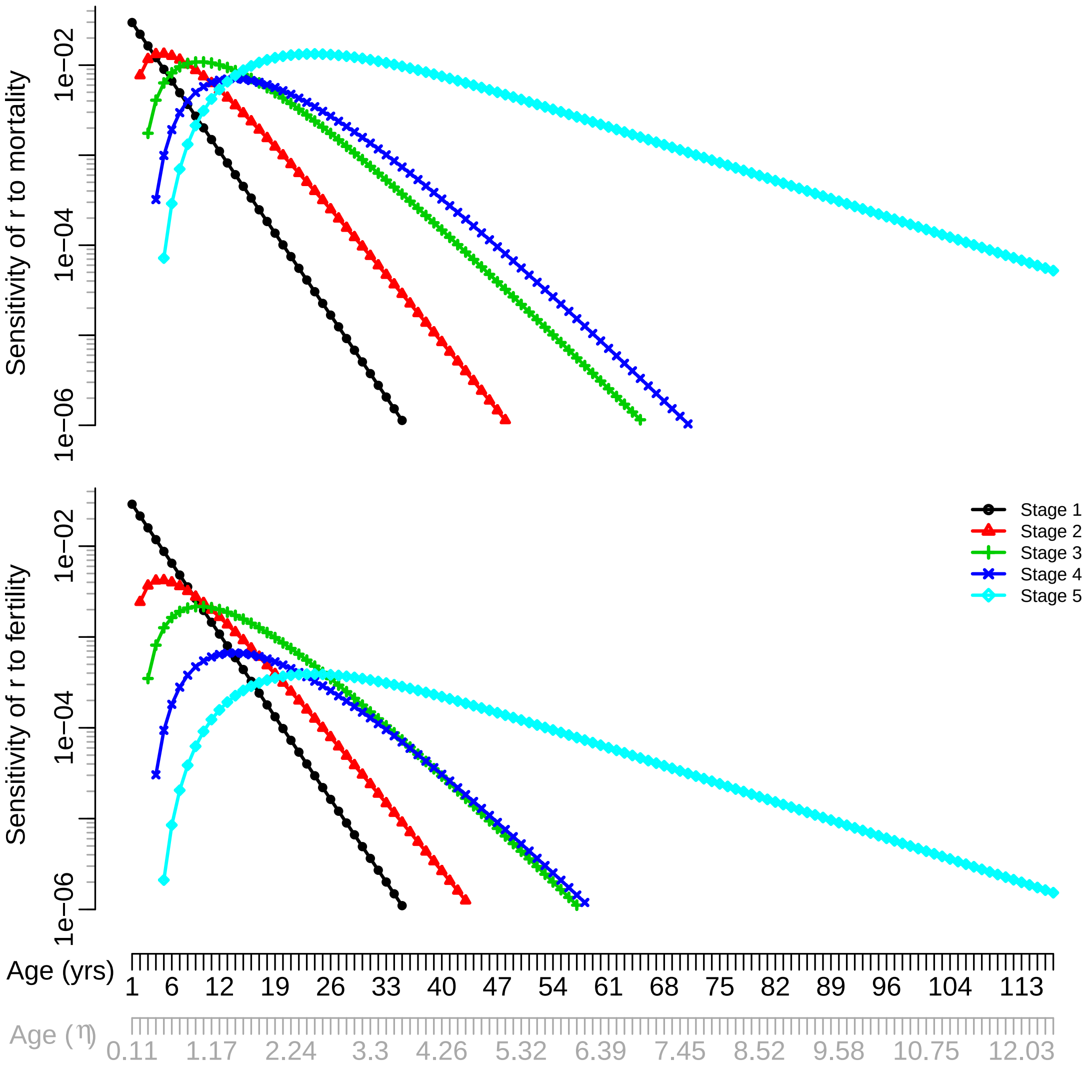

Figure 75: Selection gradients on age×stage-specific mortality and fertility. Top x axis represents age in years, whereas the bottom x axis represents time-scale normalized by the life expectancy (η) of the first non-seedbank stage (FNSS) of the species.

S1.26 *Pinus ponderosa*

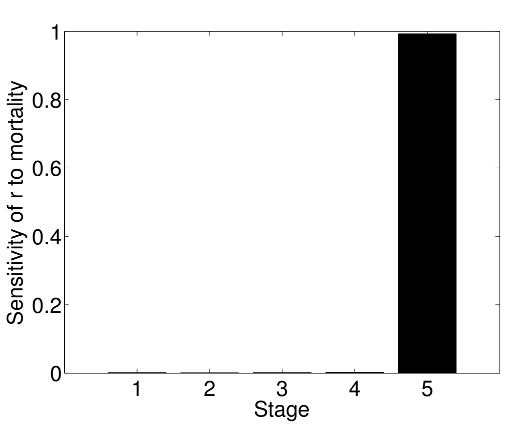

(a) Stage-specific mortality

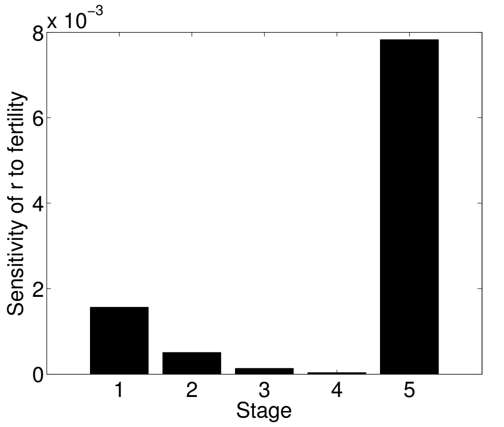

(b) Stage-specific fertility

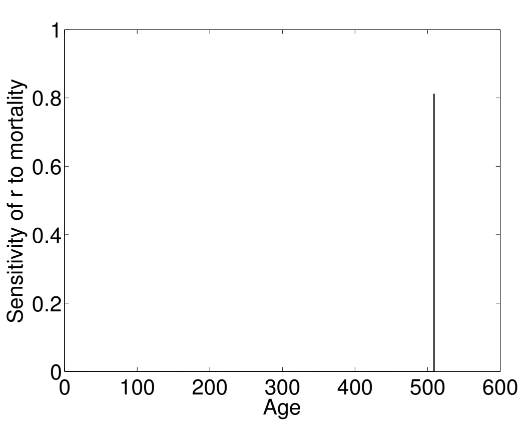

(c) Age-specific mortality

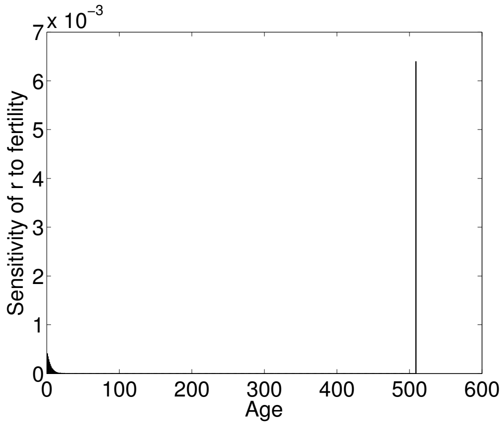

(d) Age-specific fertility

Figure 76: Selection gradients on age-specific and stage-specific mortality and fertility.

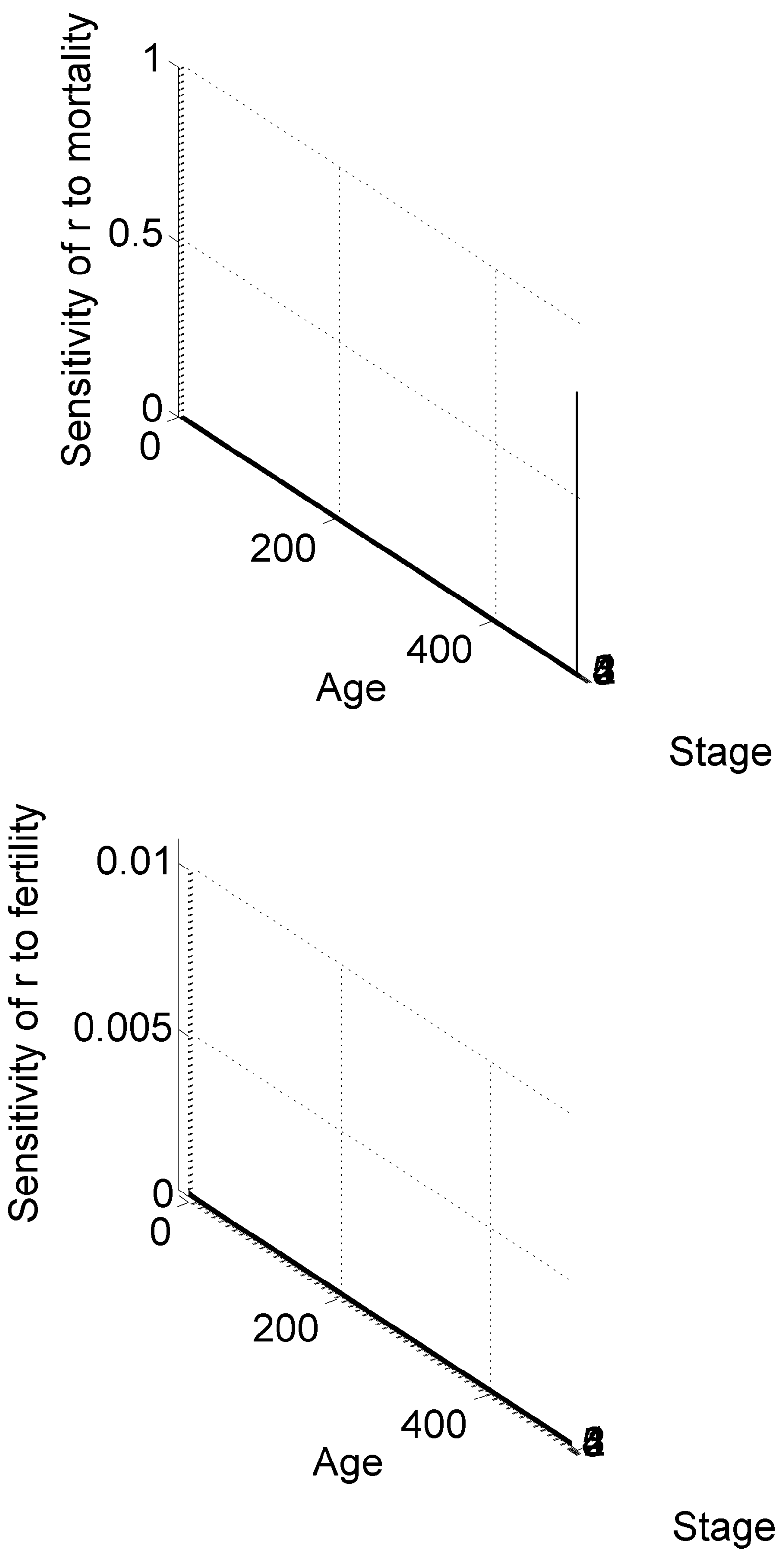

Figure 77: Selection gradients on age×stage-specific mortality and fertility.

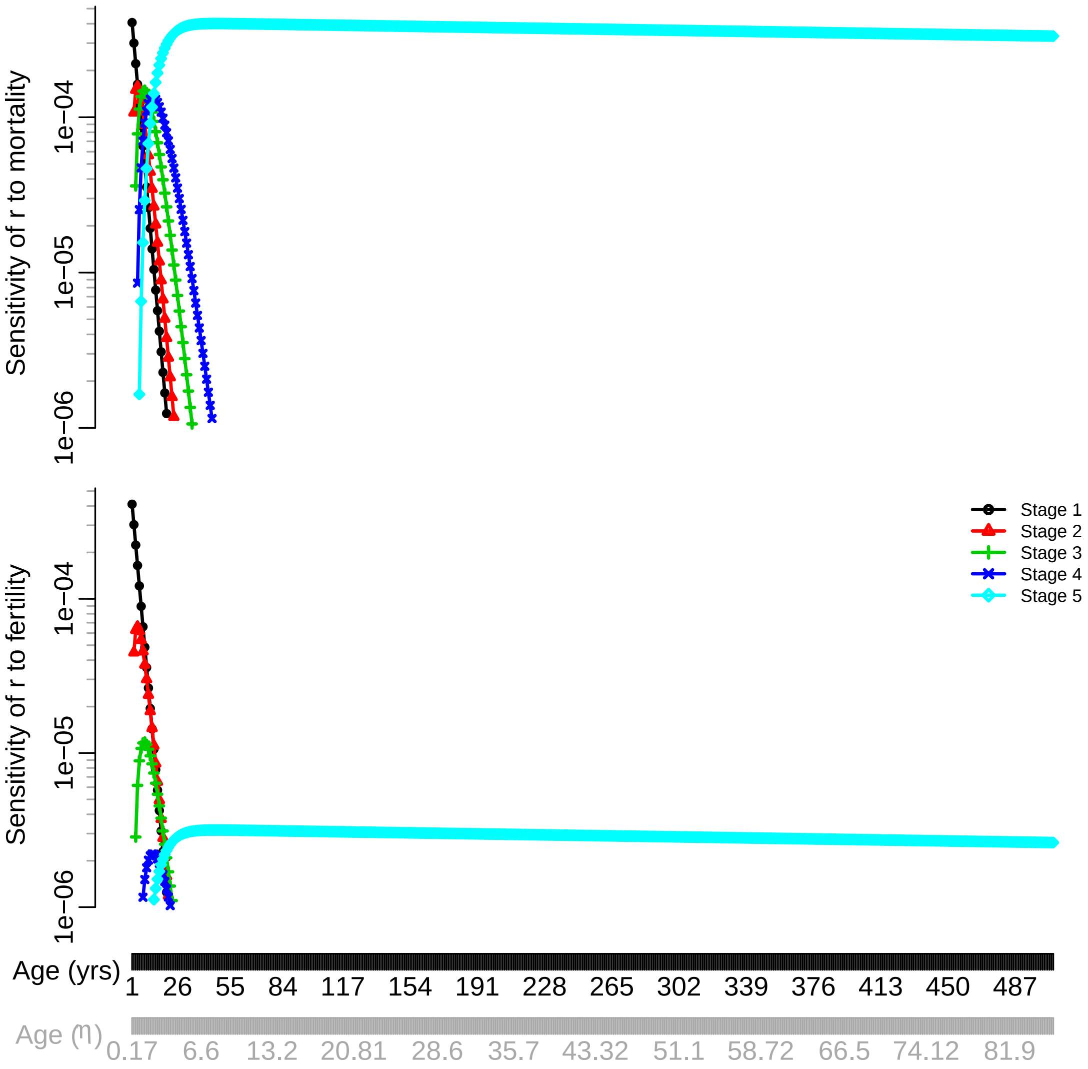

Figure 78: Selection gradients on age×stage-specific mortality and fertility. Top x axis represents age in years, whereas the bottom x axis represents time-scale normalized by the life expectancy (η) of the first non-seedbank stage (FNSS) of the species.

S1.27 *Polystichum aculeatum*

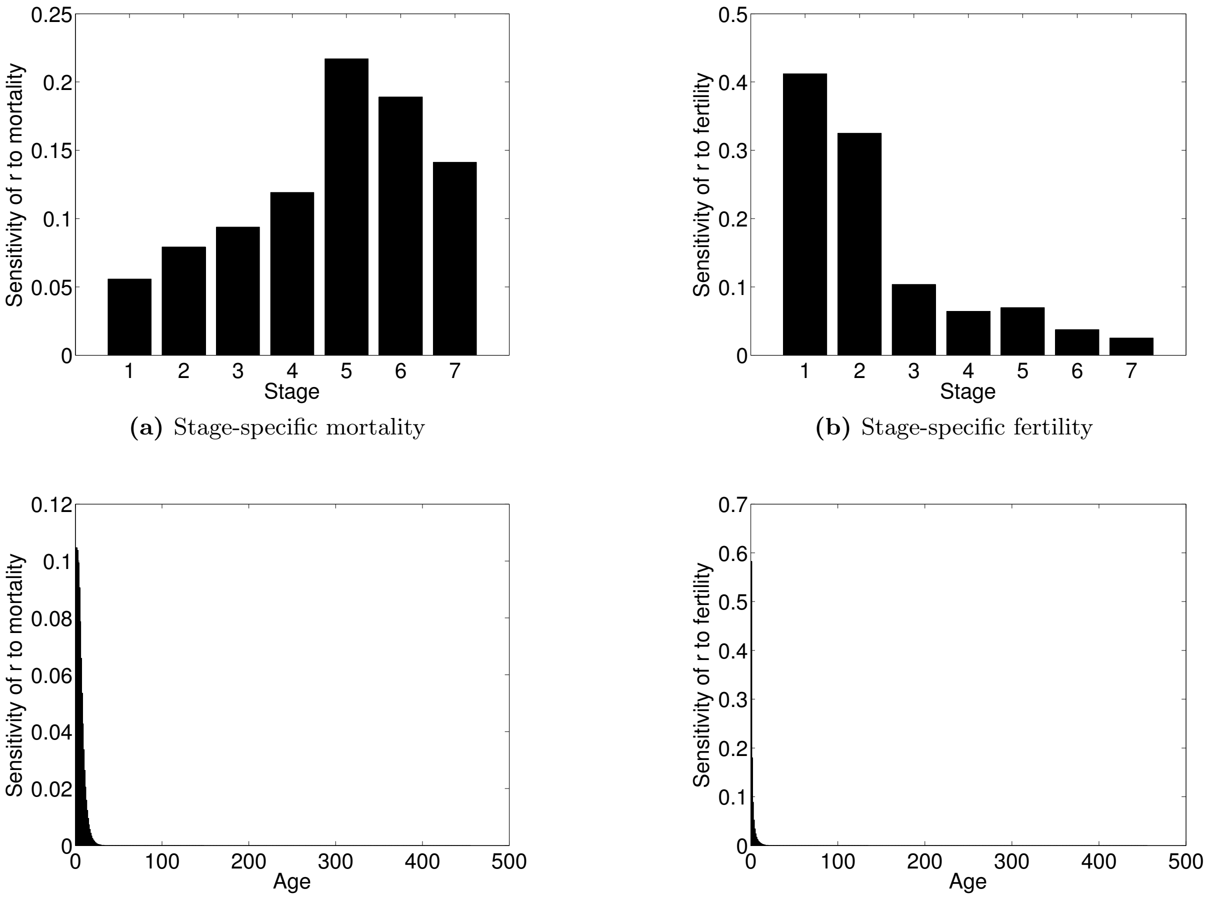

(a) Stage-specific mortality

(b) Stage-specific fertility

(c) Age-specific mortality

(d) Age-specific fertility

Figure 79: Selection gradients on age-specific and stage-specific mortality and fertility.

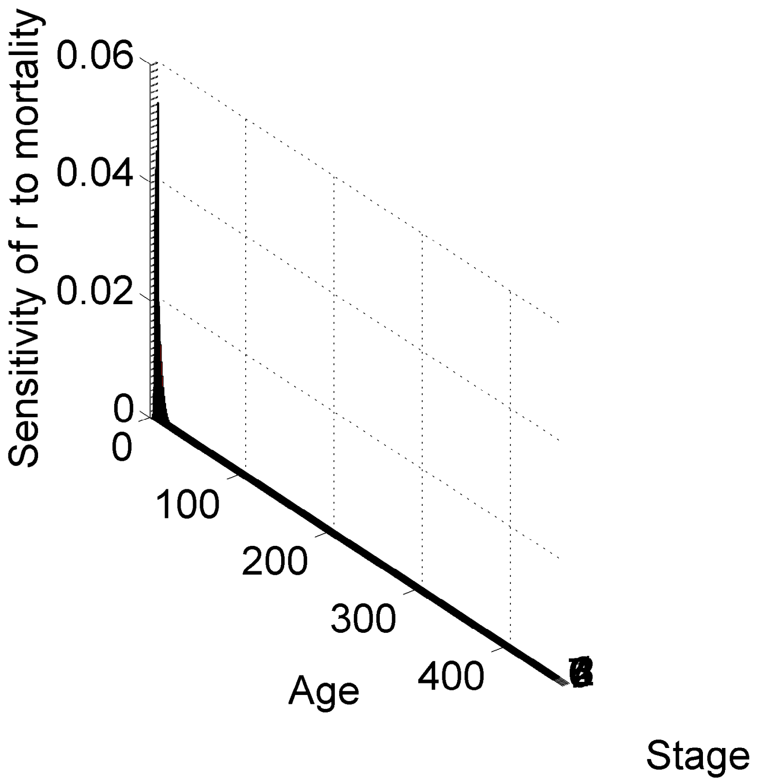

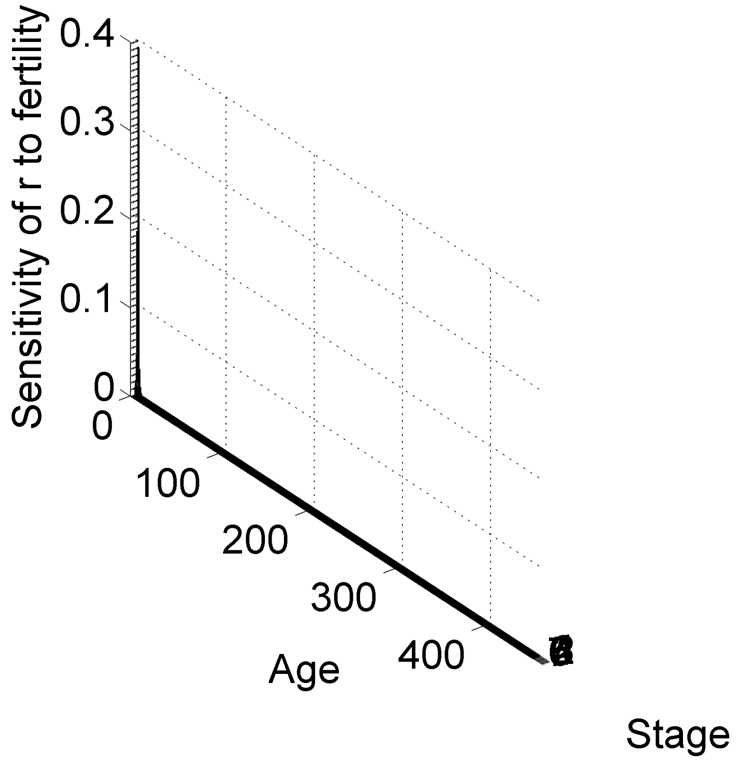

Figure 80: Selection gradients on age×stage-specific mortality and fertility.

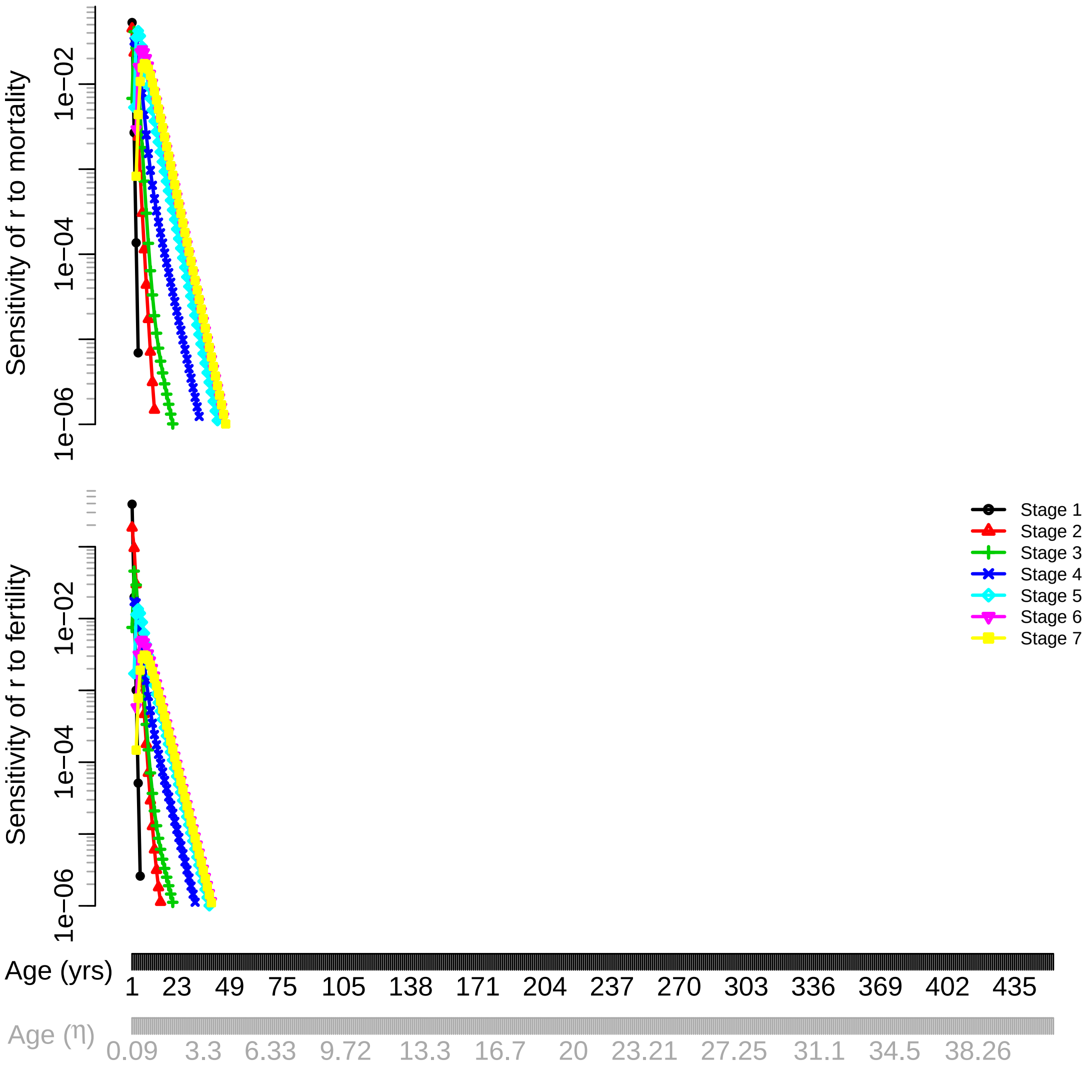

Figure 81: Selection gradients on age×stage-specific mortality and fertility. Top x axis represents age in years, whereas the bottom x axis represents time-scale normalized by the life expectancy (η) of the first non-seedbank stage (FNSS) of the species.

S1.28 *Primula vulgaris*

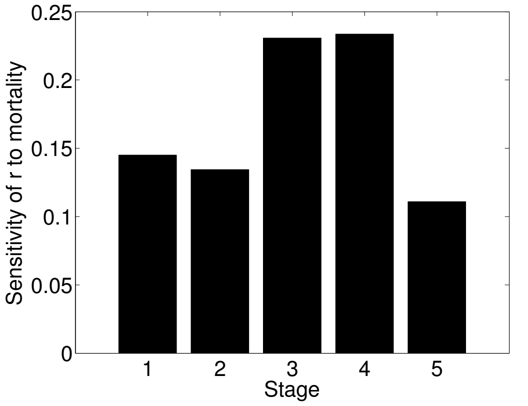

(a) Stage-specific mortality

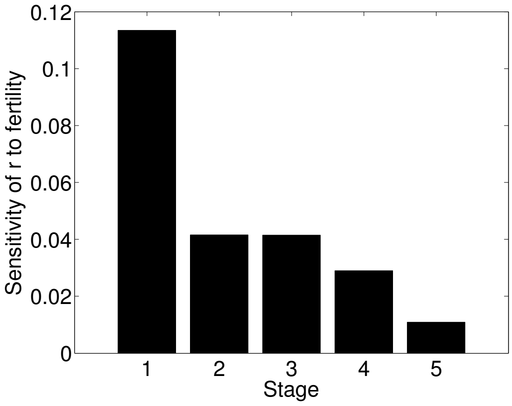

(b) Stage-specific fertility

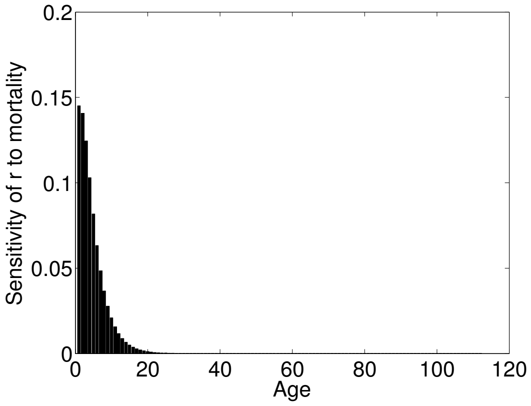

(c) Age-specific mortality

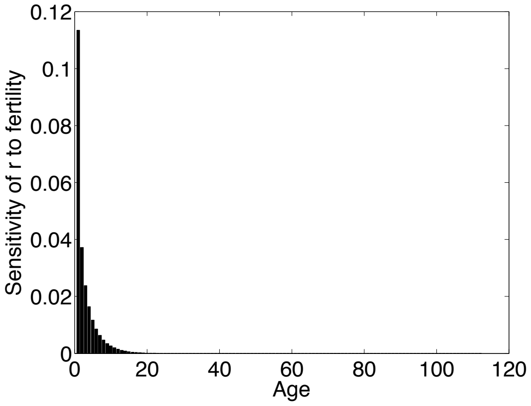

(d) Age-specific fertility

Figure 82: Selection gradients on age-specific and stage-specific mortality and fertility.

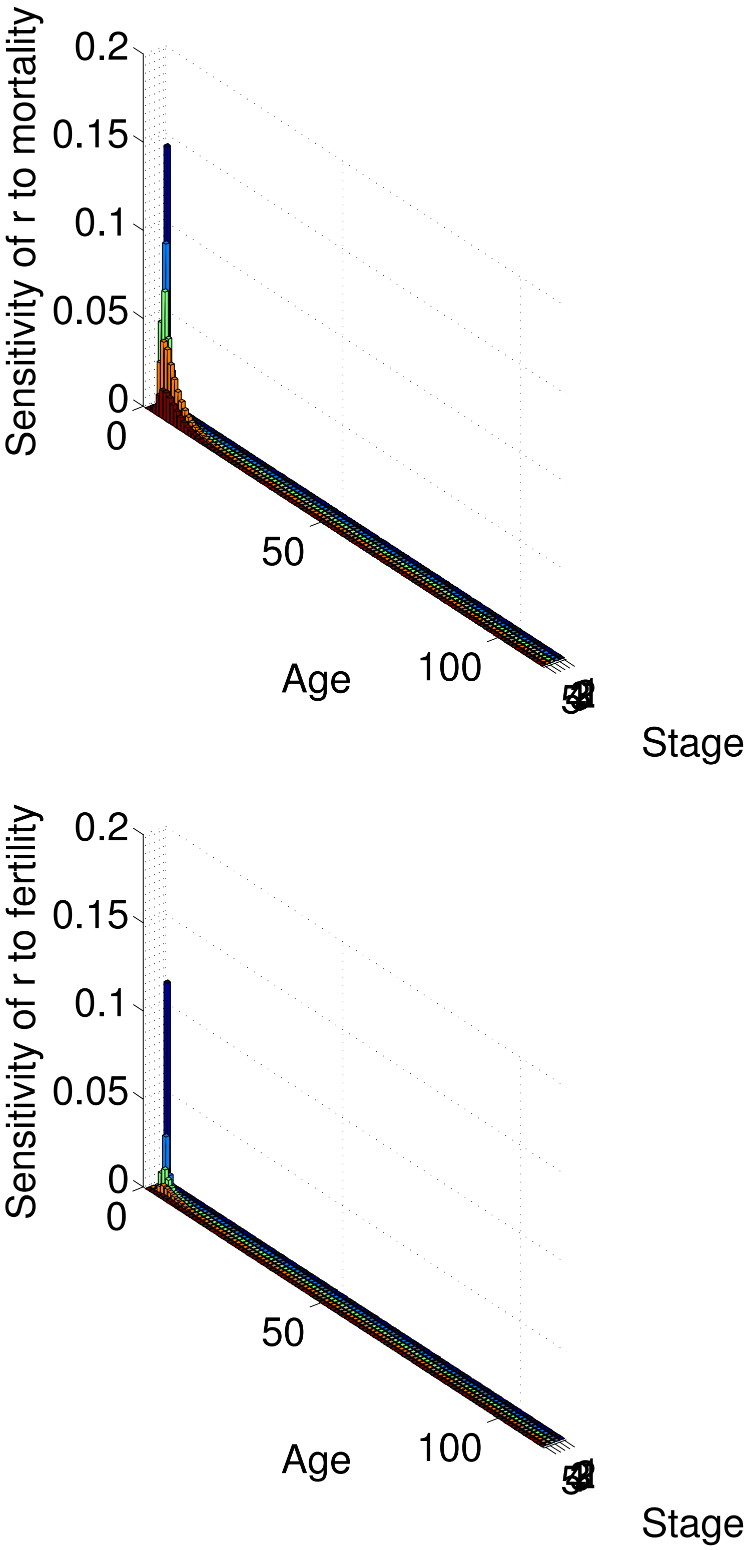

Figure 83: Selection gradients on age×stage-specific mortality and fertility.

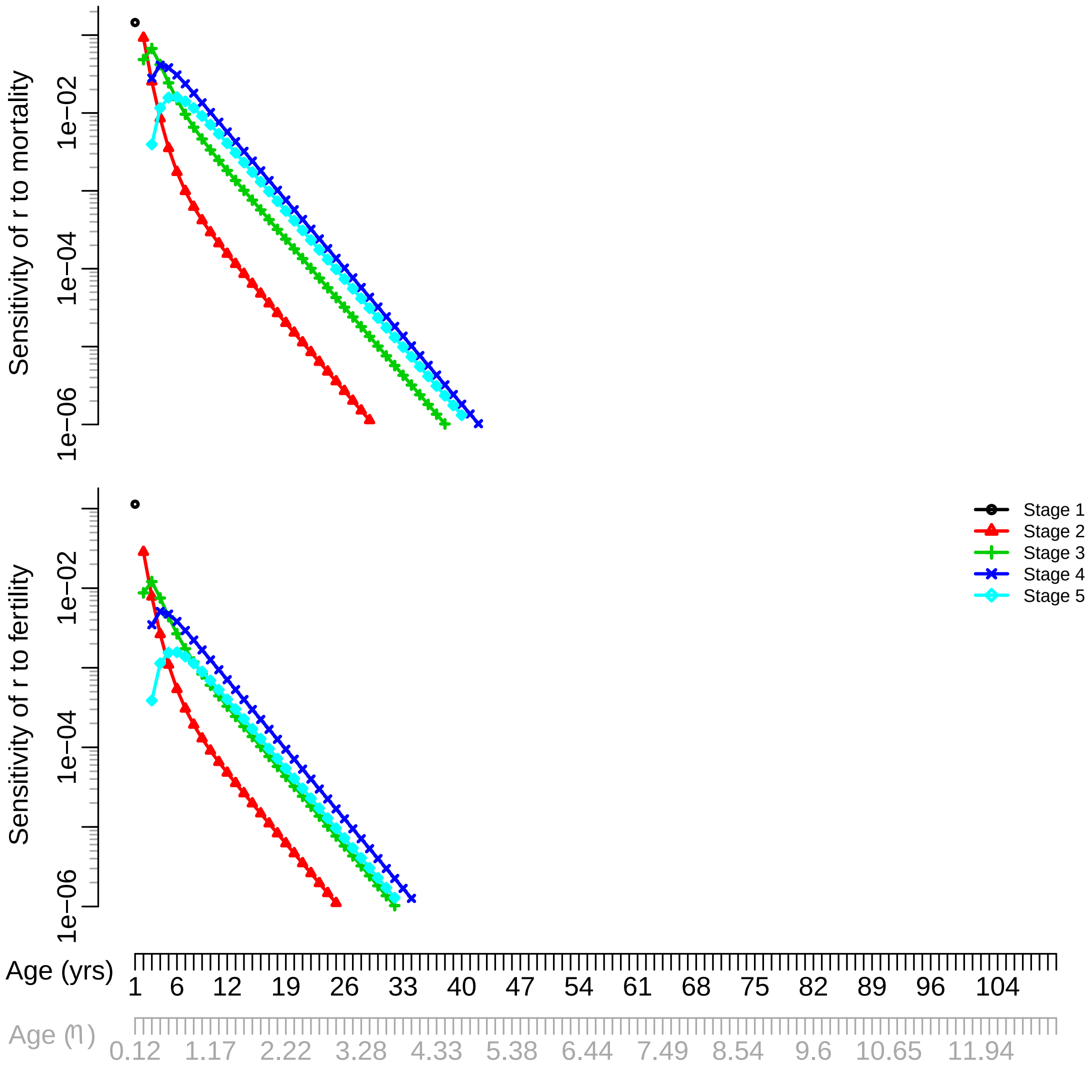

Figure 84: Selection gradients on age×stage-specific mortality and fertility. Top x axis represents age in years, whereas the bottom x axis represents time-scale normalized by the life expectancy (η) of the first non-seedbank stage (FNSS) of the species.

S1.29 *Pterocarpus angolensis*

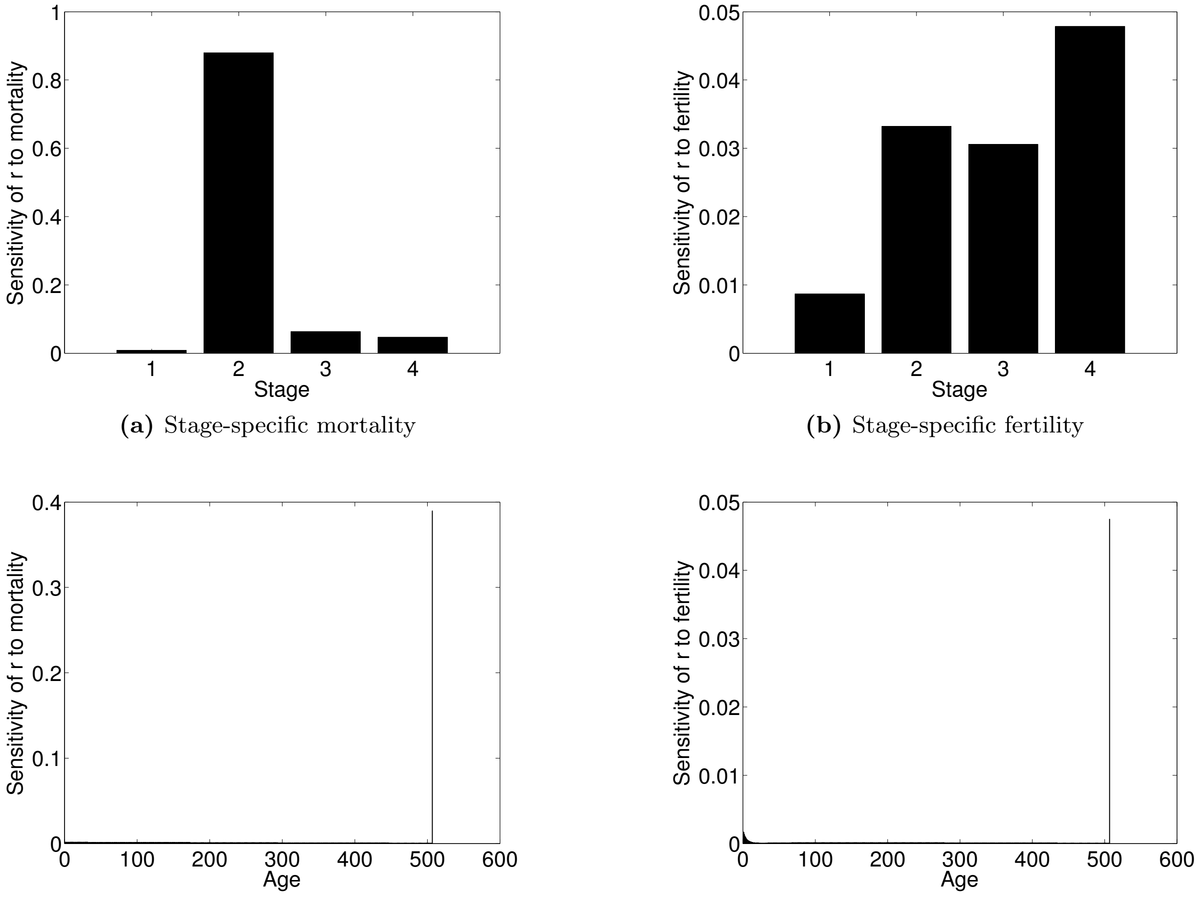

(a) Stage-specific mortality

(b) Stage-specific fertility

(c) Age-specific mortality

(d) Age-specific fertility

Figure 85: Selection gradients on age-specific and stage-specific mortality and fertility.

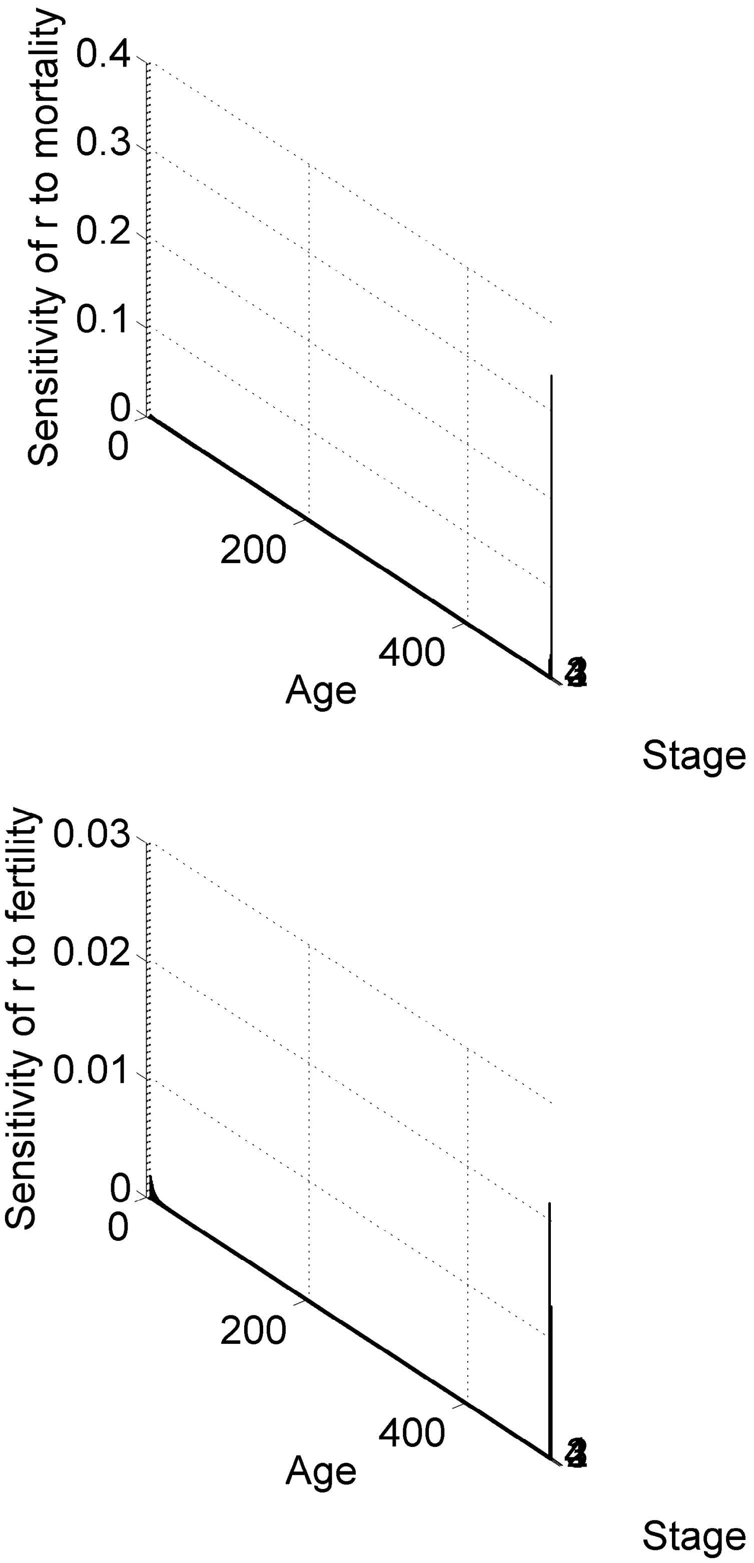

Figure 86: Selection gradients on age×stage-specific mortality and fertility.

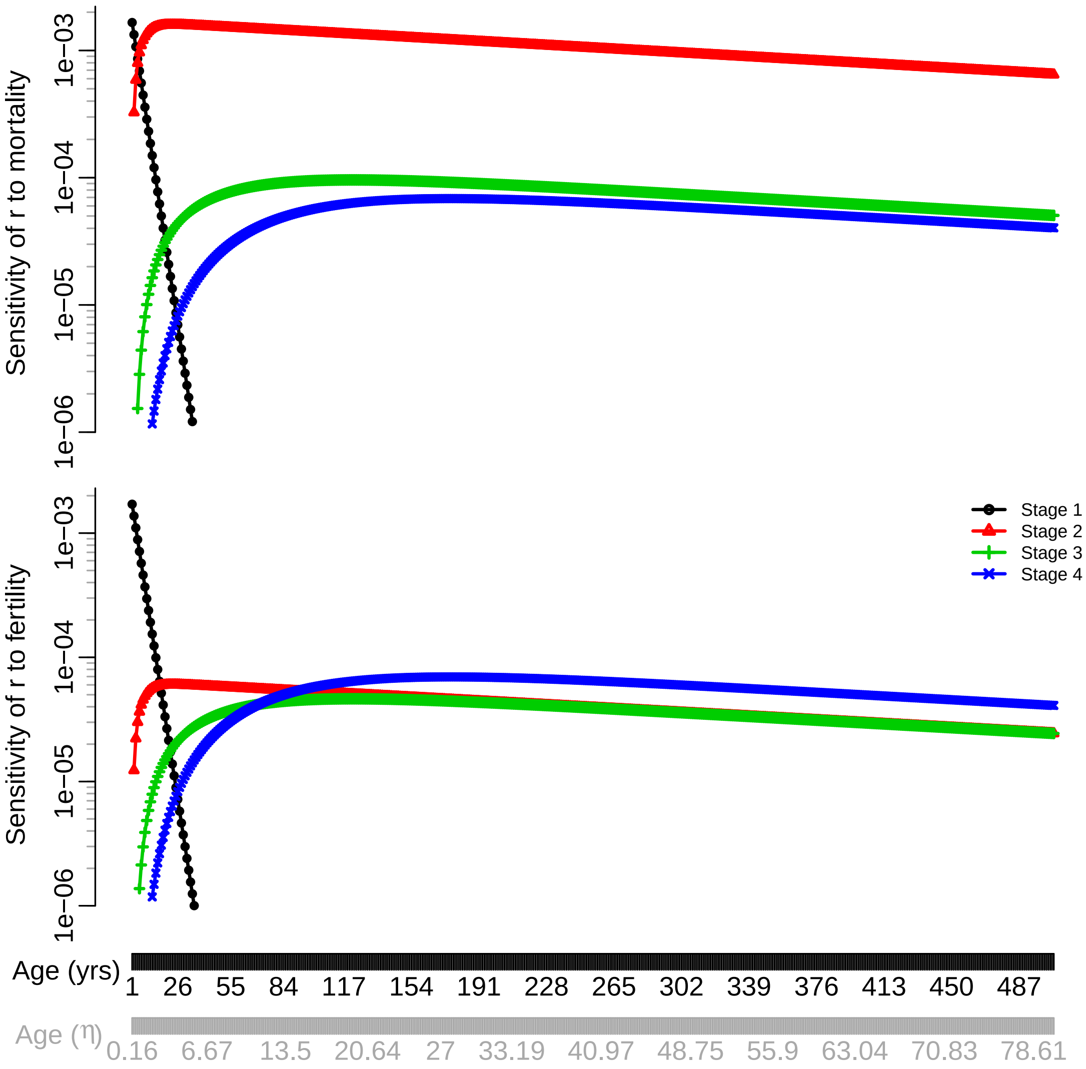

Figure 87: Selection gradients on age×stage-specific mortality and fertility. Top x axis represents age in years, whereas the bottom x axis represents time-scale normalized by the life expectancy (η) of the first non-seedbank stage (FNSS) of the species.

S1.30 *Pterocarya rhoifolia*

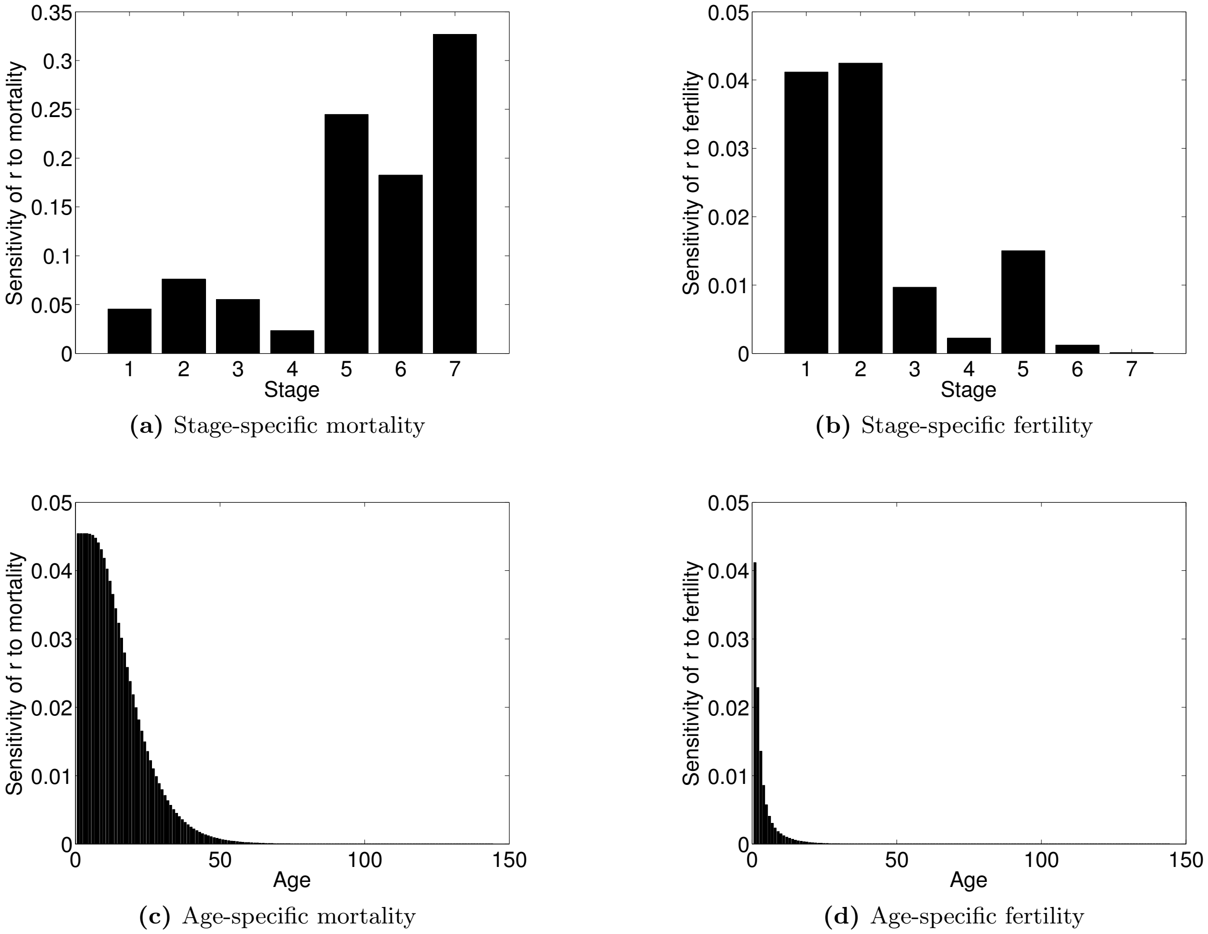

(a) Stage-specific mortality

(b) Stage-specific fertility

(c) Age-specific mortality

(d) Age-specific fertility

Figure 88: Selection gradients on age-specific and stage-specific mortality and fertility.

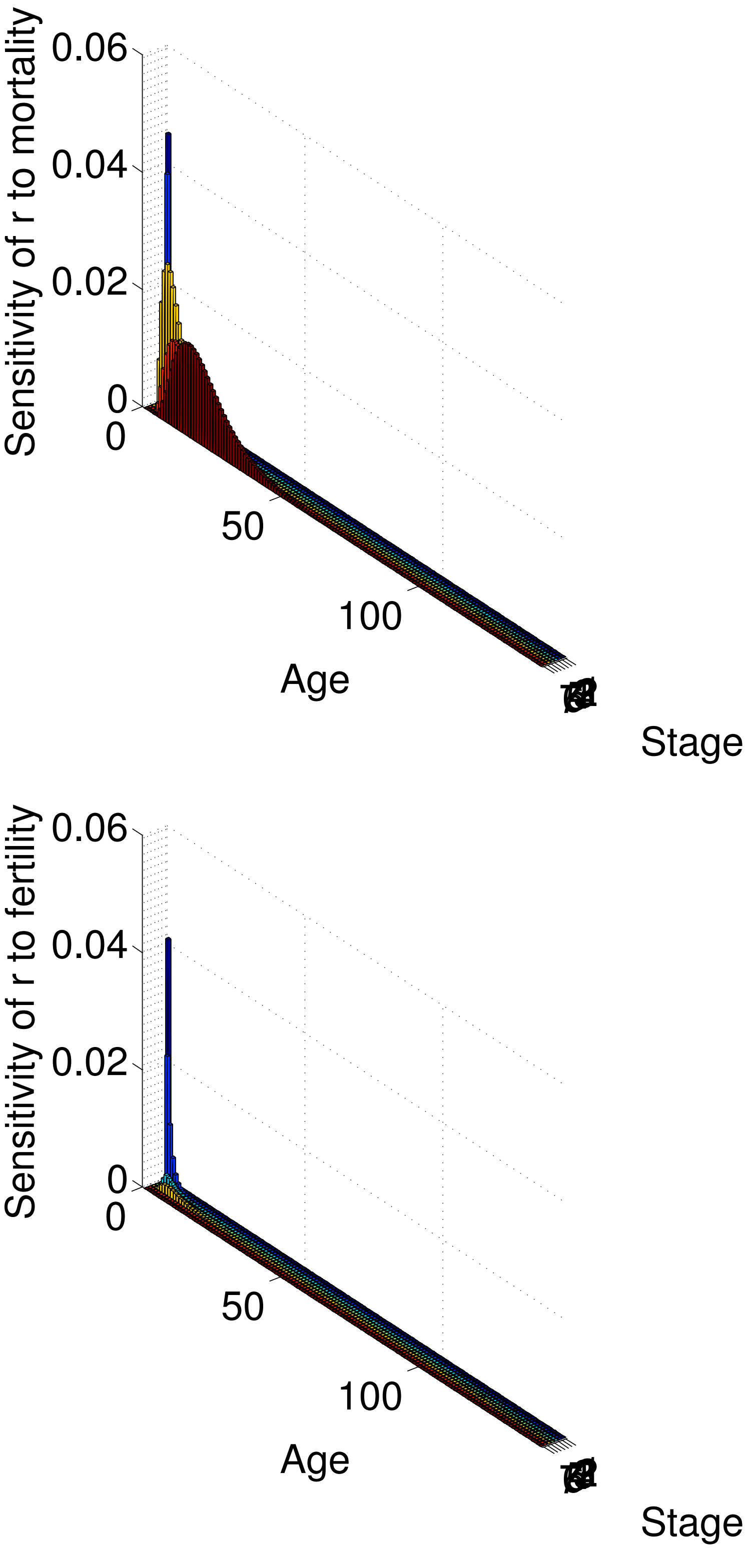

Figure 89: Selection gradients on age×stage-specific mortality and fertility.

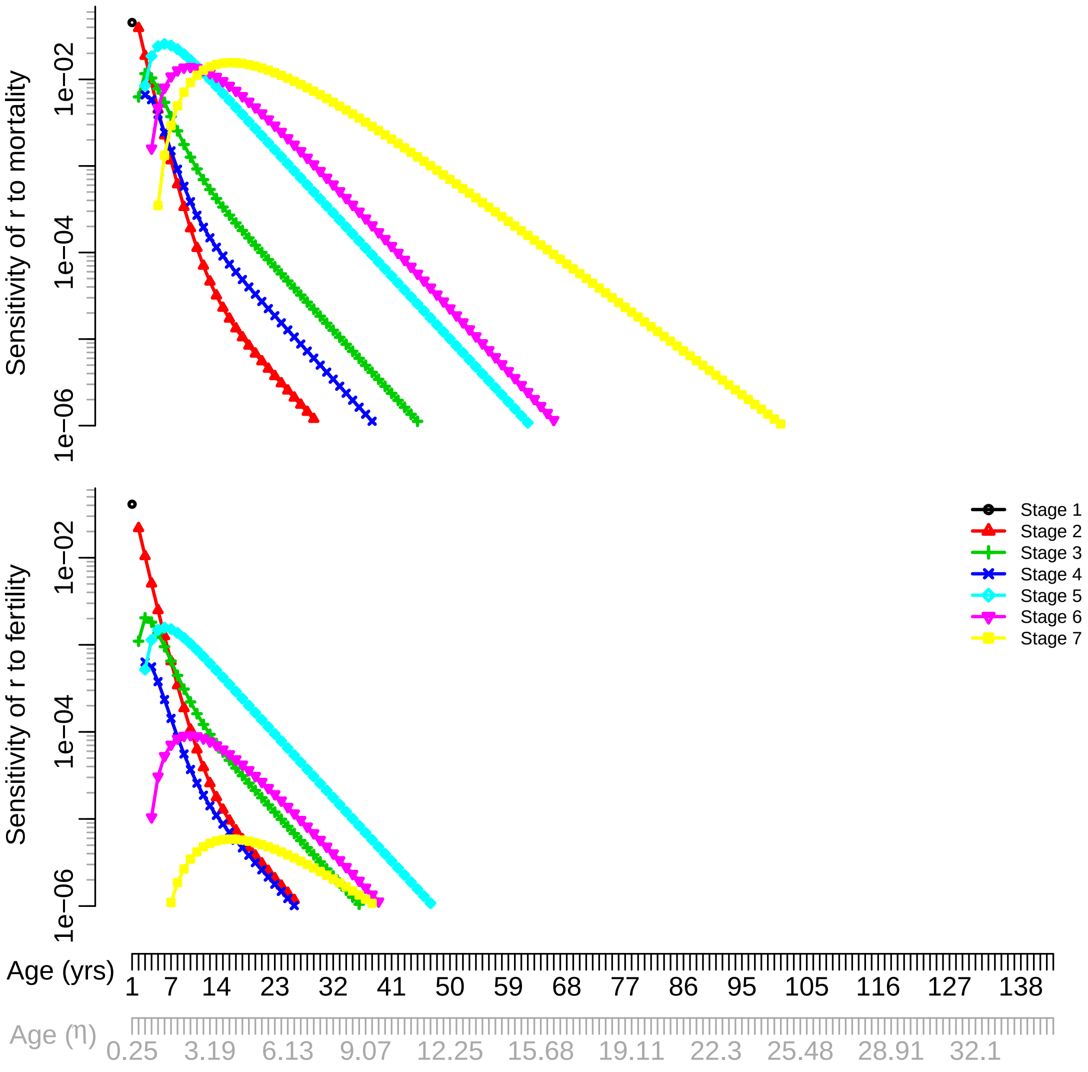

Figure 90: Selection gradients on age×stage-specific mortality and fertility. Top x axis represents age in years, whereas the bottom x axis represents time-scale normalized by the life expectancy (η) of the first non-seedbank stage (FNSS) of the species.

S1.31 *Rhododendron ponticum* (Endangered)

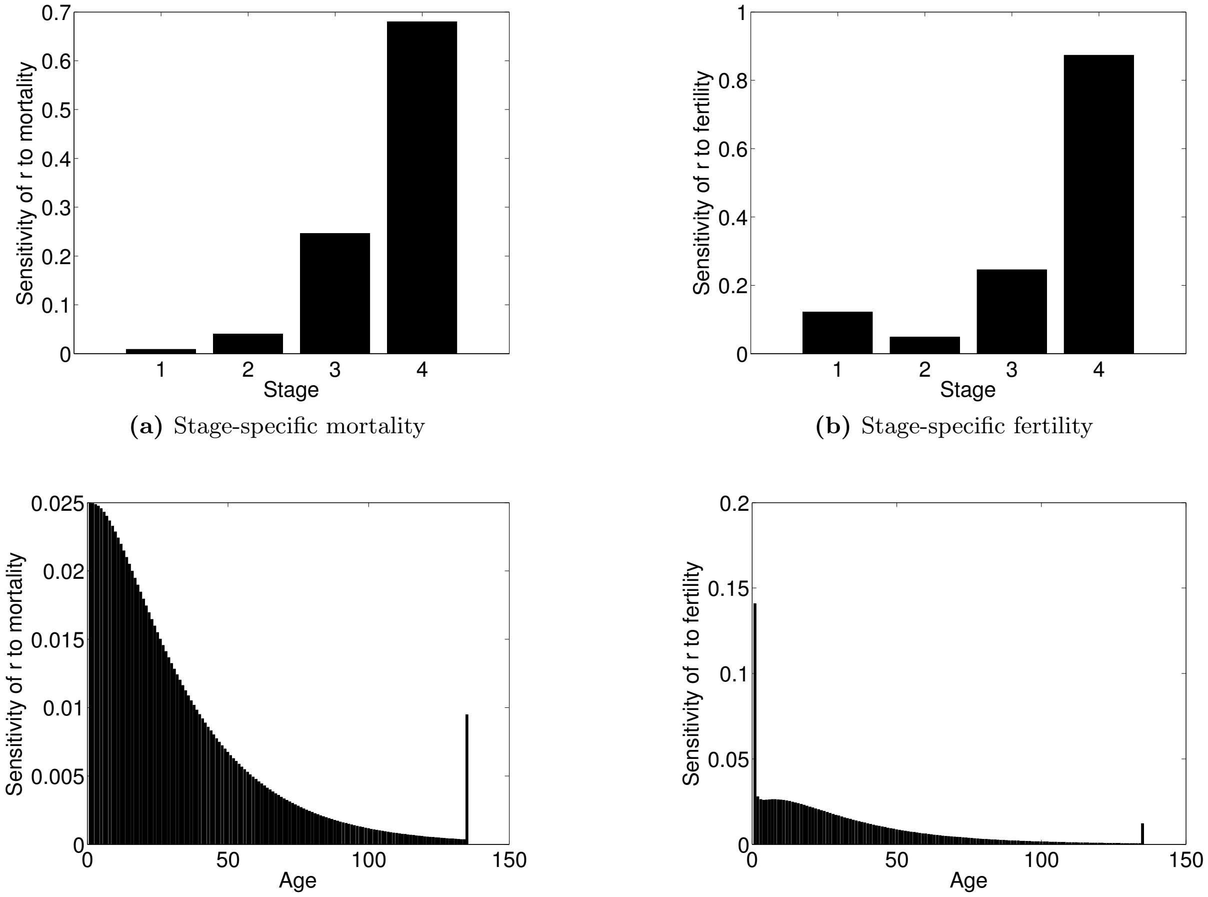

(a) Stage-specific mortality

(b) Stage-specific fertility

(c) Age-specific mortality

(d) Age-specific fertility

Figure 91: Selection gradients on age-specific and stage-specific mortality and fertility.

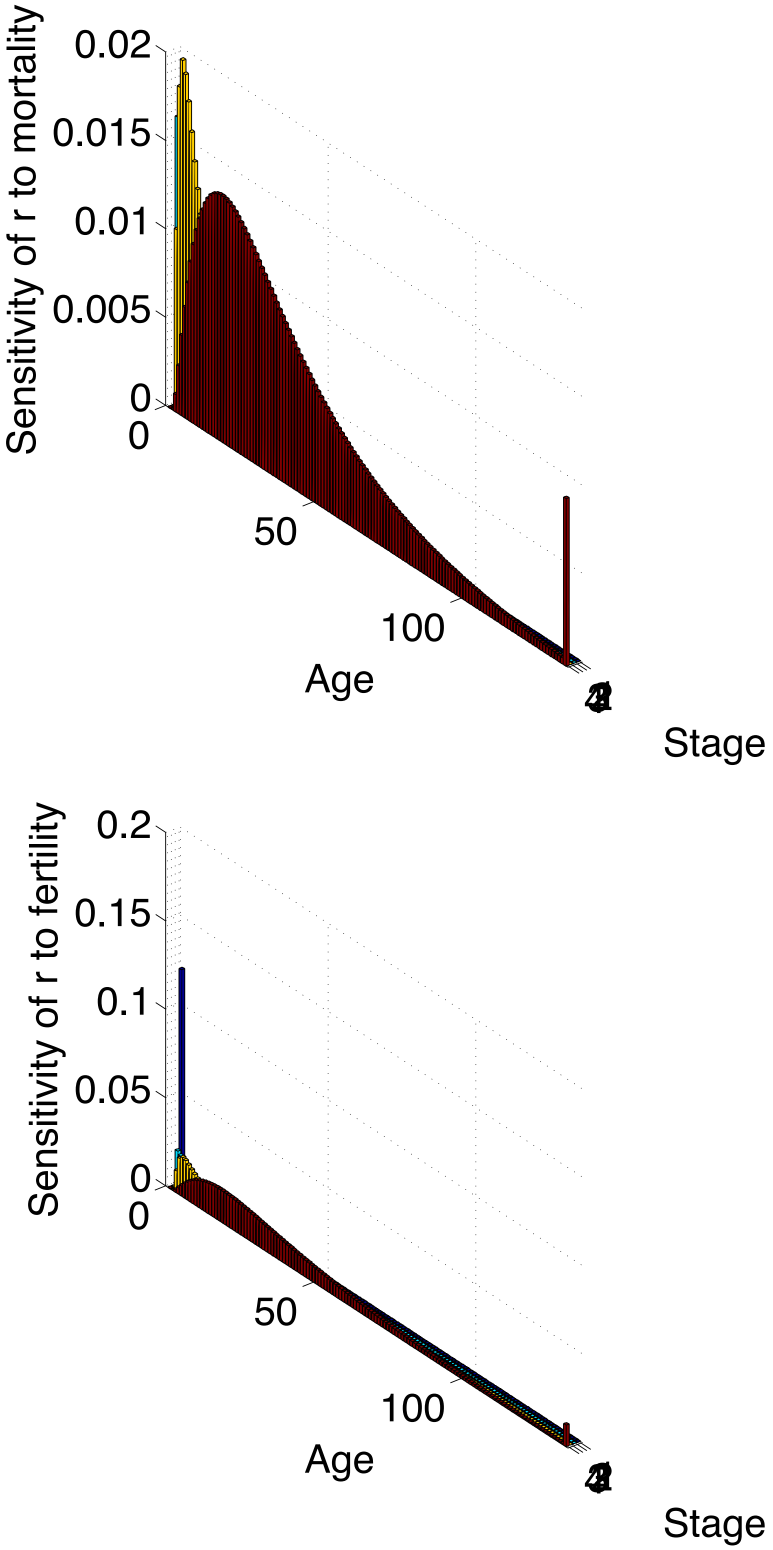

Figure 92: Selection gradients on age×stage-specific mortality and fertility.

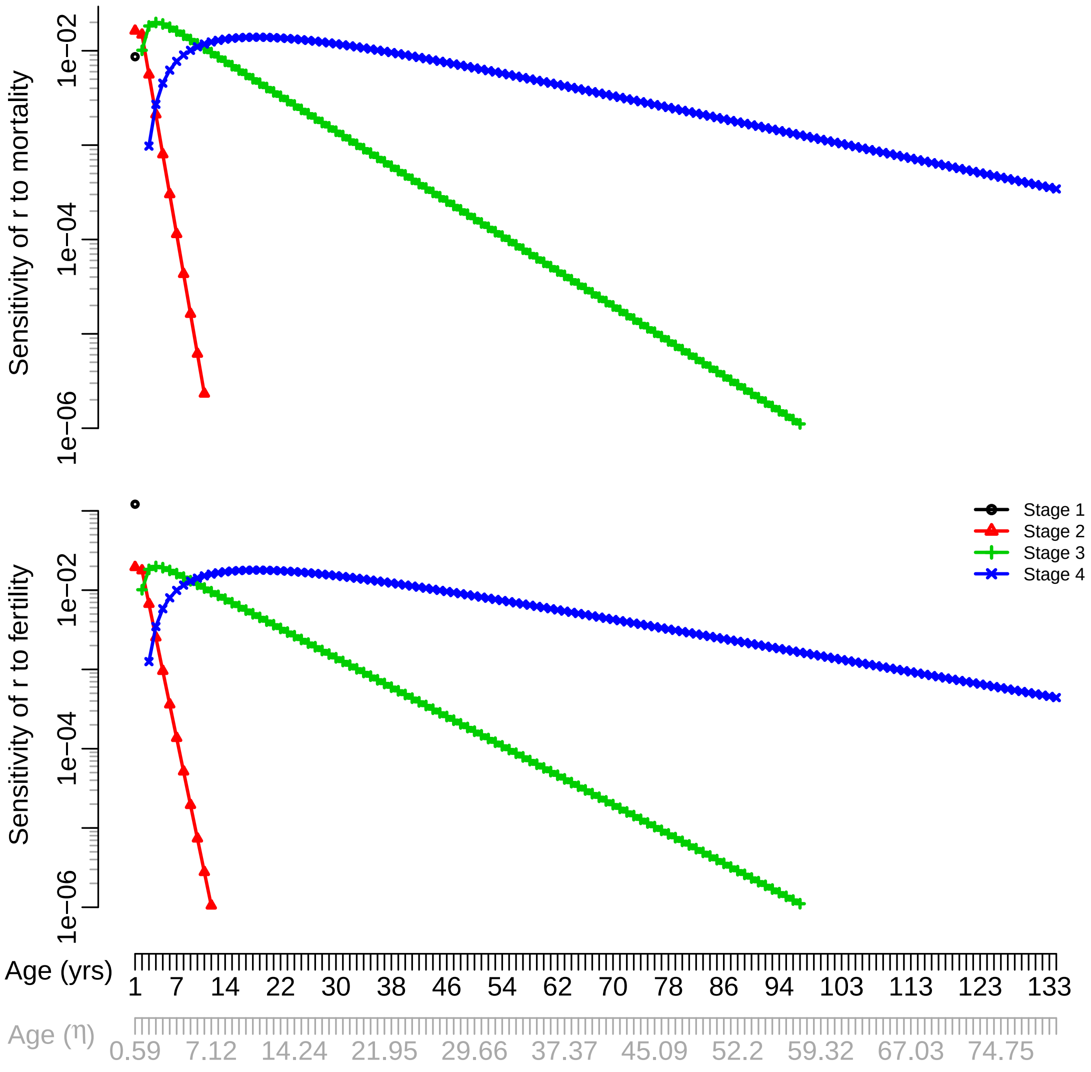

Figure 93: Selection gradients on age×stage-specific mortality and fertility. Top x axis represents age in years, whereas the bottom x axis represents time-scale normalized by the life expectancy (η) of the first non-seedbank stage (FNSS) of the species.

S1.32 *Rhododendron ponticum* (Invasive)

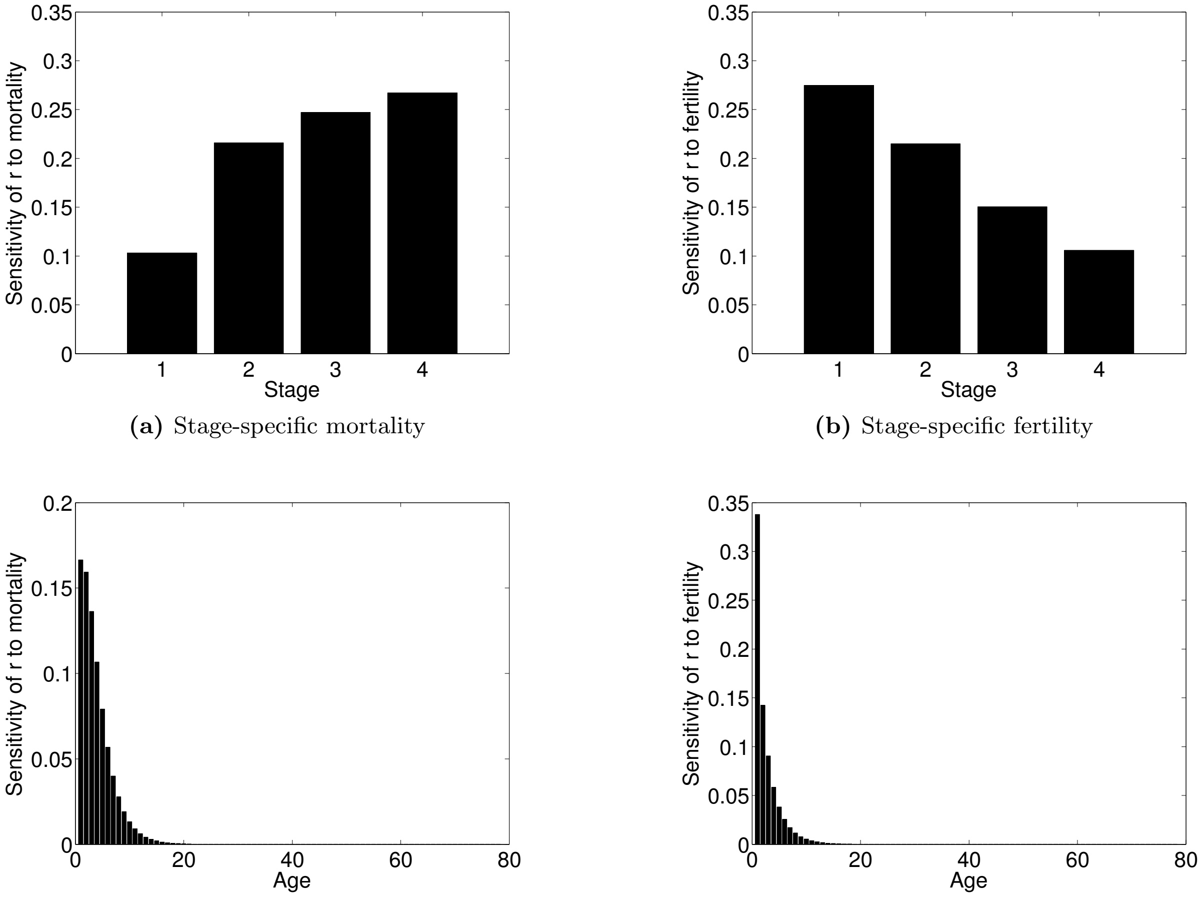

(a) Stage-specific mortality

(b) Stage-specific fertility

(c) Age-specific mortality

(d) Age-specific fertility

Figure 94: Selection gradients on age-specific and stage-specific mortality and fertility.

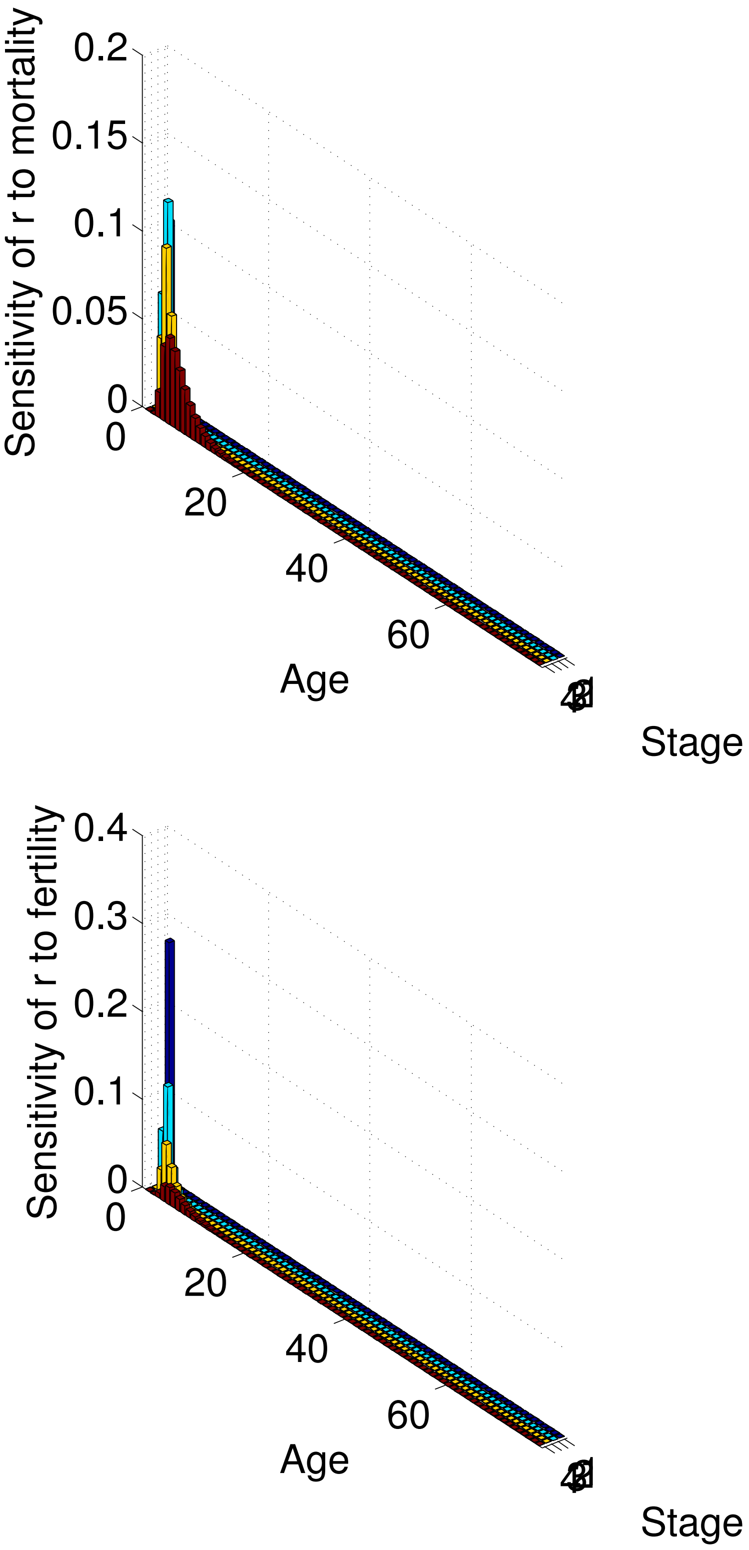

Figure 95: Selection gradients on age×stage-specific mortality and fertility.

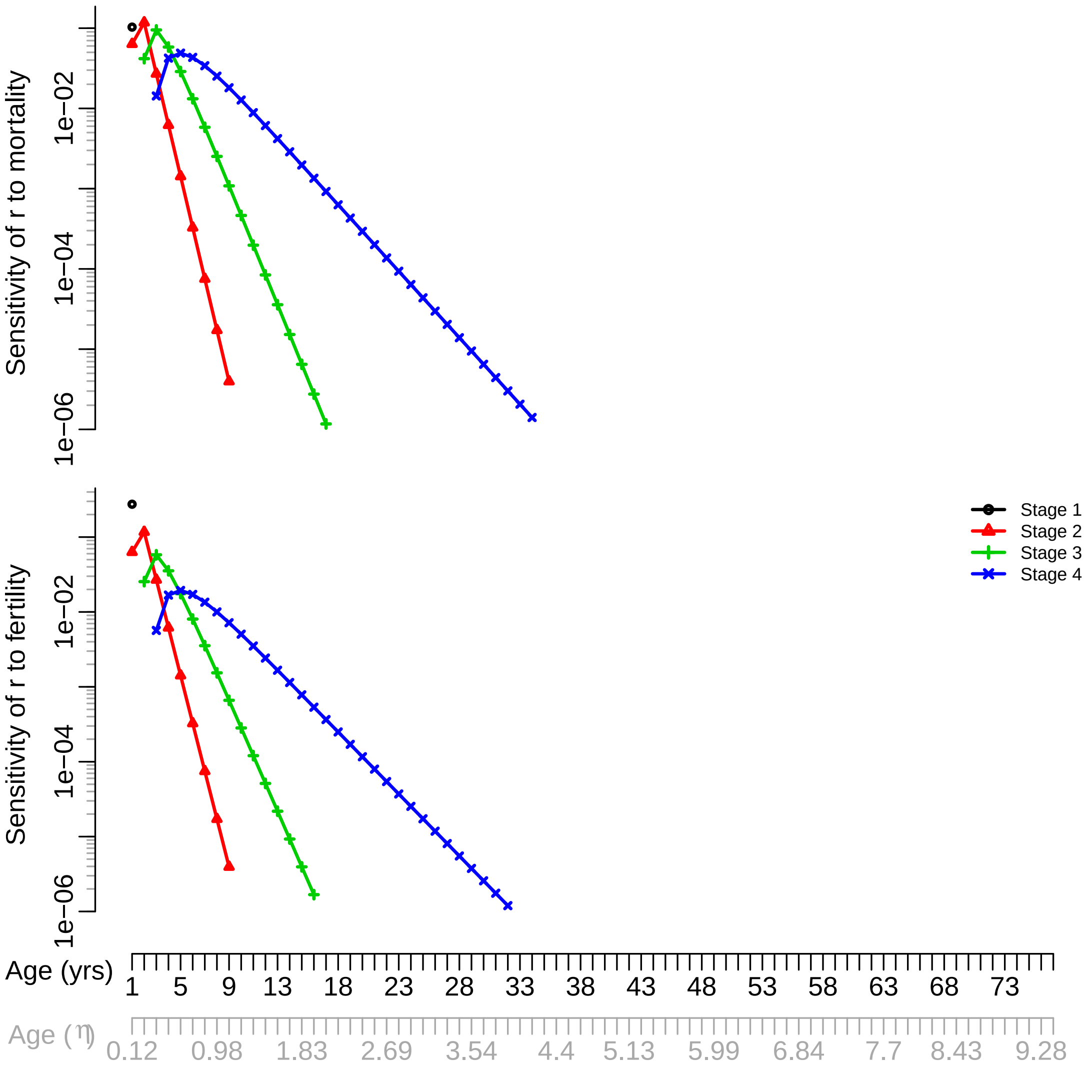

Figure 96: Selection gradients on age×stage-specific mortality and fertility. Top x axis represents age in years, whereas the bottom x axis represents time-scale normalized by the life expectancy (η) of the first non-seedbank stage (FNSS) of the species.

S1.33 *Rhopalostylis sapida*

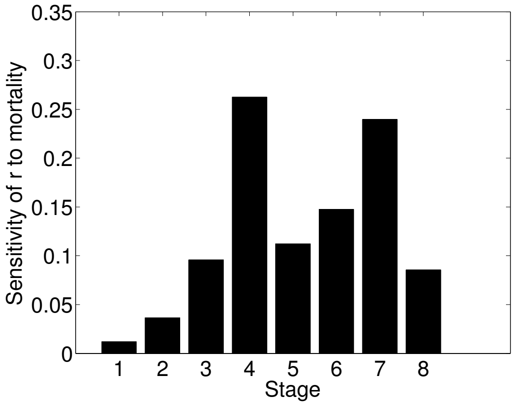

(a) Stage-specific mortality

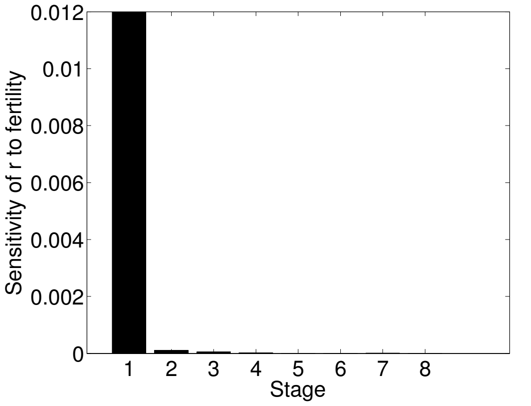

(b) Stage-specific fertility

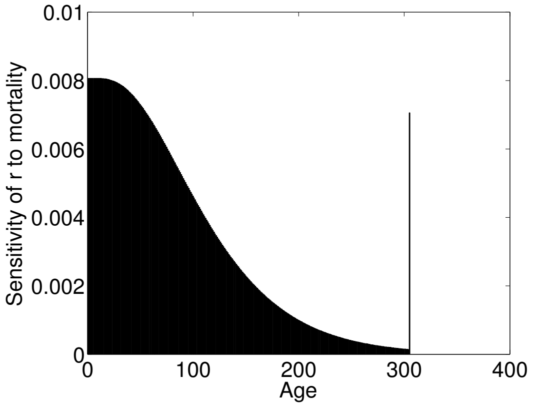

(c) Age-specific mortality

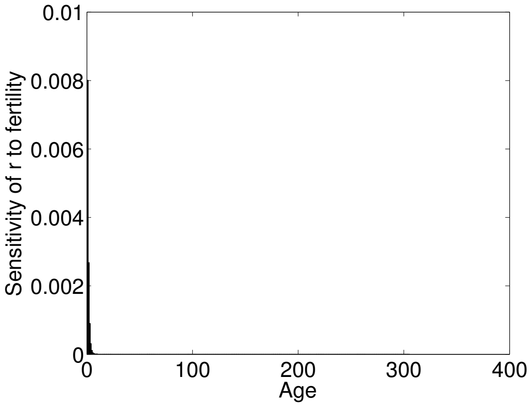

(d) Age-specific fertility

Figure 97: Selection gradients on age-specific and stage-specific mortality and fertility.

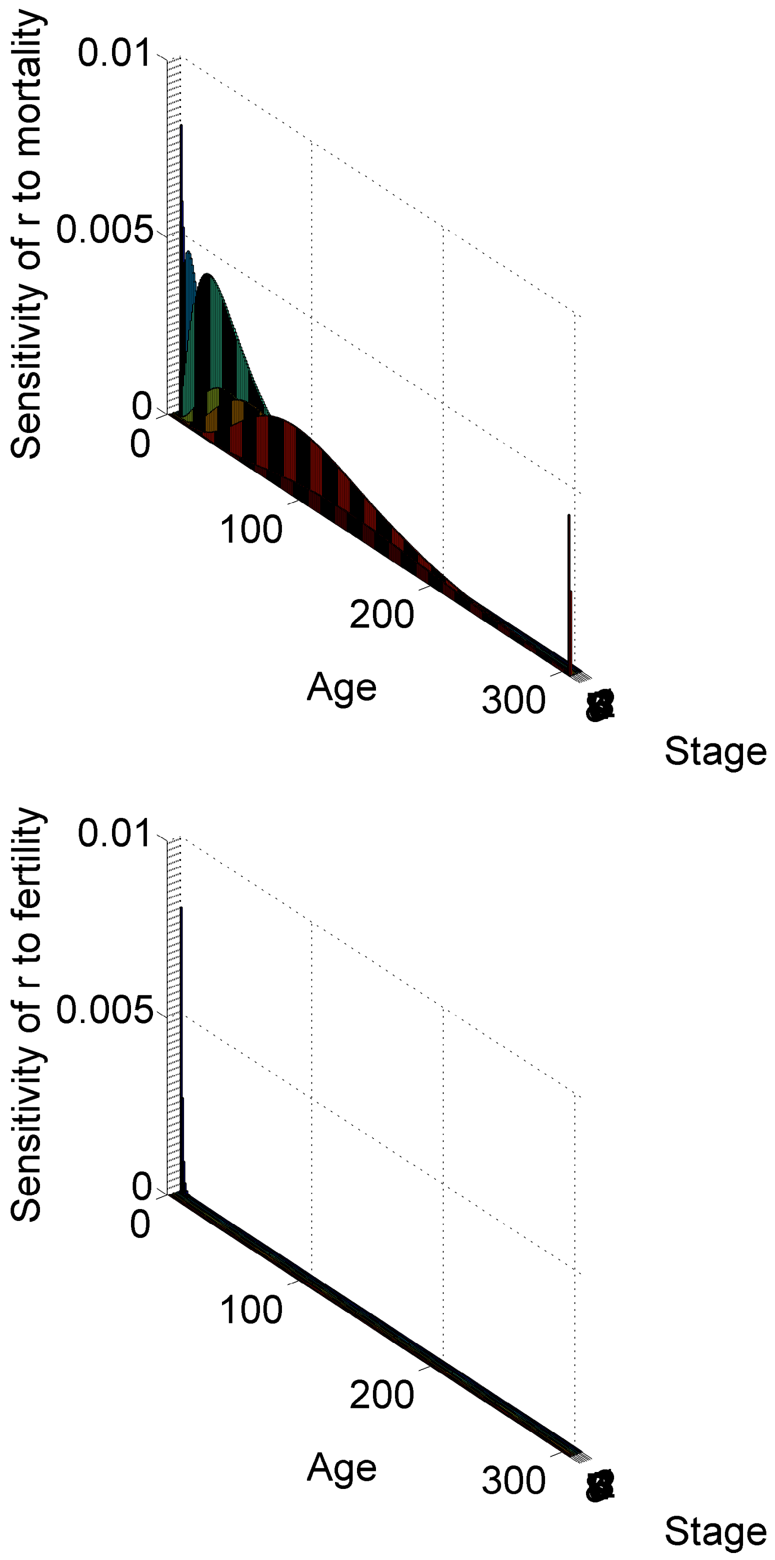

Figure 98: Selection gradients on age×stage-specific mortality and fertility.

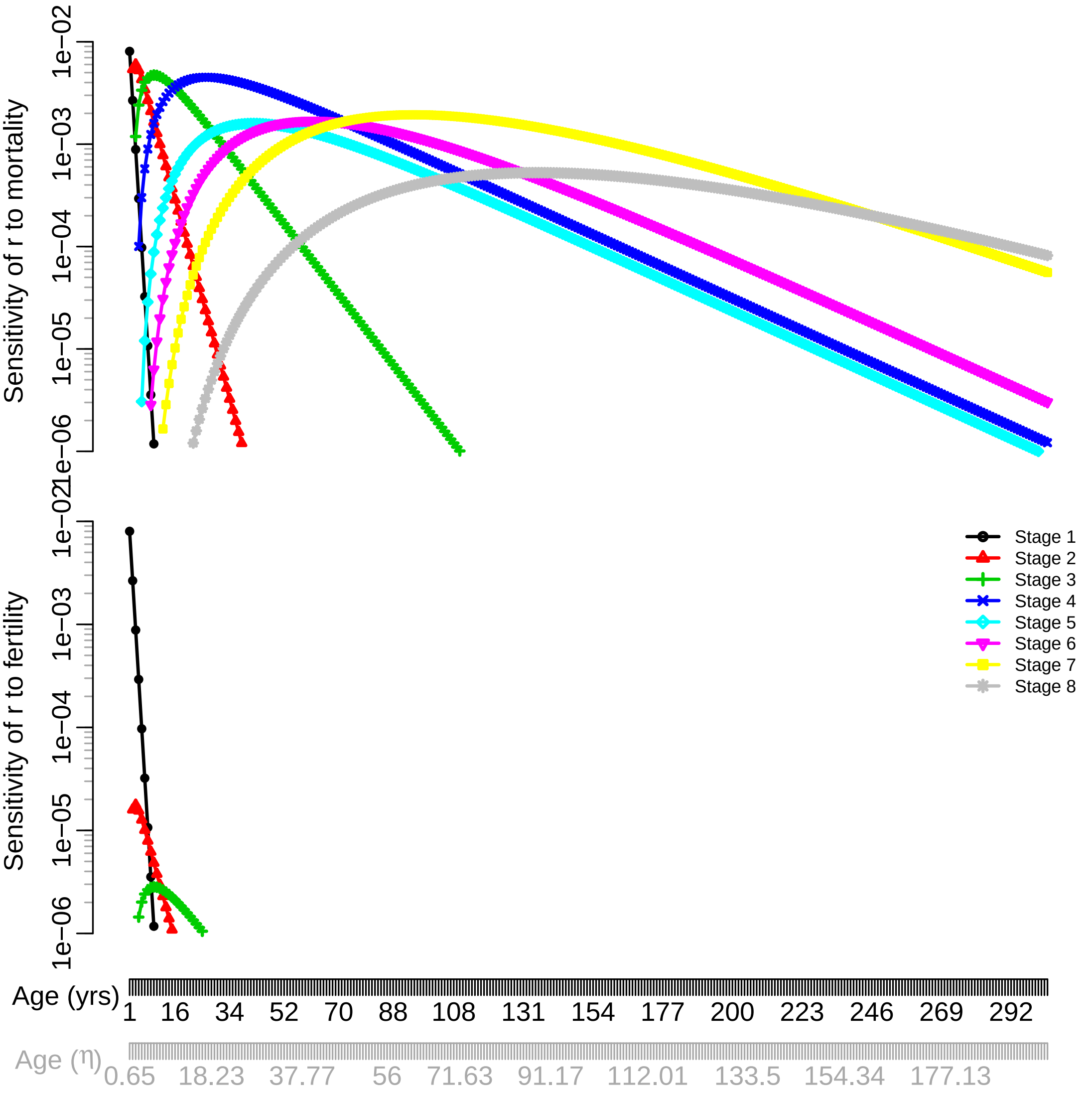

Figure 99: Selection gradients on age×stage-specific mortality and fertility. Top x axis represents age in years, whereas the bottom x axis represents time-scale normalized by the life expectancy (η) of the first non-seedbank stage (FNSS) of the species.

S1.34 *Rourea induta*

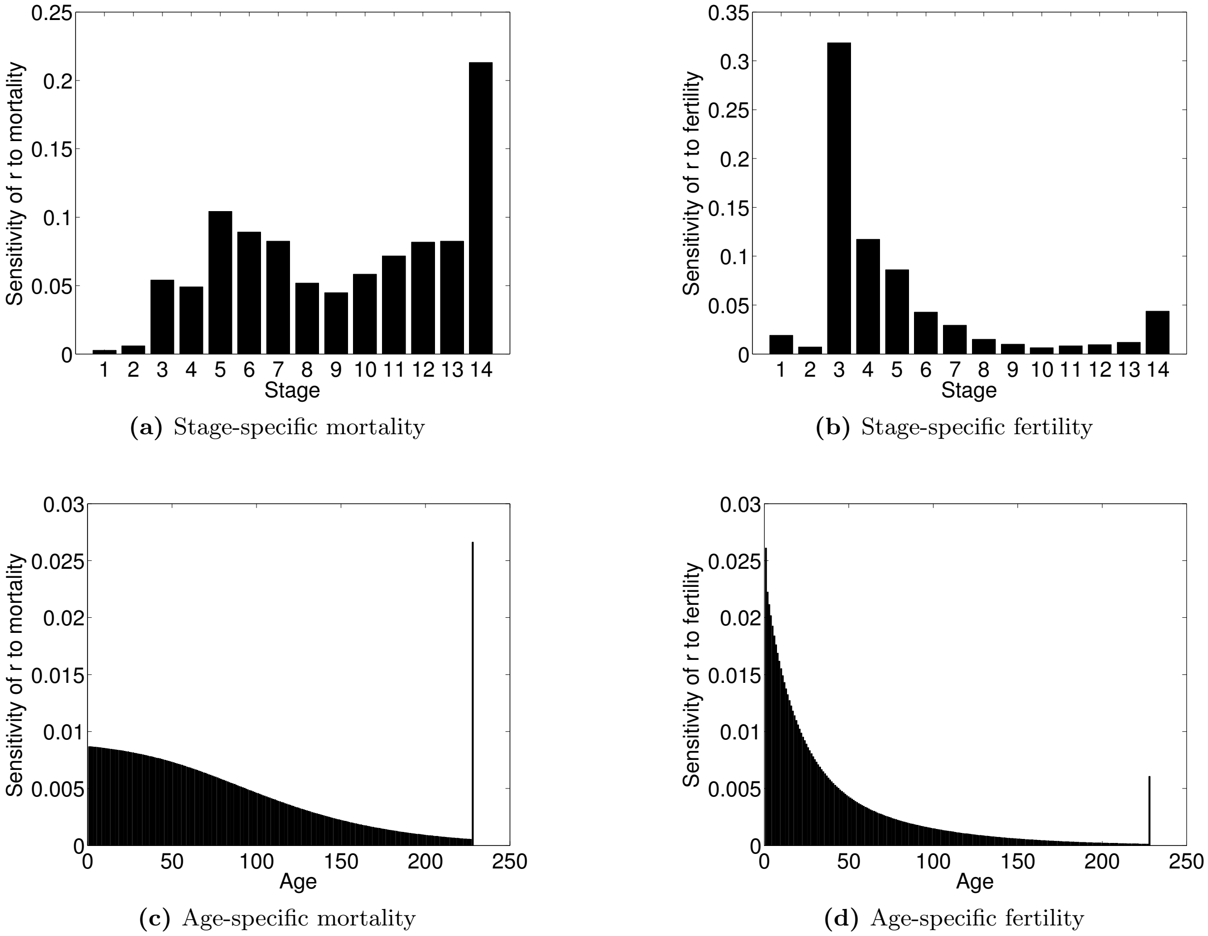

(a) Stage-specific mortality

(b) Stage-specific fertility

(c) Age-specific mortality

(d) Age-specific fertility

Figure 100: Selection gradients on age-specific and stage-specific mortality and fertility.

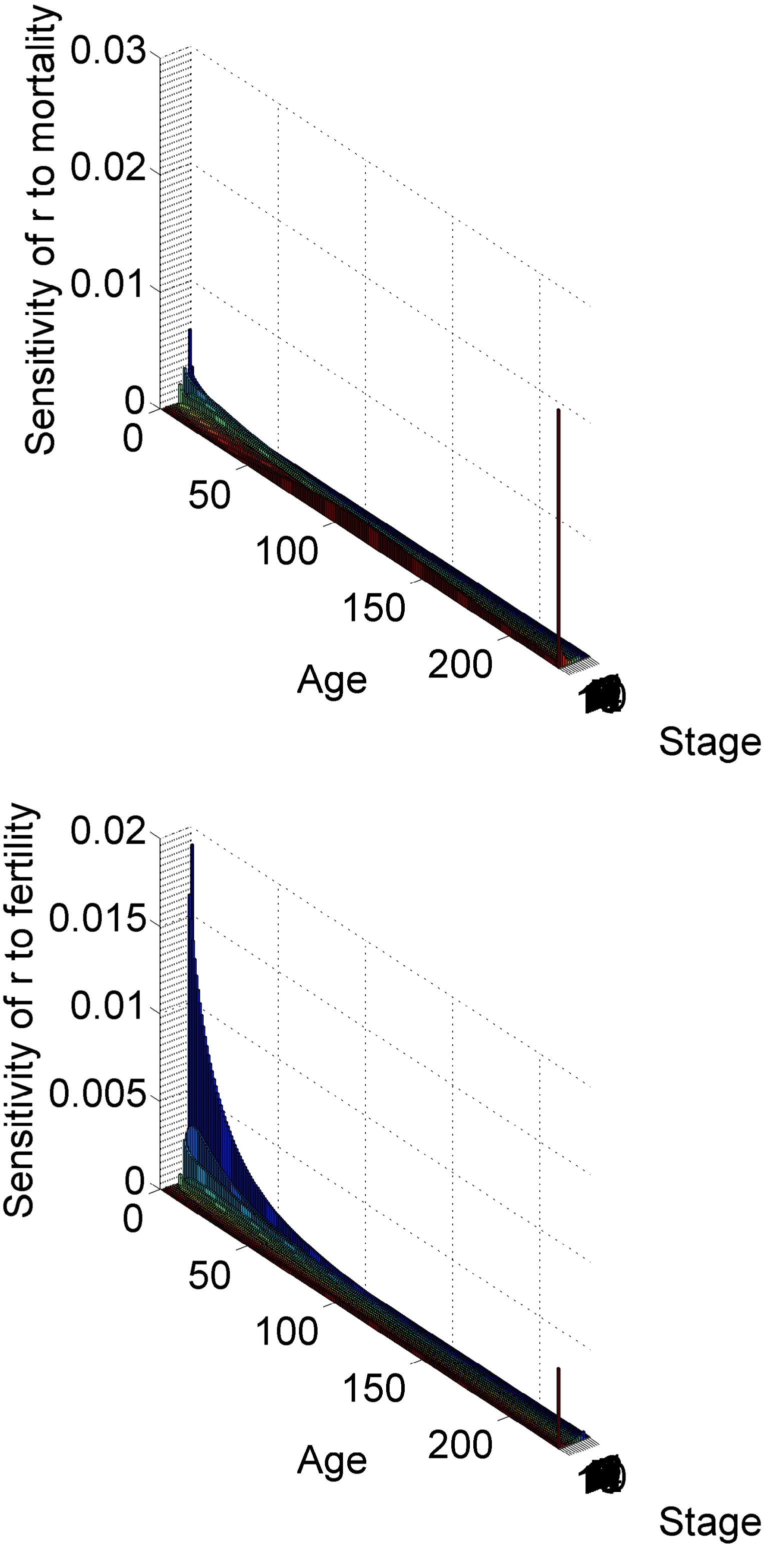

Figure 101: Selection gradients on age×stage-specific mortality and fertility.

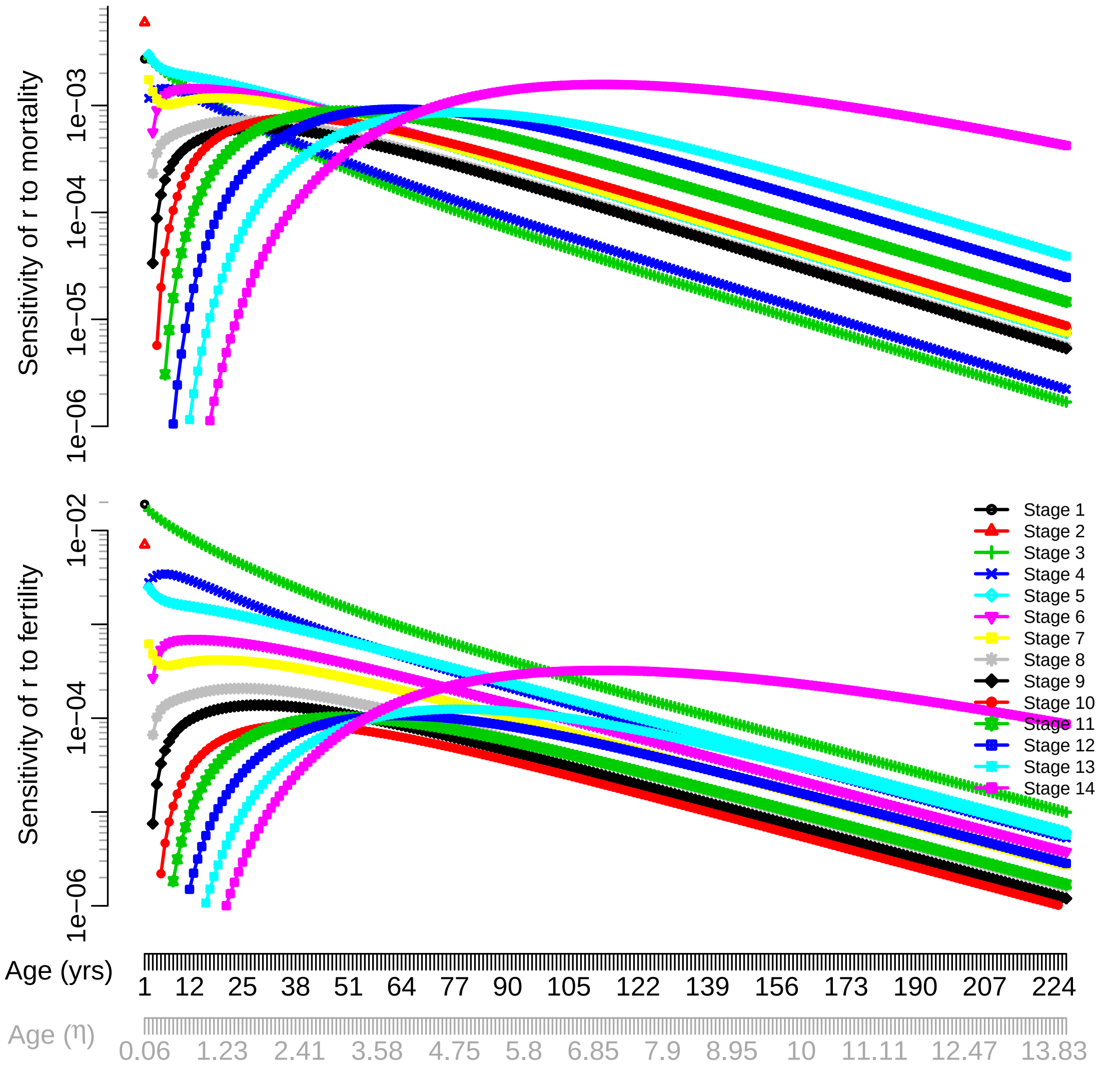

Figure 102: Selection gradients on age×stage-specific mortality and fertility. Top x axis represents age in years, whereas the bottom x axis represents time-scale normalized by the life expectancy (η) of the first non-seedbank stage (FNSS) of the species.

S1.35 *Tillandsia recurvata*

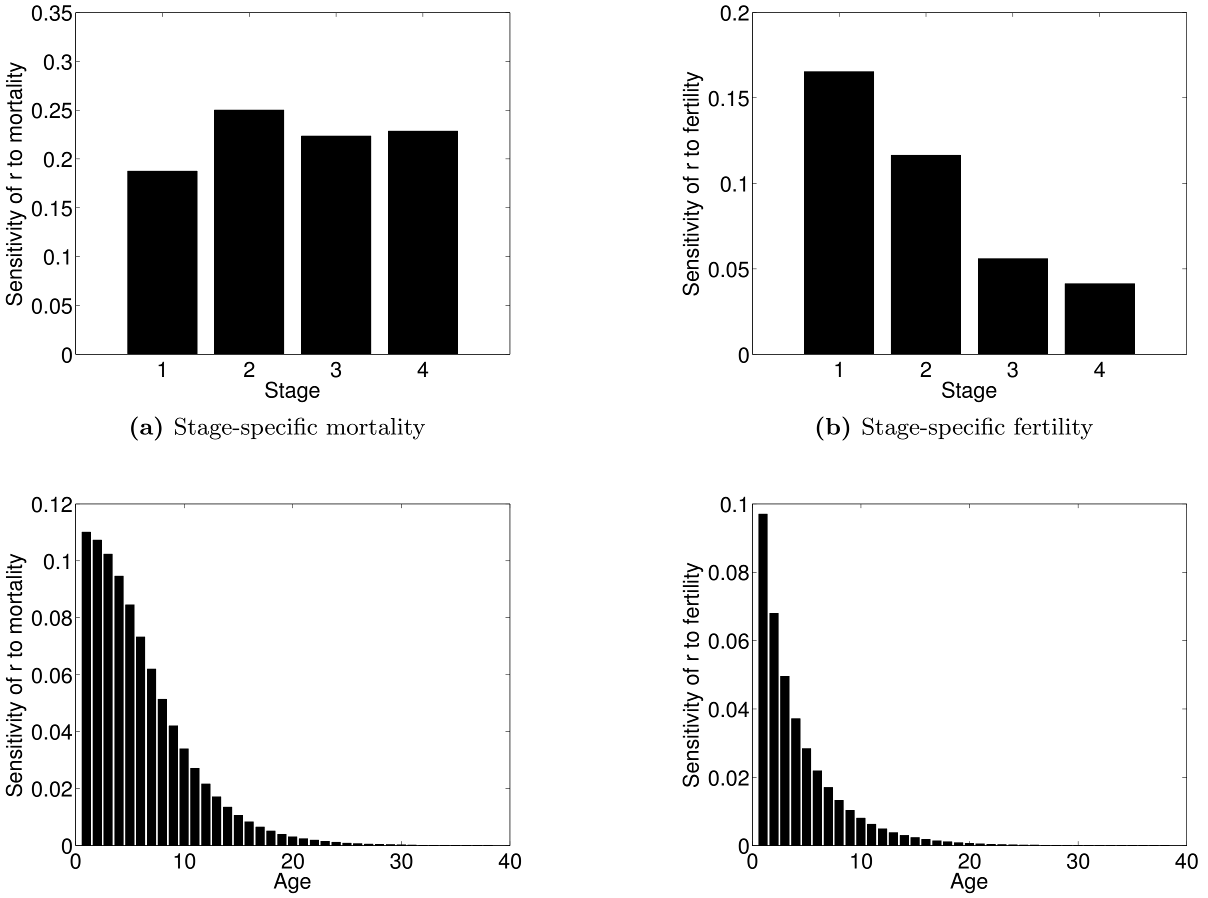

(a) Stage-specific mortality

(b) Stage-specific fertility

(c) Age-specific mortality

(d) Age-specific fertility

Figure 103: Selection gradients on age-specific and stage-specific mortality and fertility.

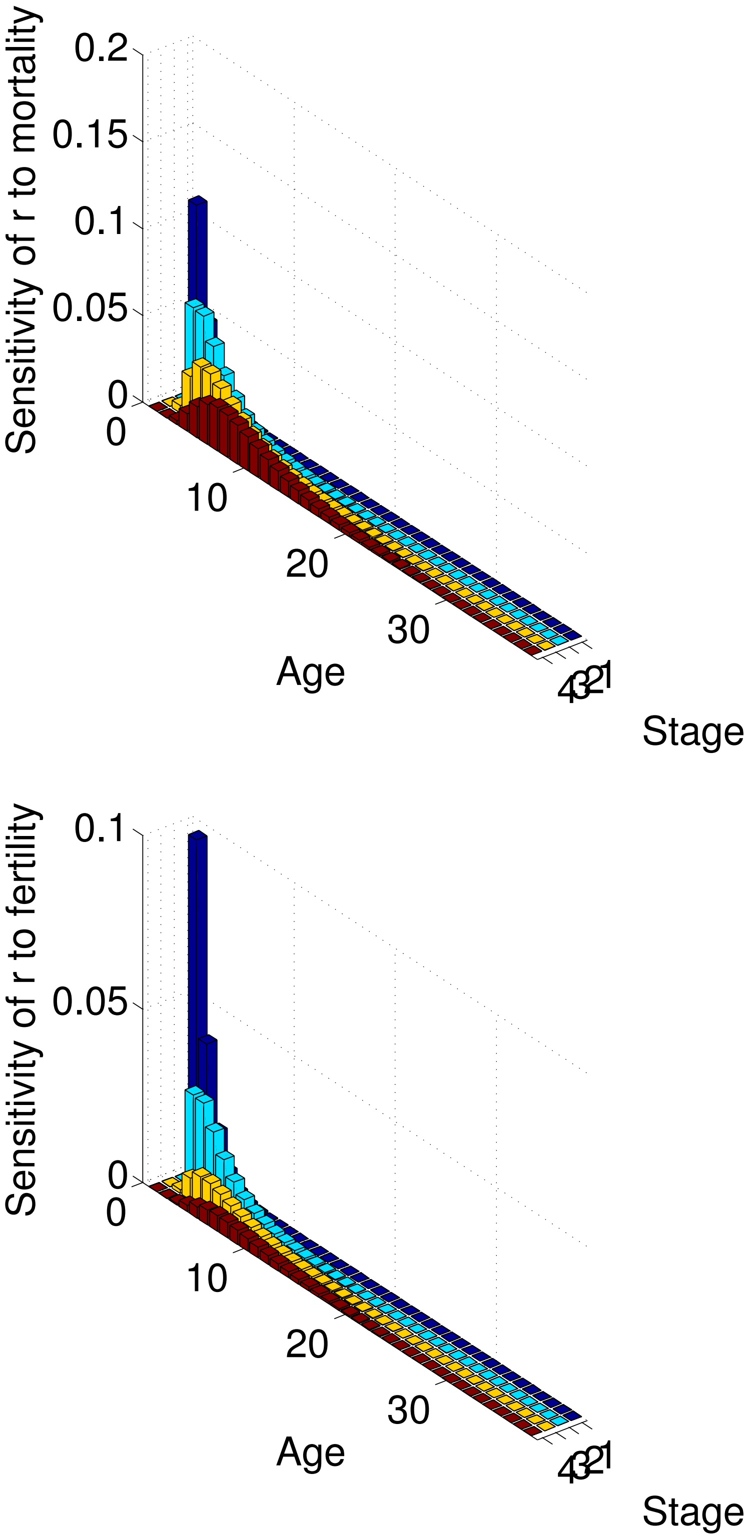

Figure 104: Selection gradients on age×stage-specific mortality and fertility.

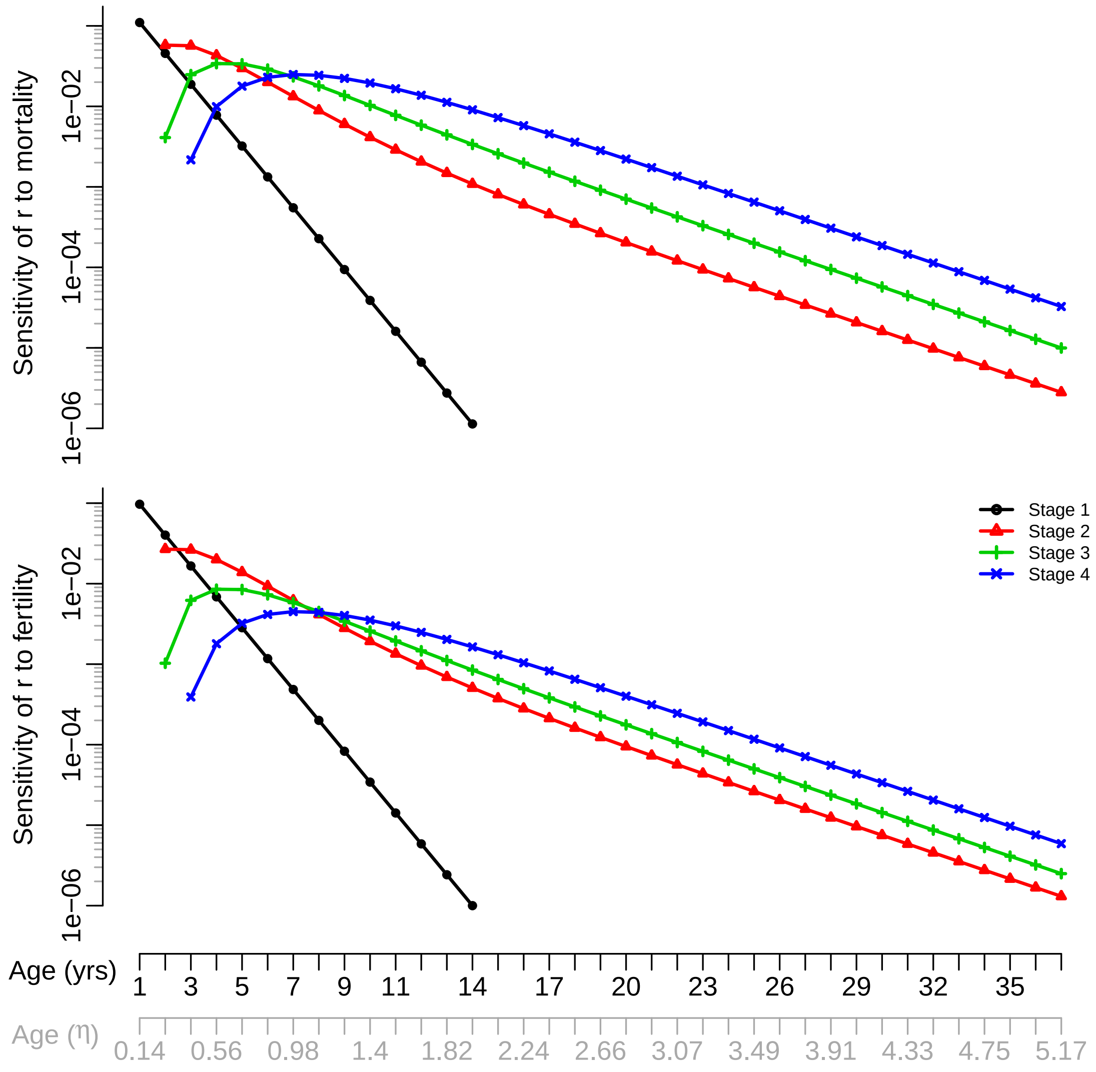

Figure 105: Selection gradients on age×stage-specific mortality and fertility. Top x axis represents age in years, whereas the bottom x axis represents time-scale normalized by the life expectancy (η) of the first non-seedbank stage (FNSS) of the species.

S1.36 *Viburnum furcatum*

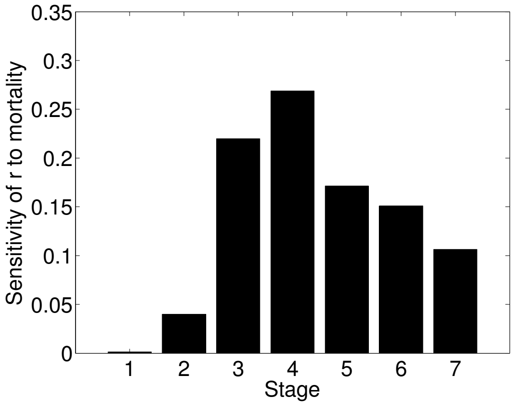

(a) Stage-specific mortality

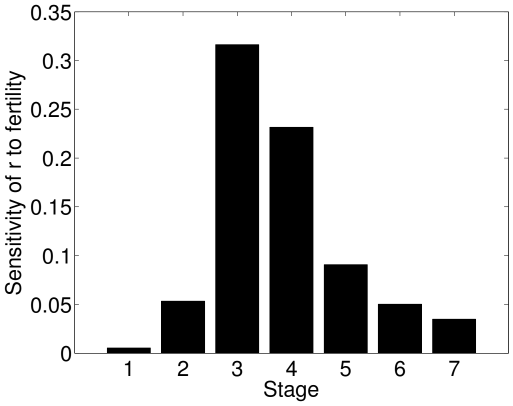

(b) Stage-specific fertility

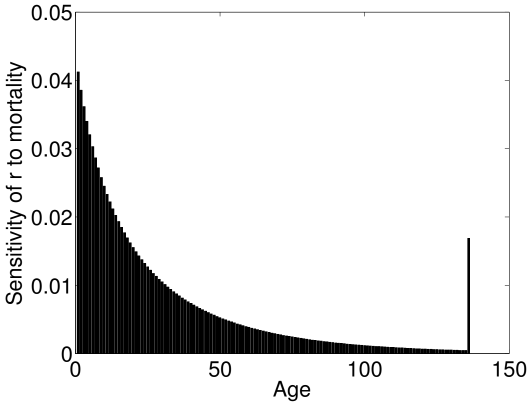

(c) Age-specific mortality

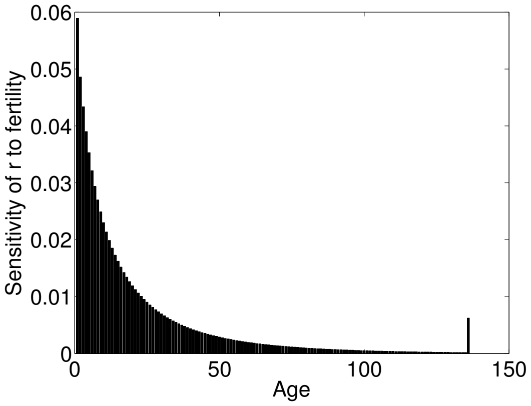

(d) Age-specific fertility

Figure 106: Selection gradients on age-specific and stage-specific mortality and fertility.

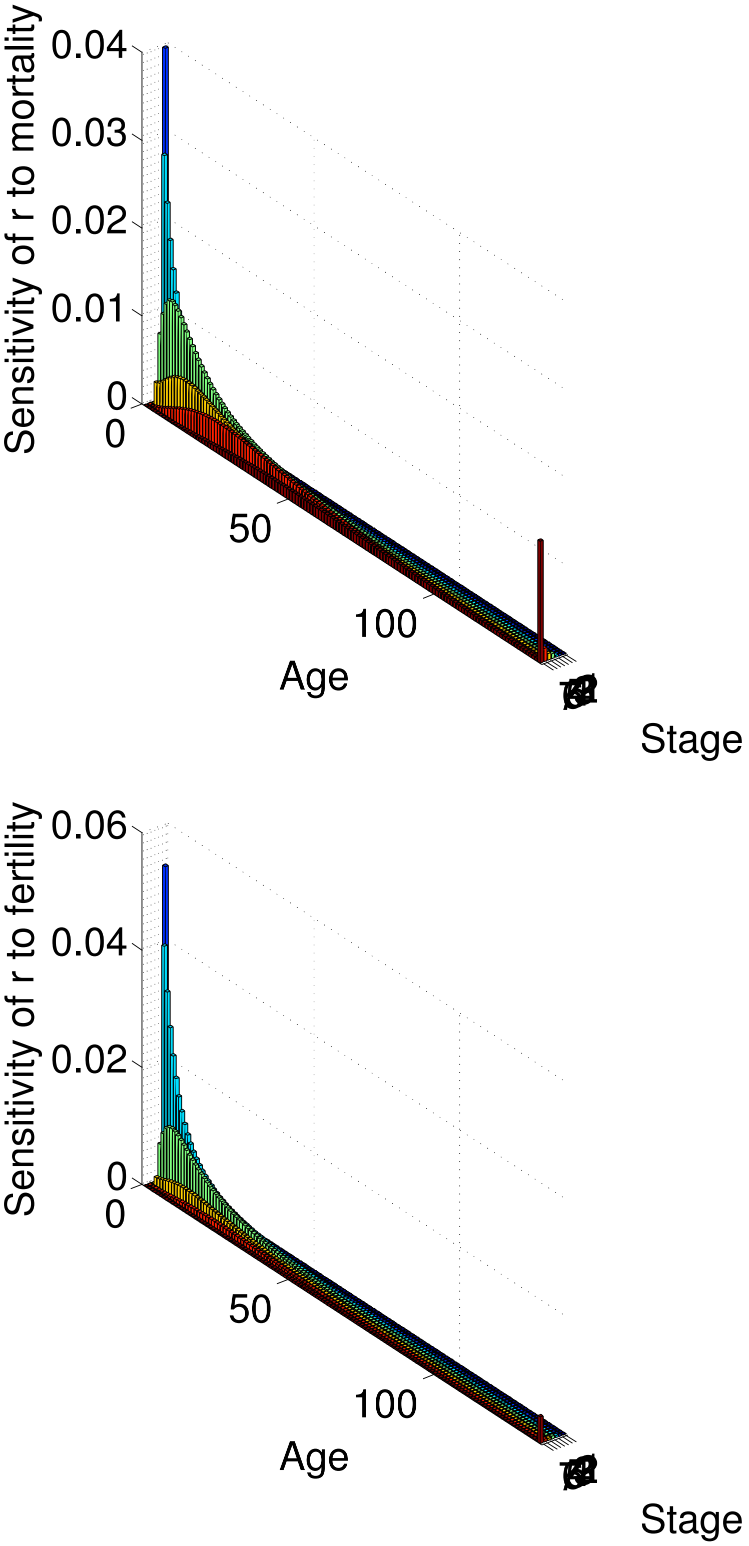

Figure 107: Selection gradients on age×stage-specific mortality and fertility.

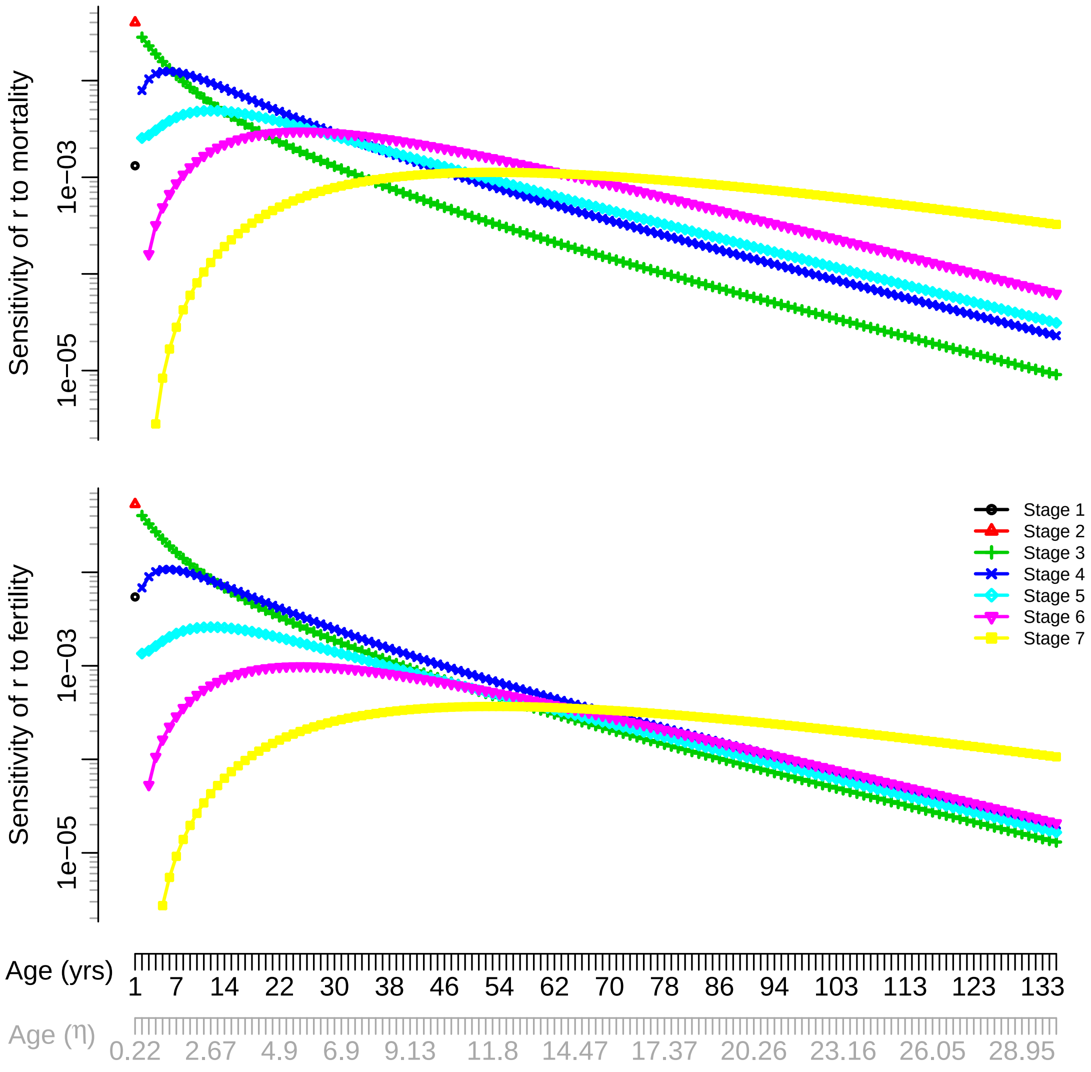

Figure 108: Selection gradients on age×stage-specific mortality and fertility. Top x axis represents age in years, whereas the bottom x axis represents time-scale normalized by the life expectancy (η) of the first non-seedbank stage (FNSS) of the species.
